# Sugar modified pyrimido[4,5-*b*]indole nucleosides: synthesis and antiviral activity†
†Electronic supplementary information (ESI) available: Experimental part and characterization data for all new compounds, table with HPLC purities of final compounds, details of biological assays and copies of NMR spectra. See DOI: 10.1039/c7md00319f


**DOI:** 10.1039/c7md00319f

**Published:** 2017-08-25

**Authors:** Juraj Konč, Michal Tichý, Radek Pohl, Jan Hodek, Petr Džubák, Marián Hajdúch, Michal Hocek

**Affiliations:** a Institute of Organic Chemistry and Biochemistry , Czech Academy of Sciences , Flemingovo nam. 2 , CZ-16610 Prague 6 , Czech Republic . Email: hocek@uochb.cas.cz; b Institute of Molecular and Translational Medicine , Palacky University and University Hospital in Olomouc , Faculty of Medicine and Dentistry , Hněvotínská 5 , CZ-775 15 Olomouc , Czech Republic; c Department of Organic Chemistry , Faculty of Science , Charles University in Prague , Hlavova 8 , CZ-12843 Prague 2 , Czech Republic

## Abstract

Three types of sugar modified pyrimido[4,5-*b*]indole nucleosides (2′-deoxy-2′-fluororibo-, 2′-deoxy-2′-fluoroarabino- and arabinonucleosides) were synthesized by glycosylation of 4,6-dichloropyrimido[4,5-*b*]indole followed by modification of sugar moiety and introduction of substituents into position 4 by cross-coupling reactions or nucleophilic substitutions. Some 2′-fluororibo- and 2′-fluoroarabinonucleosides displayed interesting anti-HCV activities (IC_50_ = 1.6–20 μM) and the latter compounds also some anti-dengue activities (IC_50_ = 10.8–40 μM).

## Abstract 2

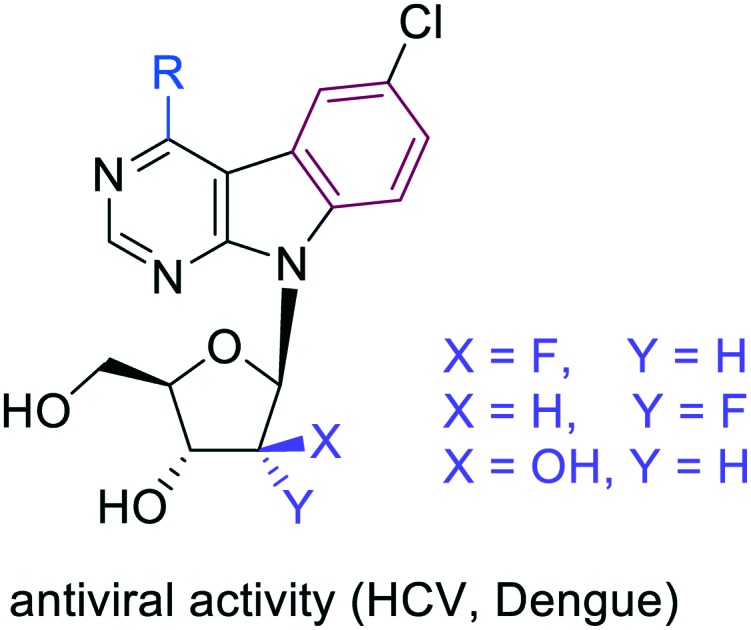
Sugar-modified pyrimido[4,5-*b*]indole nucleosides (2′-deoxy-2′-fluororibo-, 2′-deoxy-2′-fluoroarabino- and arabinonucleosides) were found to be low micromolar antivirals against HCV or dengue.

## Introduction

Modified nucleosides are one of the most important classes of antivirals.^1^,^2^ They mostly work through intracellular phosphorylation to the corresponding nucleoside triphosphates (NTPs), which inhibit the viral DNA or RNA polymerase and/or terminate the DNA or RNA chain. Modification on the nucleobase can bring higher metabolic stability and increased affinity to the enzyme, whereas sugar modifications cause termination of the chain and often bring selectivity toward viral polymerases. On the other hand, chemical modifications of nucleosides often lead to inefficient phosphorylation by nucleoside kinases and, therefore, many of the nucleoside drugs are use in form of 5′-phosphate prodrugs.^2^,^3^ These effects can be demonstrated on blockbuster drug Sofosbuvir (2′-α-fluoro-2′-β-methyluridine phosphoramidate prodrug),^4^ which is used for treatment of Hepatitis C (HCV), and on GS-5734 (phosphoramidate of 1′-cyano-ribo-C-nucleoside bearing 4-aminopyrrolo[2,1-*f*]triazine base),^5^ which is in clinical trials for treatment of Ebola virus. Despite recent progress in treatment of HCV and other viruses, there are many other, so far neglected emerging viruses,^6^ for which there is no treatment available, and hence are the challenge for the current medicinal chemistry.

**Fig. 1:**
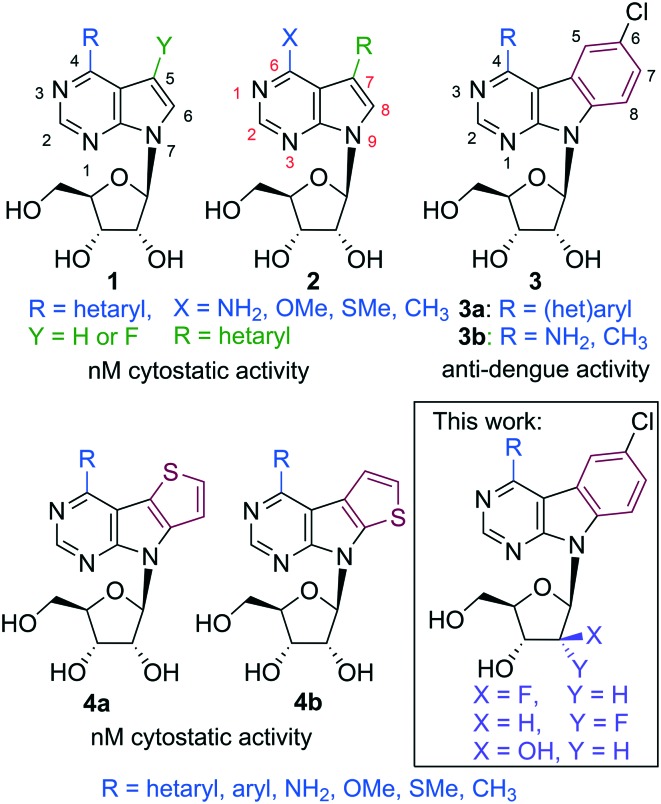
Previously reported 7-deazapurine nucleosides and fused-7-deazapurine nucleosides with cytostatic and antiviral activities. Custom purine numbering (red) is shown in structure **2**, systematic numbering (black) of pyrrolo[2,3-*d*]pyrimidines and pyrimido[4,5-*b*]indoles is shown in structures **1** and **3**, respectively.

Our long-term research of biological activities of 7-deazapurine nucleosides resulted in discovery of two main groups of cytostatics (6-hetaryl-7-deazapurines **1**^7^ and 7-hetaryl 7-deazapurines **2**^8^) with nanomolar activities against broad panel of cancer cell lines. These compounds also showed potent anti-HCV effects, which were unfortunately accompanied by cytotoxicity. These results showed the space for modification in the “major groove” part of the molecule and inspired us to design of fused-7-deazapurine nucleosides with the aim of possible selectivity modulating of antiviral and cytostatic activities. First generation of such fused nucleosides, pyrimidoindole ribonucleosides **3a** bearing various hetaryl groups in position 4,^9^ displayed negligible cytostatic activity, however, several derivatives bearing 2-hetaryl groups exerted interesting micromolar activity against dengue virus.^9^ Benzo-fused 7-deazaadenine analogues **3b** showed^10^ similarly potent anti-dengue effect and anti-HCV activity with 4-methyl derivative being the most active compound with sub-micromolar anti-HCV activity (replicon 1B) and no cytotoxicity. Second generation of fused nucleosides, thienopyrrolopyrimidine ribonucleosides **4**,^11^ were again cytostatic at nanomolar concentrations with potent anti-HCV activity accompanied by cytotoxicity and no effect against dengue virus. In order to complete the SAR of this class of compounds and to gain selectivity to RNA viruses without cytotoxicity, we designed sugar-modified nucleosides derived from 4-substituted 6-chloropyrimido[4,5-*b*]indole ribonucleosides (Fig. 1). We focused on 2′-deoxy-2′-fluororibo-, 2′-deoxy-2′-fluoroarabino- and arabinonucleosides, because related sugar modified derivatives of 7-hetaryl-7-deazapurine nucleosides were previously shown to be significantly less cytotoxic than corresponding ribonucleosides.^12^ Moreover, arabino- or 2′-fluoroarabino sugars occur in clinically used cytostatics Clofarabine^13^ and Fludarabine^14^ and also some 2′-fluororibonucleotides have displayed biological effects.^15^

## Chemistry

Our synthetic plan toward sugar modified pyrimidoindole nucleosides was based on the preparation of the key-intermediate 4,6-dichloropyrimidoindole nucleosides either by glycosylation of known heterocyclic base or by manipulation of functional group in 2′-position of the sugar moiety. The substituents could be introduced to the position 4 on pyrimidine ring either by nucleophilic substitution or by cross-coupling reaction in the final steps.

**Scheme 1:**
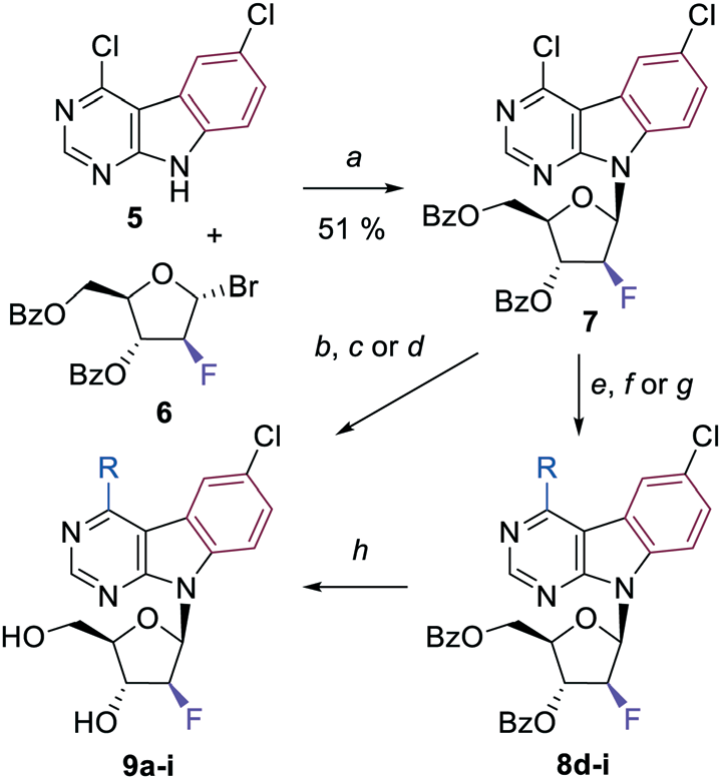
Reagents and conditions: a) KOH, TDA-1, MeCN, r.t., 30 min, then Br-arabinose **6** in MeCN, r.t., 20 h; b) aq. NH_3_, dioxane, 100 °C, 2 days; c) 1 M NaOMe in MeOH, MeOH, r.t., 3 h; d) NaSMe, EtOH, r.t., 4 h; e) (Me)_3_Al (2 M in toluene), Pd(PPh_3_)_4_, THF; 70 °C, 18 h; f) R–B(OH)_2_, K_2_CO_3_, Pd(PPh_3_)_4_, toluene, 100 °C, 17–36 h; g) R–SnBu_3_, PdCl_2_(PPh_3_)_2_, DMF, 100 °C, 17–18 h; h) 1 M NaOMe in MeOH, MeOH, r.t., 2–18 h.

A nucleobase anion glycosylation of the previously reported 4,6-dichloropyrimido[4,5-*b*]indole (**5**)^9^ with the known *α*-bromo-2-fluoroarabinose **6**^16^ furnished the desired key-intermediate fluoroarabinonucleoside **7** in 51% yield (Scheme 1) from which a series of final 4-substituted 2′-deoxy-2′-fluoroarabinonucleosides **9a–i** was then synthesized. The selection of substituents and reaction conditions was based on our previous experience with fused-deazapurine nucleosides.^9^ First, we attempted to deprotect nucleoside **7** to get free 4-chloro 2′-deoxy-2′-fluoroarabinonucleoside, however, the position 4 on pyrimidoindole base was found so reactive, that nucleophilic substitution was easier than debenzoylation and proceeded simultaneously. With the aim to introduce substituents selectively into the position 4 and keep chlorine in position 6 untouched, we applied previously optimized conditions for Suzuki coupling (catalysis by Pd(PPh_3_)_4_ in combination with potassium carbonate as a base in toluene) to synthesize 4-phenyl-, 4-(3-thienyl)- and 4-(3-furyl)-derivatives. Isomeric 2-furyl- and 2-thienyl-derivatives were obtained by Stille coupling with 2-(tributylstannyl)furan or 2-(tributylstannyl)thiophene catalyzed by PdCl_2_(PPh_3_)_2_ in DMF. Methyl group was introduced by Pd-catalyzed methylation with trimethylaluminium. All these reactions were performed starting from the benzoylated nucleoside **7** and intermediates **8d–i** were then deprotected to desired final free nucleosides **9d–i** using the standard Zemplén method – sodium methoxide in methanol. The amino-, methoxy- and methylsulfanyl-derivatives **9a**, **9b**, and **9c** were obtained by nucleophilic substitution with aqueous ammonia in dioxane at 100 °C, sodium methoxide in MeOH or sodium methanethiolate in EtOH, respectively. Benzoyl groups were simultaneously removed under reaction conditions and the final free nucleosides **9** were isolated in good yields (Scheme 1, Table 1).

**Table 1:** Synthesis of 2′-deoxy-2′-fluoroarabinonucleosides **8** and **9**

Entry	R	Conditions	Protected nucleoside	Yield [%]	Final nucleoside	Yield [%]
1	NH_2_	b	—	—	**9a**	78
2	OMe	c	—	—	**9b**	22
3	SMe	d	—	—	**9c**	32
4	Me	e	**8d**	46	**9d**	78
5	2-Furyl	g	**8e**	79	**9e**	69
6	3-Furyl	f	**8f**	77	**9f**	33
7	2-Thienyl	g	**8g**	51	**9g**	78
8	3-Thienyl	f	**8h**	53	**9h**	65
9	Phenyl	f	**8i**	55	**9i**	65

**Scheme 2:**
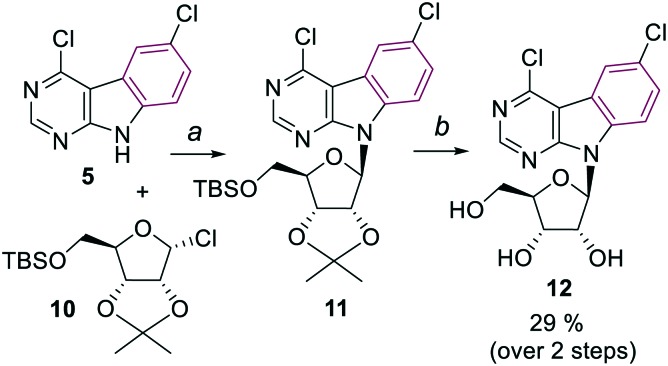
Reagents and conditions: a) KOH, TDA-1, toluene, r.t., 30 min, then **10** in toluene, r.t., 24 h; b) 90% aq. TFA, r.t., 30 min.

The synthesis of arabinonucleosides and 2′-deoxy-2′-fluororibonucleosides was envisaged by modification of 2′-position of the corresponding 4,6-dichloropyrimidoindole ribonucleoside intermediate **12**. It was prepared by stereoselective glycosylation of the pyrimidoindole nucleobase **5** with the protected 1-chlororibose **10**^17^ followed by sugar deprotection. The desired nucleoside **12** was obtained in overall 29% yield as the pure β-anomer (Scheme 2).

**Scheme 3:**
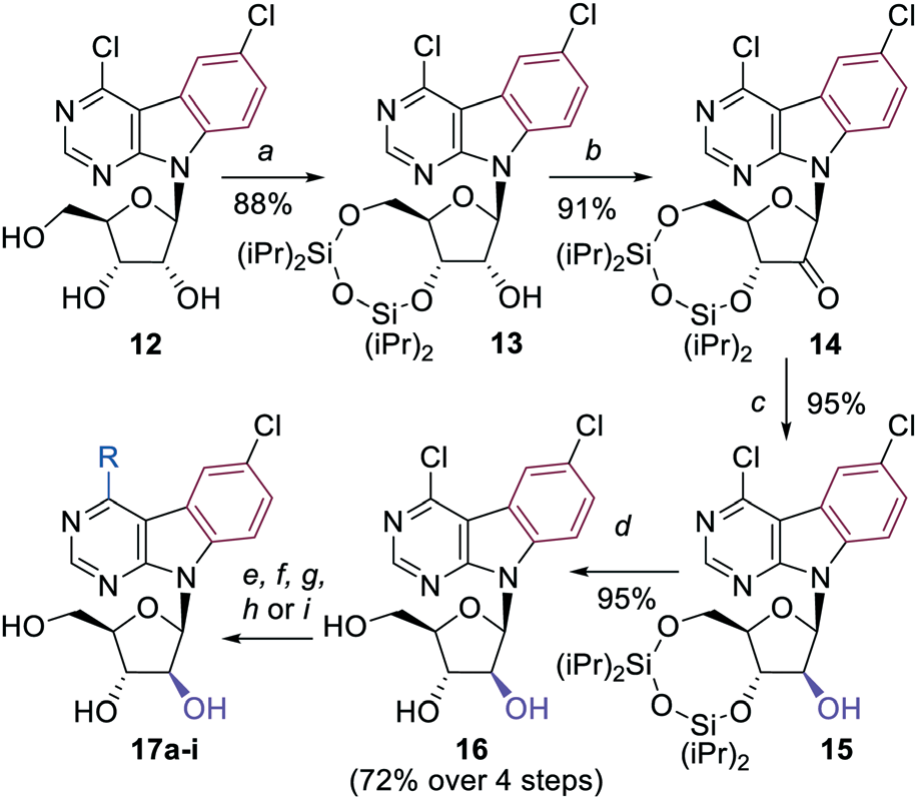
Reagents and conditions: a) TIPDSCl_2_, py, r.t., 4 h; b) Dess–Martin periodinane, DCM, 0 °C to r.t., 18 h; c) NaBH_4_, EtOH, 0 °C to r.t., 1.5 h; d) Et_3_N·3HF, THF, r.t., 18 h; e) aq. NH_3_, dioxane, 100 °C, 20 h; f) 1 M NaOMe in MeOH, MeOH, r.t., 3 h; g) NaSMe, EtOH, r.t., 3 h; h) (Me)_3_Al (2 M in toluene), Pd(PPh_3_)_4_, THF; 70 °C, 18 h; i) R–B(OH)_2_, Na_2_CO_3_, Pd(OAc)_2_, TPPTS, H_2_O/MeCN (2 : 1), 100 °C, 2–4 h.

The key 4,6-dichloropyrimido[4,5-*b*]indole arabinonucleoside intermediate **16** was then prepared by inversion of configuration at the 2′-carbon of the 3′,5′-protected ribonucleoside **13** using a sequence of redox reactions. Nucleoside **13** was first oxidized by Dess–Martin periodinane to oxo-derivative **14** in excellent 91% yield. Then a well known stereoselective reduction of **14** using NaBH_4_ in ethanol12a,b furnished the desired silylated arabinonucleoside **15**, which was deprotected to the free arabinonucleoside **16** in very good 72% yield over 4 steps (Scheme 3).

A series of 4-substituted arabinonucleosides **17a–i** was then prepared in good yields by aromatic nucleophilic substitution, Pd-catalyzed cross-coupling reaction with trimethylaluminium or aqueous-phase Suzuki cross-coupling reaction catalyzed by palladium acetate in combination with TPPTS (Scheme 3, Table 2). The only low yielding reaction was the Suzuki coupling with 2-furylboronic acid probably due to limited stability of the reagent.

**Table 2:** Synthesis of arabinonucleosides **17**

Entry	R	Conditions	Product	Yield [%]
1	NH_2_	e	**17a**	85
2	OMe	f	**17b**	77
3	SMe	g	**17c**	71
4	Me	h	**17d**	68
5	2-Furyl	i	**17e**	33
6	3-Furyl	i	**17f**	62
7	2-Thienyl	i	**17g**	70
8	3-Thienyl	i	**17h**	75
9	Phenyl	i	**17i**	58

**Scheme 4:**
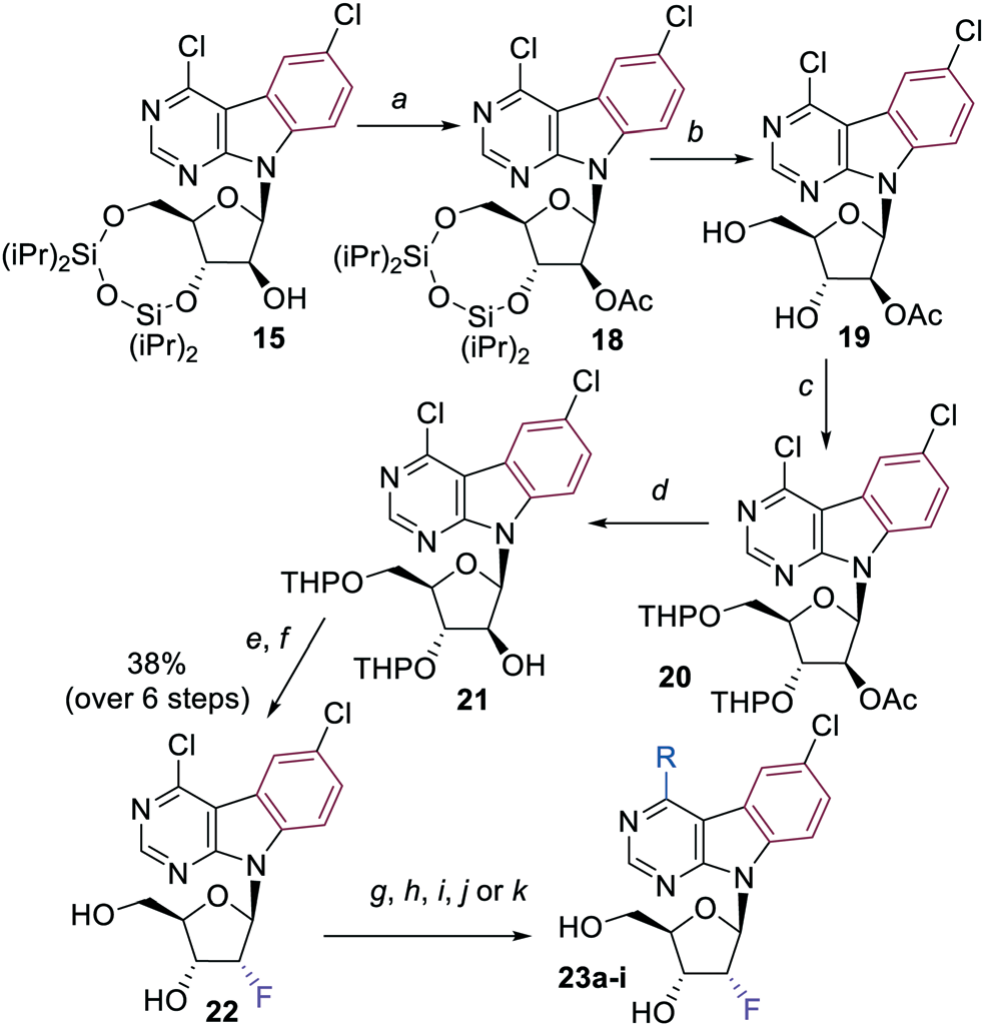
Reagents and conditions: a) Ac_2_O, Et_3_N, DMAP, MeCN, r.t., 1 h; b) Et_3_N·3HF, THF, r.t., 18 h; c) DHP, TsOH, DMF, r.t., 18 h; d) 25–30% NH_3_ in MeOH, 0 °C, 4 h; e) DAST, py, DCM, 0 °C to r.t., 18 h; f) 90% aq. TFA, r.t., 2 h; g) aq. NH_3_, dioxane, 100 °C, 20 h; h) 1 M NaOMe in MeOH, MeOH, r.t., 3 h; i) NaSMe, EtOH, r.t., 2 h; j) (Me)_3_Al (2 M in toluene), Pd(PPh_3_)_4_, THF; 70 °C, 24 h; k) R–B(OH)_2_, Na_2_CO_3_, Pd(OAc)_2_, TPPTS, H_2_O/MeCN (2 : 1), 100 °C, 2–4 h.

4,6-Dichloropyrimido[4,5-*b*]indole 2′-deoxy-2′-fluororibonucleoside **22** was selected as the key intermediate for the synthesis of a series of 2′-deoxy-2′-fluororibo derivatives. It was obtained in good 35% overall yield by a 6-step synthesis concluded by stereoselective S_N_2 fluorination of the bis-THP-protected arabinoside **21** followed by acidic deprotection (Scheme 4). A series of 4-substituted 2′-deoxy-2′-fluororibonucleosides **23a–i** was prepared analogously to arabinonucleosides **17** by nucleophilic substitutions or by Pd-catalyzed cross-coupling reactions (Scheme 4, Table 3). Again, the Suzuki reaction with 2-furylboronic acid gave low yield of desired nucleoside **23e**.

**Table 3:** Synthesis of fluororibonucleosides **23**

Entry	R	Conditions	Product	Yield [%]
1	NH_2_	g	**23a**	82
2	OMe	h	**23b**	80
3	SMe	i	**23c**	87
4	Me	j	**23d**	65
5	2-Furyl	k	**23e**	12
6	3-Furyl	k	**23f**	50
7	2-Thienyl	k	**23g**	70
8	3-Thienyl	k	**23h**	40
9	Phenyl	k	**23i**	41

## Biological activity profiling

### Antiviral activity

All the title nucleosides were subjected to screening of their antiviral activities against hepatitis C virus (HCV, genus: *Hepacivirus*, family: *Flaviridae*), respiratory syncytial virus (RSV, genus: *Respirovirus*, family: *Paramyxoviridae*), dengue virus type 2 (strain 16681, genus: *Flavivirus*, family: *Flaviviridae*), influenza (H1N1 A/Mexico/4108/2009, genus: *Influenzavirus A*, family: *Orthomyxoviridae*), human coxsackie B3 virus (strain Nancy, genus: *Enterovirus*, family: *Picornaviridae*) and human herpesvirus 1 (strain HF, genus: *Simplexvirus*, subfamily: *Alphaherpesvirinae*, family: *Herpesviridae*). The anti-coxsackie, anti-herpes, anti-influenza activity was measured by determining the extent to which the test compounds inhibited virus-induced cytopathic effect in HeLa cells, Vero cells, and MDCK cells, respectively, as previously described.^18^ None of the nucleosides showed any activity against influenza, coxsackie and human herpesvirus.

The anti-RSV activity was tested based on methods published previously.^19^ All the title arabinonucleosides and fluororibonucleosides were completely inactive against RSV. Few fluoroarabino derivatives (**9c**, **9e**, **9f**, **9i**) showed moderate micromolar activity (13.2, 34.6, 25.2 and 11.3 μM, respectively) against RSV. 2-Thienyl derivative **9g** was the most active compound with EC_50_ = 5.2 μM.

The anti-dengue activity was measured by determining the extent to which the test compounds inhibited replication in Vero cells as previously described.^10^ Fluoroarabinonucleosides **9a**, **9e**, **9g**, **9h** and arabino derivative **16** inhibited dengue virus with EC_50_ = 10–33 μM, however, their selectivity index was rather low (Table 4).

**Table 4:** Anti-HCV and anti-dengue activities of nucleosides

Compd	HCV (1B)			HCV (2A)			Dengue type 2		
	EC_50_ (μm)	CC_50_ (μm)	SI	EC_50_ (μm)	CC_50_ (μm)	SI	EC_50_ (μm)	CC_50_ (μm)	SI
**9a**	6.7	>44.4	>6.6	>44.4	33.1	0.75	10.8	12.7	1.2
**9b**	3.1	>44.4	>14.3	10.8	>44.4	>4.1	>50	>50	—
**9c**	1.6	22.9	14.3	6.9	20.2	2.9	>50	>50	—
**9d**	6.3	>44.4	>7.0	23.2	>44.4	>1.9	>50	>50	—
**9e**	4.6	>44.4	>9.7	14.7	34.6	2.4	10.5	39.0	3.7
**9f**	23.0	>44.4	>1.9	>44.4	>44.4	—	>50	>50	—
**9g**	2.5	>44.4	>17.8	13.9	34.3	2.5	27.9	39.1	1.4
**9h**	4.1	26.5	6.5	16.2	30.3	1.9	33.3	>50	1.5
**9i**	5.2	>44.4	>8.5	15.7	34.4	2.2	>50	>50	—
**16**	13.7	32.5	2.4	17.4	21.4	1.2	17.4	40.9	2.4
**17a**	22.3	>44.4	>2.0	>44.4	>44.4	—	>50	>50	—
**17c**	17.4	>44.4	>2.6	38.5	>44.4	>1.2	>50	>50	—
**17d**	18.5	40.2	2.2	>44.4	>44.4	—	>50	>50	—
**17e**	3.0	>44.4	>14.8	24.0	>44.4	>1.9	>50	>50	—
**22**	4.7	9.1	1.9	9.5	10.3	1.1	>50	>50	—
**23a**	4.7	>44.4	>9.4	>44.4	>44.4	—	>50	>50	—
**23b**	8.7	>44.4	>5.1	>44.4	>44.4	—	>50	>50	—
**23c**	6.6	29.0	4.5	17.0	>44.4	>2.6	>50	>50	—
**23d**	5.2	>44.4	>8.5	16.1	>44.4	>2.8	>50	>50	—
**23e**	2.3	>44.4	>19.3	15.7	>44.4	>2.8	>50	>50	—
**23f**	5.2	>44.4	>8.5	21.8	>44.4	>2.0	>50	>50	—
**23g**	18.4	>44.4	>2.4	19.7	>44.4	>2.3	>50	>50	—
**23h**	25.1	>44.4	>1.8	>44.4	>44.4	—	>50	>50	—
**23i**	8.2	>44.4	>5.4	20.3	>44.4	>2.2	>50	>50	—
Mericitabine	1.2	>44.4	>37	0.99	>44.4	>44	nt	nt	—

Screening of anti-HCV activities was performed as previously described^20^ and activities compared to standard Mericitabine.^21^ The series of arabinonuclesides **17**, methoxy and hetaryl derivatives was inactive, whilst amino, methylsulfanyl and methyl derivatives showed moderate anti-HCV effect (EC_50_ = 17–38 μM). On the other hand, fluoroarabino- and fluororibonucleosides **9a–i** and **23a–i** were all active against both 1B and 2A genotypes of HCV in replicon assay at (mostly) single digit micromolar concentrations (Table 4) and, more importantly, they were not cytotoxic (in contrast to the previously reported corresponding ribonucleosides^9^,^10^).

**Fig. 2:**
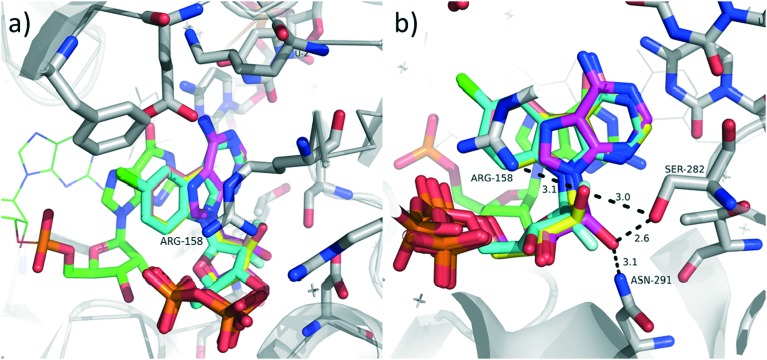
Modelled overlay of diphosphates derived from nucleosides **9a**, **17a**, **23a** and ADP in the co-crystal structure of the viral RNA-dependent RNA polymerase HCV NS5B genotype 2A in complex with RNA template, primer, Mn^2+^, and ADP (PDB code ; 4WTJ, 2.2 Å resolution). a) Detail of the tricyclic base overlayed with ADP; b) detail of ribose binding site, hydrogen bonds of 2′-OH showed as dashed lines and given in Å. Color code: C_enzyme_ grey, C_ADP_ magenta, C_**9a**-DP_ salmon, C_**17a**-DP_ yellow, C_**_23a_**-DP_ cyan, C_RNA_ green, O red, N blue, F pale cyan.

We assume that, similarly to most antiviral nucleosides,^1^,^2^ the mechanism of antiviral activity is intracellular phosphorylation of the nucleosides to NTPs and inhibition of the viral RNA polymerase. To elucidate whether the NTPs of our modified nucleosides would even fit into the active site of the polymerase, we performed a very simple docking and modelling of selected nucleotides into the known crystal structure (PDB code ; 4WTJ)^22^ of viral RNA-dependent RNA polymerase HCV NS5B genotype 2A in complex with RNA template 5′-AUCC, RNA primer 5′-PGG, Mn^2+^ and ADP, which binds to polymerase in catalytically relevant conformation but stalls the primer extension. The modelling was performed using program Moloc and the all-atom MAB force field.^23^ We selected disphosphates of three amino-substituted nucleosides **9a**, **17a** and **23a** and we used the implemented MAB force field to energy minimize them in the active site to mimic ADP in the original crystal structure. For optimization, the protein and RNA coordinates were kept fixed. The modelling clearly showed that there is enough space to accommodate the fused chlorobenzene ring in the nucleobase binding site and it could have even increased cation–π stacking with Arg158 (Fig. 2a). Also the sugar moieties in all three derivatives could adopt similar conformation as in ADP while the orientation of 2′-substituent does not seem to have a significant influence on the binding as it can form hydrogen bonds with the enzyme in ribo-configuration as well as in arabino-configuration (Fig. 2b). The differences in antiviral activities are probably mostly caused by the different efficiency of the intracellular phosphorylation of the nucleosides.

### Cytotostatic activity

The *in vitro* cytostatic activities of the title nucleosides were also evaluated against eight cancer cell lines derived from human solid tumors including lung (A549) and colon (HCT116 and HCT116p53–/–) carcinomas and leukemia cell lines (CCRF-CEM, CEM-DNR, K562 and K562-TAX). Toxicity was evaluated using nonmalignant BJ and MRC-5 fibroblasts. Concentrations inhibiting the cell growth by 50% (IC_50_) were determined as described previously,^11^ using a quantitative metabolic staining with 3-(4,5-dimethylthiazol-2yl)-5-(3-carboxymethoxyphenyl)-2-(4-sulfophenyl)-2*H*-tetrazolium (MTS)^24^ following a 3 day treatment. Results are summarized in Table 5 (only compounds with IC_50_ <50 are shown) and compared to Gemcitabine.^25^

**Table 5:** Cytostatic activities of nucleosides

Compd.	MTS, IC_50_ (μM)									
	BJ	MRC-5	A549	CCRF-CEM	CEM-DNR	HCT116	HCT116p53–/–	K562	K562-TAX	U2OS
**9a**	37.0	43.9	41.4	15.1	32.6	30.4	30.4	27.5	37.2	25.8
**9b**	47.1	>50	>50	34.2	45.9	>50	>50	>50	47.2	>50
**9c**	21.3	22.6	46.8	11.6	18.7	>50	>50	17.6	27.4	29.0
**9d**	20.9	>50	>50	5.1	7.5	>50	44.9	44.2	12.4	29.2
**9e**	26.8	28.7	44.6	13.8	25.0	>50	>50	21.7	26.3	39.9
**9f**	46.2	49.6	>50	7.2	>50	49.9	49.9	>50	39.9	37.4
**9g**	32.0	27.0	>50	18.4	25.8	>50	>50	28.1	28.1	>50
**9h**	41.8	41.8	>50	21.1	27.6	>50	>50	33.0	28.9	>50
**9i**	27.0	30.9	>50	19.7	25.4	49.5	49.5	24.8	26.3	33.4
**16**	>50	49.0	32.6	5.1	25.1	34.0	28.5	44.6	17.7	27.4
**17a**	>50	>50	>50	28.7	>50	>50	49.1	>50	>50	>50
**17c**	>50	>50	>50	11.7	24.0	36.1	34.0	26.5	25.7	26.4
**17d**	>50	>50	>50	16.1	37.2	>50	>50	>50	>50	>50
**17e**	>50	>50	>50	27.1	36.6	>50	>50	>50	42.7	>50
**17i**	>50	>50	>50	24.7	>50	>50	>50	>50	>50	>50
**22**	>50	>50	>50	3.2	36.3	44.6	46.6	>50	>50	32.3
**23b**	>50	>50	>50	34.2	37.9	>50	>50	49.4	34.0	45.2
**23c**	>50	>50	>50	22.1	24.1	>50	>50	25.6	26.1	27.2
**23d**	>50	>50	>50	10.9	10.0	44.7	48.9	>50	9.5	>50
**23e**	39.6	>50	>50	16.7	27.5	48.5	48.5	30.7	27.2	37.8
**23f**	>50	>50	>50	45.6	39.7	>50	>50	>50	>50	>50
**23g**	>50	>50	>50	16.2	31.3	>50	41.6	34.7	28.1	35.2
**23h**	>50	>50	>50	37.4	40.8	>50	>50	>50	>50	>50
Gemcitabine	>50	>50	0.05	0.02	0.10	0.03	0.41	0.10	0.05	0.18

Fluoroarabinonucleosides **9** showed only moderate (>10 μM) cytostatic activity and very poor selectivity against fibroblasts. On the other hand, fluororibonucleosides **23** showed similar activity against CEM cell lines and are not toxic to fibroblasts. Arabinonucleosides **17** bearing methoxy and hetaryl groups in position 4 are inactive against most of the cell lines, they displayed only moderate effect against CEM lines. The most cytotoxic compounds were chloro derivatives **16** and **23** with single digit micromolar activity against CCRF-CEM. In general, most of the arabinonucleosides **17**, fluoroarabinonucleosides **9** and fluororibonucleosides **23** are much less cytotoxic than corresponding ribonucleosides.

## Conclusions

We synthesized 3 sets of sugar modified pyrimidoindole nucleosides – arabino-, fluororibo- and fluoroarabino nucleosides bearing various substituents (amino, methoxy, methylsulfanyl, methyl, 2- and 3-furyl, 2- and 3-thienyl and phenyl) of the heterocyclic base 4-position. The synthesis started with the preparation of key-intermediate 4,6-dichloropyrimidoindole nucleosides, followed by the introduction of the substituent in the last step either by aromatic nucleophilic substitution or palladium catalyzed cross-coupling reactions. In cytostatic activity screening, the sugar-modified derivatives displayed only low activity (compared to ribonucleosides). Some fluoroarabino nucleosides displayed double-digit micromolar anti-dengue and anti-RSV activity. The most interesting result came from anti-HCV screening, where all fluoroarabino- and fluororibonucleosides showed single-digit micromolar activity and no cytotoxicity at maximum tested concentration (44 μM). These compounds are not only more potent than most of the corresponding ribonucleosides,^9^,^10^ but they are also much more selective.

## Abbreviations

TPPTS: Triphenylphosphine-3,3′,3′′-trisulfonic acid trisodium salt
TDA-1: Tris[2-(2-methoxyethoxy)ethyl]amine
DAST: (Diethylamino)sulfur trifluoride

## Conflicts of interest

The authors declare no competing interests.

## Supplementary Material

Supplementary information
