# Supplementary material for: Sugar modified pyrimido[4,5-b]indole nucleosides: synthesis and antiviral activity
Source: Medchemcomm. 2017 Aug 25;8(9):1856–62. doi: 10.1039/c7md00319f (PMC6084004; doi:10.1039/c7md00319f)
Supplement: Supplementary file 1 [file MD-008-C7MD00319F-s001.pdf]

Electronic Supplementary Material (ESI) for MedChemComm.
This journal is © The Royal Society of Chemistry 2017

Supporting information

for

Sugar Modified Pyrimido[4,5-*b*]indole Nucleosides: Synthesis and Antiviral Activity

Juraj Konč,[a] Michal Tichý,[a] Radek Pohl,[a] Jan Hodek,[a] Petr Džubák,[b] Marián Hajdúch,[b] and Michal Hocek*[a],[c]

[a] Institute of Organic Chemistry and Biochemistry, Czech Academy of Sciences, Flemingovo nam. 2, CZ-16610 Prague 6, Czech Republic. E-mail: hocek@uochb.cas.cz

[b] Institute of Molecular and Translational Medicine, Palacky University and University Hospital in Olomouc, Faculty of Medicine and Dentistry, Hněvotínská 5, CZ-775 15 Olomouc, Czech Republic

[c] Department of Organic Chemistry, Faculty of Science, Charles University in Prague, Hlavova 8, CZ-12843 Prague 2, Czech Republic.

Contents:

1. Experimental section
2. HPLC purity of final compounds
3. Biological activity assays and modelling details
4. Copies of NMR spectra

Experimental

1.1 General remarks

All the reagents and solvents were purchased from commercial suppliers and used as received. High pressure flash chromatography (HPFC) purifications were performed on ISCO Combiflash Rf 200 system with RediSep Rf Gold Silica Gel Disposable columns for normal-phase or Reverse Phase (C18) RediSep Rf columns for reversed-phase (RP) HPFC or on Biotage SP-1 apparatus with FLASH 25+M, FLASH 40+M for normal-phase or KP-C18-HS columns for reversed-phase (RP) HPFC. Monitoring of reactions was performed using TLC Silica gel 60 F254 plates, visualization with UV lamp (254 nm) or by a solution of 4-anisaldehyde in ethanol and 10% of sulfuric acid. NMR spectra were recorded on Bruker Avance 400 MHz spectrometer (400.1 MHz for ^{1}H and 100.6 MHz for ^{13}C) or on Bruker Avance 500 MHz spectrometer (500 MHz for ^{1}H, 125.7 MHz for ^{13}C and 470.3 MHz for ^{19}F, referenced to hexafluorobenzene [-163 ppm] as external standard), in $CDCl_3$ (TMS was used as internal standard) or DMSO-d_6 (referenced to the residual solvent signal 2.5 ppm for ^{1}H, 39.7 ppm for ^{13}C). Chemical shifts are given in ppm (δ-scale), coupling constants (*J*) in Hz. Complete assignment of all NMR signals was performed using a combination of H,H-COSY, H,H-ROESY, H,C-HSQC and H,C-HMBC experiments. Low resolution mass spectra were measured on LCQ Fleet (Thermo Fisher Scientific) using electrospray ionization (ESI). High resolution mass spectra were measured on LTQ Orbitrap XL (Thermo Fisher Scientific). Melting points were measured on Stuart automatic melting point SMP40 and are uncorrected. IR spectra (wavenumbers in cm^{-1}) were recorded on Bruker ALPHA FT-IR spectrometer using attenuated total reflection (ATR). Optical rotations were measured at 25 °C in DMSO on Autopol IV (Rudolps Research Analytical) polarimeter, $[\alpha]_D^{20}$ values are given in 10^{-1} $deg \cdot cm^2 \cdot g^{-1}$. Purity of all final compounds (unprotected nucleosides) was determined by analytical HPLC and clean NMR spectra. Analytical HPLC: Waters 600 Controller, Waters 2996 Photodiode Array Detector, Column: Gemini 5µ C18 110A (250×4.60 mm, 5 micron), Eluent: H_2O/MeCN, Flow: 1mL/min.

General Procedure A: Aqueous Suzuki Cross-coupling Reaction.

An argon-purged mixture of free nucleoside (1 eq.), the appropriate boronic acid (1.5 eq.), Na_2CO_3 (3 eq.), $Pd(OAc)_2$ (0.05 eq.), and 3,3',3''-phosphanetriyltris-(benzenesulfonic acid) trisodium salt (TPPTS; 0.12 eq.) in H_2O/MeCN (2:1) was stirred at 100 °C for the stated period of time. After cooling down, the mixture was neutralized by the addition of aqueous 1M HCl and diluted with MeOH (20 mL). Solvents were removed under reduced pressure and the residue was purified by RP-HPFC (C18 column, 10→100% MeOH in H_2O). Final products were crystallized from H_2O/MeOH mixtures.

General Procedure B: Zemplén Deprotection of Nucleosides

Protected nucleosides were dissolved in MeOH (10 mL) and 1M solution of MeONa in MeOH (1 mL) was added. The mixture was stirred at r.t. for the stated period of time and solvent was evaporated under reduced pressure.

4,6-Dichloro-9-(3,5-di-*O*-benzoyl-2-deoxy-2-fluoro-*β*-D-arabinofuranosyl)-9*H*-pyrimido-[4,5-*b*]indole (7)

Pyrimidoindole **5**[1] (1.0 g; 4.2 mmol) and finely grounded potassium hydroxide (0.71 g; 12.6 mmol) were suspended in MeCN (70 mL) and TDA-1 (1.34 mL; 4.2 mmol) was added. The mixture was stirred for 30 min at r.t. after which the crude bromose **6**[2] (5.12 g) in MeCN (55 mL) was added dropwise. The mixture was stirred for another 20 h at r.t., filtered through pad of celite and volatiles were removed under reduced pressure. The crude product was purified using HPFC (silica column, 0→30% EtOAc in hexane). Nucleoside **7** (1.24 g; 51%) was obtained as a yellowish solid: m.p. 158–163 °C; ^{1}H NMR (500.0 MHz, DMSO-d_6): 4.75 (ddd, 1H, $J_{4',3'}$ = 5.9, $J_{4',5'}$ = 4.3, 2.9, H-4'); 4.80 (dd, 1H, J_{gem} = 12.3, $J_{5'b,4'}$ = 4.3, H-5'b); 4.94 (dd, 1H, J_{gem} = 12.3, $J_{5'a,4'}$ = 2.9, H-5'a); 5.83 (ddd, 1H, $J_{H,F}$ = 51.1, $J_{2',1'}$ = 4.1, $J_{2',3'}$ = 1.9, H-2'); 5.99 (ddd, 1H, $J_{H,F}$ = 22.5, $J_{3',4'}$ = 5.9, $J_{3',2'}$ = 1.9, H-3'); 7.13 (dd, 1H, $J_{7,8}$ = 8.9, $J_{7,5}$ = 2.2, H-7); 7.17 (dd, 1H, $J_{H,F}$ = 21.4, $J_{1',2'}$ = 4.1, H-1'); 7.57, 7.60 (2 × m, 2 × 2H, H-*m*-Bz); 7.72, 7.74 (2 × m, 2 × 1H, H-*p*-Bz); 7.97 (ddd, 1H, $J_{8,7}$ = 8.9, $J_{H,F}$ = 3.2, $J_{8,5}$ = 0.5, H-8); 8.07, 8.12 (2 × m, 2 × 2H, H-*o*-Bz); 8.26 (dd, 1H, $J_{5,7}$ = 2.1, $J_{5,8}$ = 0.5, H-5); 8.93 (s, 1H, H-2); ^{13}C NMR (125.7 MHz, DMSO-d_6): 63.16 (CH_2-5'); 76.62 (d, $J_{C,F}$ = 28.6, CH-3'); 77.70 (d, $J_{C,F}$ = 2.5, CH-4'); 83.14 (d, $J_{C,F}$ = 17.5, CH-1'); 95.38 (d, $J_{C,F}$ = 193.2, CH-2'); 111.07 (C-4a); 116.37 (d, $J_{C,F}$ = 6.0, CH-8); 119.65 (C-4b); 121.50 (CH-5); 127.26 (C-6); 128.01 (CH-7); 128.89 (C-*i*-Bz); 128.99, 129.07 (CH-*m*-Bz); 129.45 (CH-*o*-Bz); 129.47 (C-*i*-Bz); 129.87 (CH-*o*-Bz); 133.87, 134.15 (CH-*p*-Bz); 137.14 (C-8a); 152.51 (C-4); 154.80 (CH-2); 155.51 (C-9a); 165.18, 165.54 (CO-Bz); ^{19}F{^{1}H} NMR (470.3 MHz, DMSO-d_6): –189.41; IR (ATR): ν =

1716, 1273, 1114, 1096, 1071, 1029, 709 cm^{-1}; ESI MS m/z (rel.%): 602.0 (100) $[M+Na]^+$; HR MS (ESI) for $C_{29}H_{21}Cl_2FN_3O_5$ $[M+H]^+$: calcd 580.08357; found 580.08368.

6-Chloro-9-(3,5-di-*O*-benzoyl-2-deoxy-2-fluoro-*β*-D-arabinofuranosyl)-4-methyl-9*H*-pyrimido[4,5-*b*]indole (8d)

Protected nucleoside **7** (300 mg; 0.52 mmol) and $Pd(PPh_3)_4$ (30 mg; 0.03 mmol) were dissolved in anhydrous THF (10 mL) and $(Me)_3Al$ (520 μL, 2M in toluene) was added. The mixture was stirred at 70 °C for 18 h. Solvents were removed under reduced pressure and the crude product was purified using HPFC (silica column, 0→40% EtOAc in hexane) to give compound **8d** (135 mg; 46%) as a yellowish solid: m.p. 139–146 °C; ^1H NMR (500.0 MHz, DMSO-d_6): 2.99 (s, 3H, CH_3); 4.73 (ddd, 1H, $J_{4',3'}$ = 6.1, $J_{4',5'}$ = 4.3, 2.9, H-4'); 4.79 (dd, 1H, J_{gem} = 12.3, $J_{5'b,4'}$ = 4.3, H-5'b); 4.94 (dd, 1H, J_{gem} = 12.3, $J_{5'a,4'}$ = 2.9, H-5'a); 5.82 (ddd, 1H, $J_{H,F}$ = 51.4, $J_{2',1'}$ = 4.3, $J_{2',3'}$ = 2.0, H-2'); 6.00 (ddd, 1H, $J_{H,F}$ = 22.7, $J_{3',4'}$ = 6.1, $J_{3',2'}$ = 2.0, H-3'); 7.05 (dd, 1H, $J_{7,8}$ = 8.9, $J_{7,5}$ = 2.2, H-7); 7.17 (dd, 1H, $J_{H,F}$ = 21.4, $J_{1',2'}$ = 4.3, H-1'); 7.53–7.65 (m, 4H, H-*m*-Bz); 7.73, 7.74 (2 × m, 2 × 1H, H-*p*-Bz); 7.93 (dd, 1H, $J_{8,7}$ = 8.9, $J_{H,F}$ = 3.6, H-8); 8.08, 8.12 (2 × m, 2 × 2H, H-*o*-Bz); 8.21 (d, 1H, $J_{5,7}$ = 2.2, H-5); 8.99 (s, 1H, H-2); ^{13}C NMR (125.7 MHz, DMSO-d_6): 22.48 (CH_3); 63.16 (CH_2-5'); 76.71 (d, $J_{C,F}$ = 28.5, CH-3'); 77.48 (d, $J_{C,F}$ = 3.5, CH-4'); 82.80 (d, $J_{C,F}$ = 17.6, CH-1'); 95.50 (d, $J_{C,F}$ = 193.1, CH-2'); 111.56 (C-4a); 115.96 (d, $J_{C,F}$ = 6.2, CH-8); 121.32 (C-4b); 122.35 (CH-5); 126.90 (C-6); 126.94 (CH-7); 128.92 (C-*i*-Bz); 129.02, 129.11 (CH-*m*-Bz); 129.48 (CH-*o*-Bz); 129.49 (C-*i*-Bz); 129.90 (CH-*o*-Bz); 133.91, 134.18 (CH-*p*-Bz); 136.87 (C-8a); 154.06 (CH-2); 154.51 (C-9a); 160.70 (C-4); 165.21, 165.66 (CO-Bz); ^{19}F NMR (470.3 MHz, DMSO-d_6): –189.50 (ddd, $J_{F,H2'}$ = 51.4, $J_{F,H3'}$ = 22.7, $J_{F,H1'}$ = 21.4); IR (ATR): ν = 1721, 1477, 1454, 1264, 1164, 1115, 1101, 1072, 1043, 1031, 711 cm^{-1}; ESI MS *m/z* (rel.%): 560.0 (57) $[M+H]^+$, 582.0 (100) $[M+Na]^+$; HR MS (ESI) for $C_{30}H_{24}ClFN_3O_5$ $[M+H]^+$: calcd 560.13830; found 560.13841.

6-Chloro-9-(3,5-di-*O*-benzoyl-2-deoxy-2-fluoro-*β*-D-arabinofuranosyl)-4-(furan-2-yl)-9*H*-pyrimido[4,5-*b*]indole (8e)

Protected nucleoside **7** (300 mg; 0.52 mmol), 2-(tributylstannyl)furan (270 mg; 0.63 mmol) and $PdCl_2(PPh_3)_2$ (18.2 mg; 0.03 mmol) were dissolved in anhydrous DMF (10 mL) and heated to 100 °C for 18 h. Solvent was co-evaporated with toluene under reduced pressure. Crude product was purified using column chromatography on silica column containing 15% of KF. Column was first washed with 3 L of hexane and the product was then eluted with 20% EtOAc in hexane. Desired product **8e** (249 mg; 79%) was obtained as a yellow foam: ^1H NMR (500.0 MHz, DMSO-d_6): 4.73 (ddd, 1H, $J_{4',3'}$ = 6.0, $J_{4',5'}$ = 4.1, 2.9, H-4'); 4.80 (dd, 1H, J_{gem} = 12.3, $J_{5'b,4'}$ = 4.1, H-5'b);

4.95 (dd, 1H, J_{gem} = 12.3, $J_{5'a,4'}$ = 2.9, H-5'a); 5.82 (ddd, 1H, $J_{H,F}$ = 51.3, $J_{2',1'}$ = 4.2, $J_{2',3'}$ = 1.8, H-2'); 5.99 (ddd, 1H, $J_{H,F}$ = 22.7, $J_{3',4'}$ = 6.0, $J_{3',2'}$ = 1.8, H-3'); 6.89 (dd, 1H, $J_{4,3}$ = 3.5, $J_{4,5}$ = 1.7, H-4-furyl); 6.99 (ddd, 1H, $J_{7,8}$ = 8.9, $J_{7,5}$ = 2.2, $J_{H,F}$ = 1.3, H-7); 7.21 (dd, 1H, $J_{H,F}$ = 22.2, $J_{1',2'}$ = 4.2, H-1'); 7.56–7.62 (m, 5H, H-3-furyl, H-*m*-Bz); 7.71–7.76 (m, 2H, H-*p*-Bz); 7.93 (dd, 1H, $J_{8,7}$ = 8.9, $J_{H,F}$ = 3.7, H-8); 8.09, 8.12 (2 × m, 2 × 2H, H-*o*-Bz); 8.34 (dd, 1H, $J_{5,4}$ = 1.7, $J_{5,3}$ = 0.9, H-5-furyl); 8.74 (d, 1H, $J_{5,7}$ = 2.2, H-5); 8.99 (s, 1H, H-2); ^{13}C NMR (125.7 MHz, DMSO-d_6): 63.17 (CH_2-5'); 76.77 (d, $J_{C,F}$ = 28.7, CH-3'); 77.63 (d, $J_{C,F}$ = 3.2, CH-4'); 82.95 (d, $J_{C,F}$ = 17.6, CH-1'); 95.54 (d, $J_{C,F}$ = 193.2, CH-2'); 106.95 (C-4a); 113.35 (CH-4-furyl); 115.76 (d, $J_{C,F}$ = 4.8, CH-8); 115.78 (CH-3-furyl); 120.68 (C-4b); 123.59 (CH-5); 126.66 (C-6); 127.17 (CH-7); 128.94 (C-*i*-Bz); 129.04, 129.13 (CH-*m*-Bz); 129.51 (CH-*o*-Bz); 129.52 (C-*i*-Bz); 129.92 (CH-*o*-Bz); 133.93, 134.19 (CH-*p*-Bz); 137.33 (C-8a); 147.15 (CH-5-furyl); 148.10 (C-4); 152.22 (C-2-furyl); 154.57 (CH-2); 156.29 (C-9a); 165.24, 165.70 (CO-Bz); ^{19}F NMR (470.3 MHz, DMSO-d_6): –189.28 (ddd, $J_{F,H2'}$ = 51.3, $J_{F,H3'}$ = 22.7, $J_{F,H1'}$ = 22.2); IR (ATR): ν = 2933, 2863, 1723, 1566, 1543, 1462, 1439, 1265, 1252, 1090, 1071, 1029, 710 cm^{-1}; ESI MS *m/z* (rel.%): 612.2 (70) $[M+H]^+$, 634.2 (100) $[M+Na]^+$; HR MS (ESI) for $C_{33}H_{24}ClFN_3O_6$ $[M+H]^+$: calcd 612.13322; found 612.13335.

6-Chloro-9-(3,5-di-*O*-benzoyl-2-deoxy-2-fluoro-*β*-D-arabinofuranosyl)-4-(furan-3-yl)-9*H*-pyrimido[4,5-*b*]indole (8f)

Protected nucleoside **7** (250 mg; 0.43 mmol), furan-3-boronic acid (73 mg; 0.65 mmol), K_2CO_3 (120 mg; 0.86 mmol) and $Pd(PPh_3)_4$ (25 mg; 0,02 mmol) were dissolved in toluene (10 mL) and heated to 100 °C for 18 h. More furan-3-boronic acid (150 mg; 1.34 mmol) and $Pd(PPh_3)_4$ (45 mg; 0.04 mmol) was added and heating continued for another 2 days. Reaction mixture was diluted with water and extracted with chloroform. Organic layer was washed with saturated NH_4Cl and water and dried over $MgSO_4$. Solvent was evaporated under reduced pressure and the crude product was purified using HPFC (silica column, 0→40% EtOAc in hexane) to give compound **8f** (200 mg; 77%) as a yellow foam: ^{1}H NMR (500.0 MHz, DMSO-d_6): 4.74 (ddd, 1H, $J_{4',3'}$ = 6.0, $J_{4',5'}$ = 4.2, 2.9, H-4'); 4.79 (dd, 1H, J_{gem} = 12.3, $J_{5'b,4'}$ = 4.2, H-5'b); 4.95 (dd, 1H, J_{gem} = 12.3, $J_{5'a,4'}$ = 2.9, H-5'a); 5.84 (ddd, 1H, $J_{H,F}$ = 51.3, $J_{2',1'}$ = 4.3, $J_{2',3'}$ = 1.9, H-2'); 6.00 (ddd, 1H, $J_{H,F}$ = 22.6, $J_{3',4'}$ = 6.0, $J_{3',2'}$ = 1.9, H-3'); 7.01 (dd, 1H, $J_{7,8}$ = 8.9, $J_{7,5}$ = 2.2, H-7); 7.13 (dd, 1H, $J_{4,5}$ = 1.9, $J_{4,2}$ = 0.9, H-4-furyl); 7.22 (dd, 1H, $J_{H,F}$ = 21.7, $J_{1',2'}$ = 4.2, H-1'); 7.53–7.65 (m, 4H, H-*m*-Bz); 7.73, 7.74 (2 × m, 2 × 1H, H-*p*-Bz); 7.94 (m, 1H, H-8); 8.01 (dd, 1H, $J_{5,4}$ = 1.9, $J_{5,2}$ = 1.5, H-5-furyl); 8.06 (d, 1H, $J_{5,7}$ = 2.2, H-5); 8.08, 8.12 (2 × m, 2 × 2H, H-*o*-Bz); 8.56 (dd, 1H, $J_{2,5}$ = 1.5, $J_{2,4}$ = 0.9, H-2-furyl); 9.05 (s, 1H, H-2); ^{13}C NMR (125.7 MHz, DMSO-d_6): 63.19 (CH_2-5'); 76.78 (d, $J_{C,F}$ = 28.5, CH-3'); 77.61 (d, $J_{C,F}$ = 3.3, CH-4'); 82.91 (d, $J_{C,F}$ = 17.6, CH-1'); 95.59 (d, $J_{C,F}$ = 193.2,

CH-2'); 110.15 (C-4a); 110.78 (CH-4-furyl); 116.12 (d, $J_{C,F}$ = 6.6, CH-8); 120.80 (C-4b); 121.51 (CH-5); 124.28 (C-3-furyl); 126.46 (C-6); 127.22 (CH-7); 128.97 (C-*i*-Bz); 129.10, 129.18 (CH-*m*-Bz); 129.55 (CH-*o*-Bz, C-*i*-Bz); 129.97 (CH-*o*-Bz); 133.99, 134.26 (CH-*p*-Bz); 137.12 (C-8a); 144.76 (CH-2-furyl); 145.03 (CH-5-furyl); 153.44 (C-4); 154.93 (CH-2); 155.64 (C-9a); 165.30, 165.74 (CO-Bz); ^{19}F NMR (470.3 MHz, DMSO-d_6): –189.44 (ddd, $J_{F,H2'}$ = 51.3, $J_{F,H3'}$ = 22.6, $J_{F,H1'}$ = 21.7); IR (ATR): ν = 1723, 1564, 1546, 1441, 1267, 1096, 1071, 803, 711 cm^{-1}; ESI MS *m/z* (rel.%): 612.0 (44) $[M+H]^+$, 634.0 (100) $[M+Na]^+$; HR MS (ESI) for $C_{33}H_{24}ClFN_3O_6$ $[M+H]^+$: calcd 612.13322; found 612.13348.

6-Chloro-9-(3,5-di-*O*-benzoyl-2-deoxy-2-fluoro-*β*-D-arabinofuranosyl)-4-(thiophen-2-yl)-9*H*-pyrimido[4,5-*b*]indole (8g)

Protected nucleoside **7** (350 mg; 0.60 mmol), 2-(tributylstannyl)thiophene (270 mg; 0.72 mmol) and $PdCl_2(PPh_3)_2$ (21 mg; 0.03 mmol) were dissolved in anhydrous DMF (10 mL) and heated to 100 °C for 17 h. Solvent was co-evaporated with toluene under reduced pressure. Crude product was purified using column chromatography on silica column containing 15% of KF. Column was first washed with 3 L of PE and the product was then eluted with 20% EtOAc in PE. Desired product **8g** (194 mg; 51%) was obtained as a white foam: ^{1}H NMR (500.0 MHz, DMSO-d_6): 4.75 (ddd, 1H, $J_{4',3'}$ = 5.9, $J_{4',5'}$ = 4.2, 2.9, H-4'); 4.80 (dd, 1H, J_{gem} = 12.3, $J_{5'b,4'}$ = 4.2, H-5'b); 4.95 (dd, 1H, J_{gem} = 12.3, $J_{5'a,4'}$ = 2.9, H-5'a); 5.85 (ddd, 1H, $J_{H,F}$ = 51.3, $J_{2',1'}$ = 4.2, $J_{2',3'}$ = 1.8, H-2'); 6.00 (ddd, 1H, $J_{H,F}$ = 22.6, $J_{3',4'}$ = 5.9, $J_{3',2'}$ = 1.8, H-3'); 7.02 (ddd, 1H, $J_{7,8}$ = 8.9, $J_{7,5}$ = 2.2, $J_{H,F}$ = 0.7, H-7); 7.23 (dd, 1H, $J_{H,F}$ = 22.2, $J_{1',2'}$ = 4.2, H-1'); 7.42 (dd, 1H, $J_{4,3}$ = 5.0, $J_{4,5}$ = 3.7, H-4-thienyl); 7.58, 7.61 (2 × m, 2 × 2H, H-*m*-Bz); 7.73, 7.74 (2 × m, 2 × 1H, H-*p*-Bz); 7.96 (dd, 1H, $J_{8,7}$ = 8.9, $J_{H,F}$ = 3.7, H-8); 8.01 (dd, 1H, $J_{3,4}$ = 5.0, $J_{3,5}$ = 1.1, H-3-thienyl); 8.07–8.10 (m, 3H, H-5-thienyl, H-*o*-Bz); 8.13 (m, 2H, H-*o*-Bz); 8.20 (d, 1H, $J_{5,7}$ = 2.2, H-5); 9.03 (s, 1H, H-2); ^{13}C NMR (125.7 MHz, DMSO-d_6): 63.17 (CH_2-5'); 76.74 (d, $J_{C,F}$ = 28.6, CH-3'); 77.62 (d, $J_{C,F}$ = 3.1, CH-4'); 82.96 (d, $J_{C,F}$ = 17.6, CH-1'); 95.55 (d, $J_{C,F}$ = 193.1, CH-2'); 108.80 (C-4a); 116.21 (d, $J_{C,F}$ = 6.4, CH-8); 120.67 (C-4b); 121.19 (CH-5); 126.37 (C-6); 127.35 (CH-7); 128.77 (CH-4-thienyl); 128.94 (C-*i*-Bz); 129.04, 129.13 (CH-*m*-Bz); 129.50 (CH-*o*-Bz, C-*i*-Bz); 129.92 (CH-*o*-Bz); 130.26 (CH-5-thienyl); 131.86 (CH-3-thienyl); 133.93, 134.20 (CH-*p*-Bz); 137.18 (C-8a); 140.83 (C-2-thienyl); 153.67 (C-4); 154.63 (CH-2); 156.03 (C-9a); 165.23, 165.68 (CO-Bz); ^{19}F NMR (470.3 MHz, DMSO-d_6): –189.30 (ddd, $J_{F,H2'}$ = 51.3, $J_{F,H3'}$ = 22.6, $J_{F,H1'}$ = 22.2); IR (ATR): ν = 1716, 1556, 1462, 1438, 1262, 1118, 1064, 999, 805, 709 cm^{-1}; ESI MS m/z (rel. %): 650 (100) $[M+Na]^+$, 628 (50) $[M+H]^+$; HR MS (ESI) for $C_{33}H_{23}ClFN_3O_5NaS$ $[M+Na]^+$: calcd 650.09232; found 650.09235.

6-Chloro-9-(3,5-di-*O*-benzoyl-2-deoxy-2-fluoro-*β*-D-arabino-furanosyl)-4-(thiophen-3-yl)-9*H*-pyrimido[4,5-*b*]indole (8h)

Protected nucleoside **7** (325 mg; 0.56 mmol), thiophene-3-boronic acid (107 mg; 0.84 mmol), K_2CO_3 (155 mg; 1.12 mmol) and $Pd(PPh_3)_4$ (33 mg; 0.03 mmol) were dissolved in toluene (10 mL) and heated to 100 °C for 17 h. Reaction mixture was diluted with water and extracted with chloroform. Organic layer was washed with saturated NH_4Cl and water and dried over $MgSO_4$. Solvent was evaporated under reduced pressure and the crude product was purified using HPFC (silica column, 0→50% EtOAc in PE) to give compound **8h** (200 mg; 53%) as a yellow foam: ^{1}H NMR (500.0 MHz, DMSO-d_6): 4.74 (ddd, 1H, $J_{4',3'}$ = 6.0, $J_{4',5'}$ = 4.2, 2.9, H-4'); 4.80 (dd, 1H, J_{gem} = 12.3, $J_{5'b,4'}$ = 4.2, H-5'b); 4.95 (dd, 1H, J_{gem} = 12.3, $J_{5'a,4'}$ = 2.9, H-5'a); 5.85 (ddd, 1H, $J_{H,F}$ = 51.3, $J_{2',1'}$ = 4.2, $J_{2',3'}$ = 1.9, H-2'); 6.01 (ddd, 1H, $J_{H,F}$ = 22.6, $J_{3',4'}$ = 6.0, $J_{3',2'}$ = 1.9, H-3'); 7.00 (dd, 1H, $J_{7,8}$ = 8.9, $J_{7,5}$ = 2.2, H-7); 7.23 (dd, 1H, $J_{H,F}$ = 21.9, $J_{1',2'}$ = 4.2, H-1'); 7.58, 7.61 (2 × m, 2 × 2H, H-*m*-Bz); 7.67 (dd, 1H, $J_{4,5}$ = 5.0, $J_{4,2}$ = 1.3, H-4-thienyl); 7.73, 7.74 (2 × m, 2 × 1H, H-*p*-Bz); 7.88 (dd, 1H, $J_{5,4}$ = 5.0, $J_{5,2}$ = 2.9, H-5-thienyl); 7.91 (d, 1H, $J_{5,7}$ = 2.2, H-5); 7.94 (dd, 1H, $J_{8,7}$ = 8.9, $J_{H,F}$ = 3.7, H-8); 8.08, 8.13 (2 × m, 2 × 2H, H-*o*-Bz); 8.34 (dd, 1H, $J_{2,5}$ = 2.9, $J_{2,4}$ = 1.3, H-2-thienyl); 9.07 (s, 1H, H-2); ^{13}C NMR (125.7 MHz, DMSO-d_6): 63.18 (CH_2-5'); 76.76 (d, $J_{C,F}$ = 28.6, CH-3'); 77.59 (d, $J_{C,F}$ = 3.3, CH-4'); 82.90 (d, $J_{C,F}$ = 17.7, CH-1'); 95.57 (d, $J_{C,F}$ = 193.1, CH-2'); 110.06 (C-4a); 116.14 (d, $J_{C,F}$ = 6.6, CH-8); 120.87 (C-4b); 121.32 (CH-5); 126.29 (C-6); 127.19 (CH-7); 128.00 (CH-5-thienyl); 128.20 (CH-4-thienyl); 128.92 (CH-2-thienyl); 128.95 (C-*i*-Bz); 129.06, 129.14 (CH-*m*-Bz); 129.51 (CH-*o*-Bz, C-*i*-Bz); 129.94 (CH-*o*-Bz); 133.94, 134.21 (CH-*p*-Bz); 137.15 (C-8a); 139.00 (C-3-thienyl); 154.88 (CH-2); 155.74 (C-9a); 155.82 (C-4); 165.25, 165.70 (CO-Bz); ^{19}F NMR (470.3 MHz, DMSO-d_6): –189.36 (ddd, $J_{F,H2'}$ = 51.3, $J_{F,H3'}$ = 22.6, $J_{F,H1'}$ = 21.9); IR (ATR): ν = 2946, 1719, 1567, 1438, 1254, 1161, 1068, 1027, 839, 794, 707 cm^{-1}; ESI MS m/z (rel. %): 628 (100) $[M+H]^+$, 650 (54) $[M+Na]^+$; HR MS (ESI) for $C_{33}H_{24}ClFN_3O_5S$ $[M+H]^+$: calcd 628.11037; found 628.11051.

6-Chloro-9-(3,5-di-*O*-benzoyl-2-deoxy-2-fluoro-*β*-D-arabinofuranosyl)-4-phenyl-9*H*-pyrimido[4,5-*b*]indole (8i)

Protected nucleoside **7** (335 mg; 0.58 mmol), phenylboronic acid (106 mg; 0.87 mmol), K_2CO_3 (160 mg; 1.16 mmol) and $Pd(PPh_3)_4$ (34 mg; 0.03 mmol) were dissolved in toluene (10 mL) and heated to 100 °C for 17 h. The mixture was diluted with water and extracted with chloroform. Organic layer was washed with saturated NH_4Cl and water and dried over $MgSO_4$. Solvent was evaporated under reduced pressure and crude product was purified using HPFC (silica column, 0→50% EtOAc in PE) to give compound **8i** (200 mg; 55%) as a yellow foam: ^{1}H NMR (500.0

MHz, DMSO-d_6): 4.75 (ddd, 1H, $J_{4',3'}$ = 6.0, $J_{4',5'}$ = 4.2, 2.9, H-4'); 4.80 (dd, 1H, J_{gem} = 12.3, $J_{5'b,4'}$ = 4.2, H-5'b); 4.95 (dd, 1H, J_{gem} = 12.3, $J_{5'a,4'}$ = 2.9, H-5'a); 5.86 (ddd, 1H, $J_{H,F}$ = 51.3, $J_{2',1'}$ = 4.2, $J_{2',3'}$ = 1.9, H-2'); 6.02 (ddd, 1H, $J_{H,F}$ = 22.6, $J_{3',4'}$ = 6.0, $J_{3',2'}$ = 1.9, H-3'); 7.00 (dd, 1H, $J_{7,8}$ = 8.9, $J_{7,5}$ = 2.2, H-7); 7.25 (dd, 1H, $J_{H,F}$ = 21.8, $J_{1',2'}$ = 4.2, H-1'); 7.58, 7.61 (2 × m, 2 × 2H, H-*m*-Bz); 7.65 (d, 1H, $J_{5,7}$ = 2.2, H-5); 7.66–7.77 (m, 5H, H-*p*-Bz, H-*m,p*-Ph); 7.90 (m, 2H, H-*o*-Ph); 7.95 (dd, 1H, $J_{8,7}$ = 8.9, $J_{H,F}$ = 3.7, H-8); 8.08, 8.14 (2 × m, 2 × 2H, H-*o*-Bz); 9.13 (s, 1H, H-2); ^{13}C NMR (125.7 MHz, DMSO-d_6): 63.19 (CH$_2$-5'); 76.75 (d, $J_{C,F}$ = 28.6, CH-3'); 77.59 (d, $J_{C,F}$ = 3.3, CH-4'); 82.90 (d, $J_{C,F}$ = 17.6, CH-1'); 95.55 (d, $J_{C,F}$ = 193.1, CH-2'); 110.27 (C-4a); 116.23 (d, $J_{C,F}$ = 6.5, CH-8); 120.80 (C-4b); 121.09 (CH-5); 126.19 (C-6); 127.24 (CH-7); 128.94 (C-*i*-Bz); 128.97 (CH-*o*-Ph); 129.04, 129.09, 129.11 (CH-m-Ph, CH-*m*-Bz); 129.49 (CH-*o*-Bz); 129.50 (C-*i*-Bz); 129.92 (CH-*o*-Bz); 130.80 (CH-*p*-Ph); 133.92, 134.20 (CH-*p*-Bz); 137.20 (C-8a); 137.51 (C-*i*-Ph); 154.98 (CH-2); 155.65 (C-9a); 160.50 (C-4); 165.23, 165.67 (CO-Bz); ^{19}F NMR (470.3 MHz, DMSO-d_6): –189.31 (ddd, $J_{F,H2'}$ = 51.3, $J_{F,H3'}$ = 22.6, $J_{F,H1'}$ = 21.8); IR (ATR): ν = 1711, 1557, 1447, 1435, 1263, 1167, 1111, 1093, 1069, 1027, 821, 705 cm^{-1}; ESI MS m/z (rel. %): 622 (100) [M+H], 644 (24) [M+Na]; HR MS (ESI) for $C_{35}H_{26}ClFN_3O_5$ [M+H]$^+$: calcd 622.15395; found 622.15408.

4-Amino-6-chloro-9-(2-deoxy-2-fluoro-*β*-D-arabinofuranosyl)-9*H*-pyrimido-[4,5-*b*]indole (9a)

Nucleoside **7** (300 mg; 0.52 mmol) was dissolved in dioxane (2 mL) and 30% aqueous ammonia (6 mL) was added. Mixture was stirred in screw-cap pressure glass tube at 100 °C for 2 days. Volatiles were removed under reduced pressure and the crude product was purified using HPFC (silica column, 0→20% MeOH in DCM) and recrystallized from a H_2O/MeOH mixture to give compound **9a** (145 mg; 78%) as white crystals: m.p. 267–272 °C; $[\alpha]_D^{20}$ +40.1 (c 0.30, DMSO); ^{1}H NMR (499.8 MHz, DMSO-d_6): 3.73–3.85 (m, 3H, H-4',5'); 4.46 (dddd, 1H, $J_{H,F}$ = 23.5, $J_{3',4'}$ = 5.5, $J_{3',OH}$ = 5.3, $J_{3',2'}$ = 2.4, H-3'); 5.14 (ddd, 1H, $J_{H,F}$ = 53.3, $J_{2',1'}$ = 4.4, $J_{2',3'}$ = 2.4, H-2'); 5.17 (t, 1H, $J_{OH,5'}$ = 5.5, OH-5'); 5.92 (d, 1H, $J_{OH,3'}$ = 5.3, OH-3'); 6.84 (dd, 1H, $J_{H,F}$ = 21.1, $J_{1',2'}$ = 4.4, H-1'); 7.35 (dd, 1H, $J_{7,8}$ = 8.8, $J_{7,5}$ = 2.1, H-7); 7.47 (bs, 2H, NH$_2$); 7.87 (dd, 1H, $J_{8,7}$ = 8.8, $J_{H,F}$ = 3.1, H-8); 8.33 (s, 1H, H-2); 8.46 (d, 1H, $J_{5,7}$ = 2.1, H-5); ^{13}C NMR (125.7 MHz, DMSO-d_6): 60.33 (CH$_2$-5'); 74.29 (d, $J_{C,F}$ = 24.1, CH-3'); 82.52 (d, $J_{C,F}$ = 17.6, CH-1'); 82.95 (d, $J_{C,F}$ = 4.6, CH-4'); 94.86 (C-4a); 98.14 (d, $J_{C,F}$ = 192.1, CH-2'); 115.43 (d, $J_{C,F}$ = 5.3, CH-8); 120.39 (CH-5); 121.77 (C-4b); 124.46 (CH-7); 126.01 (C-6); 135.33 (C-8a); 155.38 (C-9a); 155.52 (CH-2); 157.87 (C-4); ^{19}F{^{1}H} NMR (470.3 MHz, DMSO-d_6): –188.81. IR (ATR): ν = 3484, 3327, 3153, 2939, 1655,

1590, 1576, 1462, 1306, 1043, 797, 426 cm^{-1}; ESI MS *m/z* (rel.%): 353.0 (85) $[M+H]^+$, 375.0 (100) $[M+Na]^+$; HR MS (ESI) for $C_{15}H_{14}ClFN_4O_3$ $[M+H]^+$: calcd 353.08128; found 353.08112.

6-Chloro-9-(2-deoxy-2-fluoro-*β*-D-arabinofuranosyl)-4-methoxy-9*H*-pyrimido-[4,5-*b*]indole (9b)

Nucleoside **7** (210 mg, 0.36 mmol) was dissolved in dry MeOH (10 mL) and solution of sodium methoxide (1M in MeOH, 1 mL) was added. The mixture was stirred for 3 hours at r.t. Solvent was evaporated and the crude was purified by RP-HPFC (C18 column, 10→100% MeOH in H_2O) and recrystallized from a H_2O/MeOH mixture to give compound **9b** (29 mg, 22%) as a white solid: m.p. 248–250 °C; $[\alpha]_D^{20}$ +30.2 (c 0.16, DMSO); ^{1}H NMR (500.0 MHz, DMSO-d_6): 3.74–3.85 (m, 2H, H-5'); 3.87 (m, 1H, H-4'); 4.21 (s, 3H, CH_3O); 4.49 (dddd, 1H, $J_{H,F}$ = 23.5, $J_{3',4'}$ = 5.9, $J_{3',OH}$ = 5.3, $J_{3',2'}$ = 2.6, H-3'); 5.19 (t, 1H, $J_{OH,5'}$ = 5.5, OH-5′); 5.20 (ddd, 1H, $J_{H,F}$ = 53.2, $J_{2',1'}$ = 4.5, $J_{2',3'}$ = 2.6, H-2'); 5.98 (d, 1H, $J_{OH,3'}$ = 5.3, OH-3′); 6.91 (dd, 1H, $J_{H,F}$ = 20.3, $J_{1',2'}$ = 4.5, H-1'); 7.49 (dd, 1H, $J_{7,8}$ = 8.9, $J_{7,5}$ = 2.2, H-7); 7.97 (d, 1H, $J_{5,7}$ = 2.2, H-5); 7.99 (dd, 1H, $J_{8,7}$ = 8.9, $J_{H,F}$ = 3.0, H-8); 8.72 (s, 1H, H-2); ^{13}C NMR (125.7 MHz, DMSO-d_6): 54.53 (CH_3O); 60.29 (CH_2-5'); 74.20 (d, $J_{C,F}$ = 24.1, CH-3'); 82.78 (d, $J_{C,F}$ = 17.6, CH-1'); 83.11 (d, $J_{C,F}$ = 4.8, CH-4'); 98.08 (d, $J_{C,F}$ = 192.1, CH-2'); 98.40 (C-4a); 116.40 (d, $J_{C,F}$ = 5.3, CH-8); 120.36 (C-4b); 120.98 (CH-5); 126.24 (CH-7); 126.47 (C-6); 136.17 (C-8a); 155.23 (CH-2); 156.14 (C-9a); 163.97 (C-4); ^{19}F NMR (470.3 MHz, DMSO-d_6): –188.99 (ddd, $J_{F,H2'}$ = 53.2, $J_{F,H3'}$ = 23.5, $J_{F,H1'}$ = 20.3); IR (ATR): ν = 3232, 1596, 1457, 1321, 1293, 1168, 1135, 1051, 981, 874, 799, 648, 554 cm^{-1}; ESI MS m/z (rel. %): 390 (100) $[M+Na]^+$, 368 (50) $[M+H]^+$; HR MS (ESI) for $C_{16}H_{15}O_4N_3ClFNa$ $[M+Na]^+$: calcd 390.06273; found 390.06284.

6-Chloro-9-(2-deoxy-2-fluoro-*β*-D-arabinofuranosyl)-4-methylsulfanyl-9*H*-pyrimido-[4,5-*b*]indole (9c)

Protected nucleoside **7** (260 mg, 0.45 mmol) and sodium methanethiolate (52 mg, 0.74 mmol) were dissolved in anhydrous EtOH (10 mL) and stirred for 4 hours at r.t. The solvent was removed under reduced pressure and the crude product was purified by RP-HPFC (C18 column, 10→100% MeOH in H_2O). Crystallization from a H_2O/MeOH mixture gave compound **9c** (46 mg, 32%) as a white solid: m.p. 230–231 °C; $[\alpha]_D^{20}$ +35.4 (c 0.23, DMSO); ^{1}H NMR (500.0 MHz, DMSO-d_6): 2.79 (s, 3H, CH_3S); 3.75–3.85 (m, 2H, H-5'); 3.88 (m, 1H, H-4'); 4.49 (dddd, 1H, $J_{H,F}$ = 23.6, $J_{3',4'}$ = 5.6, $J_{3',OH}$ = 5.3, $J_{3',2'}$ = 2.6, H-3'); 5.21 (ddd, 1H, $J_{H,F}$ = 53.2, $J_{2',1'}$ = 4.5, $J_{2',3'}$ = 2.6, H-2'); 5.22 (t, 1H, $J_{OH,5'}$ = 5.5, OH-5′); 6.01 (d, 1H, $J_{OH,3'}$ = 5.3, OH-3′); 6.93 (dd, 1H, $J_{H,F}$ = 20.3, $J_{1',2'}$ = 4.5, H-1'); 7.56 (dd, 1H, $J_{7,8}$ = 8.9, $J_{7,5}$ = 2.2, H-7); 8.01 (d, 1H, $J_{5,7}$ = 2.2, H-5); 8.04 (dd, 1H, $J_{8,7}$ = 8.9,

$J_{H,F}$ = 3.0, H-8); 8.89 (s, 1H, H-2); ^{13}C NMR (125.7 MHz, DMSO-d_6): 12.16 (CH_3S); 60.50 (CH_2-5'); 74.44 (d, $J_{C,F}$ = 24.1, CH-3'); 82.93 (d, $J_{C,F}$ = 17.5, CH-1'); 83.44 (d, $J_{C,F}$ = 4.6, CH-4'); 98.33 (d, $J_{C,F}$ = 192.1, CH-2'); 109.54 (C-4a); 116.81 (d, $J_{C,F}$ = 5.2, CH-8); 120.73 (C-4b); 121.48 (CH-5); 126.82 (C-6); 127.22 (CH-7); 136.83 (C-8a); 153.19 (C-9a); 154.57 (CH-2); 163.19 (C-4); ^{19}F NMR (470.3 MHz, DMSO-d_6): –188.90 (ddd, $J_{F,H2'}$ = 53.2, $J_{F,H3'}$ = 23.6, $J_{F,H1'}$ = 20.3); IR (ATR): ν = 3254, 1555, 1470, 1436, 1309, 1234, 1079, 1043, 1007, 849, 729, 541, 513 cm^{-1}; ESI MS m/z (rel. %): 406 (100) [M+Na], 384 (49) [M+H]; HR MS (ESI) for $C_{16}H_{15}O_3N_3ClFNaS$ $[M+Na]^+$: calcd 406.03989; found 406.03996.

6-Chloro-9-(2-deoxy-2-fluoro-*β*-D-arabinofuranosyl)-4-methyl-9*H*-pyrimido-[4,5-*b*]indole (9d)

Overnight deprotection of **8d** (100 mg; 0.18 mmol) according to the general procedure B and purification by RP-HPFC (C18 column, 20→60% MeOH in H_2O) afforded compound **9d** (49 mg; 78%) as a white solid: m.p. 211–216 °C; $[\alpha]_D^{20}$ +33.0 (c 0.29, DMSO); ^{1}H NMR (500.0 MHz, DMSO-d_6): 2.95 (s, 3H, CH_3); 3.78 (bdd, 1H, J_{gem} = 11.9, $J_{5'b,4'}$ = 4.8, H-5'b); 3.83 (bdd, 1H, J_{gem} = 11.9, $J_{5'a,4'}$ = 3.4, H-5'a); 3.88 (ddd, 1H, $J_{4',3'}$ = 5.9, $J_{4',5'}$ = 4.8, 3.4, H-4'); 4.50 (ddd, 1H, $J_{H,F}$ = 23.5, $J_{3',4'}$ = 5.9, $J_{3',2'}$ = 2.5, H-3'); 5.20 (bs, 1H, OH-5'); 5.21 (ddd, 1H, $J_{H,F}$ = 53.3, $J_{2',1'}$ = 4.5, $J_{2',3'}$ = 2.5, H-2'); 5.99 (bs, 1H, OH-3'); 6.96 (dd, 1H, $J_{H,F}$ = 20.5, $J_{1',2'}$ = 4.5, H-1'); 7.55 (dd, 1H, $J_{7,8}$ = 8.9, $J_{7,5}$ = 2.1, H-7); 8.04 (dd, 1H, $J_{8,7}$ = 8.9, $J_{H,F}$ = 3.0, H-8); 8.18 (d, 1H, $J_{5,7}$ = 2.1, H-5); 8.90 (s, 1H, H-2); ^{13}C NMR (125.7 MHz, DMSO-d_6): 22.91 (CH_3); 60.23 (CH_2-5'); 74.22 (d, $J_{C,F}$ = 24.0, CH-3'); 82.51 (d, $J_{C,F}$ = 17.6, CH-1'); 83.12 (d, $J_{C,F}$ = 4.8, CH-4'); 98.08 (d, $J_{C,F}$ = 192.1, CH-2'); 111.35 (C-4a); 116.31 (d, $J_{C,F}$ = 5.2, CH-8); 121.18 (C-4b); 121.97 (CH-5); 126.48 (C-6); 127.10 (CH-7); 137.05 (C-8a); 154.40 (C-9a); 154.59 (CH-2); 161.03 (C-4); ^{19}F{^{1}H} NMR (470.3 MHz, DMSO-d_6): –188.81; IR (ATR): ν = 3259, 2928, 2875, 1590, 1477, 1130, 1083, 814, 795, 590, 531 cm^{-1}; ESI MS m/z (rel.%): 352.1 (18) $[M+H]^+$, 374.1 (100) $[M+Na]^+$; HR MS (ESI) for $C_{16}H_{16}ClFN_3O_3$ $[M+H]^+$: calcd 352.08592; found 352.08587.

6-Chloro-9-(2-deoxy-2-fluoro-*β*-D-arabinofuranosyl)-4-(furan-2-yl)-9*H*-pyrimido-[4,5-*b*]indole (9e)

Overnight deprotection of **8e** (212 mg; 0.35 mmol) according to the general procedure B and purification by HPFC (silica column, 0→10% MeOH in DCM) afforded compound **9e** (97 mg; 69%) as a white solid; m.p. 75–81 °C; $[\alpha]_D^{20}$ +41.3 (c 0.27, DMSO); ^{1}H NMR (499.8 MHz, DMSO-d_6): 3.77–3.92 (m, 3H, H-4',5'); 4.51 (dddd, 1H, $J_{H,F}$ = 23.7, $J_{3',4'}$ = 5.8, $J_{3',OH}$ = 5.4, $J_{3',2'}$ = 2.4, H-3'); 5.20 (t, 1H, $J_{OH,5'}$ = 5.4, OH-5'); 5.24 (ddd, 1H, $J_{H,F}$ = 53.3, $J_{2',1'}$ = 4.4, $J_{2',3'}$ = 2.4, H-2');

5.99 (d, 1H, $J_{OH,3'}$ = 5.4, OH-3'); 6.91 (dd, 1H, $J_{4,3}$ = 3.5, $J_{4,5}$ = 1.8, H-4-furyl); 7.03 (dd, 1H, $J_{H,F}$ = 21.0, $J_{1',2'}$ = 4.4, H-1'); 7.60 (dd, 1H, $J_{7,8}$ = 8.9, $J_{7,5}$ = 2.2, H-7); 7.62 (dd, 1H, $J_{3,4}$ = 3.5, $J_{3,5}$ = 0.9, H-3-furyl); 8.08 (dd, 1H, $J_{8,7}$ = 8.9, $J_{H,F}$ = 3.2, H-8); 8.36 (dd, 1H, $J_{5,4}$ = 1.8, $J_{5,3}$ = 0.9, H-5-furyl); 8.80 (d, 1H, $J_{5,7}$ = 2.2, H-5); 9.00 (s, 1H, H-2); ^{13}C NMR (125.7 MHz, DMSO-d_6): 60.21 (CH_2-5'); 74.27 (d, $J_{C,F}$ = 24.2, CH-3'); 82.68 (d, $J_{C,F}$ = 17.6, CH-1'); 83.23 (d, $J_{C,F}$ = 4.7, CH-4'); 98.13 (d, $J_{C,F}$ = 192.1, CH-2'); 106.88 (C-4a); 113.32 (CH-4-furyl); 115.63 (CH-3-furyl); 116.30 (d, $J_{C,F}$ = 5.4, CH-8); 120.46 (C-4b); 123.37 (CH-5); 126.54 (C-6); 127.70 (CH-7); 137.71 (C-8a); 147.07 (CH-5-furyl); 147.98 (C-4); 152.29 (C-2-furyl); 154.50 (CH-2); 156.19 (C-9a); ^{19}F{^{1}H} NMR (470.3 MHz, DMSO-d_6): –188.43; IR (ATR): ν = 3181, 2918, 1470, 1446, 1073, 1054, 1040, 1013, 793, 743 cm^{-1}. ESI MS *m/z* (rel.%): 404.0 (45) [M+H]$^+$, 425.9 (100) [M+Na]$^+$; HR MS (ESI) for $C_{19}H_{16}ClFN_3O_4$ [M+H]$^+$: calcd 404.08079; found 404.08078.

6-Chloro-9-(2-deoxy-2-fluoro-*β*-D-arabinofuranosyl)-4-(furan-3-yl)-9*H*-pyrimido-[4,5-*b*]indole (9f)

Compound **8f** (164 mg; 0.27 mmol) was deprotected overnight according to the general procedure B. Purification by HPFC (silica column, 0→10% MeOH in DCM) and then by RP-HPFC (C18 column, 20→60% MeOH in H_2O) gave compound **9f** (37 mg; 33%) as a white solid: m.p. 208–214 °C; $[\alpha]_D^{20}$ +58.9 (c 0.27, DMSO); ^{1}H NMR (500.0 MHz, DMSO-d_6): 3.76–3.87 (m, 2H, H-5'); 3.90 (m, 1H, H-4'); 4.51 (m, 1H, H-3'); 5.20 (t, 1H, $J_{OH,5'}$ = 5.6, OH-5'); 5.24 (ddd, 1H, $J_{H,F}$ = 53.4, $J_{2',1'}$ = 4.4, $J_{2',3'}$ = 2.5, H-2'); 6.00 (d, 1H, $J_{OH,3'}$ = 5.2, OH-3'); 7.02 (dd, 1H, $J_{H,F}$ = 20.6, $J_{1',2'}$ = 4.4, H-1'); 7.14 (dd, 1H, $J_{4,5}$ = 1.9, $J_{4,2}$ = 0.9, H-4-furyl); 7.57 (dd, 1H, $J_{7,8}$ = 9.0, $J_{7,5}$ = 2.1, H-7); 8.02 (dd, 1H, $J_{5,4}$ = 1.9, $J_{5,2}$ = 1.5, H-5-furyl); 8.07 (dd, 1H, $J_{8,7}$ = 9.0, $J_{H,F}$ = 3.1, H-8); 8.08 (d, 1H, $J_{5,7}$ = 2.1, H-5); 8.56 (dd, 1H, $J_{2,5}$ = 1.5, $J_{2,4}$ = 0.9, H-2-furyl); 9.04 (s, 1H, H-2); ^{13}C NMR (125.7 MHz, DMSO-d_6): 60.21 (CH_2-5'); 74.23 (d, $J_{C,F}$ = 24.1, CH-3'); 82.64 (d, $J_{C,F}$ = 17.5, CH-1'); 83.22 (d, $J_{C,F}$ = 4.6, CH-4'); 98.10 (d, $J_{C,F}$ = 192.1, CH-2'); 109.98 (C-4a); 110.73 (CH-4-furyl); 116.57 (d, $J_{C,F}$ = 5.1, CH-8); 120.49 (C-4b); 121.21 (CH-5); 124.28 (C-3-furyl); 126.23 (C-6); 127.61 (CH-7); 137.41 (C-8a); 144.60 (CH-2-furyl); 144.89 (CH-5-furyl); 153.17 (C-4); 154.75 (CH-2); 155.44 (C-9a); ^{19}F{^{1}H} NMR (470.3 MHz, DMSO-d_6): –188.62; IR (ATR): ν = 3266, 2926, 1593, 1477, 1296, 1057, 807, 602 cm^{-1}; ESI MS m/z (rel.%): 404.1 (32) [M+H]$^+$, 426.1 (100) [M+Na]$^+$; HR MS (ESI) for $C_{19}H_{16}ClFN_3O_4$ [M+H]$^+$: calcd 404.08089; found 404.08079.

6-Chloro-9-(2-deoxy-2-fluoro-*β*-D-arabinofuranosyl)-4-(thiophen-2-yl)-9*H*-pyrimido-[4,5-*b*]indole (9g)

Deprotection of **8g** (170 mg; 0.27 mmol) according to the general procedure B over 2 hours, RP-HPFC (C18 column, 10→100% MeOH in H_2O) and recrystallization from a H_2O/MeOH mixture afforded compound **9g** (88 mg; 78%) as white crystals: m.p. 225–227 °C; $[\alpha]_D^{20}$ +72.3 (c 0.20, DMSO); ^1H NMR (500.0 MHz, DMSO-d_6): 3.76–3.87 (m, 2H, H-5'); 3.90 (m, 1H, H-4'); 4.51 (dddd, 1H, $J_{H,F}$ = 23.7, $J_{3',4'}$ = 5.8, $J_{3',OH}$ = 5.4, $J_{3',2'}$ = 2.5, H-3'); 5.21 (t, 1H, $J_{OH,5'}$ = 5.5, OH-5′); 5.25 (ddd, 1H, $J_{H,F}$ = 53.2, $J_{2',1'}$ = 4.4, $J_{2',3'}$ = 2.5, H-2'); 6.01 (d, 1H, $J_{OH,3'}$ = 5.4, OH-3′); 7.03 (dd, 1H, $J_{H,F}$ = 20.8, $J_{1',2'}$ = 4.4, H-1'); 7.43 (dd, 1H, $J_{4,5}$ = 5.0, $J_{4,3}$ = 3.7, H-4-thienyl); 7.56 (dd, 1H, $J_{7,8}$ = 8.9, $J_{7,5}$ = 2.2, H-7); 8.01 (dd, 1H, $J_{5,4}$ = 5.0, $J_{5,3}$ = 1.1, H-5-thienyl); 8.09 (dd, 1H, $J_{8,7}$ = 8.9, $J_{H,F}$ = 3.1, H-8); 8.10 (dd, 1H, $J_{3,4}$ = 3.7, $J_{3,5}$ = 1.1, H-3-thienyl); 8.22 (d, 1H, $J_{5,7}$ = 2.2, H-5); 9.02 (s, 1H, H-2); ^{13}C NMR (125.7 MHz, DMSO-d_6): 60.21 (CH_2-5'); 74.24 (d, $J_{C,F}$ = 24.1, CH-3'); 82.73 (d, $J_{C,F}$ = 17.5, CH-1'); 83.27 (d, $J_{C,F}$ = 4.6, CH-4'); 98.13 (d, $J_{C,F}$ = 192.1, CH-2'); 108.69 (C-4a); 116.73 (d, $J_{C,F}$ = 5.4, CH-8); 120.41 (C-4b); 120.97 (CH-5); 126.24 (C-6); 127.85 (CH-7); 128.72 (CH-4-thienyl); 130.14 (CH-3-thienyl); 131.72 (CH-5-thienyl); 137.54, (C-8a); 140.93 (C-2-thienyl); 153.48 (C-4); 154.53 (CH-2); 155.89 (C-9a); ^{19}F NMR (470.3 MHz, DMSO-d_6): –188.49 (ddd, $J_{F,H2'}$ = 53.2, $J_{F,H3'}$ = 23.7, $J_{F,H1'}$ = 20.8); IR (ATR): ν = 3132, 1560, 1440, 1402, 1159, 1044, 1003, 856, 833, 806, 731, 547, 469 cm^{-1}; ESI MS m/z (rel. %): 442 (100) $[M+Na]^+$, 420 (80) $[M+H]^+$; HR MS (ESI) for $C_{19}H_{15}ClFN_3O_3NaS$ $[M+Na]^+$: calcd 442.03989; found 442.03988.

6-Chloro-9-(2-deoxy-2-fluoro-*β*-D-arabinofuranosyl)-4-(thiophen-3-yl)-9*H*-pyrimido-[4,5-*b*]indole (9h)

Deprotection of **8h** (160 mg; 0.25 mmol) according to the general procedure B over 2 hours, RP-HPFC (C18 column, 10→100% MeOH in H_2O) and recrystallization from a H_2O/MeOH mixture afforded compound **9h** (68 mg; 65%) as a beige crystalline solid: m.p. 240–241 °C; $[\alpha]_D^{20}$ +73.2 (c 0.24, DMSO); ^1H NMR (500.0 MHz, DMSO-d_6): 3.76–3.87 (m, 2H, H-5'); 3.90 (m, 1H, H-4'); 4.52 (dddd, 1H, $J_{H,F}$ = 23.7, $J_{3',4'}$ = 5.8, $J_{3',OH}$ = 5.4, $J_{3',2'}$ = 2.5, H-3'); 5.20 (t, 1H, $J_{OH,5'}$ = 5.6, OH-5′); 5.25 (ddd, 1H, $J_{H,F}$ = 53.2, $J_{2',1'}$ = 4.4, $J_{2',3'}$ = 2.5, H-2'); 6.01 (d, 1H, $J_{OH,3'}$ = 5.4, OH-3′); 7.03 (dd, 1H, $J_{H,F}$ = 20.7, $J_{1',2'}$ = 4.4, H-1'); 7.56 (dd, 1H, $J_{7,8}$ = 8.9, $J_{7,5}$ = 2.2, H-7); 7.68 (dd, 1H, $J_{4,5}$ = 5.0, $J_{4,2}$ = 1.3, H-4-thienyl); 7.88 (dd, 1H, $J_{5,4}$ = 5.0, $J_{5,2}$ = 2.9, H-5-thienyl); 7.93 (d, 1H, $J_{5,7}$ = 2.2, H-5); 8.07 (dd, 1H, $J_{8,7}$ = 8.9, $J_{H,F}$ = 3.1, H-8); 8.34 (dd, 1H, $J_{2,5}$ = 2.9, $J_{2,4}$ = 1.3, H-2-thienyl); 9.06 (s, 1H, H-2); ^{13}C NMR (125.7 MHz, DMSO-d_6): 60.26 (CH_2-5'); 74.27 (d, $J_{C,F}$ = 24.1, CH-3'); 82.68 (d, $J_{C,F}$ = 17.5, CH-1'); 83.26 (d, $J_{C,F}$ = 4.6, CH-4'); 98.15 (d, $J_{C,F}$ = 192.1, CH-2'); 109.96 (C-4a); 116.66 (d, $J_{C,F}$ = 5.3, CH-8); 120.63 (C-4b); 121.10 (CH-5); 126.16 (C-6); 127.70 (CH-7); 127.95 (CH-5-thienyl); 128.22 (CH-4-thienyl); 128.80 (CH-2-thienyl); 137.50, (C-8a); 139.09 (C-3-thienyl); 154.79 (CH-2); 155.60, 155.65 (C-4,9a); ^{19}F NMR (470.3 MHz,

DMSO-d_6): –188.58 (ddd, $J_{F,H2'}$ = 53.2, $J_{F,H3'}$ = 23.7, $J_{F,H1'}$ = 20.7); IR (ATR): ν = 3123, 1564, 1444, 1156, 1042, 1009, 802, 718, 694, 614, 589, 547, 506, 474 cm^{-1}; ESI MS m/z (rel. %): 420 (100) $[M+H]^+$, 442 (70) $[M+Na]^+$; HR MS (ESI) for $C_{19}H_{16}ClFN_3O_3S$ $[M+H]^+$: calcd 420.05794; found 420.05789.

6-Chloro-9-(2-deoxy-2-fluoro-*β*-D-arabinofuranosyl)-4-phenyl-9*H*-pyrimido-[4,5-*b*]indole (9i)

Deprotection of **8i** (165 mg; 0.27 mmol) was carried out according to the general procedure over 2 h. Purification by RP-HPFC (C18 column, 10→100% MeOH in H_2O) and recrystallization from a H_2O/MeOH mixture afforded compound **9i** (68 mg; 65%) as a yellowish solid: m.p. 214–216 °C; $[\alpha]_D^{20}$ +68.2 (c 0.25, DMSO); ^1H NMR (500.0 MHz, DMSO-d_6): 3.76–3.87 (m, 2H, H-5'); 3.91 (m, 1H, H-4'); 4.52 (dddd, 1H, $J_{H,F}$ = 23.5, $J_{3',4'}$ = 5.6, $J_{3',OH}$ = 4.6, $J_{3',2'}$ = 2.5, H-3'); 5.21 (t, 1H, $J_{OH,5'}$ = 5.6, OH-5′); 5.26 (ddd, 1H, $J_{H,F}$ = 53.3, $J_{2',1'}$ = 4.4, $J_{2',3'}$ = 2.5, H-2'); 6.02 (d, 1H, $J_{OH,3'}$ = 4.6, OH-3′); 7.04 (dd, 1H, $J_{H,F}$ = 20.7, $J_{1',2'}$ = 4.4, H-1'); 7.55 (dd, 1H, $J_{7,8}$ = 8.9, $J_{7,5}$ = 2.2, H-7); 7.66–7.72 (m, 4H, H-5, H-*m,p*-Ph); 7.88–7.92 (m, 2H, H-*o*-Ph); 8.07 (dd, 1H, $J_{8,7}$ = 8.9, $J_{H,F}$ = 3.1, H-8); 9.12 (s, 1H, H-2); ^{13}C NMR (125.7 MHz, DMSO-d_6): 60.29 (CH_2-5'); 74.29 (d, $J_{C,F}$ = 24.1, CH-3'); 82.72 (d, $J_{C,F}$ = 17.5, CH-1'); 83.31 (d, $J_{C,F}$ = 4.6, CH-4'); 98.15 (d, $J_{C,F}$ = 192.1, CH-2'); 110.18 (C-4a); 116.78 (d, $J_{C,F}$ = 5.3, CH-8); 120.58 (C-4b); 120.91 (CH-5); 126.10 (C-6); 127.77 (CH-7); 128.99 (CH-*o*-Ph); 129.12 (CH-*m*-Ph); 130.77 (CH-*p*-Ph); 137.58, 137.63 (C-8a, C-*i*-Ph); 154.92 (CH-2); 155.54 (C-9a); 160.36 (C-4); ^{19}F NMR (470.3 MHz, DMSO-d_6): –188.57 (ddd, $J_{F,H2'}$ = 53.3, $J_{F,H3'}$ = 23.5, $J_{F,H1'}$ = 20.7); IR (ATR): ν = 3426, 1555, 1436, 1097, 1068, 850, 813, 786, 768, 703, 598, 555, 478, 455 cm^{-1}; ESI MS m/z (rel. %): 414 (100) $[M+H]^+$, 436 (41) $[M+Na]^+$; HR MS (ESI) for $C_{21}H_{17}ClFN_3O_3Na$ $[M+Na]^+$: calcd 436.08347; found 436.08343.

4,6-Dichloro-9-(2,3-*O*-isopropylidene-5-*O*-tert-butyldimethylsilyl-*β*-D-ribofuranosyl)-9*H*-pyrimido[4,5-*b*]indole (11)

To a solution of protected ribose **10**[3] (960 mg, 3.2 mmol) and CCl_4 (0.45 mL, 6.7 mmol) in anhydrous toluene (10 mL) at –30 °C, tris(dimethylamino)phosphine (0.5 mL, 2.8 mmol) was added dropwise. After 10 min of vigorous stirring at –30 °C, the reaction mixture was quickly washed with ice-cold brine (10 mL), dried over $MgSO_4$ and added to a vigorously stirred suspension of pyrimidoindole **5**[1], powdered KOH (355 mg, 6.3 mmol), and TDA-1 (0.7 mL, 2.1 mmol) in anhydrous toluene (15 mL). The reaction mixture was stirred for 24 hours at r.t. After filtration and evaporation of solvents under reduced pressure, the reaction mixture was purified by

HPFC (silica column, 0→5 % EtOAc in PE) to give the crude nucleoside **11** (400 mg) as a yellow oil, which was used directly in the next step.

^{1}H NMR (500.0 MHz, DMSO-d_6): –0.054, –0.048 (2 × s, 2 × 3H, CH_3Si); 0.81 (s, 9H, $(CH_3)_3CSi$); 1.32, 1.58 (2 × s, 2 × 3H, $(CH_3)_3C$); 3.73 (dd, 1H, J_{gem} = 11.3, $J_{5'b,4'}$ = 5.3, H-5'b); 3.81 (dd, 1H, J_{gem} = 11.3, $J_{5'a,4'}$ = 4.4, H-5'a); 4.18 (ddd, 1H, $J_{4',5'}$ = 5.3, 4.4, $J_{4',3'}$ = 4.2, H-4'); 5.10 (dd, 1H, $J_{3',2'}$ = 6.7, $J_{3',4'}$ = 4.2, H-3'); 5.54 (dd, 1H, $J_{2',3'}$ = 6.7, $J_{2',1'}$ = 3.2, H-2'); 6.64 (d, 1H, $J_{1',2'}$ = 3.2, H-1'); 7.67 (dd, 1H, $J_{7,8}$ = 8.9, $J_{7,5}$ = 2.1, H-7); 8.02 (d, 1H, $J_{8,7}$ = 8.9, H-8); 8.33 (d, 1H, $J_{5,7}$ = 2.1, H-5); 8.93 (s, 1H, H-2); ^{13}C NMR (125.7 MHz, DMSO-d_6): –5.32 (CH_3Si); 18.23 ($(CH_3)_3\mathbf{C}Si$); 25.50 ($(\mathbf{C}H_3)_2C$); 25.91 ($(\mathbf{C}H_3)_3CSi$); 27.29 ($(\mathbf{C}H_3)_2C$); 62.80 (CH_2-5'); 80.27 (CH-3'); 82.25 (CH-2'); 85.57 (CH-4'); 88.48 (CH-1'); 111.32 (C-4a); 114.32 ($(CH_3)_2\mathbf{C}$); 114.46 (CH-8); 119.59 (C-4b); 121.92 (CH-5); 127.50 (C-6); 128.69 (CH-7); 136.58 (C-8a); 152.55 (C-4); 154.70 (CH-2); 155.36 (C-9a); ESI MS m/z (rel. %): 524 (6) $[M+H]^+$, 546 (25) $[M+Na]^+$; HR MS (ESI) for $C_{24}H_{31}O_4N_3Cl_2NaSi$ $[M+Na]^+$: calcd 546.13531; found 546.13532.

4,6-Dichloro-9-(*β*-D-ribonofuranosyl)-9*H*-pyrimido[4,5-*b*]indole (12)

Crude nucleoside **11** (400 mg) was treated with aqueous TFA (90% *v*/*v*, 5 mL) and stirred at r.t. for 30 min.Volatiles were removed under reduced pressure and the residue was co-evaporated few times with MeOH. Crystallization from H_2O/MeOH mixture gave the free nucleoside **12** (225 mg, 29% over 2 steps) as a white solid: m.p. 253–256 °C; $[\alpha]_D^{20}$ –49.3 (c 0.21, DMSO); ^{1}H NMR (500.0 MHz, DMSO-d_6): 3.69 (dd, 1H, J_{gem} = 12.0, $J_{5'b,4'}$ = 3.7, H-5′b); 3.72 (dd, 1H, J_{gem} = 12.0, $J_{5'a,4'}$ = 3.3, H-5′a); 4.01 (ddd, 1H, $J_{4',5'}$ = 3.7, 3.3, $J_{4',3'}$ = 2.8, H-4′); 4.23 (dd, 1H, $J_{3',2'}$ = 5.7, $J_{3',4'}$ = 2.8, H-3′); 4.72 (dd, 1H, $J_{2',1'}$ = 7.4, $J_{2',3'}$ = 5.7, H-2′); 5.12–5.42 (bm, 3H, OH-2′,3′,5′); 6.49 (d, 1H, $J_{1',2'}$ = 7.4, H-1′); 7.69 (dd, 1H, $J_{7,8}$ = 8.9, $J_{7,5}$ = 2.2, H-7); 8.25 (d, 1H, $J_{8,7}$ = 8.9, H-8); 8.33 (d, 1H, $J_{5,7}$ = 2.2, H-5); 8.91 (s, 1H, H-2); ^{13}C NMR (125.7 MHz, DMSO-d_6): 61.62 (CH_2-5′); 70.15 (CH-3′); 70.90 (CH-2′); 85.94 (CH-4′); 87.37 (CH-1′); 111.06 (C-4a); 115.51 (CH-8); 119.65 (C-4b); 121.73 (CH-5); 127.22 (C-6); 128.60 (CH-7); 136.52 (C-8a); 152.37 (C-4); 154.61 (CH-2); 156.23 (C-9a); IR (ATR): ν = 3257, 1590, 1551, 1445, 1228, 1106, 1075, 1055, 1031, 1004, 835, 621, 430 cm^{-1}; ESI MS m/z (rel. %): 370 (100) $[M+H]^+$, 392 (67) $[M+Na]^+$; HR MS (ESI) for $C_{15}H_{14}O_4N_3Cl_2$ $[M+H]^+$: calcd 370.03559; found 370.03566.

4,6-Dichloro-9-[3,5-*O*-(tetraisopropyldisiloxan-1,3-diyl)-*β*-D-ribofuranosyl]-9*H*-pyrimido[4,5-*b*]indole (13)

Nucleoside **12** (2.7 g, 7.3 mmol) was dissolved in anhydrous pyridine (50 mL) and $TIPDSCl_2$ (2.5 mL, 7.8 mmol) was added. The mixture was stirred at r.t. for 4 h and then solvents were

removed under reduced pressure. Residue was dissolved in EtOAc (50 mL) and extracted with water (50 mL). The organic layer was dried over $MgSO_4$ and evaporated under reduced pressure. The crude product was purified by HPFC (silica column, 0→10% EtOAc in PE) to give nucleoside **13** (3.94 g, 88%) as a yellowish solid: m.p. 145–147 °C; ^{1}H NMR (500.0 MHz, $CDCl_3$): 0.88–1.22 (m, 28H, $(CH_3)_2CHSi$); 3.22 (bs, 1H, OH-2′); 4.04–4.08 (m, 3H, H-4′,5′); 4.94 (td, 1H, $J_{2',3'}$ = 6.1, $J_{2',1'}$ = 2.0, H-2′); 5.28 (m, 1H, H-3′); 6.30 (d, 1H, $J_{1',2'}$ = 2.0, H-1′); 7.57 (dd, 1H, $J_{7,8}$ = 8.8, $J_{7,5}$ = 2.1, H-7); 7.64 (d, 1H, $J_{8,7}$ = 8.8, H-8); 8.36 (d, 1H, $J_{5,7}$ = 2.1, H-5); 8.72 (s, 1H, H-2); ^{13}C NMR (125.7 MHz, $CDCl_3$): 12.58, 12.76, 13.01, 13.27 ($(CH_3)_2$**C**HSi); 16.93, 17.00, 17.02, 17.15, 17.27, 17.35, 17.38, 17.42 ((**C**$H_3)_2$CHSi); 61.60 (CH_2-5′); 70.72 (CH-3′); 73.44 (CH-2′); 81.56 (CH-4′); 89.40 (CH-1′); 111.95 (CH-8); 112.28 (C-4a); 119.80 (C-4b); 122.90 (CH-5); 128.40 (C-6); 128.79 (CH-7); 137.12 (C-8a); 153.20 (C-4); 153.92 (CH-2); 155.43 (C-9a); IR (ATR): ν = 2875, 1440, 108/7, 1032, 868, 693, 615, 449 cm^{-1}; ESI MS m/z (rel. %): 612 (100) $[M+H]^+$, 634 (85) $[M+Na]^+$; HR MS (ESI) for $C_{27}H_{40}O_5N_3Cl_2Si_2$ $[M+H]^+$: calcd 612.18781; found 612.18798.

4,6-Dichloro-9-[3,5-*O*-(tetraisopropyldisiloxan-1,3-diyl)-*β*-D-*erythro*-pentofuran-2-ulosyl]-9*H*-pyrimido[4,5-*b*]indole (14)

Dess-Martin periodinane (3.13 g, 7.4 mmol) was dissolved in anhydrous DCM (20 mL) and cooled to 0 °C and then the solution of **13** (1.51 g, 2.46 mmol) in anhydrous DCM (20 mL) was added. The reaction mixture was stirred 10 min at 0 °C and then it was allowed to warm to r.t. and stirred overnight. Reaction mixture was then diluted with DCM (60 mL) and The organic phase was washed with water, dried over $MgSO_4$ and evaporated under reduced pressure. HPFC (silica column, 0→10% EtOAc in PE) afforded compound **14** (1.37 g, 91%) as a yellowish foam: ^{1}H NMR (500.0 MHz, $CDCl_3$): 0.99–1.26 (m, 28H, $(CH_3)_2CHSi$); 4.08 (ddd, 1H, $J_{4',3'}$ = 9.9, $J_{4',5'}$ = 2.9, 2.5, H-4′); 4.15 (dd, 1H, J_{gem} = 13.2, $J_{5'b,4'}$ = 2.9, H-5′b); 4.20 (dd, 1H, J_{gem} = 13.2, $J_{5'a,4'}$ = 2.5, H-5′a); 5.62 (d, 1H, $J_{3',4'}$ = 9.9, H-3′); 6.01 (s, 1H, H-1′); 7.52 (d, 1H, $J_{8,7}$ = 8.8, H-8); 7.58 (dd, 1H, $J_{7,8}$ = 8.8, $J_{7,5}$ = 2.1, H-7); 8.34 (d, 1H, $J_{5,7}$ = 2.1, H-5); 8.60 (s, 1H, H-2); ^{13}C NMR (125.7 MHz, $CDCl_3$): 12.40, 12.49, 12.90, 13.46 ($(CH_3)_2$**C**HSi); 16.74, 16.77, 16.81, 16.91, 17.19, 17.27, 17.29, 17.31 ((**C**$H_3)_2$CHSi); 60.56 (CH_2-5′); 72.27 (CH-3′); 78.59 (CH-4′); 79.56 (CH-1′); 110.80 (CH-8); 112.32 (C-4a); 119.95 (C-4b); 123.14 (CH-5); 128.92 (C-6); 128.99 (CH-7); 137.15 (C-8a); 153.31 (C-4); 153.77 (CH-2); 155.26 (C-9a); 206.22 (C-3′); IR (ATR): ν = 2945, 2868, 1786, 1584, 1553, 1460, 1435, 1171, 1130, 1101, 1075, 1027, 886, 852, 694 cm^{-1}; ESI MS m/z (rel. %): 610 (23) $[M+H]^+$, 632 (12) $[M+Na]^+$; HR MS (ESI) for $C_{27}H_{38}O_5N_3Cl_2Si_2$ $[M+H]^+$: calcd 610.17216; found 610.17223.

4,6-Dichloro-9-[3,5-*O*-(tetraisopropyldisiloxan-1,3-diyl)-*β*-D-arabinofuranosyl]-9*H*-pyrimido[4,5-*b*]indole (15)

Compound **14** (3.55 g, 5.8 mmol) was dissolved in ethanol (99%, 100 mL) and cooled to 0 °C. Then the solution of sodium borohydride (440 mg, 11.6 mmol) in ethanol (99%, 100 mL) was slowly added and the reaction mixture was stirred at r.t. for 1.5 h. Then, aqueous NH_4Cl (saturated, 60 mL) was added and the reaction mixture was extracted with EtOAc (300 mL). The organic layer was washed with water (150 mL), dried over $MgSO_4$ and evaporated under reduced pressure. HPFC (SiO_2, 0→15% EtOAc in PE) gave arabinonucleoside **15** (3.37 g, 95%) as a white foam: ^1H NMR (500.0 MHz, $CDCl_3$): 0.97–1.18 (m, 28H, $(CH_3)_2CHSi$); 3.81 (ddd, 1H, $J_{4',3'}$ = 7.7, $J_{4',5'}$ = 4.1, 3.4, H-4′); 3.98 (dd, 1H, J_{gem} = 12.7, $J_{5'b,4'}$ = 4.1, H-5′b); 4.03 (dd, 1H, J_{gem} = 12.7, $J_{5'a,4'}$ = 3.4, H-5′a); 4.76 (t, 1H, $J_{2',1'}$ = $J_{2',3'}$ = 6.6, H-2′); 4.85 (dd, 1H, $J_{3',4'}$ = 7.7, $J_{3',2'}$ = 6.6, H-3′); 6.55 (d, 1H, $J_{1',2'}$ = 6.6, H-1′); 7.56 (dd, 1H, $J_{7,8}$ = 8.9, $J_{7,5}$ = 2.1, H-7); 7.82 (d, 1H, $J_{8,7}$ = 8.9, H-8); 8.36 (d, 1H, $J_{5,7}$ = 2.1, H-5); 8.72 (s, 1H, H-2); ^{13}C NMR (125.7 MHz, $CDCl_3$): 12.45, 13.01, 13.08, 13.55 ($(CH_3)_2$**C**HSi); 17.01, 17.04, 17.09, 17.10, 17.39, 17.45, 17.54 ((**C**$H_3)_2$CHSi); 61.18 (CH_2-5′); 76.40 (CH-3′); 78.35 (CH-2′); 80.49 (CH-4′); 83.83 (CH-1′); 112.55 (C-4a); 113.80 (CH-8); 119.97 (C-4b); 122.72 (CH-5); 128.65 (C-6); 129.06 (CH-7); 138.20 (C-8a); 153.46 (CH-2); 153.53 (C-4); 155.39 (C-9a); IR (ATR): ν = 2944, 2867, 1750, 1581, 1546, 1460, 1437, 1223, 1153, 1098, 1032, 1010, 885, 837, 793, 694 cm^{-1}; ESI MS m/z (rel. %): 612 (46) $[M+H]^+$, 634 (100) $[M+Na]^+$; HR MS (ESI) for $C_{27}H_{39}O_5N_3Cl_2NaSi_2$ $[M+Na]^+$: calcd 634.16975; found 634.16989.

4,6-Dichloro-9-(*β*-D-arabinofuranosyl)-9*H*-pyrimido[4,5-*b*]indole (16)

$Et_3N·3HF$ (660 μl, 4 mmol) was added to a solution of silyl-protected nucleoside **15** (1.2 g, 2 mmol) in anhydrous THF (30 mL). The reaction mixture was stirred overnight at r.t. and evaporated under reduced pressure. RP-HPFC (C18 column, 10→100% MeOH in H_2O) and recrystallization from a H_2O/MeOH mixture afforded the free arabinonucleoside **16** (705 mg, 95%) as a white solid: m.p. 217–219 °C; $[α]_D^{20}$ –19.1 (c 0.20, DMSO); ^1H NMR (500.0 MHz, DMSO-*d*$_6$): 3.76–3.88 (m, 3H, H-4′,5′); 4.15 (td, 1H, $J_{3',4'}$ = $J_{3',OH}$ = 4.8, $J_{3',2'}$ = 3.3, H-3′); 4.23 (td, 1H, $J_{2',1'}$ = $J_{2',OH}$ = 4.8, $J_{2',3'}$ = 3.3, H-2′); 5.16 (t, 1H, $J_{OH,5'}$ = 5.3, OH-5′); 5.33 (d, 1H, $J_{OH,2'}$ = 4.8, OH-2′); 5.62 (d, 1H, $J_{OH,3'}$ = 4.8, OH-3′); 6.81 (d, 1H, $J_{1',2'}$ = 4.9, H-1′); 7.62 (dd, 1H, $J_{7,8}$ = 9.0, $J_{7,5}$ = 2.2, H-7); 8.16 (d, 1H, $J_{8,7}$ = 9.0, H-8); 8.26 (d, 1H, $J_{5,7}$ = 2.2, H-5); 8.89 (s, 1H, H-2); ^{13}C NMR (125.7 MHz, DMSO-*d*$_6$): 60.98 (CH_2-5′); 76.44 (CH-3′); 77.69 (CH-2′); 84.18 (CH-4′); 86.02 (CH-1′); 110.88 (C-4a); 117.86 (CH-8); 119.29 (C-4b); 121.02 (CH-5); 126.63 (C-6); 128.12 (CH-7); 138.42 (C-8a); 152.02 (C-4); 154.53 (CH-2); 155.58 (C-9a); IR (ATR): ν = 3302, 1590, 1442,

1296, 1219, 1157, 1064, 1033, 830, 566, 426 cm^{-1}; ESI MS m/z (rel. %): 392 (100) $[M+Na]^+$; HR MS (ESI) for $C_{15}H_{13}O_4N_3Cl_2Na$ $[M+Na]^+$: calcd 392.01753; found 392.01764.

4-Amino-9-(*β*-D-arabinofuranosyl)-6-chloro-9*H*-pyrimido[4,5-*b*]indole (17a)

Arabinoside **16** (120 mg, 0.32 mmol) was dissolved in dioxane (3 mL) and aqueous ammonia (30%, 3 mL) was added. The reaction mixture was stirred in screw-cap pressure glass tube at 100 °C for 20 h and then solvents were evaporated under reduced pressure. RP-HPFC (C18 column, 10→100% MeOH in H_2O) and recrystallization from a H_2O/MeOH mixture afforded nucleoside **17a** (95 mg, 85%) as a white solid: m.p. 298–301 °C; $[\alpha]_D^{20}$ 0 (c 0.23, DMSO); ^1H NMR (500.0 MHz, DMSO-d_6): 3.69–3.85 (m, 3H, H-4′,5′); 4.10–4.17 (m, 2H, H-2′,3′); 5.14 (t, 1H, $J_{OH,5'}$ = 5.2, OH-5′); 5.33 (d, 1H, $J_{OH,2'}$ = 5.2, OH-2′); 5.51 (d, 1H, $J_{OH,3'}$ = 4.7, OH-3′); 6.69 (d, 1H, $J_{1',2'}$ = 4.7, H-1′); 7.30 (dd, 1H, $J_{7,8}$ = 8.9, $J_{7,5}$ = 2.2, H-7); 7.37 (bs, 2H, NH_2); 7.92 (d, 1H, $J_{8,7}$ = 8.9, H-8); 8.30 (s, 1H, H-2); 8.42 (d, 1H, $J_{5,7}$ = 2.2, H-5); ^{13}C NMR (125.7 MHz, DMSO-d_6): 61.24 (CH_2-5′); 76.73 (CH-3′); 77.67 (CH-2′); 83.75 (CH-4′); 85.32 (CH-1′); 94.95 (C-4a); 116.22 (CH-8); 120.06 (CH-5); 121.56 (C-4b); 123.99 (CH-7); 125.41 (C-6); 136.09 (C-8a); 155.21 (CH-2); 155.53 (C-9a); 157.78 (C-4); IR (ATR): ν = 3120, 1654, 1597, 1460, 1319, 1218, 1033, 907, 855, 795, 525 cm^{-1}; ESI MS m/z (rel. %): 351 (100) $[M+H]^+$, 373 (56) $[M+Na]^+$; HR MS (ESI) for $C_{15}H_{16}O_4N_4Cl$ $[M+H]^+$: calcd 351.08546; found 351.08557.

9-(*β*-D-Arabinofuranosyl)-6-chloro-4-methoxy-9*H*-pyrimido[4,5-*b*]indole (17b)

Nucleoside **16** (80 mg, 0.22 mmol) was dissolved in dry MeOH (5 mL) and solution of sodium methoxide (1M in MeOH, 2 mL) was added. The mixture was stirred for 3 h at r.t. Solvent evaporation, purification by RP-HPFC (C18 column, 10→100% MeOH in H_2O) and recrystallization from a H_2O/MeOH mixture gave nucleoside **17b** (62 mg, 77%) as a white solid: m.p. 238–241 °C; $[\alpha]_D^{20}$ –8.4 (c 0.30, DMSO); ^1H NMR (500.0 MHz, DMSO-d_6): 3.75–3.85 (m, 3H, H-4′,5′); 4.15 (td, 1H, $J_{3',4'}$ = $J_{3',OH}$ = 4.8, $J_{3',2'}$ = 3.3, H-3′); 4.20 (td, 1H, $J_{2',1'}$ = $J_{2',OH}$ = 4.9, $J_{2',3'}$ = 3.3, H-2′); 4.20 (s, 3H, CH_3O); 5.32 (t, 1H, $J_{OH,5'}$ = 5.1, OH-5′); 5.32 (d, 1H, $J_{OH,2'}$ = 4.9, OH-2′); 5.55 (d, 1H, $J_{OH,3'}$ = 4.8, OH-3′); 6.77 (d, 1H, $J_{1',2'}$ = 4.9, H-1′); 7.45 (dd, 1H, $J_{7,8}$ = 8.9, $J_{7,5}$ = 2.2, H-7); 7.95 (d, 1H, $J_{5,7}$ = 2.2, H-5); 8.04 (d, 1H, $J_{8,7}$ = 8.9, H-8); 8.68 (s, 1H, H-2); ^{13}C NMR (125.7 MHz, DMSO-d_6): 54.38 (CH_3O); 61.12 (CH_2-5′); 76.57 (CH-3′); 77.67 (CH-2′); 83.93 (CH-4′); 85.65 (CH-1′); 98.31 (C-4a); 117.22 (CH-8); 120.15 (C-4b); 120.61 (CH-5); 125.68 (CH-7); 125.82 (C-6); 136.92 (C-8a); 154.85 (CH-2); 156.33 (C-9a); 163.81 (C-4); IR (ATR): ν = 3299, 1598, 1566, 1460, 1323, 1294, 1192, 1124, 1052, 943, 769, 722, 588, 560 cm^{-1}; ESI MS m/z (rel.

%): 388 (100) [M+Na]$^+$; HR MS (ESI) for $C_{16}H_{16}O_5N_3ClNa$ [M+Na]$^+$: calcd 388.06707; found 388.06722.

9-(*β*-D-Arabinofuranosyl)-6-chloro-4-methylsulfanyl-9*H*-pyrimido[4,5-*b*]indole (17c)

Nucleoside **16** (80 mg, 0.22 mmol) and sodium methanethiolate (30 mg, 0.43 mmol) were dissolved in anhydrous EtOH (10 mL) and stirred for 3 h at r.t. The solvent was removed under reduced pressure and the crude product was purified by RP-HPFC (C18 column, 10→100% MeOH in H_2O). Recrystallization from a H_2O/MeOH mixture gave compound **17c** (60 mg, 71%) as a white solid: m.p. 181–183 °C; $[\alpha]_D^{20}$ –14.9 (c 0.28, DMSO); ^{1}H NMR (500.0 MHz, DMSO-d_6): 2.80 (s, 3H, CH_3S); 3.76–3.85 (m, 3H, H-4′,5′); 4.15 (td, 1H, $J_{3',4'} = J_{3',OH} = 4.8$, $J_{3',2'} = 3.2$, H-3′); 4.20 (td, 1H, $J_{2',1'} = J_{2',OH} = 4.9$, $J_{2',3'} = 3.2$, H-2′); 5.11 (t, 1H, $J_{OH,5'} = 5.2$, OH-5′); 5.30 (d, 1H, $J_{OH,2'} = 4.9$, OH-2′); 5.56 (d, 1H, $J_{OH,3'} = 4.8$, OH-3′); 6.79 (d, 1H, $J_{1',2'} = 4.9$, H-1′); 7.52 (dd, 1H, $J_{7,8} = 8.9$, $J_{7,5} = 2.2$, H-7); 8.01 (d, 1H, $J_{5,7} = 2.2$, H-5); 8.10 (d, 1H, $J_{8,7} = 8.9$, H-8); 8.87 (s, 1H, H-2); ^{13}C NMR (125.7 MHz, DMSO-d_6): 11.83 (CH_3S); 61.05 (CH_2-5′); 76.54 (CH-3′); 77.68 (CH-2′); 83.99 (CH-4′); 85.57 (CH-1′); 109.33 (C-4a); 117.38 (CH-8); 120.28 (C-4b); 120.87 (CH-5); 125.89 (C-6); 126.40 (CH-7); 137.38 (C-8a); 153.10 (C-9a); 154.03 (CH-2); 162.32 (C-4); IR (ATR): ν = 3278, 1557, 1473, 1433, 1292, 1236, 1163, 1119, 1072, 943, 844, 802, 593, 565 cm^{-1}; ESI MS m/z (rel. %): 404 (100) [M+Na]$^+$; HR MS (ESI) for $C_{16}H_{16}O_4N_3ClNaS$ [M+Na]$^+$: calcd 404.04423; found 404.04433.

9-(*β*-D-Arabinofuranosyl)-6-chloro-4-methyl-9*H*-pyrimido[4,5-*b*]indole (17d)

Nucleoside **16** (80 mg, 0.22 mmol) and $Pd(PPh_3)_4$ (13 mg, 0.011 mmol) were dissolved in anhydrous THF (5 mL) and Me_3Al (2M in toluene, 220 μL) was added. The mixture was stirred at 70 °C for 18 h and solvents were evaporated. RP-HPFC (C18 column, 10→100% MeOH in H_2O) and recrystallization from a H_2O/MeOH mixture gave nucleoside **17d** (52 mg, 68%) as a white solid: m.p. 233–235 °C; $[\alpha]_D^{20}$ –10.7 (c 0.21, DMSO); ^{1}H NMR (500.0 MHz, DMSO-d_6): 2.97 (s, 3H, CH_3); 3.76–3.87 (m, 3H, H-4′,5′); 4.16 (dd, 1H, $J_{3',4'} = 4.5$, $J_{3',2'} = 3.3$, H-3′); 4.22 (td, 1H, $J_{2',1'} = 4.9$, $J_{2',3'} = 3.3$, H-2′); 6.81 (d, 1H, $J_{1',2'} = 4.9$, H-1′); 7.53 (dd, 1H, $J_{7,8} = 8.9$, $J_{7,5} = 2.2$, H-7); 8.11 (d, 1H, $J_{8,7} = 8.9$, H-8); 8.17 (d, 1H, $J_{5,7} = 2.2$, H-5); 8.93 (s, 1H, H-2); ^{13}C NMR (125.7 MHz, DMSO-d_6): 22.48 (CH_3O); 61.06 (CH_2-5′); 76.59 (CH-3′); 77.64 (CH-2′); 83.99 (CH-4′); 85.50 (CH-1′); 111.50 (C-4a); 117.31 (CH-8); 120.94 (C-4b); 121.67 (CH-5); 126.12 (C-6); 126.86 (CH-7); 138.01 (C-8a); 153.70 (CH-2); 154.55 (C-9a); 159.89 (C-4); IR (ATR): ν = 3271, 1564, 1475, 1068, 1030, 836, 567, 425 cm^{-1}; ESI MS m/z (rel. %): 350 (12) [M+H]$^+$, 372 (100) [M+Na]$^+$; HR MS (ESI) for $C_{16}H_{16}O_4N_3ClNa$ [M+Na]$^+$: calcd 372.07215; found 372.07232.

9-(*β*-D-Arabinofuranosyl)-6-chloro-4-(furan-2-yl)-9*H*-pyrimido[4,5-*b*]indole (17e)

Arabinoside **16** (120 mg, 0.32 mmol) was reacted with furan-2-boronic acid (54 mg, 0.48 mmol) in 3 mL of H_2O/MeCN (2:1) for 2 h according to the general procedure A. Recrystallization from a H_2O/MeOH mixture gave **17e** (42 mg, 33%) as a greenish solid: m.p. 241–243 °C; $[\alpha]_D^{20}$ –7.2 (c 0.15, DMSO); ^{1}H NMR (500.0 MHz, DMSO-d_6): 3.77–3.87 (m, 3H, H-4′,5′); 4.16 (m, 1H, H-3′); 4.24 (td, 1H, $J_{2',1'} = J_{2',OH}$ = 4.9 Hz, $J_{2',3'}$ = 3.2 Hz, H-2′); 5.14 (bs, 1H, OH-5′); 5.34 (d, 1H, $J_{OH,2'}$ = 5.0 Hz, OH-2′); 5.58 (bd, 1H, $J_{OH,3'}$ = 4.8 Hz, OH-3′); 6.88 (d, 1H, $J_{1',2'}$ = 4.9 Hz, H-1′); 6.91 (dd, 1H, $J_{4,3}$ = 3.5 Hz, $J_{4,5}$ = 1.8 Hz, H-4-furyl); 7.55 (dd, 1H, $J_{7,8}$ = 8.9 Hz, $J_{7,5}$ = 2.2 Hz, H-7); 7.61 (dd, 1H, $J_{3,4}$ = 3.5 Hz, $J_{3,5}$ = 0.9 Hz, H-3-furyl); 8.14 (dd, 1H, $J_{8,7}$ = 8.9 Hz, $J_{8,5}$ = 0.6 Hz, H-8); 8.36 (dd, 1H, $J_{5,4}$ = 1.8 Hz, $J_{5,3}$ = 0.9 Hz, H-5-furyl); 8.77 (dd, 1H, $J_{5,7}$ = 2.2 Hz, $J_{5,8}$ = 0.6 Hz, H-5); 8.97 (s, 1H, H-2); ^{13}C NMR (125.7 MHz, DMSO-d_6): 61.07 (CH_2-5′); 76.70 (CH-3′); 77.70 (CH-2′); 84.01 (CH-4′); 85.65 (CH-1′); 107.03 (C-4a); 113.33 (CH-4-furyl); 115.37 (CH-3-furyl); 117.22 (CH-8); 120.26 (C-4b); 123.02 (CH-5); 125.96 (C-6); 127.24 (CH-7); 138.60 (C-8a); 146.92 (CH-5-furyl); 144.67 (C-4); 152.50 (C-2-furyl); 154.29 (CH-2); 156.40 (C-9a); IR (ATR): ν = 3194, 1542, 1474, 1439, 1296, 1073, 1044, 1024, 799, 750, 592, 562, 524 cm^{-1}; ESI MS m/z (rel. %): 424 (100) $[M+Na]^+$; HR MS (ESI) for $C_{19}H_{16}O_5N_3ClNa$ $[M+Na]^+$: calcd 424.06707; found 424.06713.

9-(*β*-D-Arabinofuranosyl)-6-chloro-4-(furan-3-yl)-9*H*-pyrimido[4,5-*b*]indole (17f)

Arabinoside **16** (120 mg, 0.32 mmol) was reacted with furan-3-boronic acid (54 mg, 0.48 mmol) in 3 mL of H_2O/MeCN (2:1) for 2 h according to the general procedure A. Recrystallization from H_2O/MeOH mixture gave **17f** (80 mg, 62%) as a tan solid: m.p. 186–188 °C; $[\alpha]_D^{20}$ +19.1 (c 0.19, DMSO); ^{1}H NMR (500.0 MHz, DMSO-d_6): 3.76–3.87 (m, 3H, H-4′,5′); 4.16 (td, 1H, $J_{3',4'} = J_{3',OH}$ = 4.7 Hz, $J_{3',2'}$ = 3.2 Hz, H-3′); 4.24 (td, 1H, $J_{2',1'} = J_{2',OH}$ = 5.0 Hz, $J_{2',3'}$ = 3.2 Hz, H-2′); 5.12 (t, 1H, $J_{OH,5'}$ = 5.1 Hz, OH-5′); 5.34 (d, 1H, $J_{OH,2'}$ = 5.0 Hz, OH-2′); 5.57 (d, 1H, $J_{OH,3'}$ = 4.8 Hz, OH-3′); 6.87 (d, 1H, $J_{1',2'}$ = 4.9 Hz, H-1′); 7.12 (dd, 1H, $J_{4,5}$ = 1.8 Hz, $J_{4,2}$ = 0.9 Hz, H-4-furyl); 7.52 (dd, 1H, $J_{7,8}$ = 9.0 Hz, $J_{7,5}$ = 2.2 Hz, H-7); 8.02 (t, 1H, $J_{5,2} = J_{5,4}$ = 1.7 Hz, H-5-furyl); 8.05 (dd, 1H, $J_{5,7}$ = 2.2 Hz, $J_{5,8}$ = 0.5 Hz, H-5); 8.13 (dd, 1H, $J_{8,7}$ = 9.0 Hz, $J_{8,5}$ = 0.5 Hz, H-8); 8.54 (dd, 1H, $J_{2,5}$ = 1.6 Hz, $J_{2,4}$ = 0.9 Hz, H-2-furyl); 9.00 (s, 1H, H-2); ^{13}C NMR (125.7 MHz, DMSO-d_6): 61.05 (CH_2-5′); 76.64 (CH-3′); 77.66 (CH-2′); 84.01 (CH-4′); 85.57 (CH-1′); 110.05 (C-4a); 110.77 (CH-4-furyl); 117.46 (CH-8); 120.26 (C-4b); 120.82 (CH-5); 124.43 (C-3-furyl); 125.63 (C-6); 127.12 (CH-7); 138.27 (C-8a); 144.43 (CH-2-furyl); 144.89 (CH-5-furyl); 152.73 (C-4); 154.52 (CH-2); 155.62 (C-9a); IR (ATR): ν = 3363, 1565, 1477, 1451, 1113, 1073, 1056, 1026, 876, 833,

690, 642, 601, 549 cm^{-1}; ESI MS m/z (rel. %): 424 (100) [M+Na]$^+$, 402 (39) [M+H]$^+$; HR MS (ESI) for $C_{19}H_{16}O_5N_3ClNa$ [M+Na]$^+$: calcd 424.06707; found 424.06720.

9-(*β*-D-Arabinofuranosyl)-6-chloro-4-(thiophen-2-yl)-9*H*-pyrimido[4,5-*b*]indole (17g)

Arabinoside **16** (120 mg, 0.32 mmol) was reacted with thiophene-2-boronic acid (62 mg, 0.48 mmol) in 3 mL of H_2O/MeCN (2:1) for 2 h according to the general procedure A. Recrystallization from H_2O/MeOH mixture gave **17g** (94 mg, 70%) as a tan solid: m.p. 192–194 °C; $[\alpha]_D^{20}$ +18.1 (c 0.26, DMSO); ^{1}H NMR (500.0 MHz, DMSO-d_6): 3.77–3.87 (m, 3H, H-4′,5′); 4.16 (td, 1H, $J_{3',4'}$ = $J_{3',OH}$ = 4.8, $J_{3',2'}$ = 3.2, H-3′); 4.25 (td, 1H, $J_{2',1'}$ = $J_{2',OH}$ = 4.8, $J_{2',3'}$ = 3.2, H-2′); 5.13 (t, 1H, $J_{OH,5'}$ = 5.0, OH-5′); 5.35 (d, 1H, $J_{OH,2'}$ = 4.8, OH-2′); 5.59 (d, 1H, $J_{OH,3'}$ = 4.8, OH-3′); 6.88 (d, 1H, $J_{1',2'}$ = 4.8, H-1′); 7.43 (dd, 1H, $J_{4,5}$ = 5.0, $J_{4,3}$ = 3.7, H-4-thienyl); 7.54 (dd, 1H, $J_{7,8}$ = 9.0, $J_{7,5}$ = 2.2, H-7); 7.99 (dd, 1H, $J_{5,4}$ = 5.0, $J_{5,3}$ = 1.1, H-5-thienyl); 8.06 (dd, 1H, $J_{3,4}$ = 3.7, $J_{3,5}$ = 1.1, H-3-thienyl); 8.15 (d, 1H, $J_{8,7}$ = 9.0, H-8); 8.20 (d, 1H, $J_{5,7}$ = 2.2, H-5); 8.98 (s, 1H, H-2); ^{13}C NMR (125.7 MHz, DMSO-d_6): 61.06 (CH_2-5′); 76.66 (CH-3′); 77.69 (CH-2′); 84.06 (CH-4′); 85.71 (CH-1′); 108.77 (C-4a); 117.61 (CH-8); 120.16 (C-4b); 120.58 (CH-5); 125.65 (C-6); 127.36 (CH-7); 128.69 (CH-4-thienyl); 129.83 (CH-3-thienyl); 131.48 (CH-5-thienyl); 138.42 (C-8a); 141.16 (C-2-thienyl); 153.07 (C-4); 154.30 (CH-2); 156.06 (C-9a); IR (ATR): ν = 3358, 1562, 1447, 1217, 1172, 1138, 1057, 918, 707, 632, 483 cm^{-1}; ESI MS m/z (rel. %): 418 (100) [M+H]$^+$; HR MS (ESI) for $C_{19}H_{17}O_4N_3ClS$ [M+H]$^+$: calcd 418.06228; found 418.06235.

9-(*β*-D-Arabinofuranosyl)-6-chloro-4-(thiophen-3-yl)-9*H*-pyrimido[4,5-*b*]indole (17h)

Arabinoside **16** (120 mg, 0.32 mmol) was reacted with thiophene-3-boronic acid (62 mg, 0.48 mmol) in 3 mL of H_2O/MeCN (2:1) for 2 h according to the general procedure A. Recrystallization from H_2O/MeOH mixture gave **17h** (100 mg, 75%) as a white crystalline solid: m.p. 207–209 °C; $[\alpha]_D^{20}$ +9.2 (c 0.22 , DMSO); ^{1}H NMR (500.0 MHz, DMSO-d_6): 3.77–3.87 (m, 3H, H-4′,5′); 4.17 (td, 1H, $J_{3',4'}$ = $J_{3',OH}$ = 4.7 Hz, $J_{3',2'}$ = 3.2 Hz, H-3′); 4.25 (td, 1H, $J_{2',1'}$ = $J_{2',OH}$ = 5.0 Hz, $J_{2',3'}$ = 3.2 Hz, H-2′); 5.12 (bt, 1H, $J_{OH,5'}$ = 5.2 Hz, OH-5′); 5.35 (d, 1H, $J_{OH,2'}$ = 5.0 Hz, OH-2′); 5.58 (d, 1H, $J_{OH,3'}$ = 4.8 Hz, OH-3′); 6.88 (d, 1H, $J_{1',2'}$ = 4.9 Hz, H-1′); 7.51 (dd, 1H, $J_{7,8}$ = 9.0 Hz, $J_{7,5}$ = 2.2 Hz, H-7); 7.66 (dd, 1H, $J_{4,5}$ = 5.0 Hz, $J_{4,2}$ = 1.3 Hz, H-4-thienyl); 7.88 (dd, 1H, $J_{5,4}$ = 5.0 Hz, $J_{5,2}$ = 2.9 Hz, H-5-thienyl); 7.91 (dd, 1H, $J_{5,7}$ = 2.2 Hz, $J_{5,8}$ = 0.5 Hz, H-5); 8.13 (dd, 1H, $J_{8,7}$ = 9.0 Hz, $J_{8,5}$ = 0.5 Hz, H-8); 8.31 (dd, 1H, $J_{2,5}$ = 2.9 Hz, $J_{2,4}$ = 1.3 Hz, H-2-thienyl); 9.02 (s, 1H, H-2); ^{13}C NMR (125.7 MHz, DMSO-d_6): 61.07 (CH_2-5′); 76.65 (CH-3′); 77.67 (CH-2′); 84.04 (CH-4′); 85.60 (CH-1′); 109.98 (C-4a); 117.51 (CH-8); 120.36 (C-4b); 120.69 (CH-5); 125.51 (C-6); 127.17 (CH-7); 127.89 (CH-5-thienyl); 128.18 (CH-4-thienyl); 128.51 (CH-2-thienyl); 138.33 (C-

8a); 139.27 (C-3-thienyl); 154.52 (CH-2); 155.21 (C-4); 155.75 (C-9a); IR (ATR): ν = 3800, 1564, 1449, 1105, 1072, 1056, 999, 813, 777, 587, 502, 444 cm^{-1}; ESI MS m/z (rel. %): 440 (100) $[M+Na]^+$, 418 (51) $[M+H]^+$; HR MS (ESI) for $C_{19}H_{16}O_4N_3ClNaS$ $[M+Na]^+$: calcd 440.04423; found 440.04424.

9-(*β*-D-Arabinofuranosyl)-6-chloro-4-phenyl-9*H*-pyrimido[4,5-*b*]indole (17i)

Arabinoside **16** (120 mg, 0.32 mmol) was reacted with phenylboronic acid (59 mg, 0.48 mmol) in 3 mL of H_2O/MeCN (2:1) for 4 h according to the general procedure A. Recrystallization from H_2O/MeOH mixture gave **17i** (76 mg, 58%) as a tan solid: m.p. 183–185 °C; $[\alpha]_D^{20}$ –6.5 (c 0.17, DMSO); ^1H NMR (500.0 MHz, DMSO-d_6): 3.78–3.88 (m, 3H, H-4′,5′); 4.18 (td, 1H, $J_{3',4'} = J_{3',OH} = 4.8$, $J_{3',2'} = 3.2$, H-3′); 4.26 (td, 1H, $J_{2',1'} = J_{2',OH} = 4.8$, $J_{2',3'} = 3.2$, H-2′); 5.13 (t, 1H, $J_{OH,5'} = 5.3$, OH-5′); 5.36 (d, 1H, $J_{OH,2'} = 4.8$, OH-2′); 5.59 (d, 1H, $J_{OH,3'} = 4.8$, OH-3′); 6.90 (d, 1H, $J_{1',2'} = 4.8$, H-1′); 7.50 (dd, 1H, $J_{7,8} = 9.0$, $J_{7,5} = 2.2$, H-7); 7.64 (d, 1H, $J_{5,7} = 2.2$, H-5); 7.67–7.72 (m, 3H, H-*m,p*-Ph); 7.88 (m, 2H, H-*o*-Ph); 8.13 (d, 1H, $J_{8,7} = 9.0$, H-8); 9.08 (s, 1H, H-2); ^{13}C NMR (125.7 MHz, DMSO-d_6): 61.10 (CH_2-5′); 76.67 (CH-3′); 77.68 (CH-2′); 84.10 (CH-4′); 85.66 (CH-1′); 110.16 (C-4a); 117.61 (CH-8); 120.30 (C-4b); 120.50 (CH-5); 125.44 (C-6); 127.21 (CH-7); 128.91 (CH-*o*-Ph); 129.09 (CH-*m*-Ph); 130.62 (CH-*p*-Ph); 137.83 (C-*i*-Ph); 138.39 (C-8a); 154.64 (CH-2); 155.66 (C-9a); 159.88 (C-4); IR (ATR): ν = 3365, 1561, 1448, 1219, 1174, 1058, 918, 883, 767, 702, 640, 601, 483 cm^{-1}; ESI MS m/z (rel. %): 412 (100) $[M+H]^+$, 434 (59) $[M+Na]^+$; HR MS (ESI) for $C_{21}H_{19}O_4N_3Cl$ $[M+H]^+$: calcd 412.10586; found 412.10594.

4,6-Dichloro-9-[2-*O*-acetyl-3,5-*O*-(tetraisopropyldisiloxan-1,3-diyl)-*β*-D-arabinofuranosyl]-9*H*-pyrimido[4,5-*b*]indole (18)

Protected arabinonucleoside **15** (3.49 g, 5.7 mmol) was dissolved in anhydrous MeCN (100 mL) and Et_3N (0.95 mL, 6.8 mmol), DMAP (70 mg, 0.57 mmol) and acetic anhydride (0.65 mL, 6.8 mmol) were added. The mixture was stirred at r.t. for 1 h and then evaporated under reduced pressure. Residue was dissolved in EtOAc (60 mL), extracted with water (50 mL) and saturated $NaHCO_3$ (30 mL), dried over $MgSO_4$ and evaporated under reduced pressure to give crude acetate **18** (3.62 g, 96%) as a yellowish foam: ^1H NMR (500.0 MHz, $CDCl_3$): 0.98–1.28 (m, 28H, $(CH_3)_2CHSi$); 1.32 (s, 3H, CH_3CO); 3. 90 (ddd, 1H, $J_{4',3'} = 8.5$, $J_{4',5'} = 3.8$, 3.2, H-4′); 4.14 (dd, 1H, $J_{gem} = 12.7$, $J_{5'b,4'} = 3.2$, H-5′b); 4.27 (dd, 1H, $J_{gem} = 12.7$, $J_{5'a,4'} = 3.8$, H-5′a); 5.11 (bdd, 1H, $J_{3',4'} = 8.5$, $J_{3',2'} = 6.5$, H-3′); 5.56 (dd, 1H, $J_{2',1'} = 6.9$, $J_{2',3'} = 6.5$, H-2′); 7.00 (d, 1H, $J_{1',2'} = 6.9$, H-1′); 7.51 (dd, 1H, $J_{7,8} = 8.9$, $J_{7,5} = 2.2$, H-7); 7.79 (d, 1H, $J_{8,7} = 8.9$, H-8); 8.33 (d, 1H, $J_{5,7} = 2.2$, H-5); 8.80 (s, 1H, H-2); ^{13}C NMR (125.7 MHz, $CDCl_3$): 12.44, 13.03, 13.13, 13.33 ($(CH_3)_2$**C**HSi); 16.80,

16.84, 16.91, 16.96, 17.36, 17.43, 17.52, 17.54 ((**C**H_3)$_2$CHSi); 19.56 (**C**H_3CO); 60.85 (CH_2-5′); 74.09 (CH-3′); 79.19 (CH-2′); 80.02 (CH-4′); 81.74 (CH-1′); 111.57 (C-4a); 114.54 (CH-8); 119.80 (C-4b); 122.42 (CH-5); 128.29 (C-6); 128.51 (CH-7); 137.09 (C-8a); 153.02 (C-4); 154.11 (CH-2); 155.89 (C-9a); 169.49 (**C**OCH_3); ESI MS m/z (rel. %): 676 (100) $[M+Na]^+$; HR MS (ESI) for $C_{29}H_{41}O_6N_3Cl_2NaSi_2$ $[M+Na]^+$: calcd 676.18032; found 676.1804215.

4,6-Dichloro-9-(2-*O*-acetyl-*β*-D-arabinofuranosyl)-9*H*-pyrimido[4,5-*b*]indole (19)

A solution of acetate **18** (6.2 g, 9.5 mmol) in anhydrous THF (100 mL) was treated with $Et_3N·3HF$ (3.1 mL, 20 mmol). The mixture was stirred overnight at r.t. Evaporation under reduced pressure and HPFC purification (silica column, 0→5% MeOH in DCM) gave **19** (3.7 g, 94%) as a yellowish foam: ^{1}H NMR (500.0 MHz, DMSO-d_6): 1.25 (s, 3H, CH_3CO); 3.79 (dd, 1H, J_{gem} = 12.0, $J_{5'b,4'}$ = 5.1, H-5′b); 3.84 (dd, 1H, J_{gem} = 12.0, $J_{5'a,4'}$ = 3.5, H-5′a); 3.91 (ddd, 1H, $J_{4',3'}$ = 6.4, $J_{4',5'}$ = 5.1, 3.5, H-4′); 4.48 (bdd, 1H, $J_{3',4'}$ = 6.4, $J_{3',2'}$ = 3.8, H-3′); 5.51 (bs, 1H, OH-5′); 5.27 (dd, 1H, $J_{2',1'}$ = 5.4, $J_{2',3'}$ = 3.8, H-2′); 5.92 (bs, 1H , OH-3′); 7.02 (d, 1H, $J_{1',2'}$ = 5.4, H-1′); 7.70 (dd, 1H, $J_{7,8}$ = 8.9, $J_{7,5}$ = 2.2, H-7); 8.10 (d, 1H, $J_{8,7}$ = 8.9, H-8); 8.27 (d, 1H, $J_{5,7}$ = 2.2, H-5); 8.92 (s, 1H, H-2); ^{13}C NMR (125.7 MHz, DMSO-d_6): 19.71 (**C**H_3CO); 60.25 (CH_2-5′); 73.58 (CH-3′); 79.91 (CH-2′); 82.94 (CH-1′); 83.29 (CH-4′); 110.77 (C-4a); 116.88 (CH-8); 119.31 (C-4b); 121.37 (CH-5); 127.16 (C-6); 128.48 (CH-7); 137.16 (C-8a); 152.27 (C-4); 154.71 (CH-2); 155.54 (C-9a); 169.22 (**C**OCH_3); IR (ATR): ν = 2945, 2867, 1750, 1580, 1546, 1460, 1437, 1219, 1153, 1098, 1032, 884, 835, 793, 693 cm^{-1}; ESI MS m/z (rel. %): 434 (100) $[M+Na]^+$; HR MS (ESI) for $C_{17}H_{15}O_5N_3Cl_2Na$ $[M+Na]^+$: calcd 434.02810; found 434.02816.

4,6-Dichloro-9-[2-*O*-acetyl-3,5-di-*O*-(tetrahydropyran-2-yl)-*β*-D-arabinofuranosyl]-9*H*-pyrimido[4,5-*b*]indole (20)

Compound **19** (1.83 g, 4.4 mmol) and $TsOH·H_2O$ (840 mg, 8.8 mmol) were dissolved in anhydrous DMF (50 mL) and the mixture was cooled to 0 °C. 3,4-Dihydro-2*H*-pyran (12.2 mL, 134 mmol) was added and the mixture was allowed to warm to r.t., stirred overnight, diluted with EtOAc (100 mL) and extracted with aqueous $NaHCO_3$ (saturated, 50 mL). The organic layer was dried over $MgSO_4$ and evaporated under reduced pressure. HPFC purification (silica column, 10→50% EtOAc in PE) furnished the crude product **20** (2.43 g) as a yellow oil. This intermediate was used directly in the next step. ESI MS m/z (rel. %): 580 (14) $[M+H]^+$, 602 (100) $[M+Na]^+$; HR MS (ESI) for $C_{27}H_{31}O_7N_3Cl_2Na$ $[M+Na]^+$: calcd 602.14313; found 602.14319.

4,6-Dichloro-9-[3,5-di-*O*-(tetrahydropyran-2-yl)-*β*-D-arabinofuranosyl]-9*H*-pyrimido[4,5-*b*]indole (21)

Crude THP-protected acetate **20** (2.4 g) was dissolved in methanolic ammonia (27%, 200 mL) at 0 °C and stirred for 4 h at 0 °C. The evaporation of the solution under reduced pressure resulted in crude THP-protected arabinoside **21** (2.1 g) as a yellow oil. This intermediate was used directly in the next step. ESI MS m/z (rel. %): 538 (14) $[M+H]^+$, 560 (100) $[M+Na]^+$; HR MS (ESI) for $C_{25}H_{29}O_6N_3Cl_2Na$ $[M+Na]^+$: calcd 560.13256; found 560.13257.

4,6-Dichloro-9-(2-deoxy-2-fluoro-*β*-D-ribofuranosyl)-9*H*-pyrimido[4,5-*b*]indole (22)

THP-protected arabinonucleoside **21** (2.1 g, 3.9 mmol) was dissolved in anhydrous DCM (55 mL) and anhydrous pyridine was added (2.4 mL, 9.75 mL). The mixture was cooled to 0 °C and treated with DAST (2.6 mL; 19.7 mmol). The mixture was allowed to warm to r.t. and stirred overnight. Then, it was diluted with DCM (60 mL) and neutralized with aqueous $NaHCO_3$ (saturated, 90 mL). The organic phase was washed with water (90 mL), dried over $MgSO_4$ and evaporated under reduced pressure. The crude product was then dissolved in aqueous TFA (90% *v*/*v*, 20 mL) and stirred at r.t. for 2 h. The solution was evaporated under reduced pressure and co-evaporated with MeOH. RP-HPFC purification (C18 column, 10→100% MeOH in H_2O) and recrystallization (H_2O/MeOH 3:1) gave the free fluororibonucleoside **22** (640 mg, 38% over four steps) as a beige solid: m.p. 249–251 °C; $[\alpha]_D^{20}$ –26.2 (c 0.24, DMSO); ^1H NMR (500.0 MHz, DMSO-d_6): 3.61 (dd, 1H, J_{gem} = 12.3, $J_{5'b,4'}$ = 4.5, H-5'b); 3.74 (dd, 1H, J_{gem} = 12.3, $J_{5'a,4'}$ = 2.6, H-5'a); 4.02 (dddd, 1H, $J_{4',3'}$ = 6.3, $J_{4',5'}$ = 4.5, 2.6, $J_{H,F}$ = 1.0, H-4'); 4.63 (ddd, 1H, $J_{H,F}$ = 14.8, $J_{3',4'}$ = 6.3, $J_{3',2'}$ = 5.4, H-3'); 5.71 (ddd, 1H, $J_{H,F}$ = 53.6, $J_{2',3'}$ = 5.4, $J_{2',1'}$ = 3.9, H-2'); 6.72 (dd, 1H, $J_{H,F}$ = 19.3, $J_{1',2'}$ = 3.9, H-1'); 7.71 (dd, 1H, $J_{7,8}$ = 8.9, $J_{7,5}$ = 2.1, H-7); 8.18 (d, 1H, $J_{8,7}$ = 8.9, H-8); 8.31 (d, 1H, $J_{5,7}$ = 2.1, H-5); 8.91 (s, 1H, H-2); ^{13}C NMR (125.7 MHz, DMSO-d_6): 60.78 (CH_2-5'); 68.45 (d, $J_{C,F}$ = 15.7, CH-3'); 84.20 (CH-4'); 86.02 (d, $J_{C,F}$ = 34.0, CH-1'); 91.75 (d, $J_{C,F}$ = 187.0, CH-2'); 111.41 (C-4a); 114.60 (CH-8); 119.53 (C-4b); 121.85 (CH-5); 127.55 (C-6); 128.92 (CH-7); 136.78 (C-8a); 152.54 (C-4); 154.70 (CH-2); 155.67 (C-9a); ^{19}F NMR (470.4 MHz, DMSO-d_6): –198.47 (ddd, $J_{F,H}$ = 53.6, 19.3, 14.8); IR (ATR): ν = 3279, 1589, 1443, 1295, 1227, 1104, 1057, 1028, 1003, 833, 808, 537 cm^{-1}; ESI MS m/z (rel. %): 372 (100) $[M+H]^+$; HR MS (ESI) for $C_{15}H_{13}O_3N_3ClF$ $[M+H]^+$: calcd 372.03125; found 372.03137.

4-Amino-6-chloro-9-(2-deoxy-2-fluoro-*β*-D-ribofuranosyl)-9*H*-pyrimido[4,5-*b*]indole (23a)

Fluororiboside **22** (90 mg, 0.24 mmol) was dissolved in dioxane (3 mL) and aqueous ammonia (30%, 3 mL) was added. The mixture was stirred in screw-cap pressure glass tube at 100 °C for

20 h and then solvents were evaporated under reduced pressure. RP-HPFC (C18 column, 10→100% MeOH in H_2O) and recrystallization from a H_2O/MeOH mixture gave nucleoside **23a** (69 mg, 82%) as a white solid: m.p. 275–278 °C; $[\alpha]_D^{20}$ –44.9 (c 0.21, DMSO); ^{1}H NMR (500.0 MHz, DMSO-d_6): 3.59 (ddd, 1H, J_{gem} = 12.2, $J_{5'b,OH}$ = 5.9, $J_{5'b,4'}$ = 4.5, H-5'b); 3.72 (ddd, 1H, J_{gem} = 12.2, $J_{5'a,OH}$ = 5.2, $J_{5'a,4'}$ = 2.6, H-5'a); 3.97 (dddd, 1H, $J_{4',3'}$ = 5.8, $J_{4',5'}$ = 4.5, 2.6, $J_{H,F}$ = 1.2, H-4'); 4.56 (dddd, 1H, $J_{H,F}$ = 13.0, $J_{3',OH}$ = 6.2, $J_{3',4'}$ = 5.8, $J_{3',2'}$ = 5.5, H-3'); 5.16 (dd, 1H, $J_{OH,5'}$ = 5.9, 5.2, OH-5′); 5.65 (ddd, 1H, $J_{H,F}$ = 54.1, $J_{2',3'}$ = 5.5, $J_{2',1'}$ = 4.4, H-2'); 5.69 (d, 1H, $J_{OH,3'}$ = 6.2, OH-3′); 6.60 (dd, 1H, $J_{H,F}$ = 19.3, $J_{1',2'}$ = 4.4, H-1'); 7.41 (dd, 1H, $J_{7,8}$ = 8.8, $J_{7,5}$ = 2.1, H-7); 7.51 (bs, 2H, NH_2); 7.90 (d, 1H, $J_{8,7}$ = 8.8, H-8); 8.31 (s, 1H, H-2); 8.51 (d, 1H, $J_{5,7}$ = 2.1, H-5); ^{13}C NMR (125.7 MHz, DMSO-d_6): 61.18 (CH_2-5'); 68.61 (d, $J_{C,F}$ = 15.5, CH-3'); 84.09 (CH-4'); 85.45 (d, $J_{C,F}$ = 33.3, CH-1'); 91.45 (d, $J_{C,F}$ = 186.9, CH-2'); 95.37 (C-4a); 112.85 (CH-8); 120.93 (CH-5); 121.28 (C-4b); 124.88 (CH-7); 126.48 (C-6); 134.57 (C-8a); 155.50 (CH-2); 155.67 (C-9a); 157.99 (C-4); ^{19}F NMR (470.4 MHz, DMSO-d_6): –199.28 (ddd, $J_{F,H}$ = 54.1, 19.3, 13.0); IR (ATR): ν = 3163, 1575, 1463, 1303, 1198, 1059, 901, 857, 799, 775, 425 cm^{-1}; ESI MS m/z (rel. %): 353 (100) $[M+H]^+$, 375 (31) $[M+Na]^+$; HR MS (ESI) for $C_{15}H_{15}O_3N_4ClF$ $[M+H]^+$: calcd 353.08112; found 353.08124.

6-Chloro-9-(2-deoxy-2-fluoro-*β*-D-ribofuranosyl)-4-methoxy-9*H*-pyrimido[4,5-*b*]indole (23b)

Nucleoside **22** (90 mg, 0.24 mmol) was dissolved in dry MeOH (5 mL) and sodium methoxide (1M in MeOH, 2 mL) was added. The mixture was stirred for 3 h at r.t. Solvent was evaporated and crude product was purified by RP-HPFC (C18 column, 10→100% MeOH in H_2O) and recrystallized from a H_2O/MeOH mixture. Nucleoside **23b** (71 mg, 80%) was obtained as a white solid: m.p. 201–205 °C; $[\alpha]_D^{20}$ –33.6 (c 0.22, DMSO); ^{1}H NMR (500.0 MHz, DMSO-d_6): 3.60 (ddd, 1H, J_{gem} = 12.2, $J_{5'b,OH}$ = 5.6, $J_{5'b,4'}$ = 4.6, H-5'b); 3.73 (dd, 1H, J_{gem} = 12.2, $J_{5'a,OH}$ = 5.3, $J_{5'a,4'}$ = 2.6, H-5'a); 4.00 (dddd, 1H, $J_{4',3'}$ = 6.3, $J_{4',5'}$ = 4.6, 2.6, $J_{H,F}$ = 1.0, H-4'); 4.20 (s, 3H, CH_3O); 4.60 (dtd, 1H, $J_{H,F}$ = 13.8, $J_{3',4'}$ = $J_{3',OH}$ = 6.3, $J_{3',2'}$ = 5.4, H-3'); 5.07 (dd, 1H, $J_{OH,5'}$ = 5.6, 5.3, OH-5′); 5.69 (ddd, 1H, $J_{H,F}$ = 53.6, $J_{2',3'}$ = 5.4, $J_{2',1'}$ = 4.2, H-2'); 5.73 (d, 1H, $J_{OH,3'}$ = 6.3, OH-3′); 6.67 (dd, 1H, $J_{H,F}$ = 19.2, $J_{1',2'}$ = 4.2, H-1'); 7.54 (dd, 1H, $J_{7,8}$ = 8.8, $J_{7,5}$ = 2.2, H-7); 8.00 (d, 1H, $J_{5,7}$ = 2.2, H-5); 8.05 (d, 1H, $J_{8,7}$ = 8.8, H-8); 8.71 (s, 1H, H-2); ^{13}C NMR (125.7 MHz, DMSO-d_6): 54.52 (CH_3O); 60.97 (CH_2-5'); 68.52 (d, $J_{C,F}$ = 15.6, CH-3'); 84.11 (CH-4'); 85.74 (d, $J_{C,F}$ = 33.5, CH-1'); 91.64 (d, $J_{C,F}$ = 187.1, CH-2'); 98.91 (C-4a); 113.96 (CH-8); 120.39 (C-4b); 121.45 (CH-5); 126.59 (CH-7); 126.86 (C-6); 135.39 (C-8a); 155.20 (CH-2); 156.39 (C-9a); 164.06 (C-4); ^{19}F NMR (470.4 MHz, DMSO-d_6): –199.04 (ddd, $J_{F,H}$ = 53.6, 19.2, 13.8); IR (ATR): ν = 3313, 1600, 1460,

1327, 1189, 1109, 1069, 1045, 894, 851, 536, 432 cm^{-1}; ESI MS m/z (rel. %): 368 (79) $[M+H]^+$, 390 (100) $[M+Na]^+$; HR MS (ESI) for $C_{16}H_{15}O_4N_3ClFNa$ $[M+Na]^+$: calcd 390.06273; found 390.06282.

6-Chloro-9-(2-deoxy-2-fluoro-*β*-D-ribofuranosyl)-4-methylsulfanyl-9*H*-pyrimido[4,5-*b*]indole (23c)

Nucleoside **22** (90 mg, 0.24 mmol) and sodium methanethiolate (35 mg, 0.50 mmol) were dissolved in anhydrous EtOH (10 mL) and stirred for 2 h at r.t. The solvent was evaporated under reduced pressure. RP-HPFC (C18 column, 10→100% MeOH in H_2O) and recrystallization from H_2O/MeOH mixture gave compound **23c** (80 mg, 87%) as a white solid: m.p. 205–209 °C; $[\alpha]_D^{20}$ –34.6 (c 0.19, DMSO); ^{1}H NMR (500.0 MHz, DMSO-d_6): 2.80 (s, 3H, CH_3S); 3.59 (ddd, 1H, J_{gem} = 12.2, $J_{5'b,OH}$ = 5.5, $J_{5'b,4'}$ = 4.6, H-5'b); 3.73 (dd, 1H, J_{gem} = 12.2, $J_{5'a,OH}$ = 5.3, $J_{5'a,4'}$ = 2.6, H-5'a); 4.00 (dddd, 1H, $J_{4',3'}$ = 6.2, $J_{4',5'}$ = 4.6, 2.6, $J_{H,F}$ = 1.3, H-4'); 4.62 (dtd, 1H, $J_{H,F}$ = 13.8, $J_{3',4'}$ = $J_{3',OH}$ = 6.2, $J_{3',2'}$ = 5.4, H-3'); 5.07 (dd, 1H, $J_{OH,5'}$ = 5.5, 5.3, OH-5′); 5.69 (ddd, 1H, $J_{H,F}$ = 53.8, $J_{2',3'}$ = 5.4, $J_{2',1'}$ = 4.0, H-2'); 5.75 (d, 1H, $J_{OH,3'}$ = 6.2, OH-3′); 6.69 (dd, 1H, $J_{H,F}$ = 19.5, $J_{1',2'}$ = 4.0, H-1'); 7.62 (dd, 1H, $J_{7,8}$ = 8.8, $J_{7,5}$ = 2.1, H-7); 8.04 (d, 1H, $J_{5,7}$ = 2.1, H-5); 8.11 (d, 1H, $J_{8,7}$ = 8.8, H-8); 8.88 (s, 1H, H-2); ^{13}C NMR (125.7 MHz, DMSO-d_6): 11.92 (CH_3S); 60.91 (CH_2-5'); 68.50 (d, $J_{C,F}$ = 15.6, CH-3'); 84.08 (CH-4'); 85.74 (d, $J_{C,F}$ = 33.8, CH-1'); 91.75 (d, $J_{C,F}$ = 186.8, CH-2'); 109.74 (C-4a); 114.15 (CH-8); 120.51 (C-4b); 121.71 (CH-5); 126.94 (C-6); 127.32 (CH-7); 135.84 (C-8a); 153.24 (C-9a); 154.27 (CH-2); 163.16 (C-4); ^{19}F NMR (470.4 MHz, DMSO-d_6): –198.57 (ddd, $J_{F,H}$ = 53.8, 19.5, 13.8); IR (ATR): ν = 3247, 1558, 1472, 1433, 1295, 1235, 1049, 966, 906, 839, 794, 594, 535 cm^{-1}; ESI MS m/z (rel. %): 384 (81) $[M+H]^+$, 406 (100) $[M+Na]^+$; HR MS (ESI) for $C_{16}H_{15}O_3N_3ClFNaS$ $[M+Na]^+$: calcd 406.03989; found 406.03999.

6-Chloro-9-(2-deoxy-2-fluoro-*β*-D-ribofuranosyl)-4-methyl-9*H*-pyrimido[4,5-*b*]indole (23d)

Nucleoside **22** (90 mg, 0.24 mmol) and $Pd(PPh_3)_4$ (14 mg, 0.012 mmol) were dissolved in anhydrous THF (4 mL) and Me_3Al (2M in toluene, 250 µL) was added. The mixture was stirred at 70 °C for 24 h. Solvents evaporation under reduced pressure, RP-HPFC purification (C18 column, 10→100% MeOH in H_2O) and recrystallization from a H_2O/MeOH mixture gave compound **23d** (55 mg, 65%) as a white solid: m.p. 247–251 °C; $[\alpha]_D^{20}$ –45.2 (c 0.18, DMSO); ^{1}H NMR (500.0 MHz, DMSO-d_6): 2.95 (s, 3H, CH_3); 3.59 (ddd, 1H, J_{gem} = 12.2, $J_{5'b,OH}$ = 5.5, $J_{5'b,4'}$ = 4.7, H-5'b); 3.73 (dd, 1H, J_{gem} = 12.2, $J_{5'a,OH}$ = 5.3, $J_{5'a,4'}$ = 2.6, H-5'a); 4.00 (dddd, 1H, $J_{4',3'}$ = 6.2, $J_{4',5'}$ = 4.7, 2.6, $J_{H,F}$ = 1.0, H-4'); 4.62 (dtd, 1H, $J_{H,F}$ = 14.0, $J_{3',4'}$ = $J_{3',OH}$ = 6.2, $J_{3',2'}$ = 5.4, H-3'); 5.08 (dd, 1H, $J_{OH,5'}$ = 5.5, 5.3, OH-5′); 5.71 (ddd, 1H, $J_{H,F}$ = 53.9, $J_{2',3'}$ = 5.4, $J_{2',1'}$ = 4.1, H-2'); 5.75

(d, 1H, $J_{OH,3'}$ = 6.2, OH-3′); 6.70 (dd, 1H, $J_{H,F}$ = 19.4, $J_{1',2'}$ = 4.1, H-1'); 7.62 (dd, 1H, $J_{7,8}$ = 8.8, $J_{7,5}$ = 2.1, H-7); 8.09 (d, 1H, $J_{8,7}$ = 8.8, H-8); 8.23 (d, 1H, $J_{5,7}$ = 2.1, H-5); 8.89 (s, 1H, H-2); ^{13}C NMR (125.7 MHz, DMSO-d_6): 23.03 (CH_3); 60.97 (CH_2-5'); 68.53 (d, $J_{C,F}$ = 15.6, CH-3'); 84.07 (CH-4'); 85.54 (d, $J_{C,F}$ = 33.6, CH-1'); 91.57 (d, $J_{C,F}$ = 186.9, CH-2'); 111.84 (C-4a); 113.92 (CH-8); 121.27 (C-4b); 122.59 (CH-5); 126.94 (C-6); 127.56 (CH-7); 136.30 (C-8a); 154.60 (CH-2); 154.73 (C-9a); 161.33 (C-4); IR (ATR): ν = 3309, 1580, 1449, 1371, 1299, 1183, 1133, 1103, 1068, 1024, 859, 828, 808, 621, 525, 480, 425, 389 cm^{-1}; ^{19}F NMR (470.4 MHz, DMSO-d_6): –198.88 (ddd, $J_{F,H}$ = 53.9, 19.4, 14.0); ESI MS m/z (rel. %): 352 (100) [M+H]$^+$, 374 (60) [M+Na]$^+$; HR MS (ESI) for $C_{16}H_{16}O_3N_3ClF$ [M+H]$^+$: calcd 352.08587; found 352.08597.

6-Chloro-9-(2-deoxy-2-fluoro-*β*-D-ribofuranosyl)-4-(furan-2-yl)-9*H*-pyrimido-[4,5-*b*]indole (23e)

Nucleoside **22** (130 mg, 0.35 mmol) was reacted with furan-2-boronic acid (59 mg, 0.53 mmol) in 4 mL of H_2O/MeCN (2:1) for 2 h according to the general procedure A. Recrystallization from H_2O/MeOH mixture gave nucleoside **23e** (17 mg, 12%) as a white solid: m.p. 203–206 °C; $[\alpha]_D^{20}$ –48.9 (c 0.14, DMSO); ^{1}H NMR (500.0 MHz, DMSO-d_6): 3.62 (dt, 1H, J_{gem} = 12.3 Hz, $J_{5'a,OH}$ = $J_{5'a,4'}$ = 5.0 Hz, H-5′a); 3.75 (ddd, 1H, J_{gem} = 12.3 Hz, $J_{5'b,OH}$ = 5.1 Hz, $J_{5'b,4'}$ = 2.6 Hz, H-5′b); 4.01 (dddd, 1H, $J_{4',3'}$ = 6.4, $J_{4',5'a}$ = 4.7 Hz, $J_{4',5'b}$ = 2.6 Hz, $J_{4',F}$ = 1.1 Hz, H-4′); 4.64 (dtd, 1H, $J_{3',F}$ = 14.3 Hz, $J_{3',4'}$ = $J_{3',OH}$ = 6.3 Hz, $J_{3',2'}$ = 5.5 Hz, H-3′); 5.09 (t, 1H, $J_{OH,5'}$ = 5.4 Hz, OH-5′); 5.73 (ddd, 1H, $J_{2',F}$ = 53.9 Hz, $J_{2',3'}$ = 5.5 Hz, $J_{2',1'}$ = 4.0 Hz, H-2′); 5.76 (bd, 1H, $J_{OH,3'}$ = 6.2 Hz, OH-3′); 6.76 (dd, 1H, $J_{1',F}$ = 19.8 Hz, $J_{1',2'}$ = 4.0 Hz, H-1′); 6.91 (dd, 1H, $J_{4,3}$ = 3.5 Hz, $J_{4,5}$ = 1.8 Hz, H-4-furyl); 7.62 (dd, 1H, $J_{3,4}$ = 3.5 Hz, $J_{3,5}$ = 0.9 Hz, H-3-furyl); 7.65 (dd, 1H, $J_{7,8}$ = 8.9 Hz, $J_{7,5}$ = 2.2 Hz, H-7); 8.11 (d, 1H, $J_{8,7}$ = 8.9 Hz, H-8); 8.36 (dd, 1H, $J_{5,4}$ = 1.8 Hz, $J_{5,3}$ = 0.9 Hz, H-5-furyl); 8.82 (d, 1H, $J_{5,7}$ = 2.2 Hz, H-5); 8.98 (s, 1H, H-2); ^{13}C NMR (125.7 MHz, DMSO-d_6): 60.95 (CH_2-5′); 68.51 (d, $J_{C,F}$ = 15.6 Hz, CH-3′); 84.08 (CH-4′); 85.71 (d, $J_{C,F}$ = 34.2 Hz, CH-1′); 91.58 (d, $J_{C,F}$ = 186.4 Hz, CH-2′); 107.31 (C-4a); 113.41 (CH-4-furyl); 113.80 (CH-8); 115.73 (CH-3-furyl); 120.59 (C-4b); 124.01 (CH-5); 126.99 (C-6); 128.16 (CH-7); 136.93 (C-8a); 147.16 (CH-5-furyl); 148.18 (C-4); 152.30 (C-2-furyl); 154.49 (CH-2); 156.56 (C-9a); ^{19}F NMR (470.4 MHz, DMSO-d_6): –198.06 (ddd, 1F, $J_{F,2'}$ = 53.9 Hz, $J_{F,1'}$ = 19.8 Hz, $J_{F,3'}$ = 14.3 Hz, F-2′); IR (ATR): ν = 3313, 2936, 1571, 1544, 1466, 1441, 1048, 802, 750, 595, 537 cm^{-1}; ESI MS m/z (rel. %): 426 (100) [M+Na]$^+$, 404 (27) [M+H]$^+$; HR MS (ESI) for $C_{19}H_{15}O_4N_3ClFNa$ [M+Na]$^+$: calcd 426.06273; found 426.06275.

6-Chloro-9-(2-deoxy-2-fluoro-*β*-D-ribofuranosyl)-4-(furan-3-yl)-9*H*-pyrimido-[4,5-*b*]indole (23f)

Nucleoside **22** (130 mg, 0.35 mmol) was reacted with furan-3-boronic acid (59 mg, 0.53 mmol) in 4 mL of H_2O/MeCN (2:1) for 2 h according to the general procedure A. Recrystallization from H_2O/MeOH mixture gave nucleoside **23f** (70 mg, 50%) as a white solid: m.p. 184–186 °C; $[\alpha]_D^{20}$ –33.5 (c 0.21, DMSO); ^{1}H NMR (500.0 MHz, DMSO-d_6): 3.62 (ddd, 1H, J_{gem} = 12.3 Hz, $J_{5'a,OH}$ = 5.5 Hz, $J_{5'a,4'}$ = 4.5 Hz, H-5′a); 3.75 (ddd, 1H, J_{gem} = 12.3 Hz, $J_{5'b,OH}$ = 5.4 Hz, $J_{5'b,4'}$ = 2.6 Hz, H-5′b); 4.02 (dddd, 1H, $J_{4',3'}$ = 6.2, $J_{4',5'a}$ = 4.5 Hz, $J_{4',5'b}$ = 2.6 Hz, $J_{4',F}$ = 1.2 Hz, H-4′); 4.64 (dtd, 1H, $J_{3',F}$ = 14.0 Hz, $J_{3',4'}$ = $J_{3',OH}$ = 6.2 Hz, $J_{3',2'}$ = 5.2 Hz, H-3′); 5.09 (t, 1H, $J_{OH,5'}$ = 5.4 Hz, OH-5′); 5.74 (ddd, 1H, $J_{2',F}$ = 53.8 Hz, $J_{2',3'}$ = 5.5 Hz, $J_{2',1'}$ = 4.1 Hz, H-2′); 5.77 (bd, 1H, $J_{OH,3'}$ = 6.2 Hz, OH-3′); 6.75 (dd, 1H, $J_{1',F}$ = 19.4 Hz, $J_{1',2'}$ = 4.1 Hz, H-1′); 7.12 (dd, 1H, $J_{4,5}$ = 1.9 Hz, $J_{4,2}$ = 0.9 Hz, H-4-furyl); 7.63 (dd, 1H, $J_{7,8}$ = 8.9 Hz, $J_{7,5}$ = 2.1 Hz, H-7); 8.02 (t, 1H, $J_{5,2}$ = $J_{5,4}$ = 1.7 Hz, H-5-furyl); 8.10 (d, 1H, $J_{5,7}$ = 2.1 Hz, H-5); 8.12 (d, 1H, $J_{8,7}$ = 8.9 Hz, H-8); 8.55 (dd, 1H, $J_{2,5}$ = 1.6 Hz, $J_{2,4}$ = 0.9 Hz, H-2-furyl); 9.02 (s, 1H, H-2); ^{13}C NMR (125.7 MHz, DMSO-d_6): 60.97 (CH_2-5′); 68.54 (d, $J_{C,F}$ = 15.7 Hz, CH-3′); 84.15 (CH-4′); 85.65 (d, $J_{C,F}$ = 33.7 Hz, CH-1′); 91.59 (d, $J_{C,F}$ = 186.9 Hz, CH-2′); 110.48 (C-4a); 110.75 (CH-4-furyl); 114.16 (CH-8); 120.63 (C-4b); 121.82 (CH-5); 124.32 (C-3-furyl); 126.71 (C-6); 128.10 (CH-7); 136.66 (C-8a); 144.66 (CH-2-furyl); 145.01 (CH-5-furyl); 153.43 (C-4); 154.76 (CH-2); 155.83 (C-9a); ^{19}F NMR (470.4 MHz, DMSO-d_6): –198.88 (bdt, 1F, $J_{F,2'}$ = 53.8 Hz, $J_{F,1'}$ = $J_{F,3'}$ = 16.6 Hz, F-2′); IR (ATR): ν = 3149, 1570, 1471, 1440, 1293, 1109, 1070, 805, 599, 539 cm^{-1}; ESI MS m/z (rel. %): 426 (100) $[M+Na]^+$, 404 (60) $[M+H]^+$; HR MS (ESI) for $C_{19}H_{16}O_4N_3ClF$ $[M+H]^+$: calcd 404.08079; found 404.08090.

6-Chloro-9-(2-deoxy-2-fluoro-*β*-D-ribofuranosyl)-4-(thiophen-2-yl)-9*H*-pyrimido-[4,5-*b*]indole (23g)

Nucleoside **22** (90 mg, 0.24 mmol) was reacted with thiophene-2-boronic acid (46 mg, 0.36 mmol) in 3 mL of H_2O/MeCN (2:1) for 4 h according to the general procedure A. Recrystallization from H_2O/MeOH mixture furnished nucleoside **23g** (94 mg, 70%) as a tan solid: m.p. 206–211 °C; $[\alpha]_D^{20}$ –37.1 (c 0.19, DMSO); ^{1}H NMR (500.0 MHz, DMSO-d_6): 3.62 (ddd, 1H, J_{gem} = 12.2, $J_{5'b,OH}$ = 5.5, $J_{5'b,4'}$ = 4.6, H-5'b); 3.75 (ddd, 1H, J_{gem} = 12.2, $J_{5'a,OH}$ = 5.3, $J_{5'a,4'}$ = 2.6, H-5'a); 4.02 (dddd, 1H, $J_{4',3'}$ = 6.3, $J_{4',5'}$ = 4.6, 2.6, $J_{H,F}$ = 1.0, H-4'); 4.65 (dtd, 1H, $J_{H,F}$ = 14.4, $J_{3',4'}$ = $J_{3',OH}$ = 6.3, $J_{3',2'}$ = 5.4, H-3'); 5.09 (dd, 1H, $J_{OH,5'}$ = 5.5, 5.3, OH-5′); 5.74 (ddd, 1H, $J_{H,F}$ = 53.8, $J_{2',3'}$ = 5.4, $J_{2',1'}$ = 4.0, H-2'); 5.78 (d, 1H, $J_{OH,3'}$ = 6.3, OH-3′); 6.77 (dd, 1H, $J_{H,F}$ = 19.7, $J_{1',2'}$ = 4.0, H-1'); 7.43 (dd, 1H, $J_{4,5}$ = 5.0, $J_{4,3}$ = 3.7, H-4-thienyl); 7.65 (dd, 1H, $J_{7,8}$ = 8.8, $J_{7,5}$ = 2.1, H-7); 8.01 (dd, 1H, $J_{5,4}$ = 5.0, $J_{5,3}$ = 1.1, H-5-thienyl); 8.09 (dd, 1H, $J_{3,4}$ = 3.7, $J_{3,5}$ = 1.1, H-3-thienyl); 8.15 (d, 1H, $J_{8,7}$ = 8.8,

H-8); 8.25 (d, 1H, $J_{5,7}$ = 2.1, H-5); 9.00 (s, 1H, H-2); ^{13}C NMR (125.7 MHz, DMSO-d_6): 60.95 (CH_2-5'); 68.52 (d, $J_{C,F}$ = 15.6, CH-3'); 84.11 (CH-4'); 85.75 (d, $J_{C,F}$ = 34.0, CH-1'); 91.67 (d, $J_{C,F}$ = 186.8, CH-2'); 109.17 (C-4a); 114.26 (CH-8); 120.52 (C-4b); 121.55 (CH-5); 126.67 (C-6); 128.30 (CH-7); 128.80 (CH-4-thienyl); 130.17 (CH-3-thienyl); 131.84 (CH-5-thienyl); 136.80 (C-8a); 140.94 (C-2-thienyl); 153.70 (C-4); 154.52 (CH-2); 156.24 (C-9a); ^{19}F NMR (470.4 MHz, DMSO-d_6): –198.28 (ddd, $J_{F,H}$ = 53.8, 19.7, 14.4); IR (ATR): ν = 3090, 1569, 1443, 1291, 1051, 965, 804, 717, 539, 436 cm^{-1}; ESI MS m/z (rel. %): 420 (100) [M+H]$^+$, 442 (79) [M+Na]$^+$; HR MS (ESI) for $C_{19}H_{16}O_3N_3ClFS$ [M+H]$^+$: calcd 420.05794; found 420.05797.

6-Chloro-9-(2-deoxy-2-fluoro-*β*-D-ribofuranosyl)-4-(thiophen-3-yl)-9*H*-pyrimido-[4,5-*b*]indole (23h)

Nucleoside **22** (130 mg, 0.35 mmol) was reacted with thiophene-3-boronic acid (67 mg, 0.52 mmol) in 4 mL of H_2O/MeCN (2:1) for 2 h according to the general procedure A. Recrystallization from H_2O/MeOH mixture gave nucleoside **23h** (58 mg, 40%) as a beige solid: m.p. 221–222 °C; $[\alpha]_D^{20}$ –32.7 (c 0.16, DMSO); ^{1}H NMR (500.0 MHz, DMSO-d_6): 3.62 (dd, 1H, J_{gem} = 12.2 Hz, $J_{5'a,4'}$ = 4.5 Hz, H-5′a); 3.75 (dd, 1H, J_{gem} = 12.2 Hz, $J_{5'b,4'}$ = 2.6 Hz, H-5′b); 4.02 (m, 1H, H-4′); 4.65 (m, 1H, H-3′); 5.08 (bs, 1H, OH-5′); 5.75 (ddd, 1H, $J_{2',F}$ = 53.9 Hz, $J_{2',3'}$ = 5.5 Hz, $J_{2',1'}$ = 4.1 Hz, H-2′); 5.76 (bd, 1H, $J_{OH,3'}$ = 5.7 Hz, OH-3′); 6.77 (dd, 1H, $J_{1',F}$ = 19.5 Hz, $J_{1',2'}$ = 4.1 Hz, H-1′); 7.62 (dd, 1H, $J_{7,8}$ = 8.9 Hz, $J_{7,5}$ = 2.1 Hz, H-7); 7.67 (dd, 1H, $J_{4,5}$ = 5.0 Hz, $J_{4,2}$ = 1.3 Hz, H-4-thienyl); 7.89 (dd, 1H, $J_{5,4}$ = 5.0 Hz, $J_{5,2}$ = 2.9 Hz, H-5-thienyl); 7.96 (d, 1H, $J_{5,7}$ = 2.1 Hz, H-5); 8.13 (d, 1H, $J_{8,7}$ = 8.9 Hz, H-8); 8.33 (dd, 1H, $J_{2,5}$ = 2.9 Hz, $J_{2,4}$ = 1.3 Hz, H-2-thienyl); 9.04 (s, 1H, H-2); ^{13}C NMR (125.7 MHz, DMSO-d_6): 60.95 (CH_2-5′); 68.51 (d, $J_{C,F}$ = 15.6 Hz, CH-3′); 84.11 (CH-4′); 85.65 (d, $J_{C,F}$ = 33.8 Hz, CH-1′); 91.59 (d, $J_{C,F}$ = 186.9 Hz, CH-2′); 110.40 (C-4a); 114.17 (CH-8); 120.70 (C-4b); 121.64 (CH-5); 126.54 (C-6); 128.00 (CH-5-thienyl); 128.08 (CH-7); 128.16 (CH-4-thienyl); 128.79 (CH-2-thienyl); 136.72 (C-8a); 139.06 (C-3-thienyl); 154.73 (CH-2); 155.83 and 155.92 (C-4, 9a); ^{19}F NMR (470.4 MHz, DMSO-d_6): –198.49 (ddd, 1F, $J_{F,2'}$ = 53.9 Hz, $J_{F,1'}$ = 19.3 Hz, $J_{F,3'}$ = 14.8 Hz, F-2′); IR (ATR): ν = 3068, 1570, 1445, 1291, 1093, 1050, 1028, 969, 888, 840, 688, 538, 497 cm^{-1}; ESI MS m/z (rel. %): 442 (100) [M+Na]$^+$, 420 (87) [M+H]$^+$; HR MS (ESI) for $C_{19}H_{15}O_3N_3ClFNaS$ [M+Na]$^+$: calcd 442.03989; found 442.03990.

6-Chloro-9-(2-deoxy-2-fluoro-*β*-D-ribofuranosyl)-4-phenyl-9*H*-pyrimido-[4,5-*b*]indole (23i)

Nucleoside **22** (130 mg, 0.35 mmol) was reacted with phenylboronic acid (59 mg, 0.48 mmol) in 4 mL of H_2O/MeCN (2:1) for 2 h according to the general procedure A. Recrystallization from

H_2O/MeOH mixture gave nucleoside **23i** (59 mg, 41%) as a tan solid: m.p. 192–193 °C; $[\alpha]_D^{20}$ –44.8 (c 0.20, DMSO); ^{1}H NMR (500.0 MHz, DMSO-d_6): 3.63 (ddd, 1H, J_{gem} = 12.2 Hz, $J_{5'a,OH}$ = 5.6 Hz, $J_{5'a,4'}$ = 4.6 Hz, H-5´a); 3.76 (ddd, 1H, J_{gem} = 12.2 Hz, $J_{5'b,OH}$ = 5.3 Hz, $J_{5'b,4'}$ = 2.6 Hz, H-5´b); 4.03 (dddd, 1H, $J_{4',3'}$ = 6.2, $J_{4',5'a}$ = 4.6 Hz, $J_{4',5'b}$ = 2.6 Hz, $J_{4',F}$ = 1.1 Hz, H-4´); 4.66 (dtd, 1H, $J_{3',F}$ = 14.2 Hz, $J_{3',4'}$ = $J_{3',OH}$ = 6.2 Hz, $J_{3',2'}$ = 5.5 Hz, H-3´); 5.10 (bt, 1H, $J_{OH,5'}$ = 5.4 Hz, OH-5´); 5.76 (ddd, 1H, $J_{2',F}$ = 53.9 Hz, $J_{2',3'}$ = 5.5 Hz, $J_{2',1'}$ = 4.1 Hz, H-2´); 5.78 (bd, 1H, $J_{OH,3'}$ = 6.2 Hz, OH-3´); 6.77 (dd, 1H, $J_{1',F}$ = 19.5 Hz, $J_{1',2'}$ = 4.1 Hz, H-1´); 7.61 (dd, 1H, $J_{7,8}$ = 8.9 Hz, $J_{7,5}$ = 2.2 Hz, H-7); 7.66–7.72 (m, 4H, H-*m*,*p*-Ph, H-5); 7.89 (m, 2H, H-*o*-Ph); 8.13 (d, 1H, $J_{8,7}$ = 8.9 Hz, H-8); 9.10 (s, 1H, H-2); ^{13}C NMR (125.7 MHz, DMSO-d_6): 60.98 (CH_2-5´); 68.56 (d, $J_{C,F}$ = 15.6 Hz, CH-3´); 84.15 (CH-4´); 85.72 (d, $J_{C,F}$ = 33.8 Hz, CH-1´); 91.65 (d, $J_{C,F}$ = 187.0 Hz, CH-2´); 110.66 (C-4a); 114.30 (CH-8); 120.67 (C-4b); 121.46 (CH-5); 126.50 (C-6); 128.19 (CH-7); 128.96 (CH-*o*-Ph); 129.16 (CH-*m*-Ph); 130.82 (CH-*p*-Ph); 136.83 (C-8a); 137.63 (C-*i*-Ph); 154.88 (CH-2); 155.86 (C-9a); 160.56 (C-4); ^{19}F NMR (470.4 MHz, DMSO-d_6): –198.45 (ddd, 1F, $J_{F,2'}$ = 53.9 Hz, $J_{F,1'}$ = 19.5 Hz, $J_{F,3'}$ = 14.2 Hz, F-2´); IR (ATR): ν = 3311, 1565, 1473, 1071, 1049, 814, 766, 705, 659, 605, 556, 515, 441 cm^{-1}; ESI MS m/z (rel. %): 436 (100) $[M+Na]^+$, 414 (68) $[M+H]^+$; HR MS (ESI) for $C_{21}H_{17}O_3N_3ClFNa$ $[M+Na]^+$: calcd 436.08347; found 436.08348.

HPLC Purity of Final Compounds

Supplementary Table S1. HPLC Purity of Final Compounds

Compound	t_r [min]	Purity [%]
**9a**	13.13	99.27
**9b**	15.17	98.63
**9c**	15.91	99.64
**9d**	13.90	99.87
**9e**	15.29	98.59
**9f**	14.81	96.36
**9g**	15.71	99.88
**9h**	15.32	99.59
**9i**	15.57	99.93
**16**	14.05	98.88
**17a**	12.52	96.09
**17b**	14.04	99.56
**17c**	14.60	100.0
**17d**	13.04	99.00
**17e**	14.16	97.03
**17f**	13.83	96.70
**17g**	14.54	98.80
**17h**	14.26	98.86
**17i**	14.45	97.94
**22**	15.31	95.59
**23a**	13.36	98.92
**23b**	15.48	95.06
**23c**	16.23	99.68
**23d**	14.04	99.78
**23e**	15.49	99.08
**23f**	14.99	99.43
**23g**	15.91	98.75
**23h**	15.51	95.86
**23i**	15.80	99.72

Method: H_2O:MeCN = 100:0 for 5 min, gradient to H_2O:MeCN = 50:50 over 5 min, gradient to H_2O:MeCN = 5:95 over 5 min, gradient to H_2O:MeCN = 0:100 over 5 min, H_2O:MeCN = 0:100 for 15 min.

Antiviral and cytotoxicity assays

Viruses and cells

Dengue virus type 2 (strain 16681) was obtained from Dr. Jochen Bodem, University of Wurzburg (Wurzburg, Germany), influenza virus (H1N1 A/Mexico/4108/2009) from Diagnostic Hybrids (Athens, USA), Human coxsackie B3 virus (strain Nancy) and human herpesvirus 1 (strain HF) were obtained from the American Type Culture Collection (ATCC, Manassas, USA). Madin-Darby canine kidney cells (MDCK) and HB-46 cells were obtained from ATCC, HeLa cells through the NIH AIDS Reagent Program, Division of AIDS, NIAID, NIH from Dr. Richard Axel, and Vero cells from the European Collection of Cell Cultures (Salisbury, UK). All cell lines were mycoplasma negative (routinely tested at Generi Biotech, Czech Republic) and maintained in Dulbecco's Modified Eagle's Medium (DMEM) with L-glutamine, 10 % fetal bovine serum (FBS), 100 U of penicillin/ml and 100 µg of streptomycin/ml (all Sigma-Aldrich, St. Louis, USA) in 5 % CO2 at 37 °C.

Screening of antiviral activity

Dengue virus

The anti-dengue activity was measured by determining the extent to which the test compounds inhibited replication in Vero cells as previously described.[4] Briefly, three-fold serial dilutions of compounds were added in triplicate in a 96-well plate with 20,000 Vero cells plated day before in DMEM medium. After 1 hour incubation dengue type 2 virus was added at multiplicity of infection 0.3 IU/cell. After three days of incubation cells were fixed, permeabilized, washed, incubated with DENV-2 specific antibody (harvested from HB-46 cells) overnight at 4 °C, followed by 1.5 hour incubation with Cy3-labeled donkey anti-mouse IgG (Jackson ImmunoResearch Europe) and documented using fluorescence microscope with camera (Carl Zeiss, Jena, Germany). Images were processed in ImageJ program (NIH) and drug concentrations required to reduce fluorescence by 50 % (EC50) were calculated using nonlinear regression analysis from plots of percentage of

fluorescent cells versus log10 drug concentration using nonlinear regression analysis with GraphPad Prism version 7.03 for Windows (GraphPad Software, La Jolla, USA).

Coxsackie virus, herpes virus, influenza virus

The anti-coxsackie, anti-herpes, anti-influenza activity was measured by determining the extent to which the test compounds inhibited virus-induced cytopathic effect in HeLa cells, Vero cells, and MDCK cells, respectively, as previously described[5] for anti-coxsackie activity. Briefly, three-fold serial dilutions of compounds were added in triplicate in a 96-well plate with 30,000 HeLa cells, 25,000 Vero cells, and 20,000 MDCK cells plated day before in DMEM medium with 2 % FBS, 2 % FBS, and 0.19 % bovine serum albumin with 1 μg/ml TPCK-trypsin, respectively. After one hour of incubation coxsackie virus, herpesvirus, and influenza virus was added at multiplicity of infection 0.005 IU/cell, 0.1 IU/cell, and 0.005 IU/cell, respectively. Following incubation at 37 °C in 5 % CO2 incubator for two days, three days, and two days, respectively, the cell viability was determined by addition of XTT solution (Sigma-Aldrich) for 4 hours and the absorbance of newly formed orange formazan solution was measured using Victor X3 plate reader (Perkin Elmer, Waltham, USA). Drug concentrations required to reduce viral cytopathic effect by 50 % (EC50) were calculated using nonlinear regression analysis from plots of percentage cell viability versus log10 drug concentration using GraphPad Prism v.7.03 (GraphPad Software).

HCV and RSV

Cell Culture. Huh-luc replicon cells were obtained from ReBlikon GmbH (Mainz, Germany). The cell lines were cultured in Dulbeccos's modified Eagles' medium (DMEM) with GlutaMAX-I (Invitrogen, Carlsbad, CA) supplemented with 10% fetal bovine serum (FBS; HyClone, Logan, UT), 1 U/mL penicillin (Invitrogen). GT1b (consensus) and GT2a (JFH1) are authentic subgenomic replicons of the indicated strain and replicon cell lines were created as previously reported.[6] Luciferase HEp-2 cells (ATCC, Manassas, VI) were maintained in MEM media supplemented with 10% FBS and penicillin/streptomycin. The cells were passaged twice per week

to maintain sub-confluent densities. The cell expression was quantified after 3 days of incubation using a commercial luciferase assay (Promega, Madison, WI). RSV strain A2 was obtained from Advanced Biotechnologies (Columbia, MD).

Antiviral EC_{50} determination. Determination of 50% effective concentration (EC_{50}) was conducted in HCV replicon assay as previously described.[7] Briefly, replicon cells were seeded into 96-well plates. Compounds were serially diluted in DMSO at 200× final concentrations and then added to the assay plate. Luciferase expression was quantified after 3 days of incubation using a commercial luciferase assay (Promega, Madison, WI). Data were fit to the logistic dose response equation $y = a/(1+(x/b)^c)$, and EC_{50} values were calculated from the resulting equations as described previously.[8] The anti-RSV activity was tested based on methods published previously.[9] Compounds were 3-fold serially diluted in source plates from which 100 nL of the diluted compound was transferred to a 384-well cell culture plate using an acoustic transfer apparatus (Echo, Labcyte, Sunnyvale, CA). HEp-2 cells at a density of 50,000 cells/mL were then infected by adding RSV strain A2 (Advanced Biotechnologies, Columbia, MD) at a titer of $1 \times 10^{4.5}$ tissue culture infectious doses/mL. The cells were cultured for 4 days at 37°C and the RSV-induced cytopathic effect was determined by CellTiter-GloTM Viability Reagent (Promega Biosciences, Inc., Madison, WI) using an Envision plate reader (Perkin Elmer, Waltham, MA). Data were fit to the logistic dose response equation $y = a/(1+(x/b)^c)$ as described above.

Manual Modelling using Moloc

Diphosphates derived from three aminonucleosides **9a**, **17a** and **23a** were manually docked within the known co-crystal structure of viral RNA-dependent RNA polymerase HCV NS5B genotype 2A in complex with RNA template 5'-AUCC, RNA primer 5'-PGG, Mn^{2+} and ADP (PDB code 4WTJ).[10] The enzyme and RNA structures without ADP were fixed and the energy of the system

was minimized using the MAB force field,[11] as impletented in the computer program Moloc. Evaluation of binding modes of all three nucleotides was done manually.

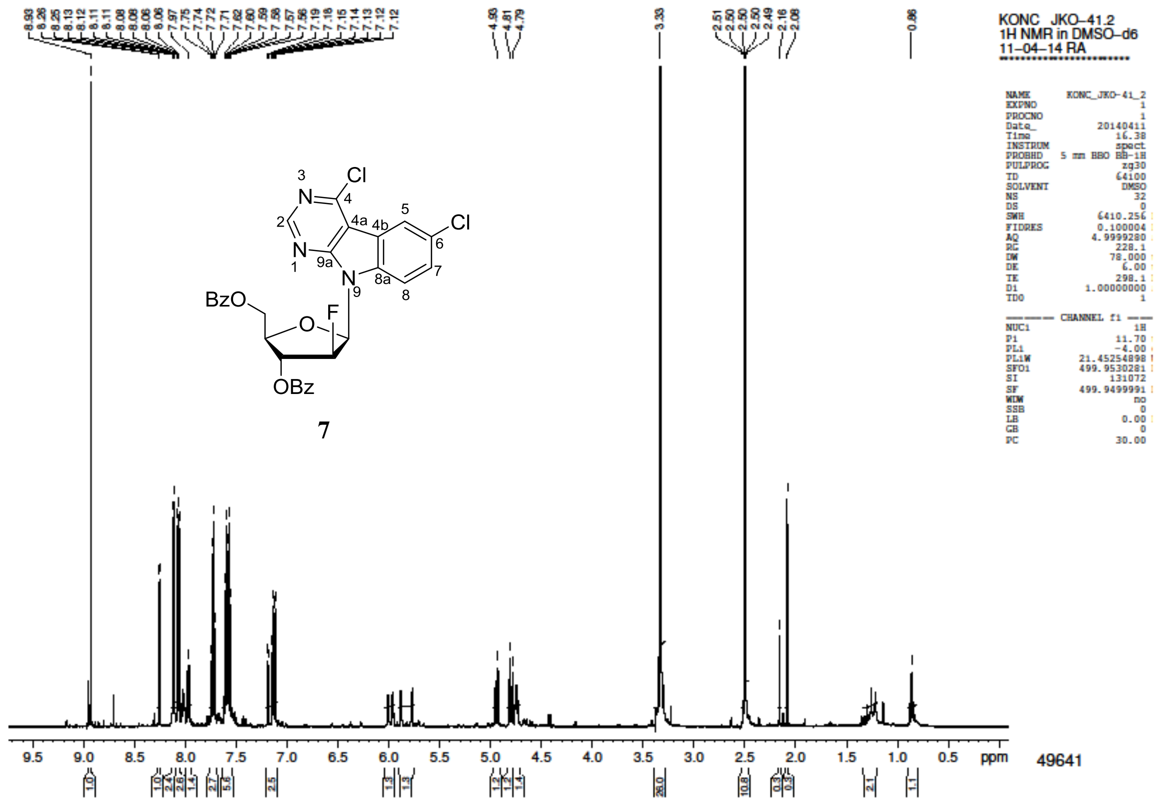
KONC JKO-41.2
1H NMR in DMSO-d6
11-04-14 RA
7
49641
ppm

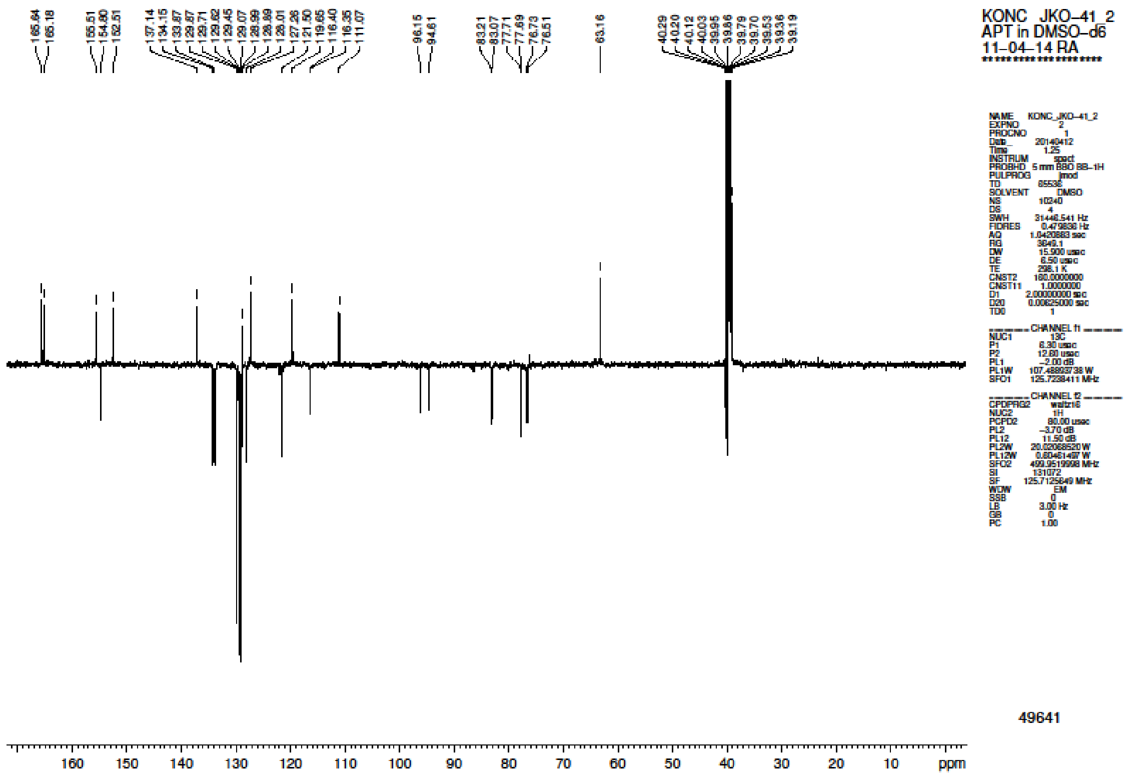
KONC JKO-41_2
APT in DMSO-d6
11-04-14 RA
49641
ppm

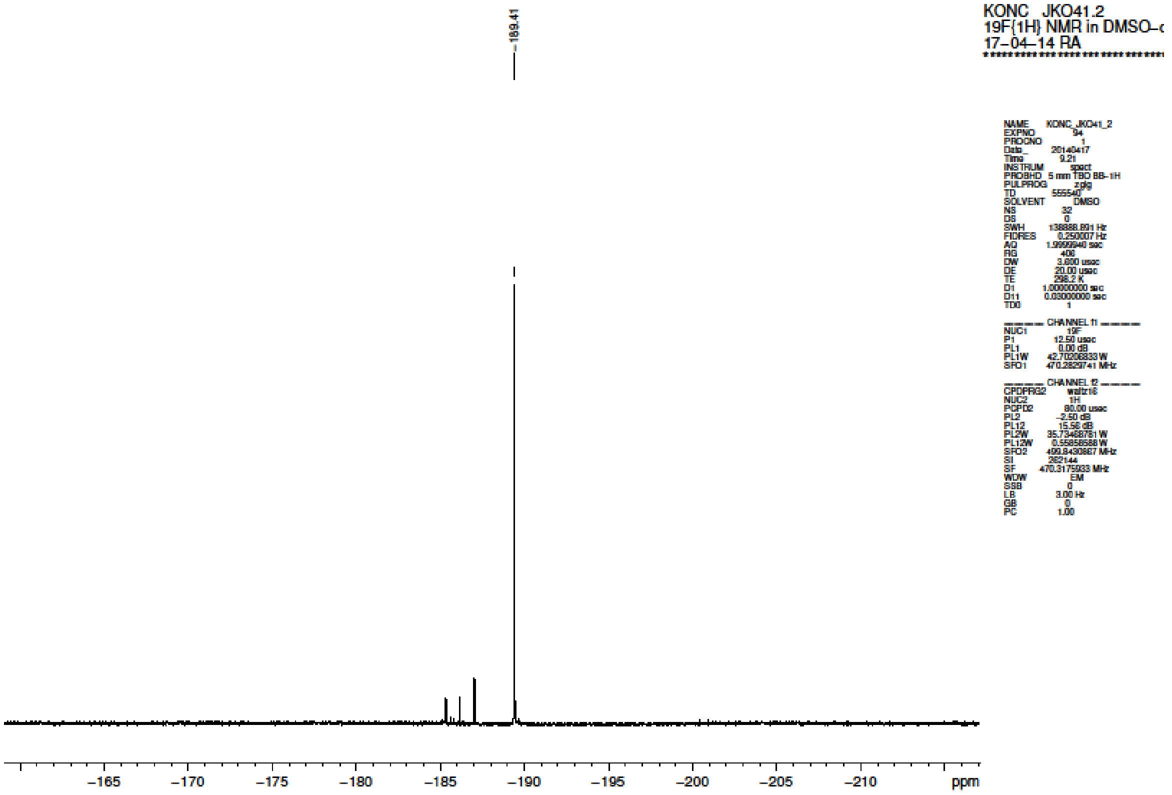
KONC JKO41.2
19F{1H} NMR in DMSO-c
17-04-14 RA
-189.41
-165 -170 -175 -180 -185 -190 -195 -200 -205 -210 ppm

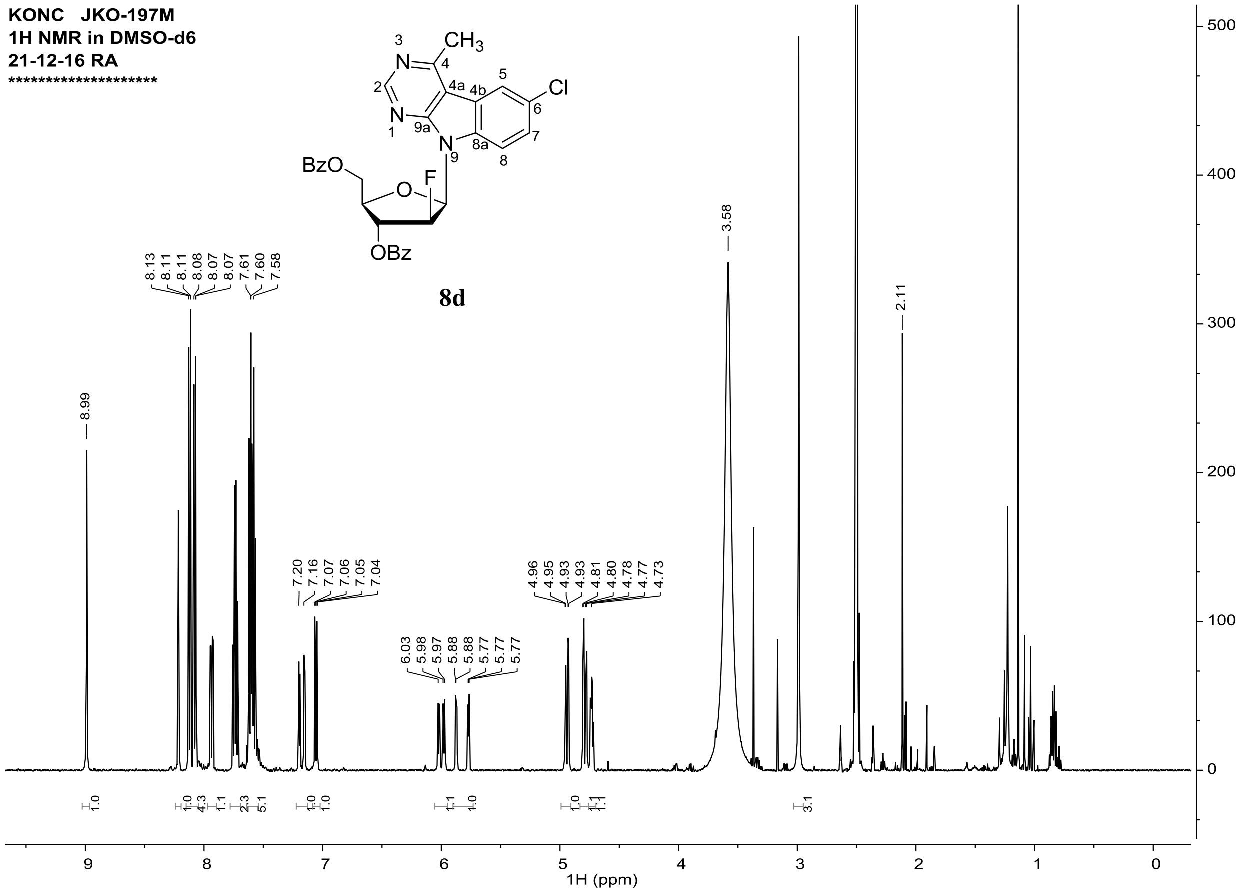
KONC JKO-197M
1H NMR in DMSO-d6
21-12-16 RA
8d
BzO
OBz
8.99
8.13 8.11 8.11 8.08 8.07 8.07 7.61 7.60 7.58
7.20 7.16 7.07 7.06 7.05 7.04
6.03 5.98 5.97 5.88 5.88 5.77 5.77 5.77
4.96 4.95 4.93 4.93 4.81 4.80 4.78 4.77 4.73
3.58
2.11
1H (ppm)

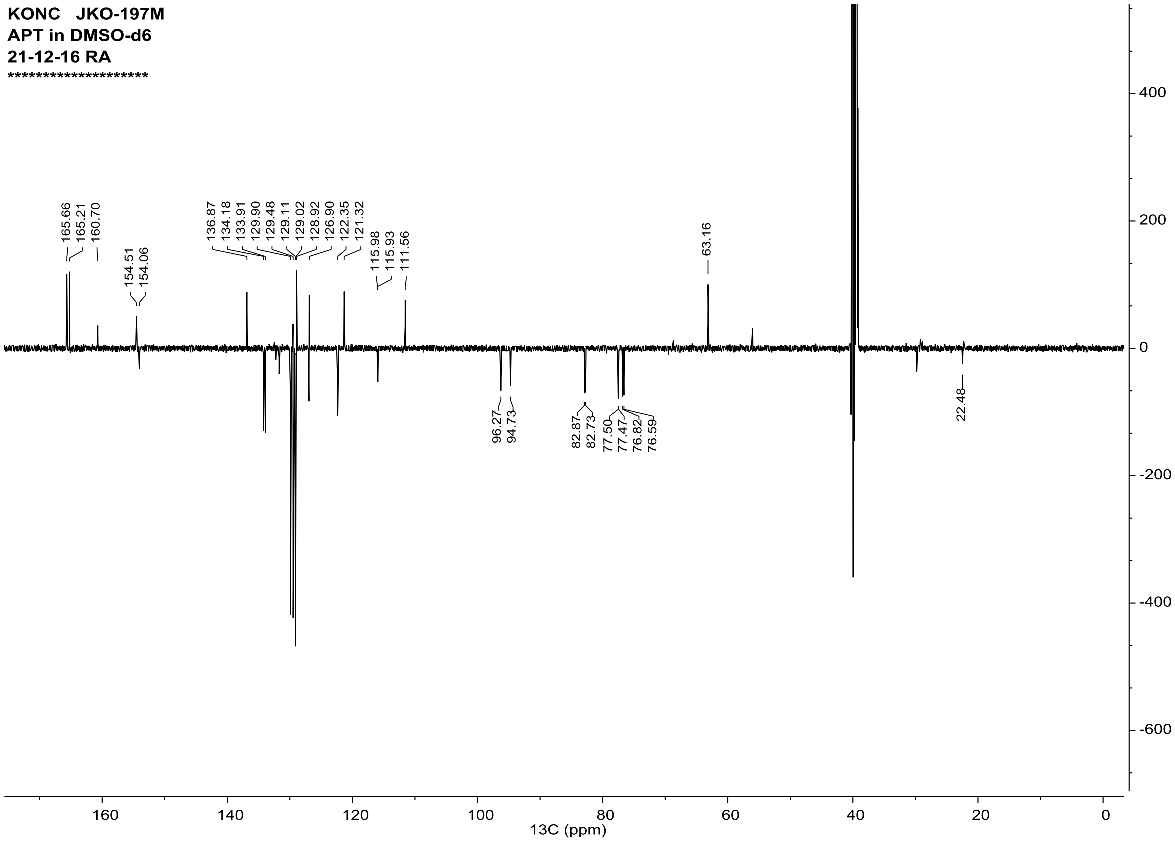
KONC JKO-197M
APT in DMSO-d6
21-12-16 RA
****
165.66
165.21
160.70
154.51
154.06
136.87
134.18
133.91
129.90
129.48
129.11
129.02
128.92
126.90
122.35
121.32
115.98
115.93
111.56
63.16
96.27
94.73
82.87
82.73
77.50
77.47
76.82
76.59
22.48
13C (ppm)

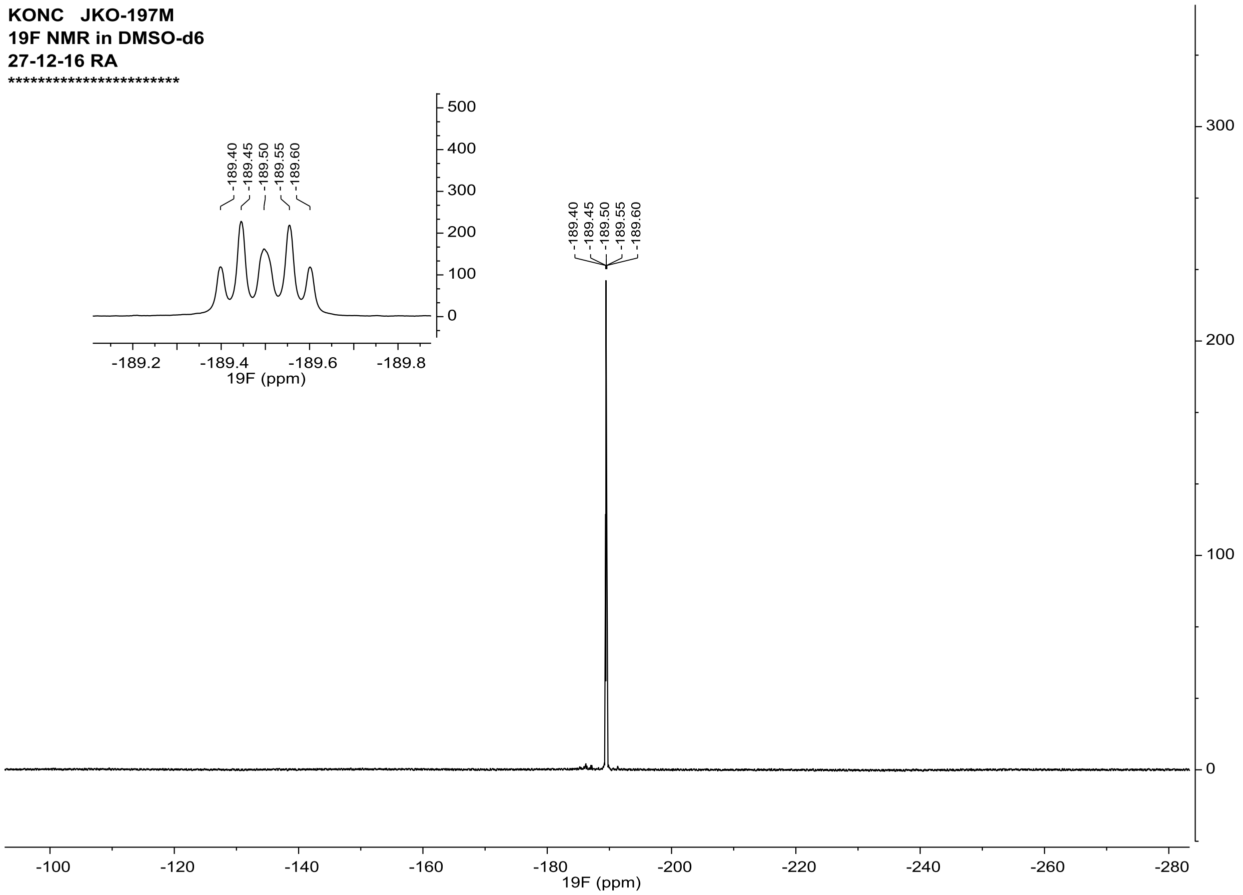
KONC JKO-197M
19F NMR in DMSO-d6
27-12-16 RA
****
-189.40
-189.45
-189.50
-189.55
-189.60
19F (ppm)

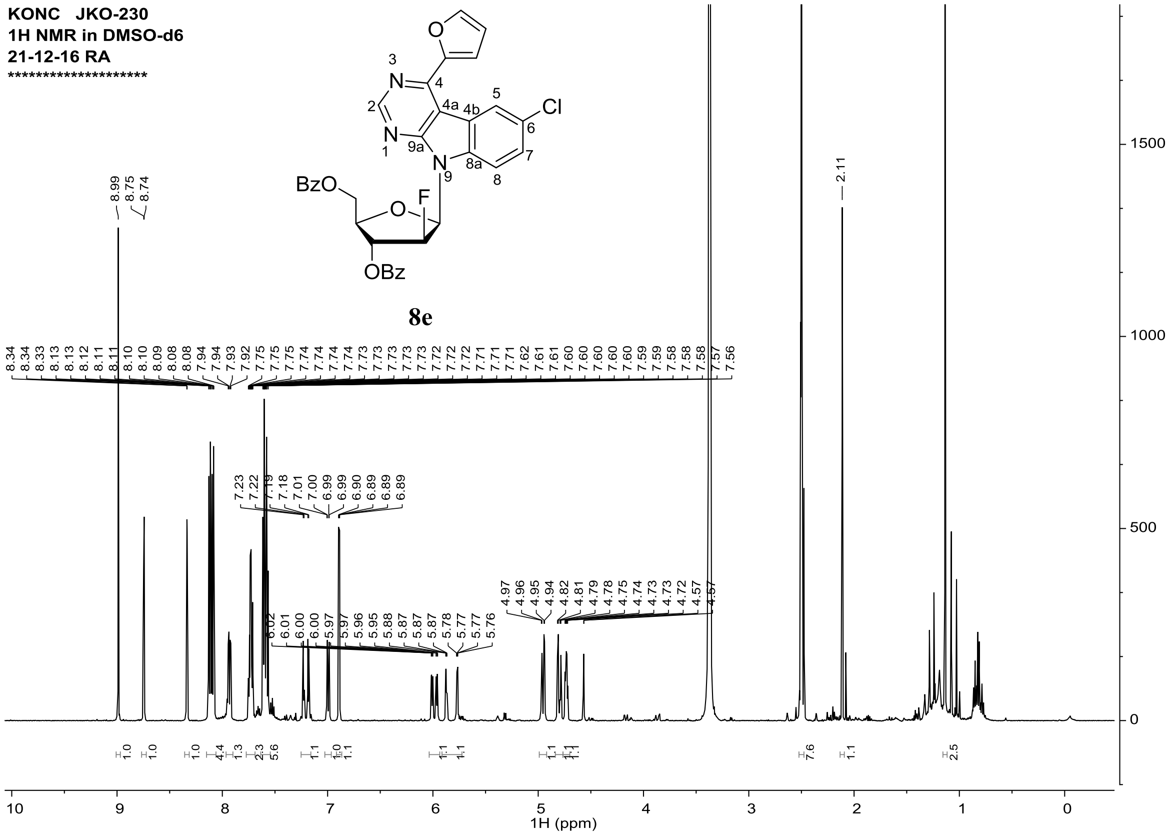
KONC JKO-230
1H NMR in DMSO-d6
21-12-16 RA
8e
1H (ppm)

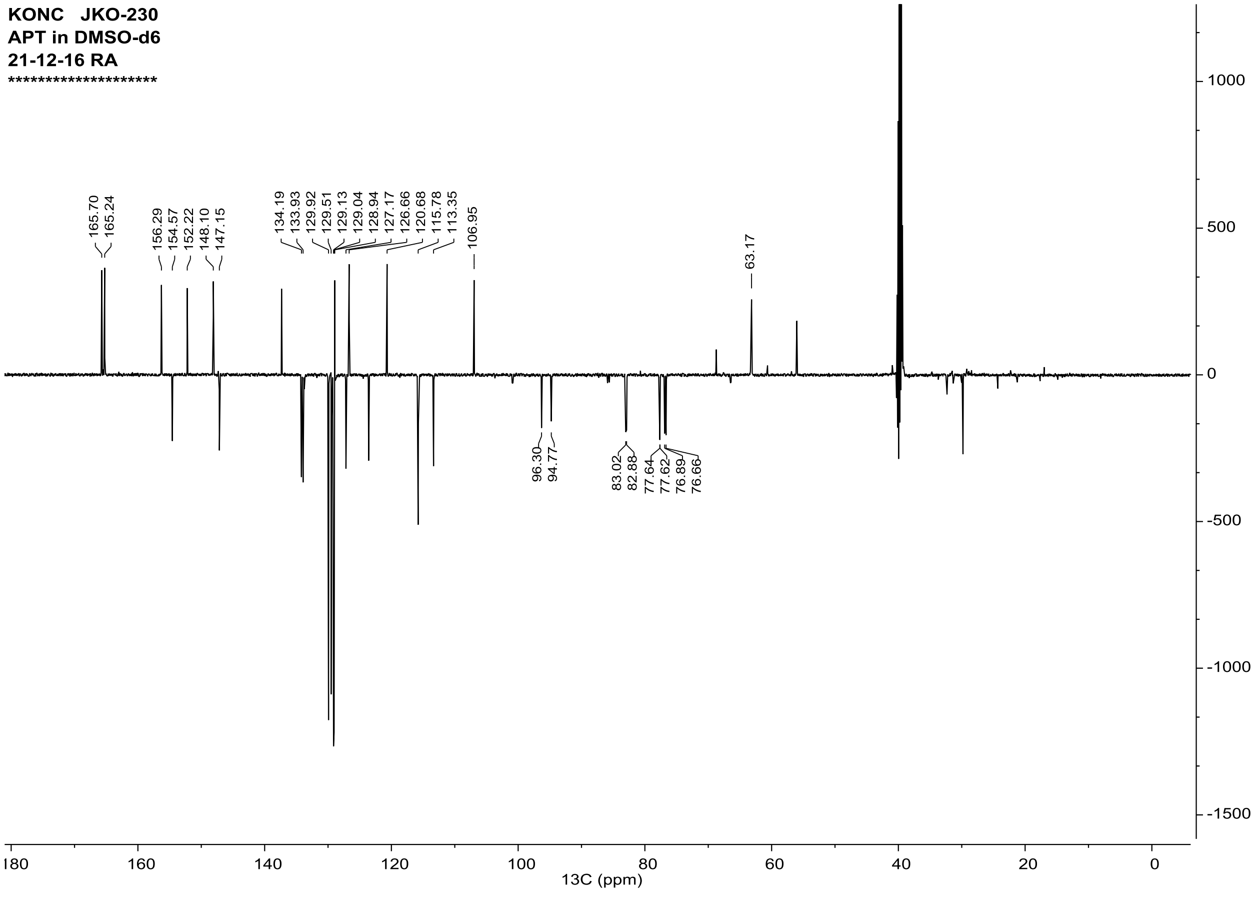
KONC JKO-230
APT in DMSO-d6
21-12-16 RA
13C (ppm)

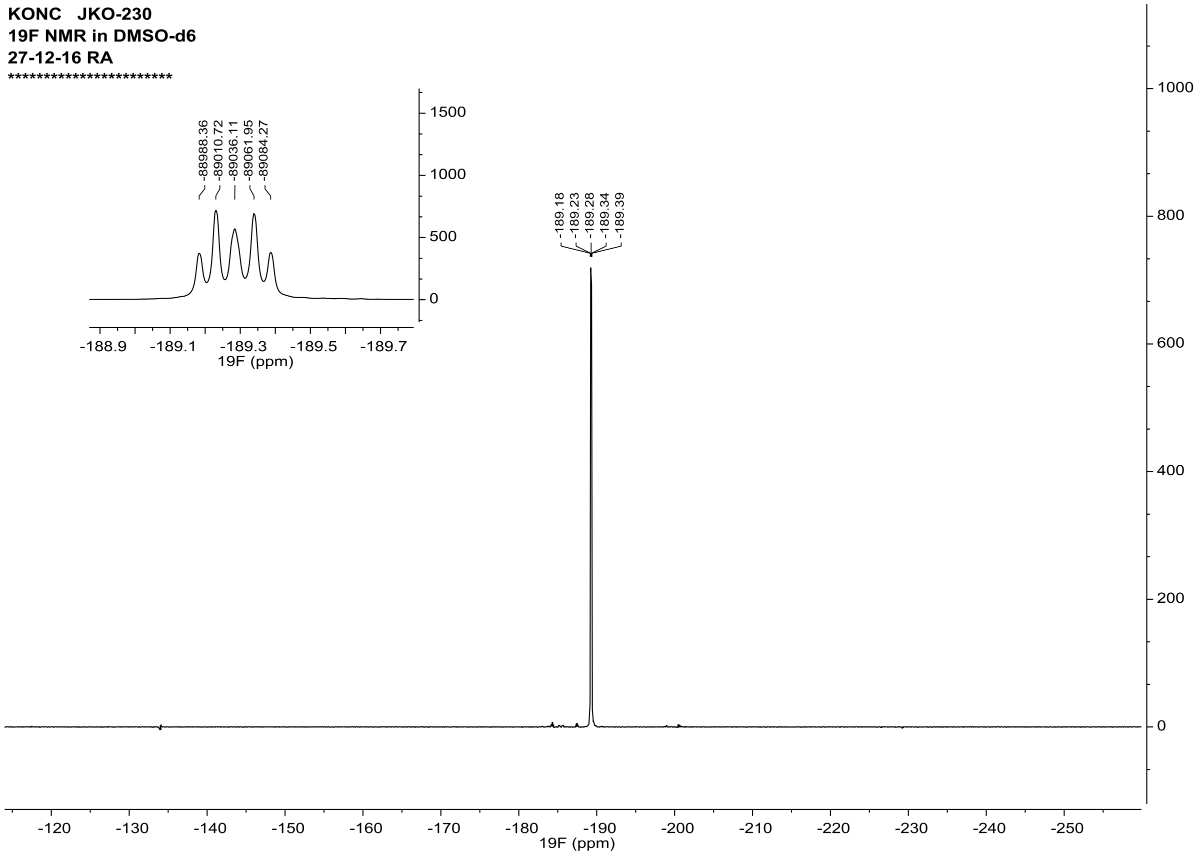
KONC JKO-230
19F NMR in DMSO-d6
27-12-16 RA
****
-88988.36
-89010.72
-89036.11
-89061.95
-89084.27
-189.18
-189.23
-189.28
-189.34
-189.39
19F (ppm)

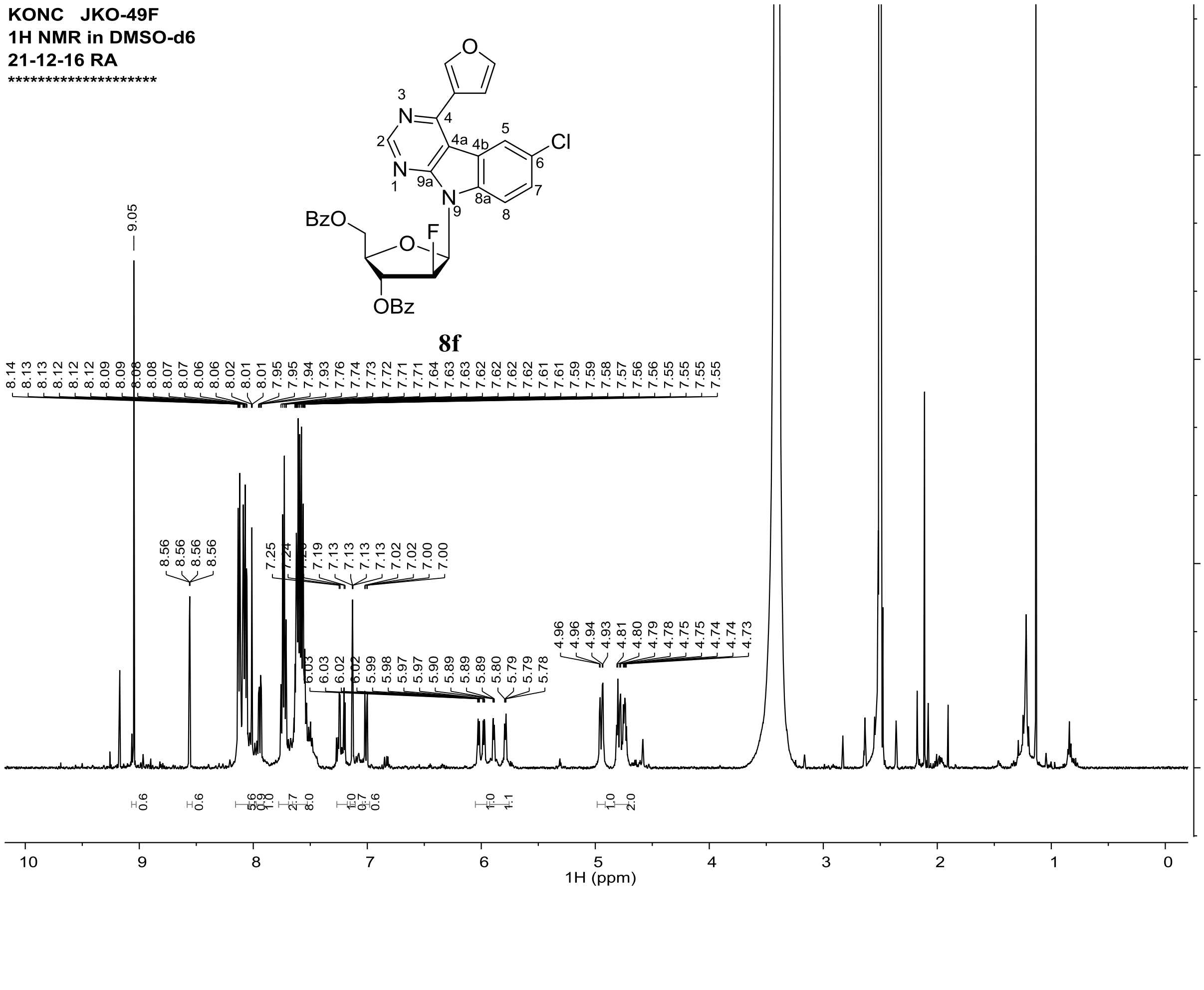
KONC JKO-49F
1H NMR in DMSO-d6
21-12-16 RA
****
8f
BzO
OBz
F
Cl
9.05
8.56
8.56
8.56
8.56
1H (ppm)

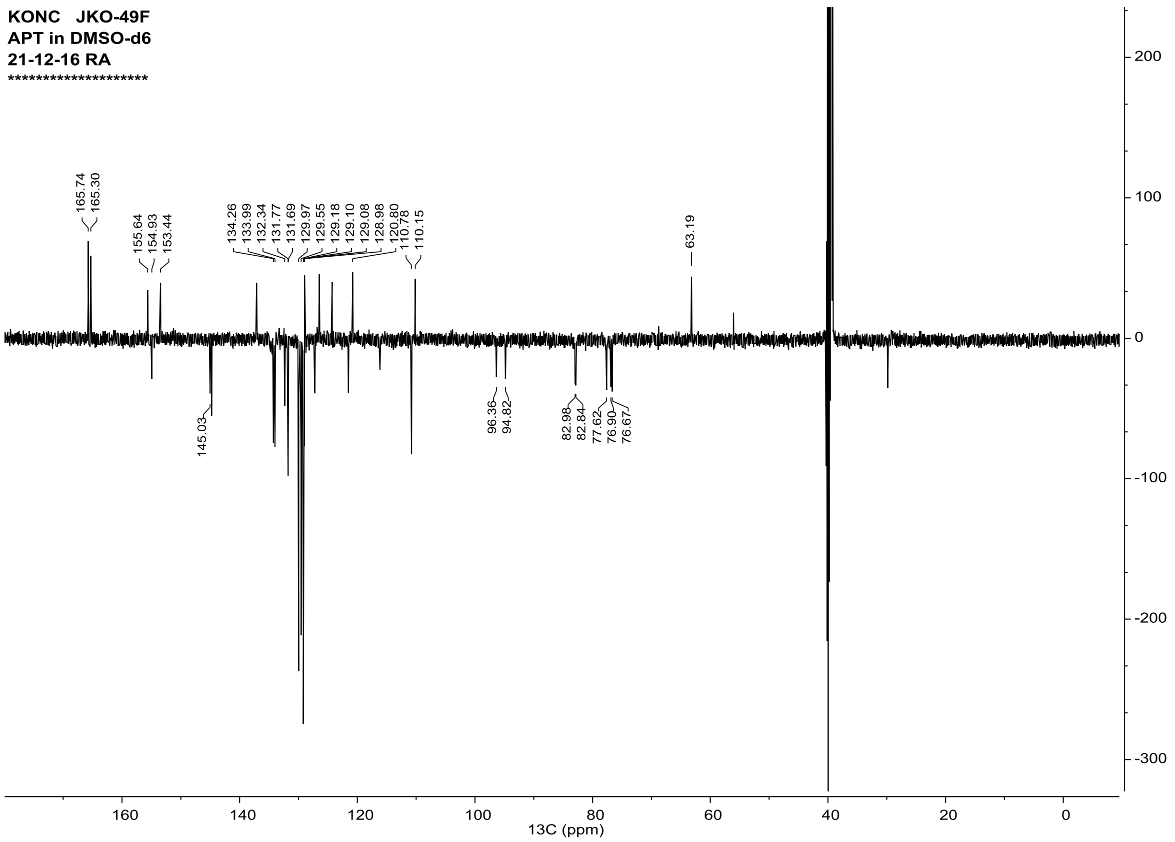
KONC JKO-49F
APT in DMSO-d6
21-12-16 RA
****
165.74
165.30
155.64
154.93
153.44
134.26
133.99
132.34
131.77
131.69
129.97
129.55
129.18
129.10
129.08
128.98
120.80
110.78
110.15
63.19
145.03
96.36
94.82
82.98
82.84
77.62
76.90
76.67
13C (ppm)

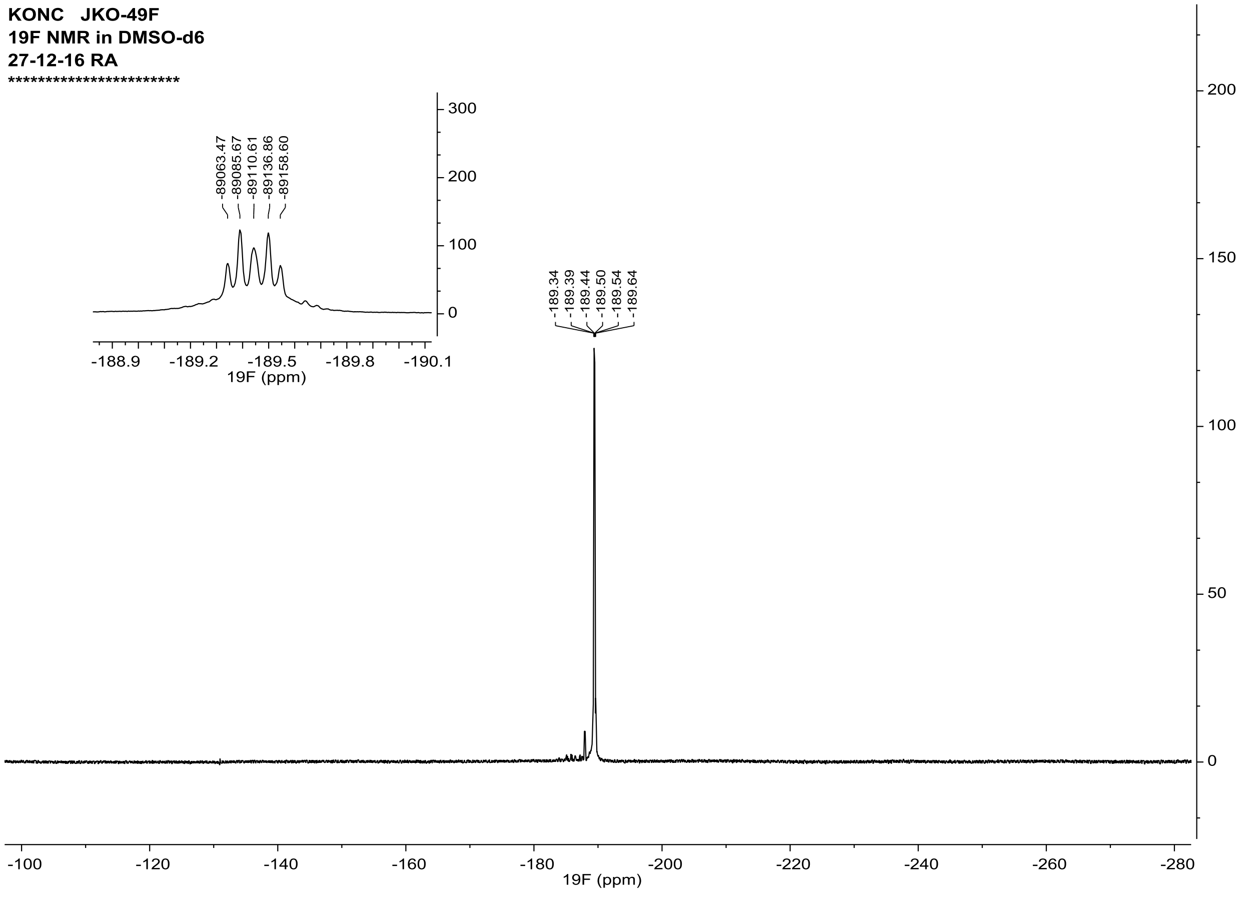
KONC JKO-49F
19F NMR in DMSO-d6
27-12-16 RA
****
-89063.47
-89085.67
-89110.61
-89136.86
-89158.60
19F (ppm)
-189.34
-189.39
-189.44
-189.50
-189.54
-189.64
19F (ppm)

KONC JKO-231
1H NMR in DMSO-d6
23-12-16 RA
****

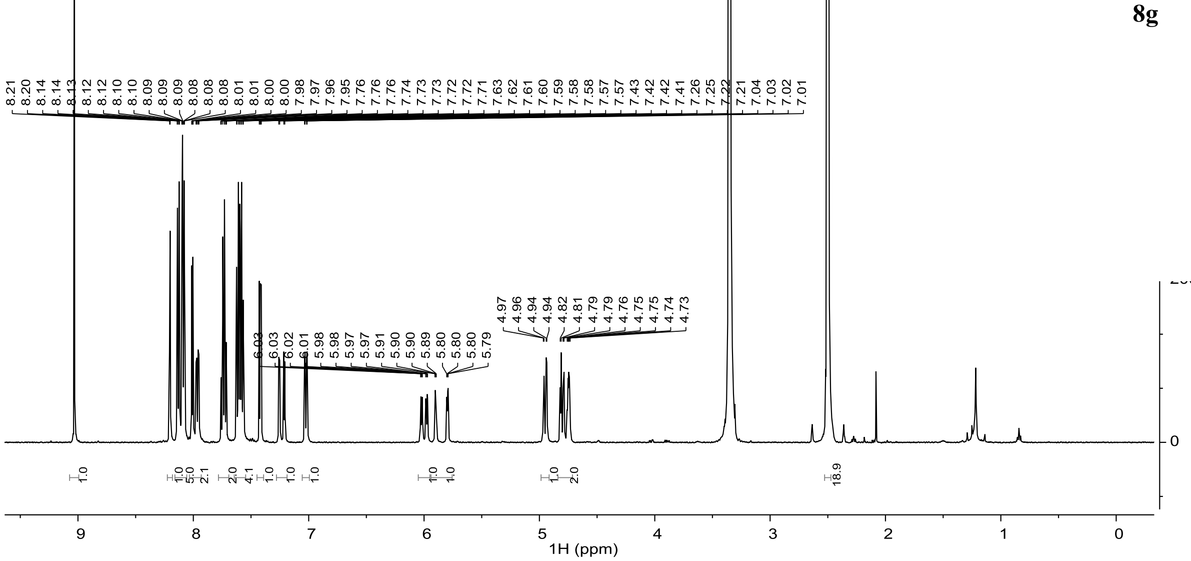

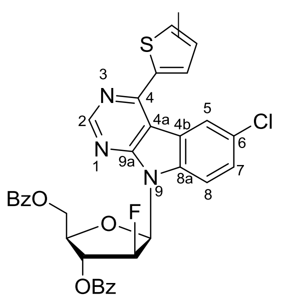

8g

KONC JKO-231
APT in DMSO-d6
23-12-16 RA
****

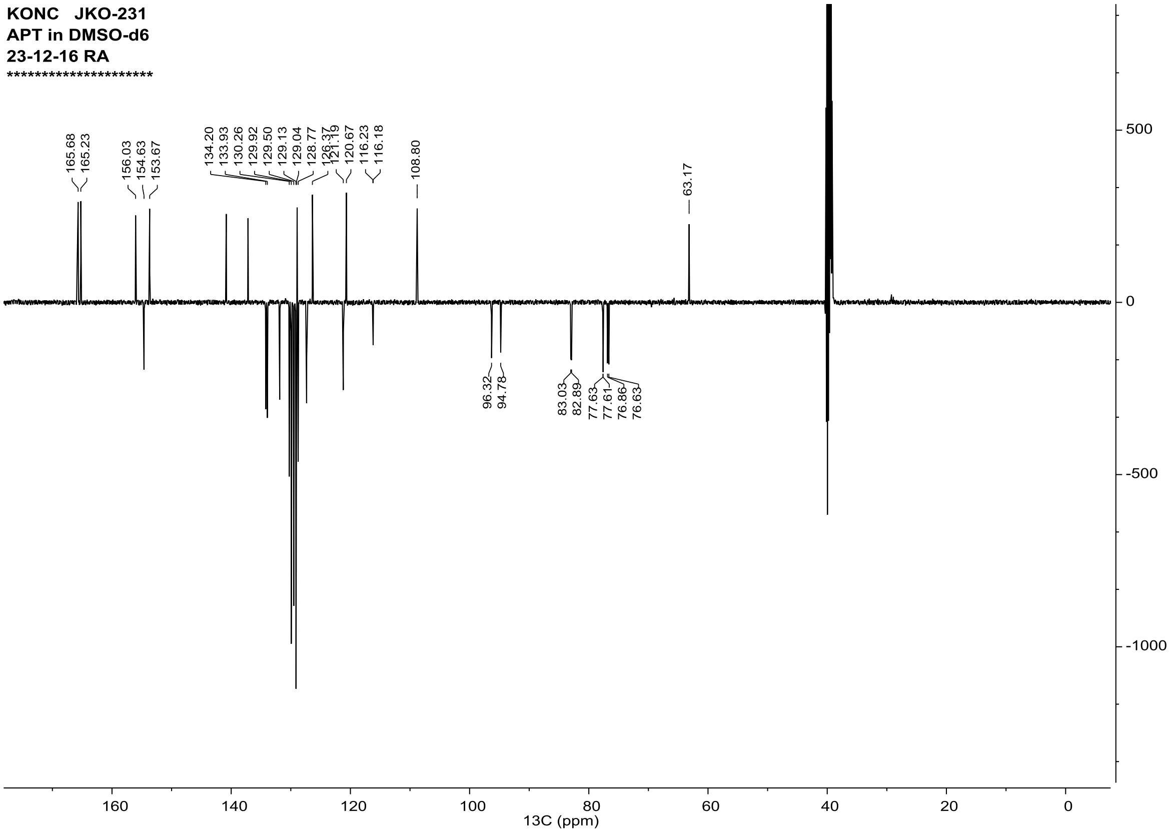

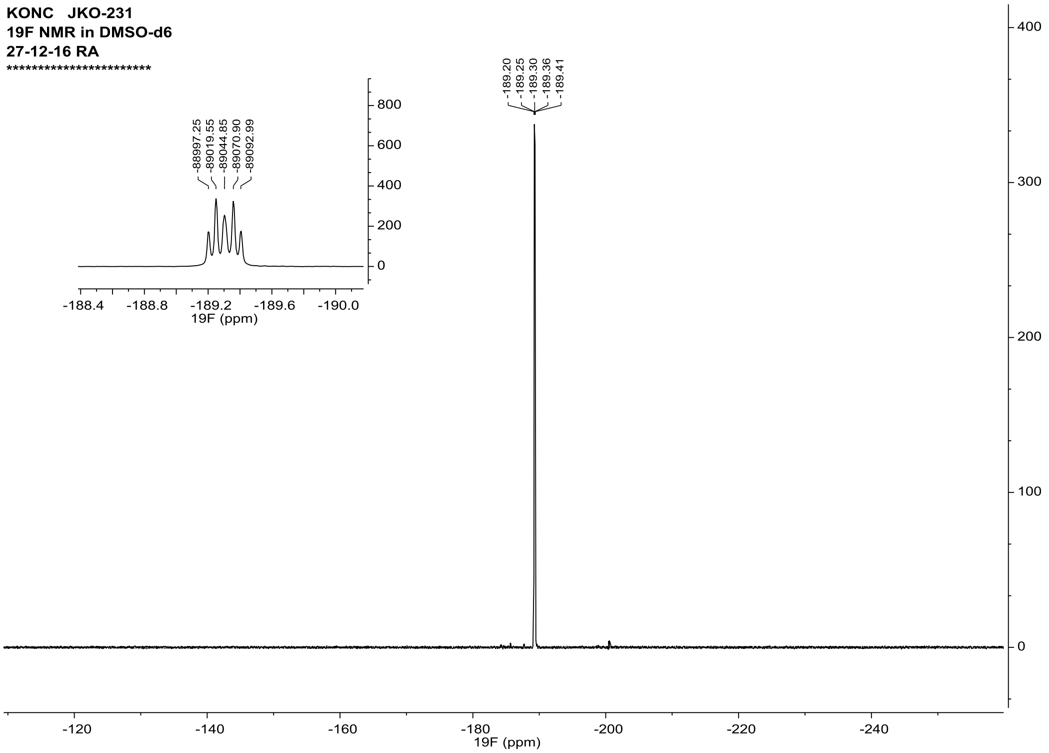
KONC JKO-231
19F NMR in DMSO-d6
27-12-16 RA
****
-189.20
-189.25
-189.30
-189.36
-189.41
-88997.25
-89019.55
-89044.85
-89070.90
-89092.99
19F (ppm)
19F (ppm)

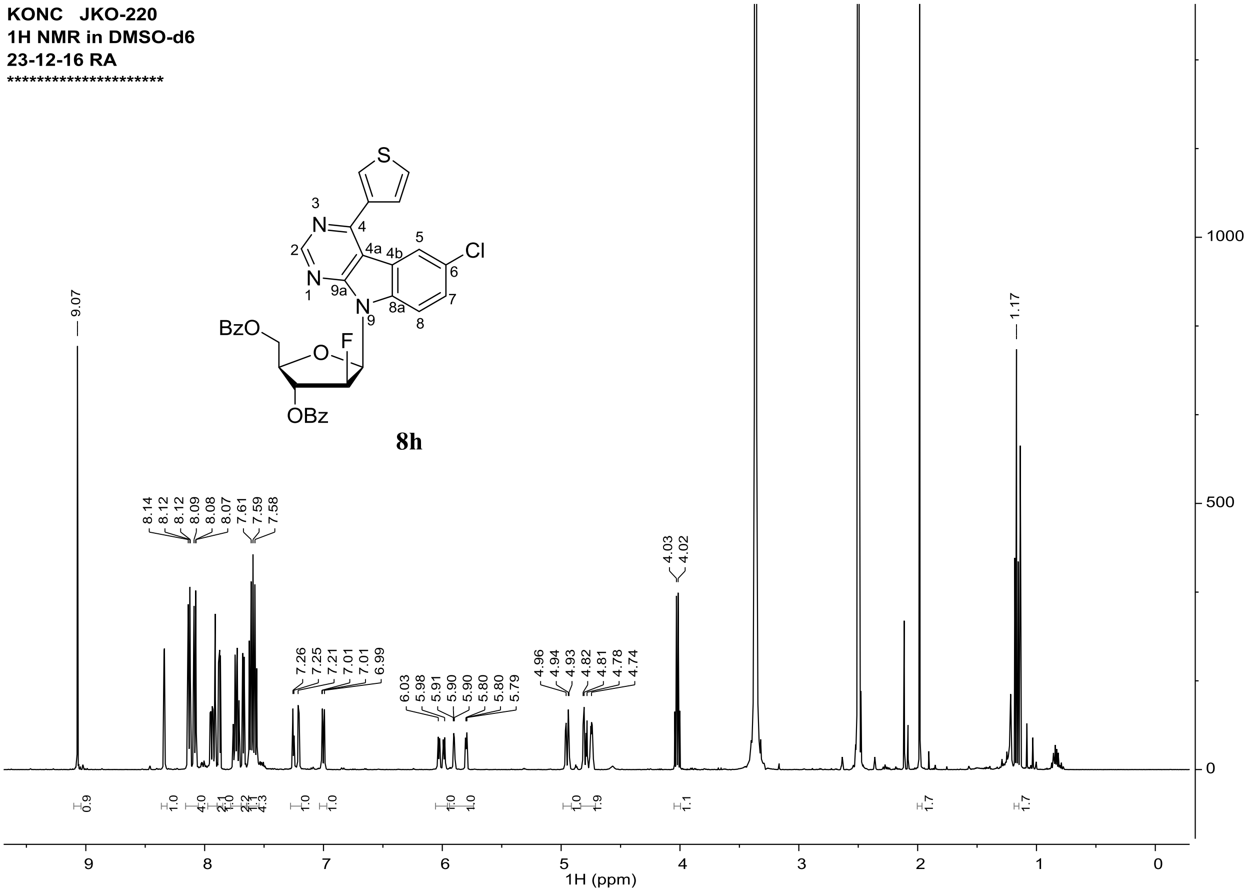
KONC JKO-220
1H NMR in DMSO-d6
23-12-16 RA
****
BzO
OBz
F
O
N
S
Cl
8h
9.07
8.14
8.12
8.12
8.09
8.08
8.07
7.61
7.59
7.58
7.26
7.25
7.21
7.01
7.01
6.99
6.03
5.98
5.91
5.90
5.90
5.80
5.80
5.79
4.96
4.94
4.93
4.82
4.81
4.78
4.74
4.03
4.02
1.17
1H (ppm)

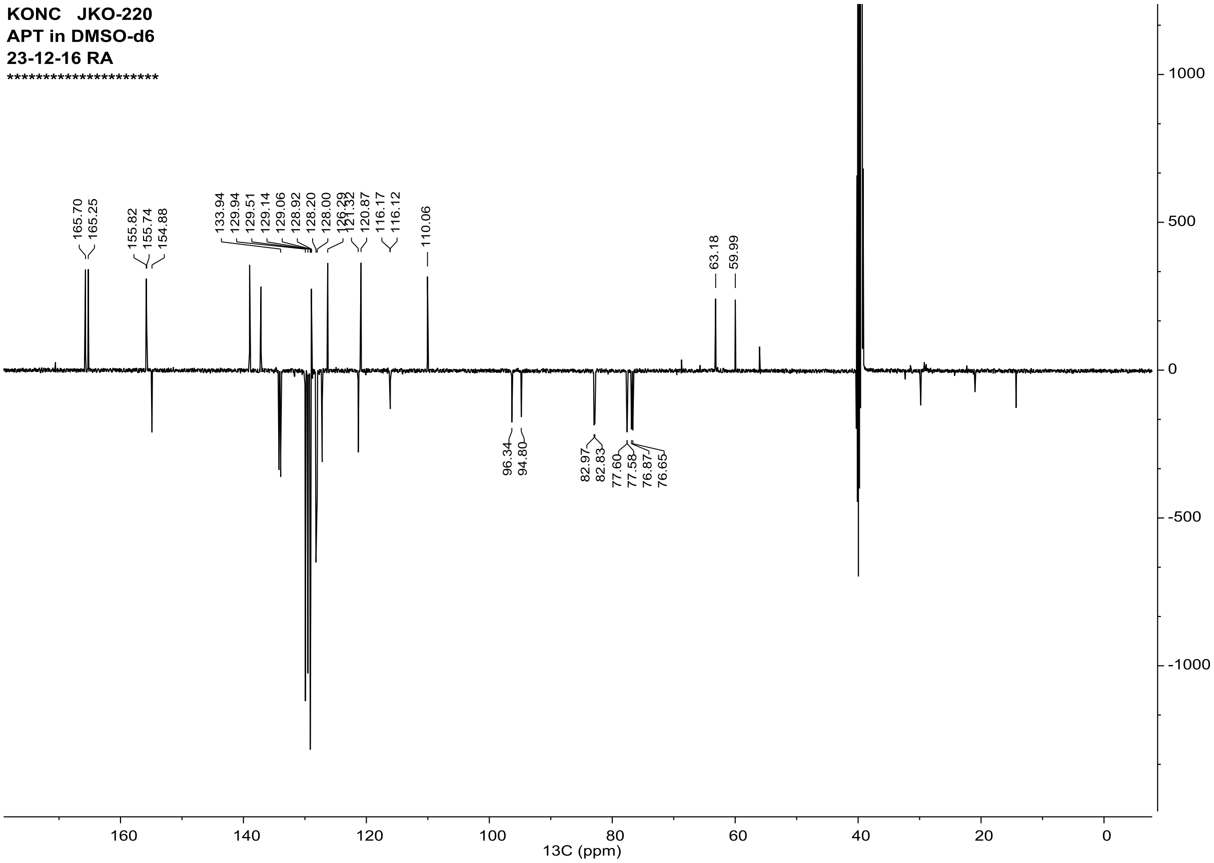

KONC JKO-220
APT in DMSO-d6
23-12-16 RA
****
13C (ppm)

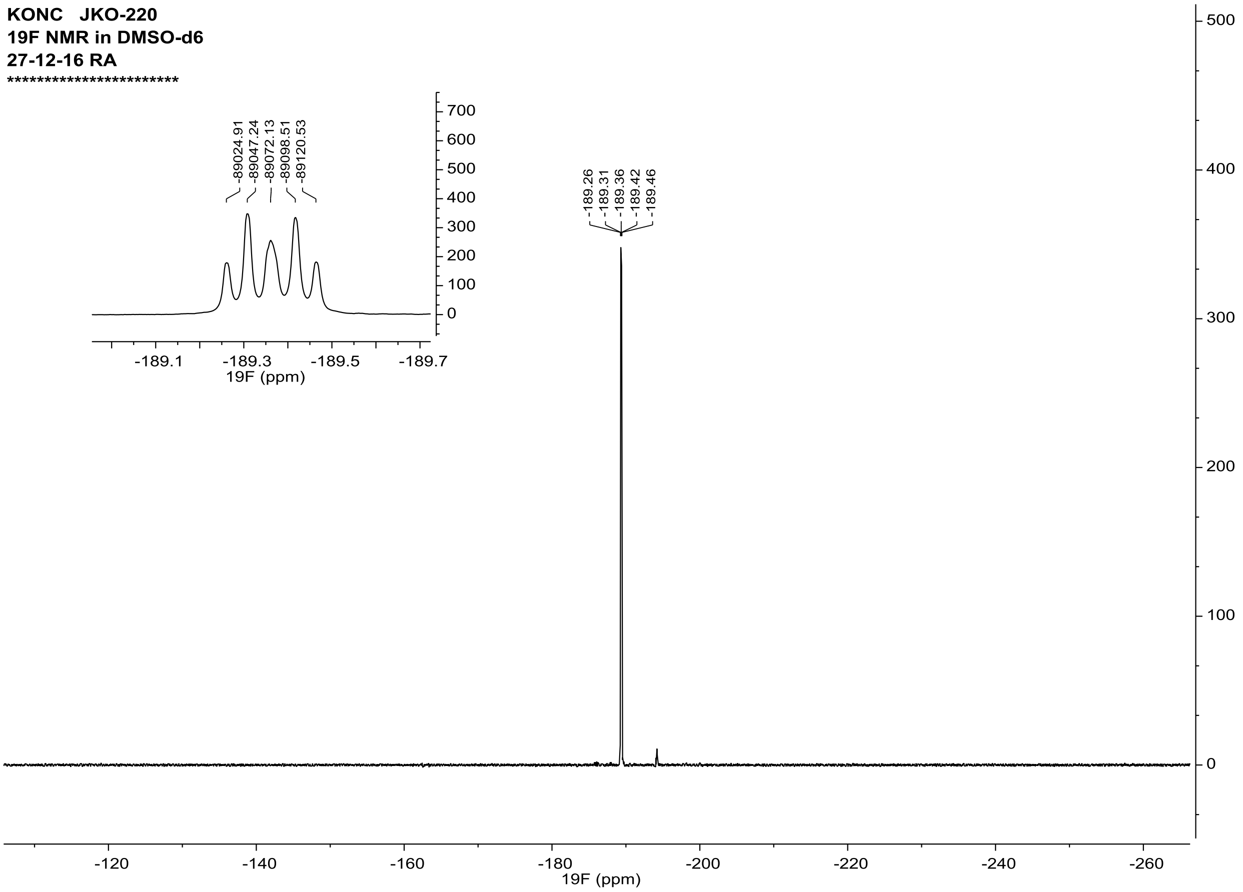

KONC JKO-220
19F NMR in DMSO-d6
27-12-16 RA
****
19F (ppm)

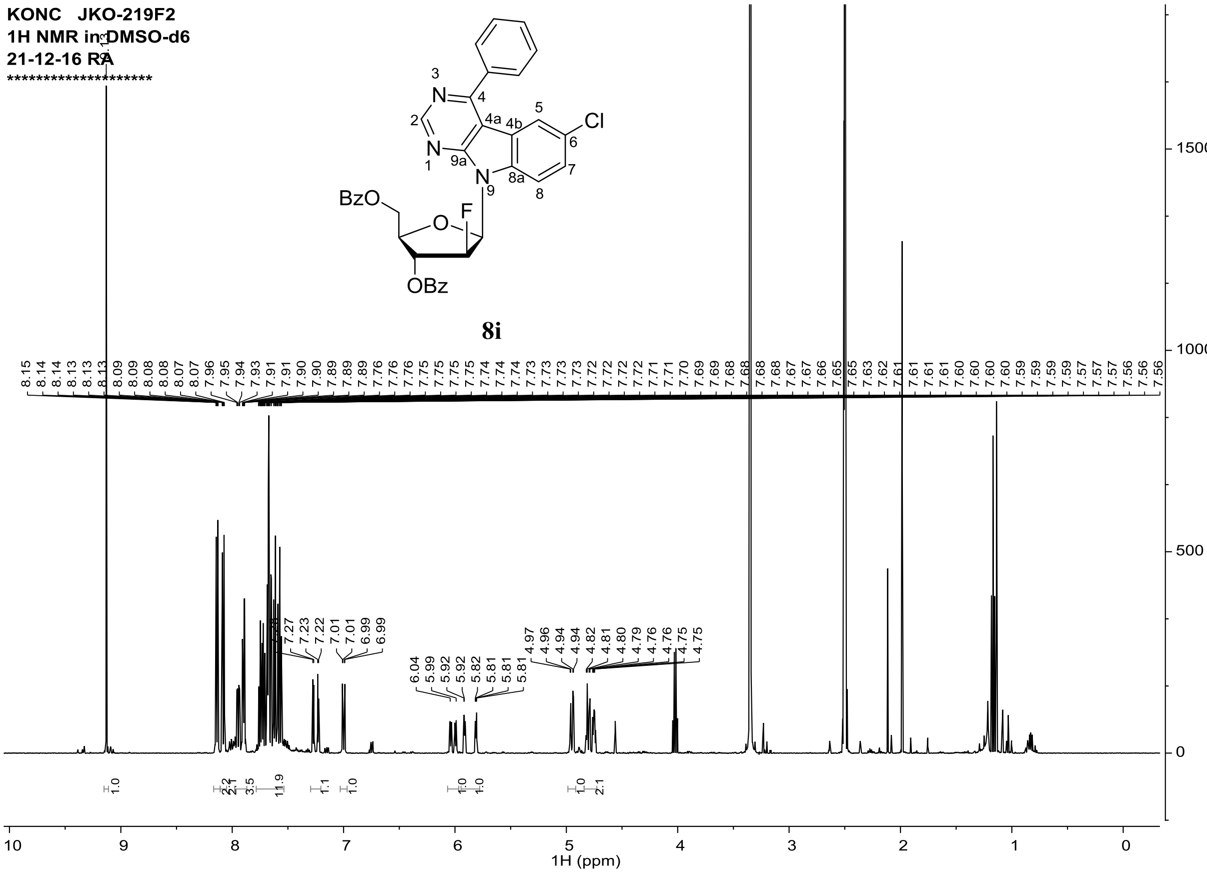
KONC JKO-219F2
1H NMR in DMSO-d6
21-12-16 RA
BzO
O
F
OBz
N
Cl
8i
8.15 8.14 8.14 8.13 8.13 8.13 8.09 8.09 8.08 8.08 8.07 8.07 7.96 7.95 7.94 7.93 7.91 7.91 7.90 7.90 7.89 7.89 7.76 7.76 7.76 7.75 7.75 7.75 7.74 7.74 7.74 7.73 7.73 7.73 7.72 7.72 7.72 7.72 7.71 7.70 7.69 7.69 7.68 7.68 7.68 7.67 7.67 7.66 7.65 7.65 7.63 7.62 7.61 7.61 7.61 7.61 7.60 7.60 7.60 7.60 7.59 7.59 7.59 7.57 7.57 7.56 7.56 7.56
7.27 7.23 7.22 7.01 7.01 6.99 6.99
6.04 5.99 5.92 5.92 5.82 5.81 5.81 5.81
4.97 4.96 4.94 4.94 4.82 4.81 4.80 4.79 4.76 4.76 4.75 4.75
1.0 2.2 2.1 3.5 11.9 1.1 1.0 1.0 1.0 1.0 2.1
1H (ppm)

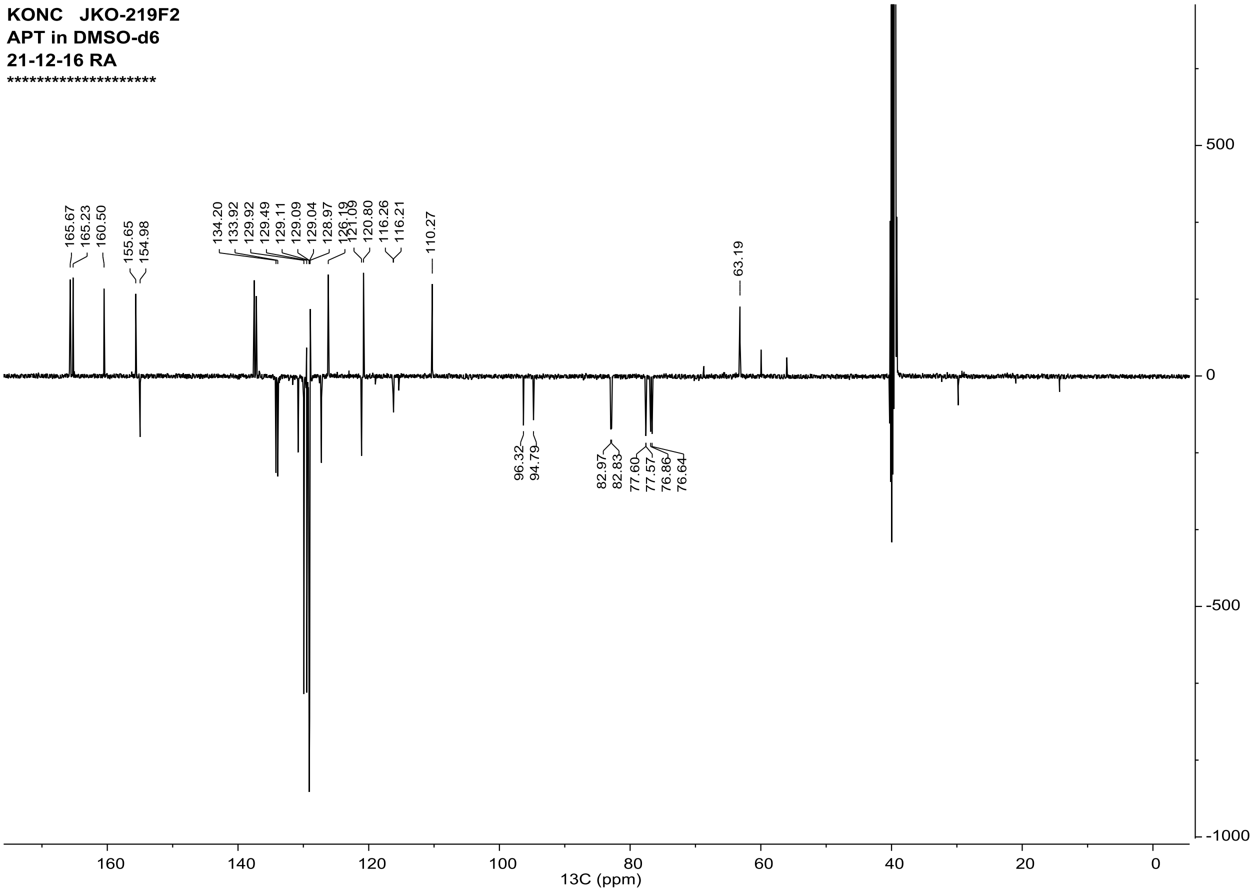
KONC JKO-219F2
APT in DMSO-d6
21-12-16 RA
165.67 165.23 160.50 155.65 154.98
134.20 133.92 129.92 129.49 129.11 129.09 129.04 128.97 126.19 121.09 120.80 116.26 116.21 110.27
63.19
96.32 94.79
82.97 82.83 77.60 77.57 76.86 76.64
13C (ppm)

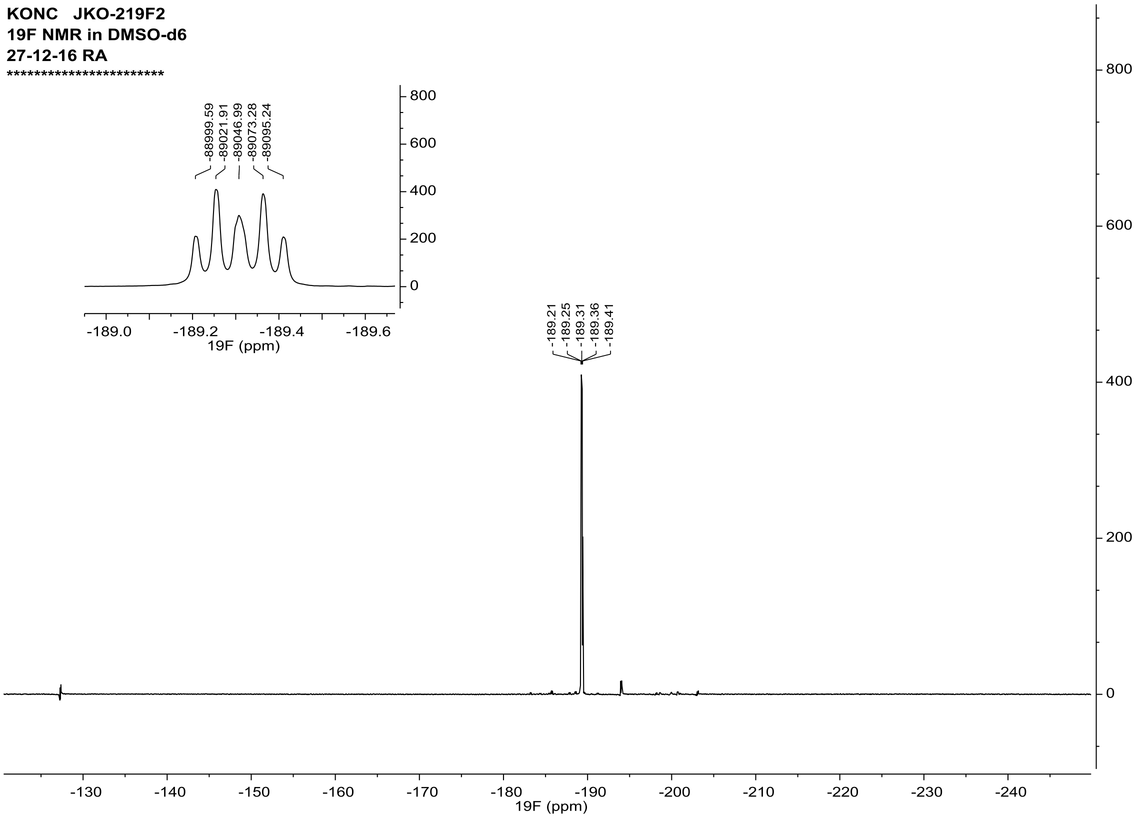
KONC JKO-219F2
19F NMR in DMSO-d6
27-12-16 RA
-88999.59
-89021.91
-89046.99
-89073.28
-89095.24
-189.21
-189.25
-189.31
-189.36
-189.41
19F (ppm)

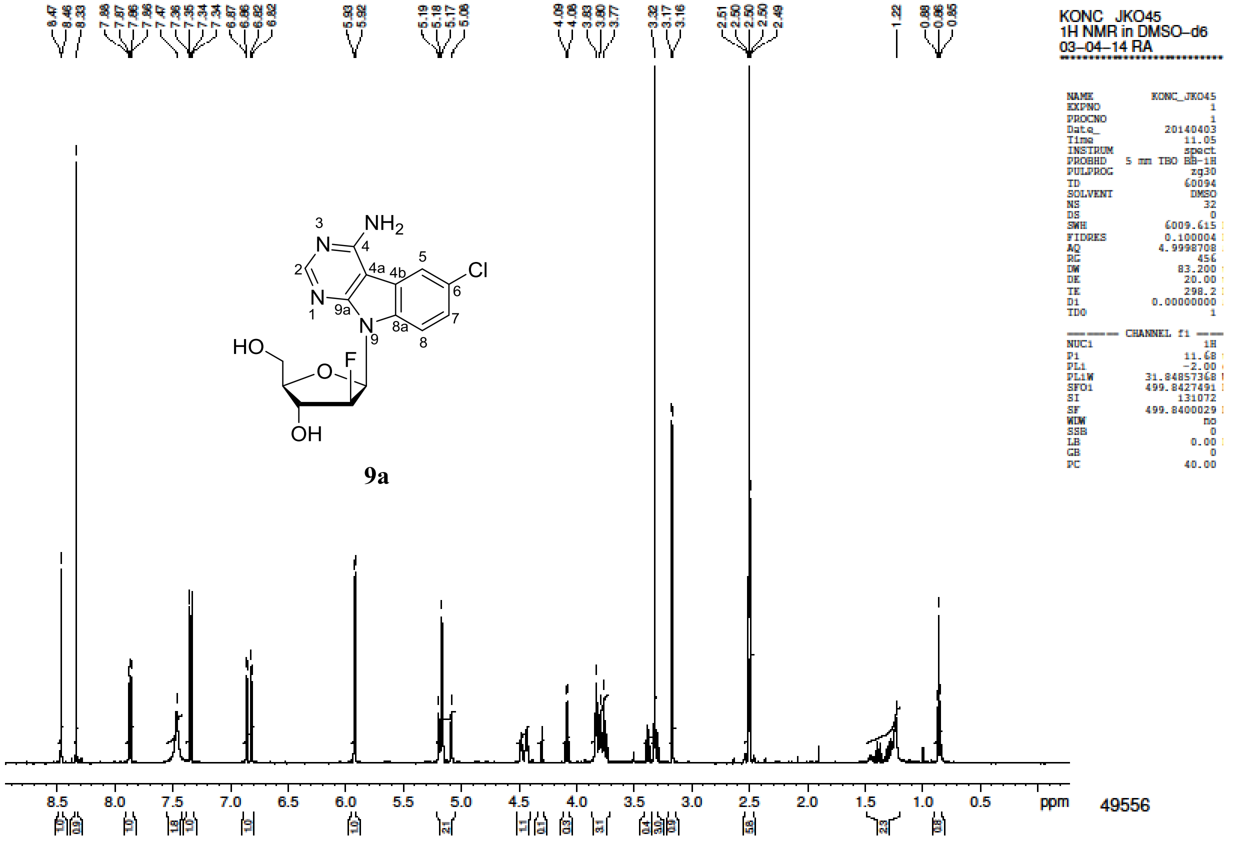
KONC JKO45
1H NMR in DMSO-d6
03-04-14 RA
NH2
Cl
HO
F
OH
9a
ppm
49556

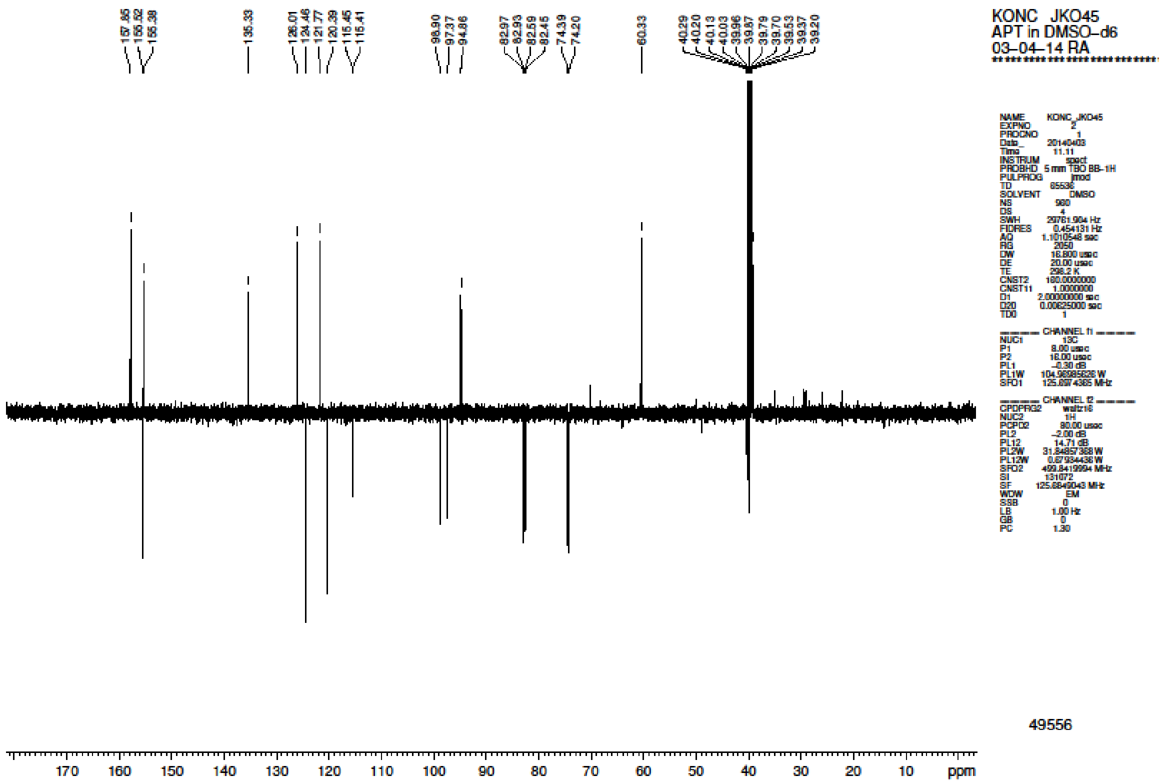
KONC JKO45
APT in DMSO–d6
03–04–14 RA
157.65
156.62
155.39
135.33
126.01
124.46
121.77
120.39
115.45
115.41
98.90
97.37
94.86
82.97
82.93
82.59
82.45
74.39
74.20
60.33
40.29
40.20
40.13
40.03
39.96
39.87
39.79
39.70
39.53
39.37
39.20
49556
170
160
150
140
130
120
110
100
90
80
70
60
50
40
30
20
10
ppm

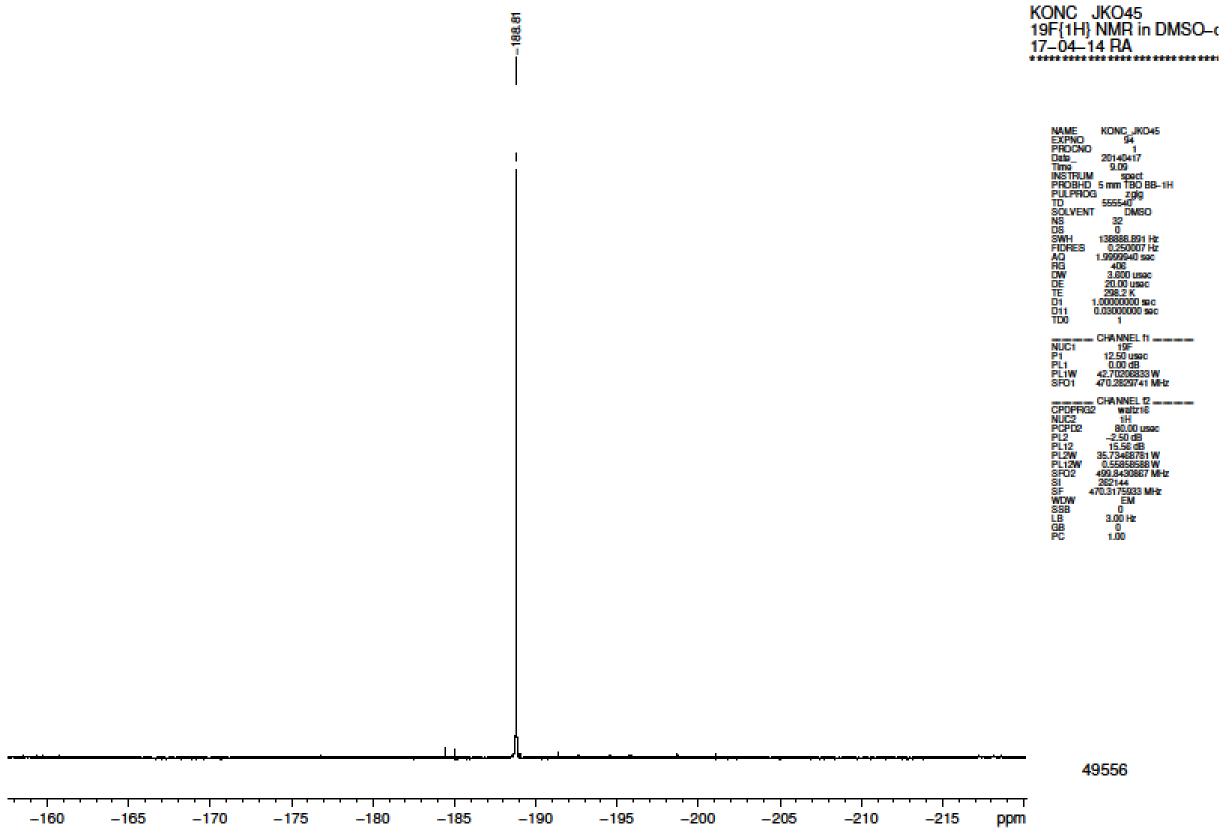
KONC JKO45
19F{1H} NMR in DMSO–
17–04–14 RA
–188.81
49556
–160
–165
–170
–175
–180
–185
–190
–195
–200
–205
–210
–215
ppm

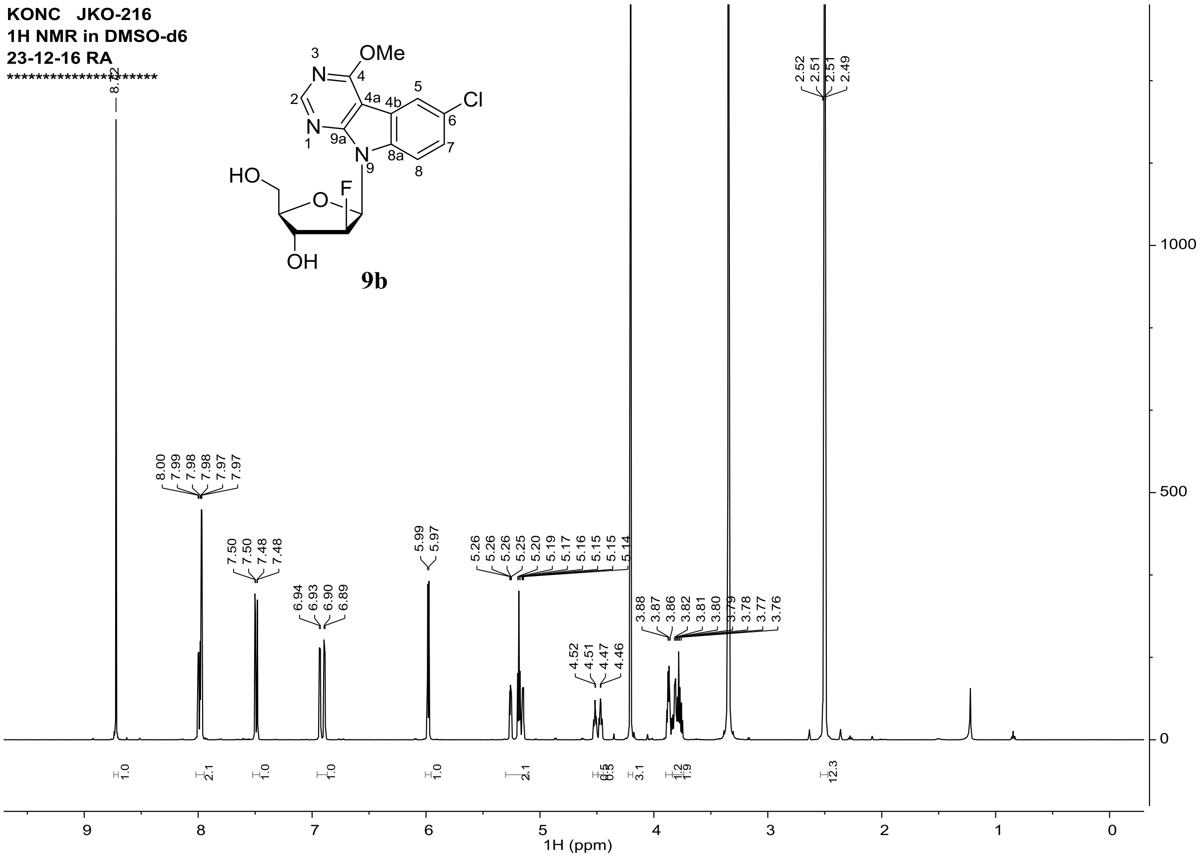
KONC JKO-216
1H NMR in DMSO-d6
23-12-16 RA
****
OMe
Cl
HO
O
F
OH
N
9b
8.72
8.00 7.99 7.98 7.98 7.97 7.97
7.50 7.50 7.48 7.48
6.94 6.93 6.90 6.89
5.99 5.97
5.26 5.26 5.26 5.25 5.20 5.19 5.17 5.16 5.15 5.15 5.14
4.52 4.51 4.47 4.46
3.88 3.87 3.86 3.82 3.81 3.80 3.79 3.78 3.77 3.76
2.52 2.51 2.51 2.49
1.0 2.1 1.0 1.0 1.0 2.1 0.5 0.5 3.1 1.2 1.9 12.3
1000
500
0
9 8 7 6 5 4 3 2 1 0
1H (ppm)

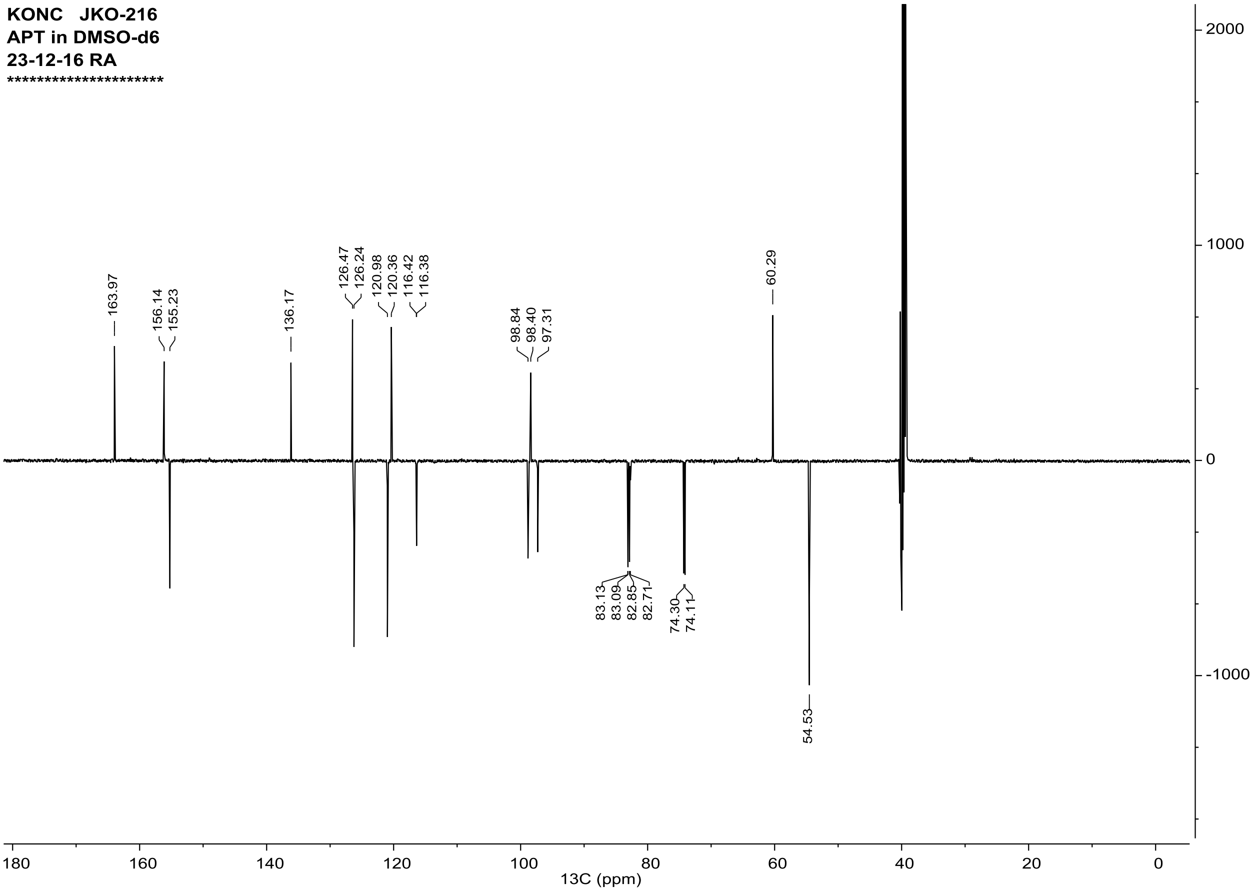
KONC JKO-216
APT in DMSO-d6
23-12-16 RA
****
163.97
156.14 155.23
136.17
126.47 126.24 120.98 120.36 116.42 116.38
98.84 98.40 97.31
83.13 83.09 82.85 82.71
74.30 74.11
60.29
54.53
2000
1000
0
-1000
180 160 140 120 100 80 60 40 20 0
13C (ppm)

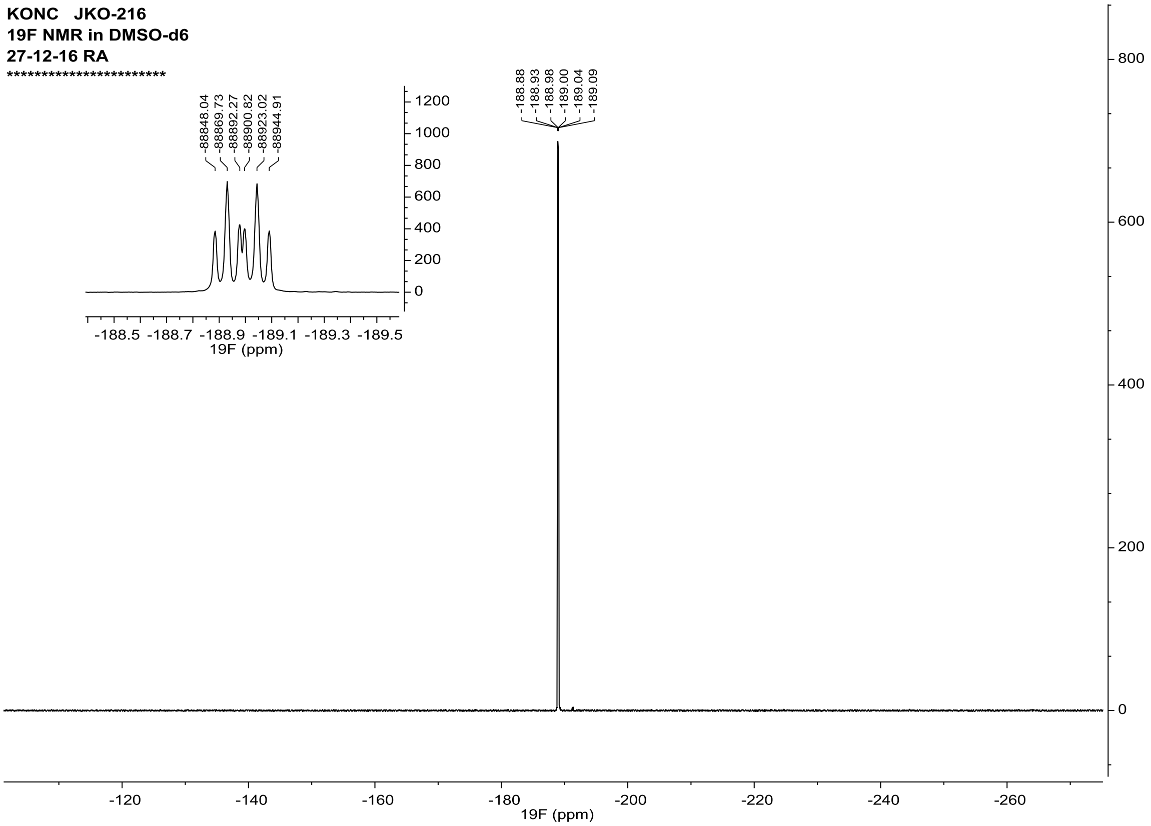
KONC JKO-216
19F NMR in DMSO-d6
27-12-16 RA
19F (ppm)

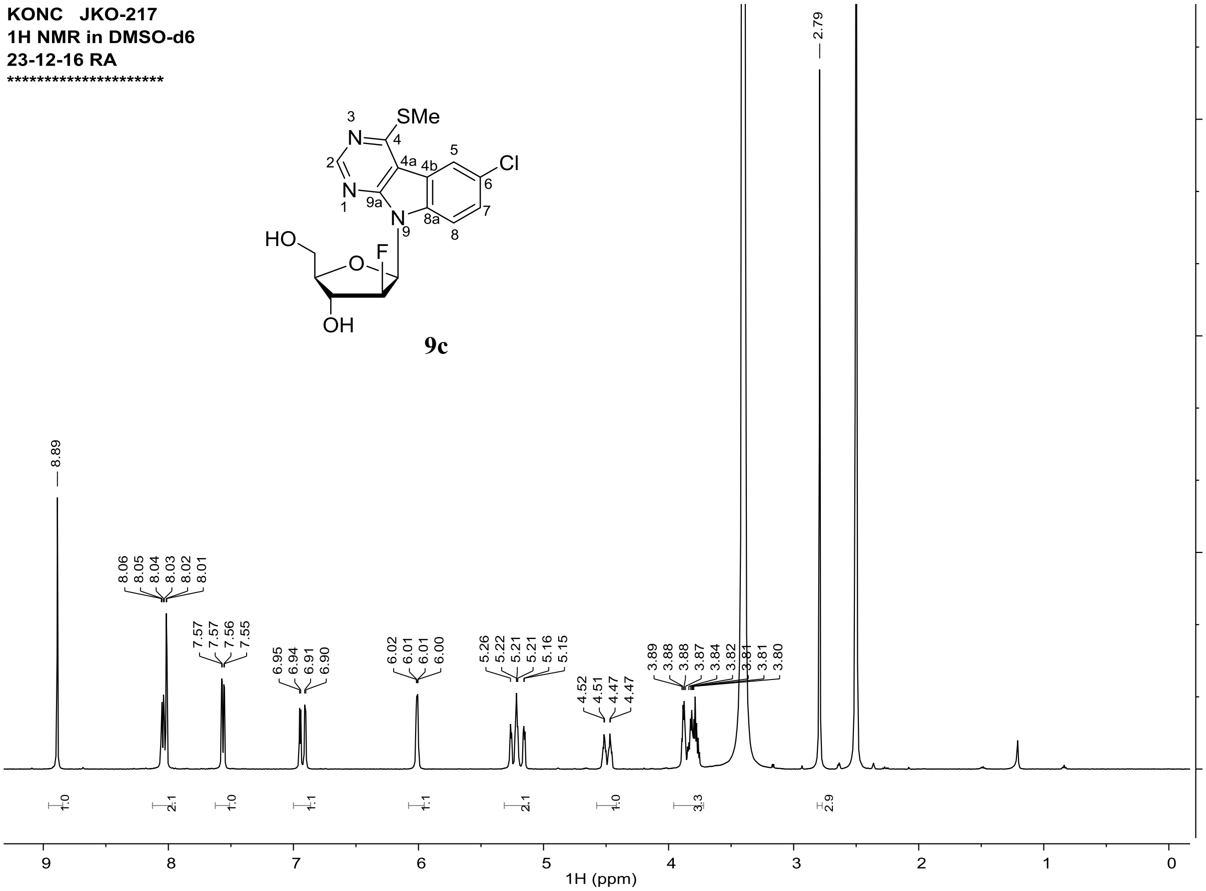
KONC JKO-217
1H NMR in DMSO-d6
23-12-16 RA
9c
1H (ppm)

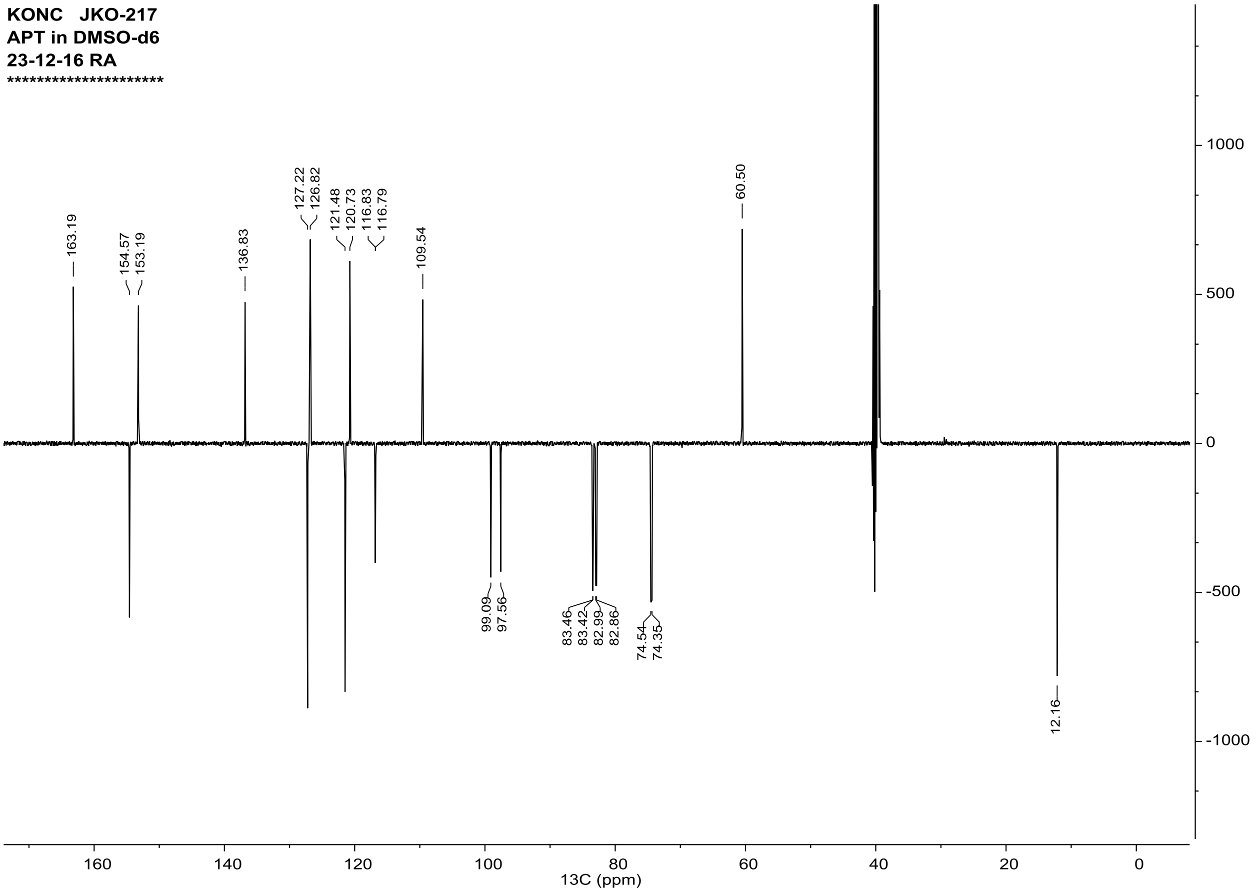
KONC JKO-217
APT in DMSO-d6
23-12-16 RA
****
163.19
154.57
153.19
136.83
127.22
126.82
121.48
120.73
116.83
116.79
109.54
99.09
97.56
83.46
83.42
82.99
82.86
74.54
74.35
60.50
12.16
13C (ppm)

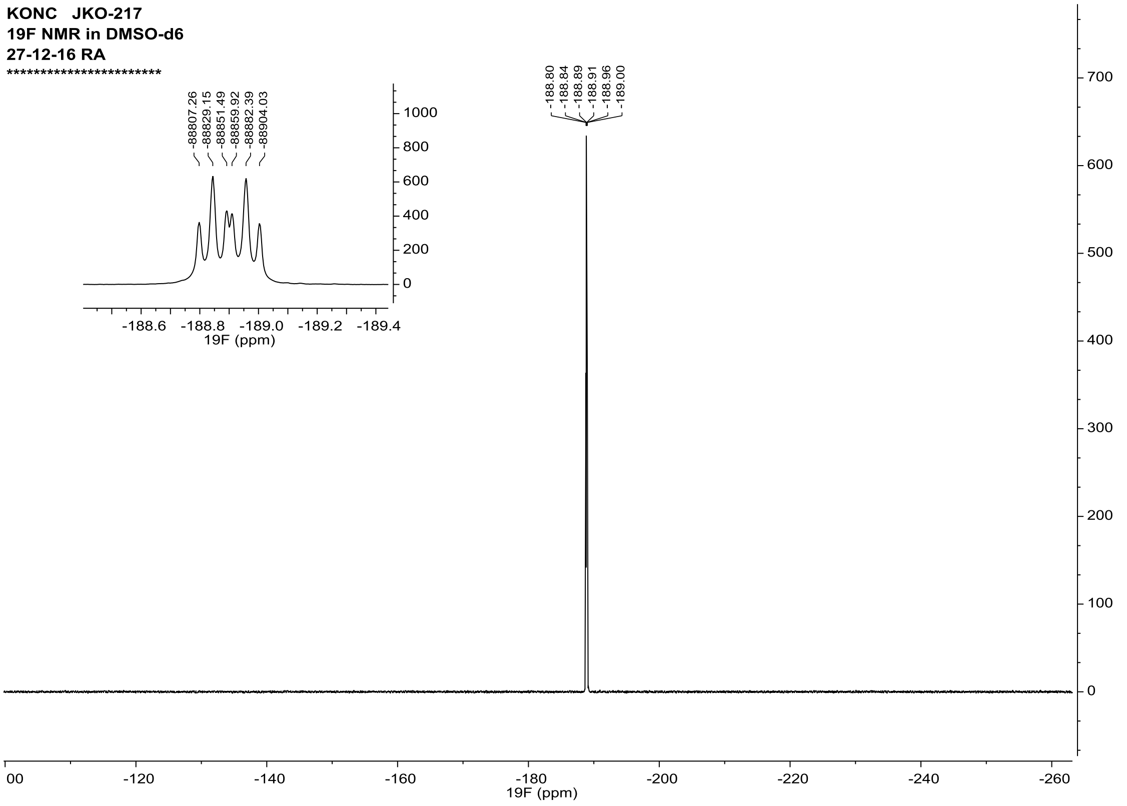
KONC JKO-217
19F NMR in DMSO-d6
27-12-16 RA
****
-88807.26
-88829.15
-88851.49
-88859.92
-88882.39
-88904.03
19F (ppm)
-188.80
-188.84
-188.89
-188.91
-188.96
-189.00
19F (ppm)

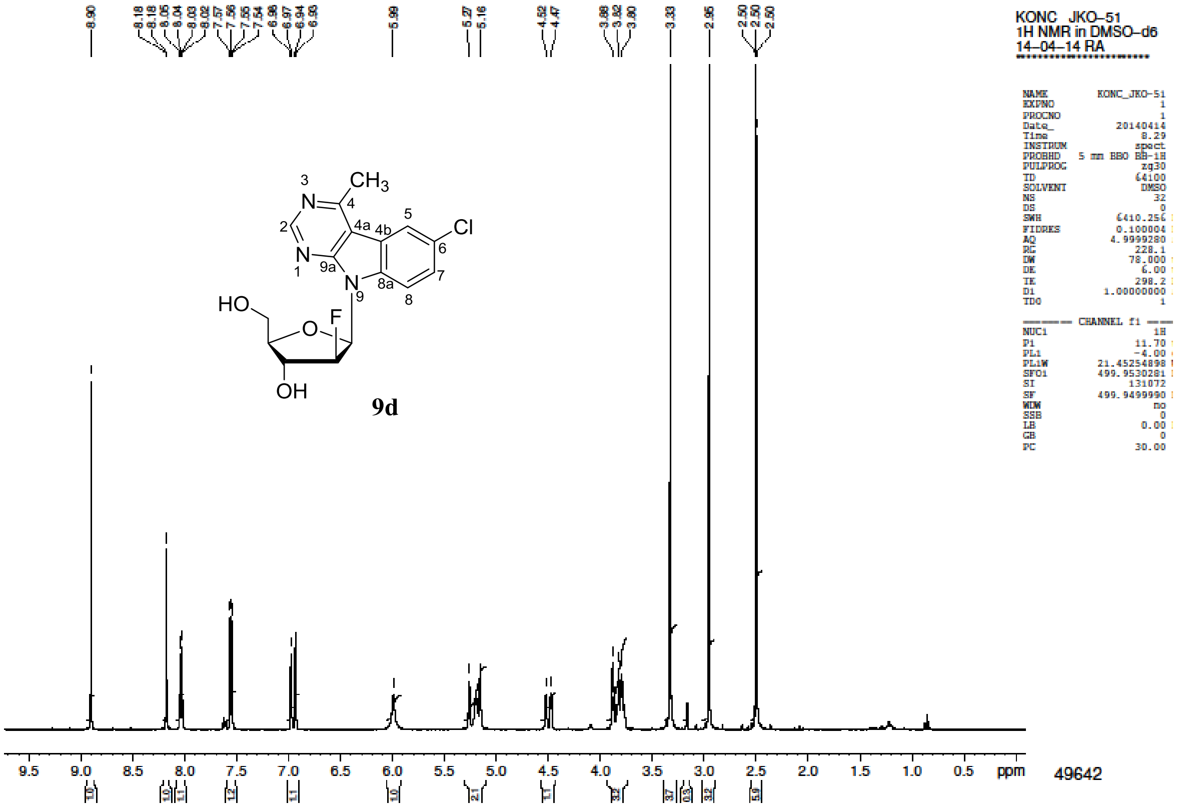
KONC_JKO-51
1H NMR in DMSO-d6
14-04-14 RA
8.90
8.18 8.18 8.05 8.04 8.03 8.02 7.57 7.56 7.55 7.54 6.98 6.97 6.94 6.93
5.99
5.27 5.16
4.52 4.47
3.88 3.82 3.80
3.33
2.95
2.50 2.50 2.50
CH3
Cl
HO
F
OH
9d
9.5 9.0 8.5 8.0 7.5 7.0 6.5 6.0 5.5 5.0 4.5 4.0 3.5 3.0 2.5 2.0 1.5 1.0 0.5 ppm
49642
NAME KONC_JKO-51
EXPNO 1
PROCNO 1
Date_ 20140414
Time 8.29
INSTRUM spect
PROBHD 5 mm BBO BB-1H
PULPROG zg30
TD 64100
SOLVENT DMSO
NS 32
DS 0
SWH 6410.256
FIDRES 0.100004
AQ 4.9999280
RG 228.1
DW 78.000
DE 6.00
TE 298.2
D1 1.00000000
TD0 1
CHANNEL f1
NUC1 1H
P1 11.70
PL1 -4.00
PL1W 21.45254898
SFO1 499.9530281
SI 131072
SF 499.9499990
WDW no
SSB 0
LB 0.00
GB 0
PC 30.00

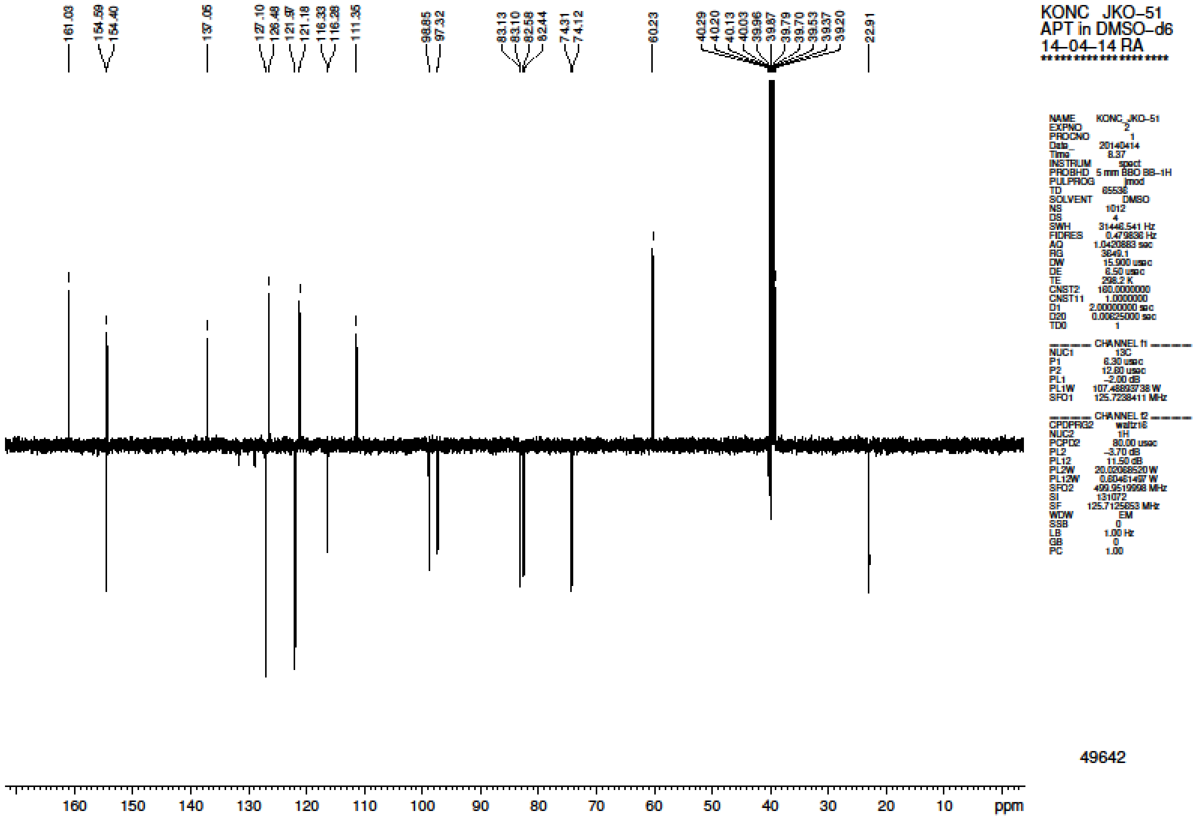
KONC_JKO-51
APT in DMSO-d6
14-04-14 RA
161.03
154.59 154.40
137.05
127.10 126.48 121.97 121.18 116.33 116.28 111.35
98.85 97.32
83.13 83.10 82.58 82.44 74.31 74.12
60.23
40.29 40.20 40.13 40.03 39.96 39.87 39.79 39.70 39.53 39.37 39.20
22.91
160 150 140 130 120 110 100 90 80 70 60 50 40 30 20 10 ppm
49642

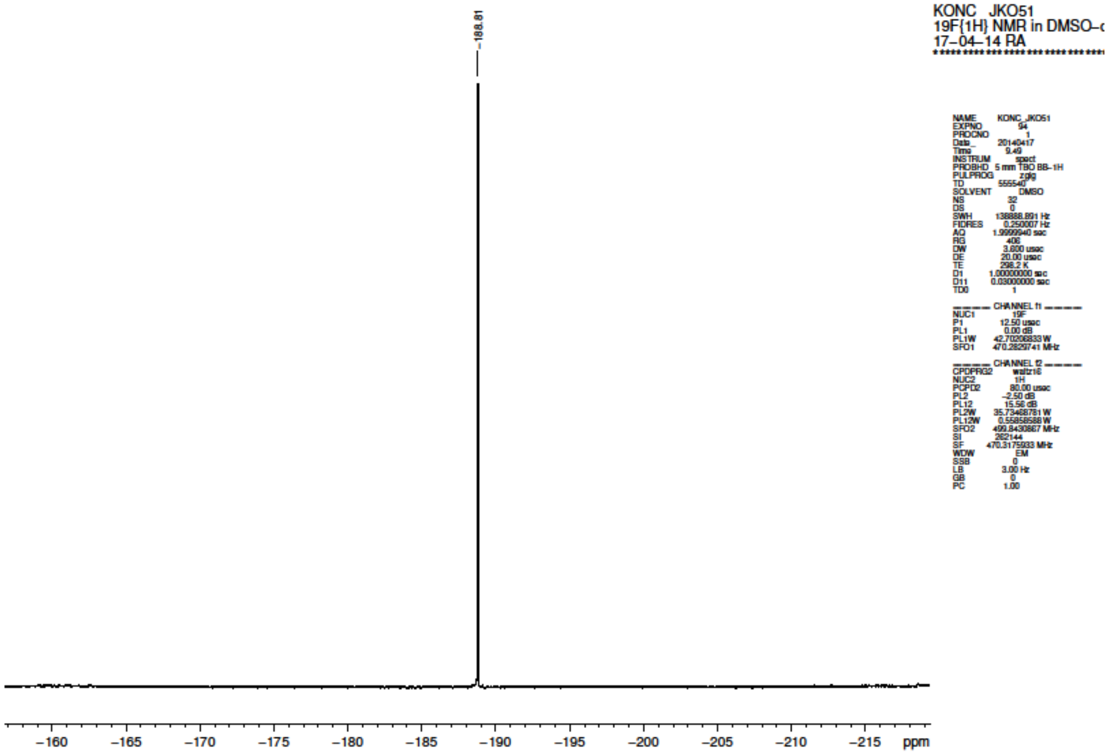
KONC JKO51
19F{1H} NMR in DMSO-d
17-04-14 RA
-188.81
ppm

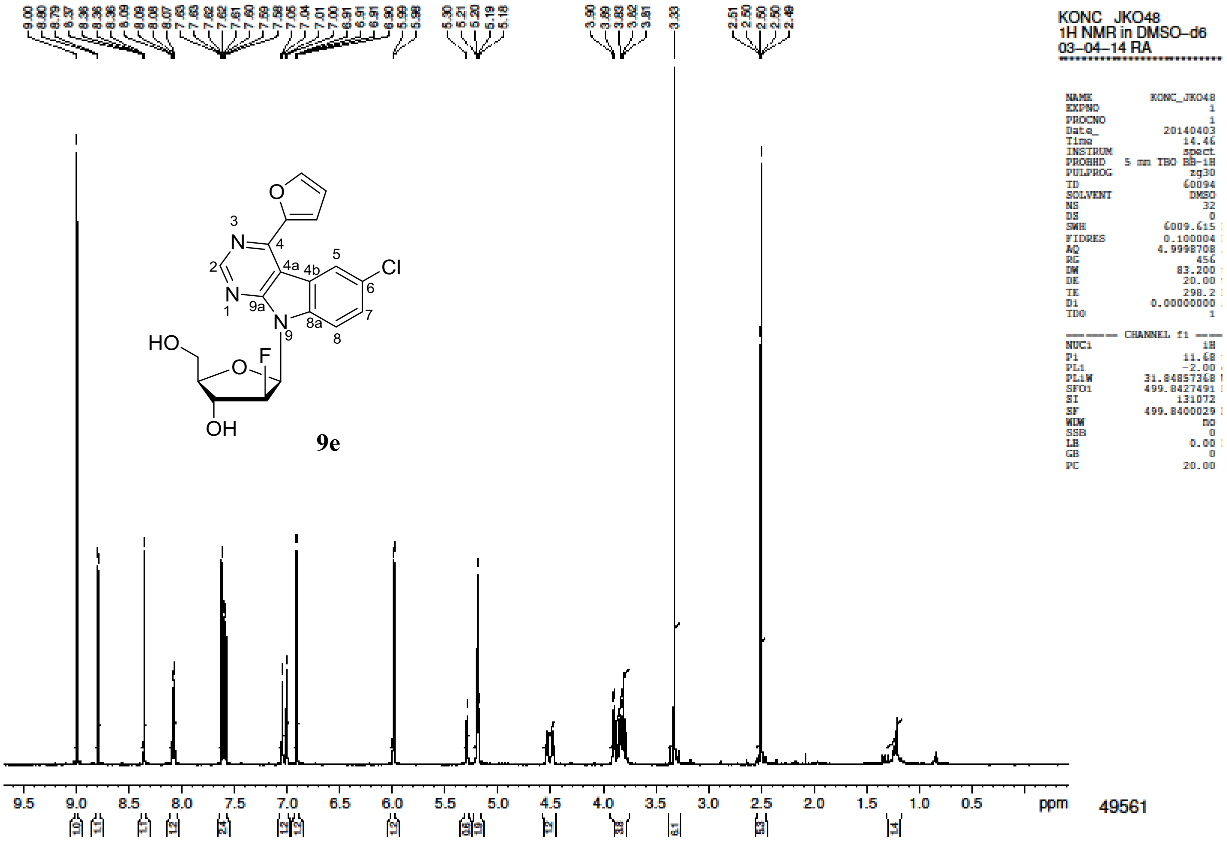
KONC JKO48
1H NMR in DMSO-d6
03-04-14 RA
9e
ppm
49561

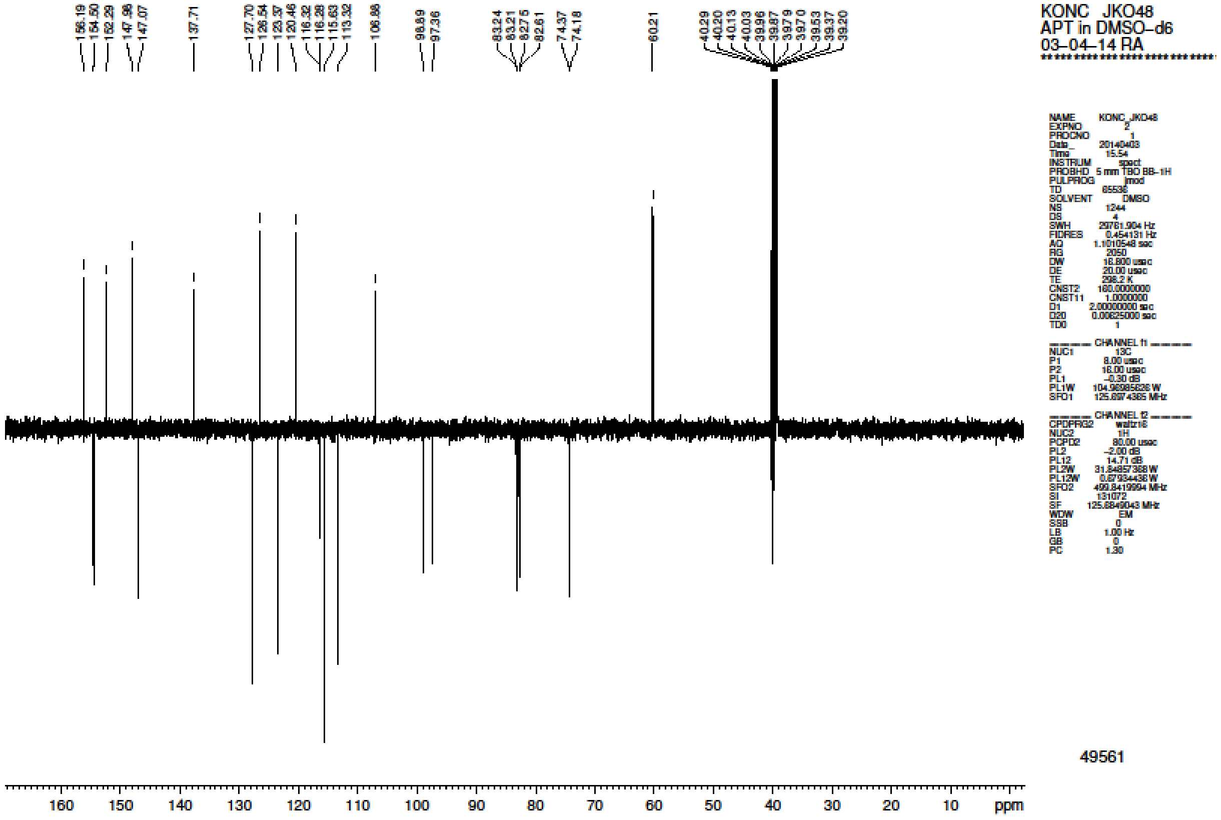
KONC JKO48
APT in DMSO–d6
03–04–14 RA
156.19
154.50
152.29
147.98
147.07
137.71
127.70
126.54
123.37
120.46
116.32
116.28
115.63
113.32
106.88
98.89
97.36
83.24
83.21
82.75
82.61
74.37
74.18
60.21
40.29
40.20
40.13
40.03
39.96
39.87
39.79
39.70
39.53
39.37
39.20
49561
160
150
140
130
120
110
100
90
80
70
60
50
40
30
20
10
ppm

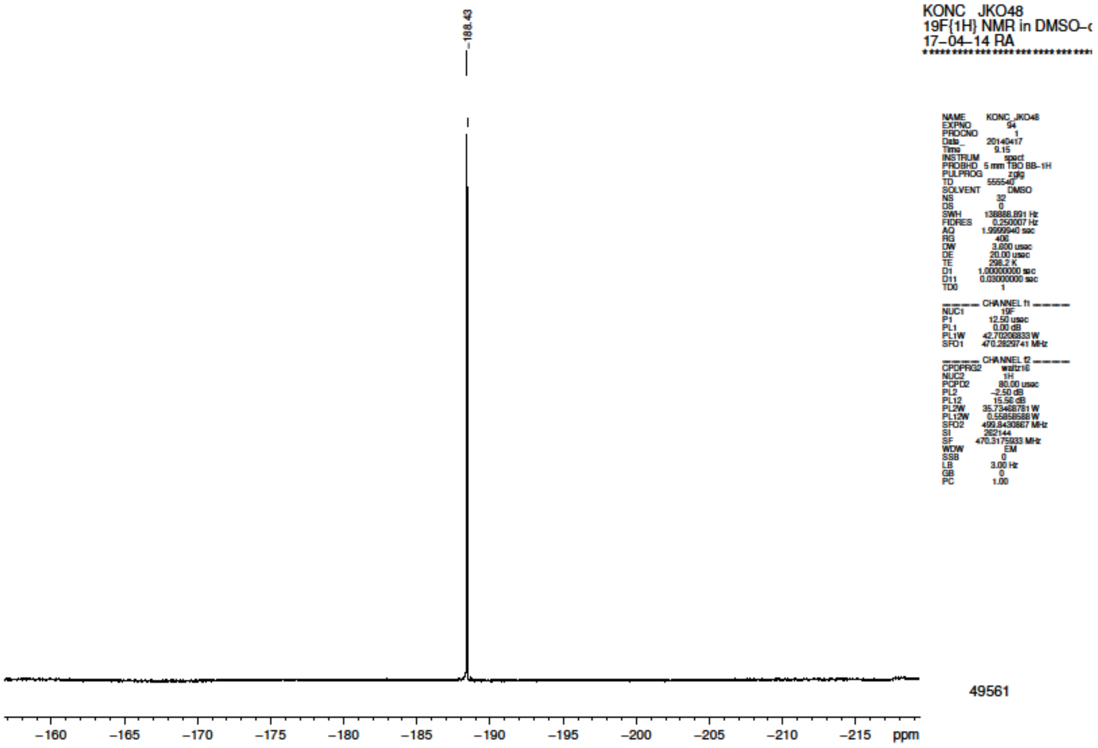
KONC JKO48
19F{1H} NMR in DMSO–d
17–04–14 RA
–188.43
49561
–160
–165
–170
–175
–180
–185
–190
–195
–200
–205
–210
–215
ppm

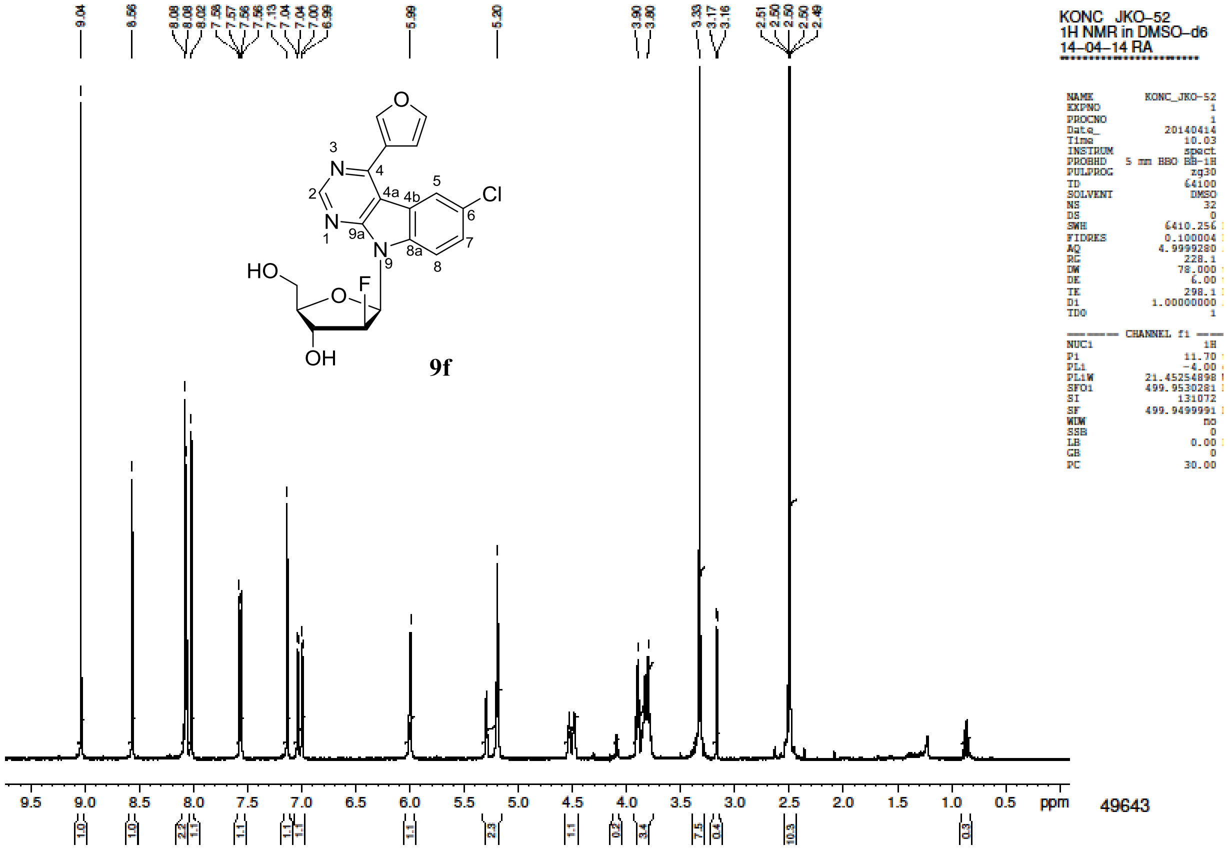
KONC JKO-52
1H NMR in DMSO-d6
14-04-14 RA
9f
49643

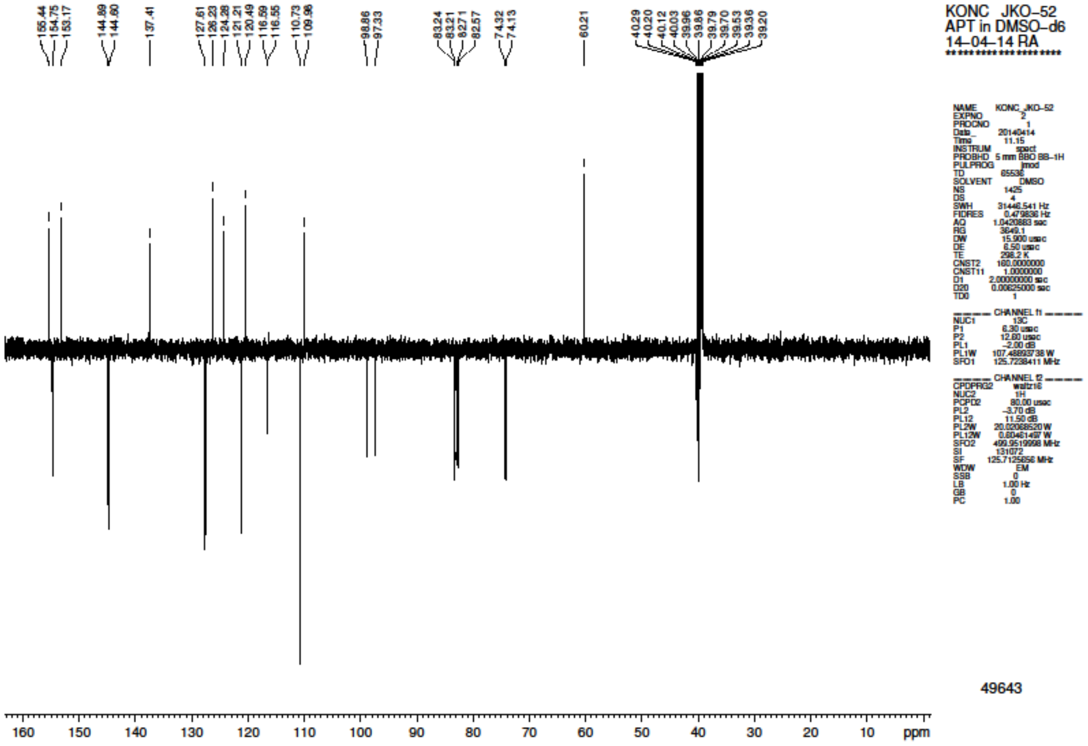
KONC JKO-52
APT in DMSO-d6
14-04-14 RA
49643

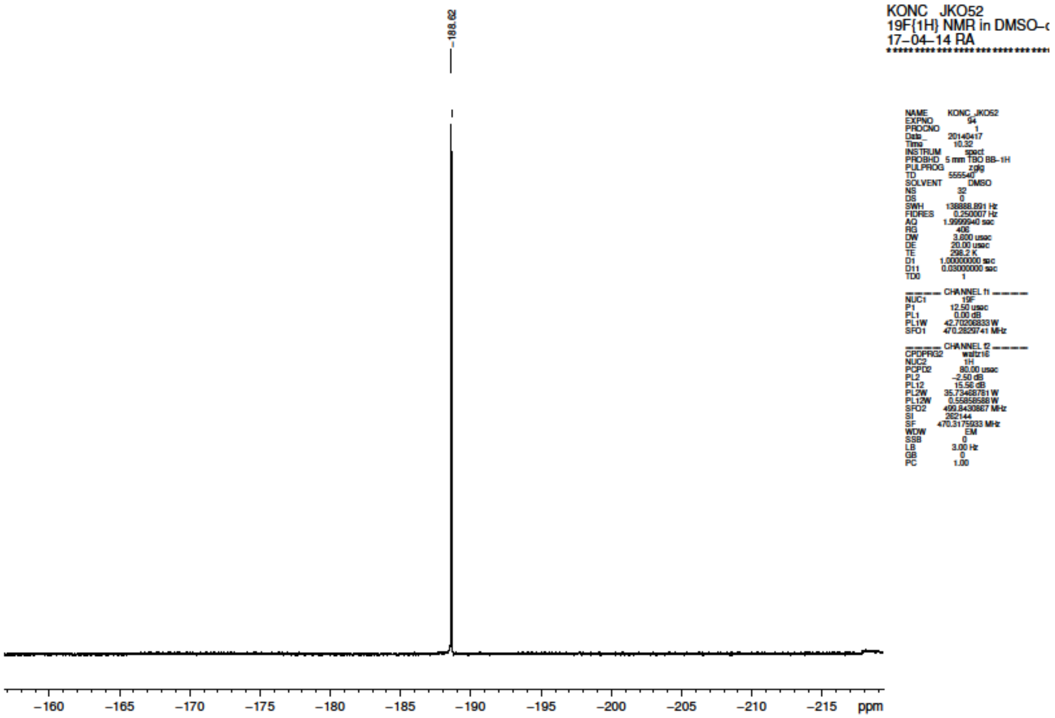
KONC JKO52
19F{1H} NMR in DMSO-d
17-04-14 RA
-188.62
ppm

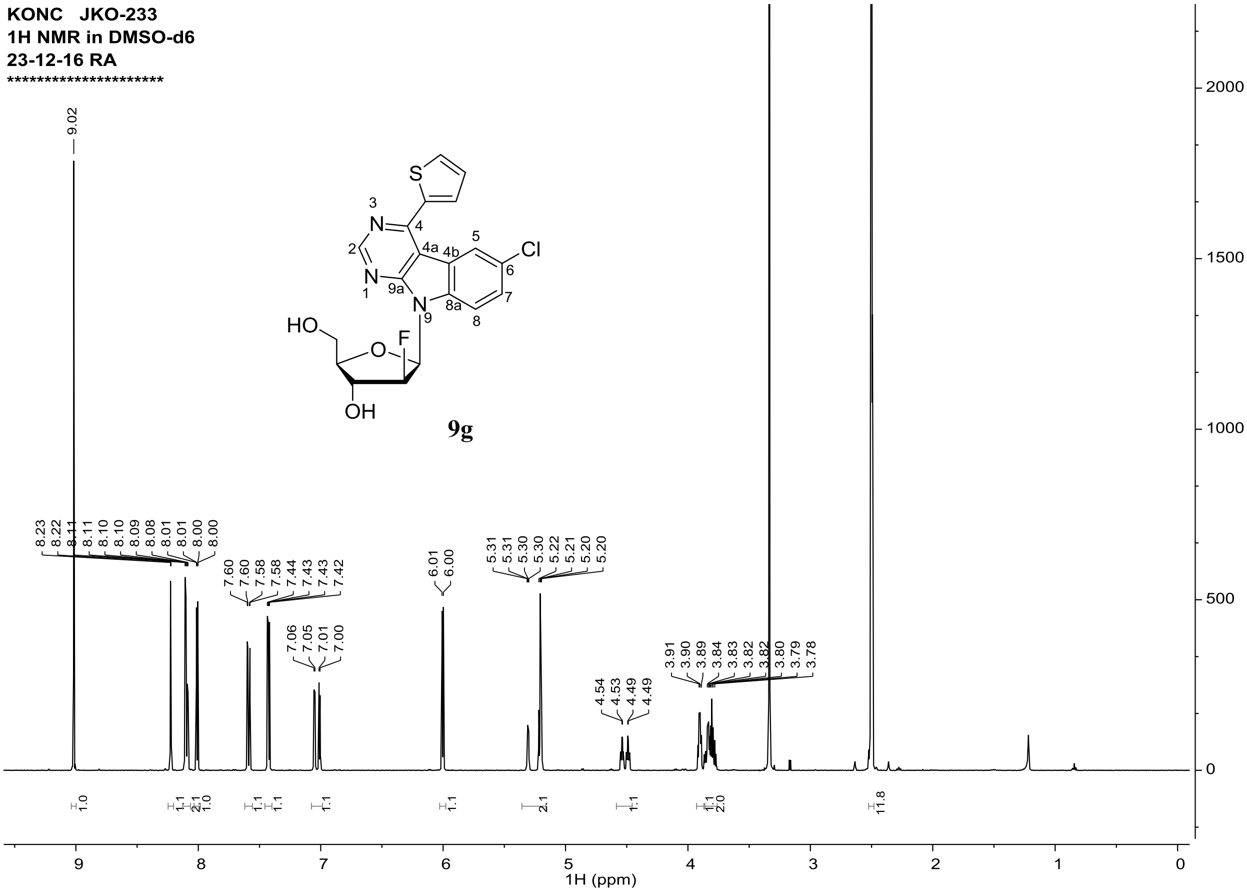
KONC JKO-233
1H NMR in DMSO-d6
23-12-16 RA
9g
1H (ppm)

KONC JKO-233
APT in DMSO-d6
23-12-16 RA
****

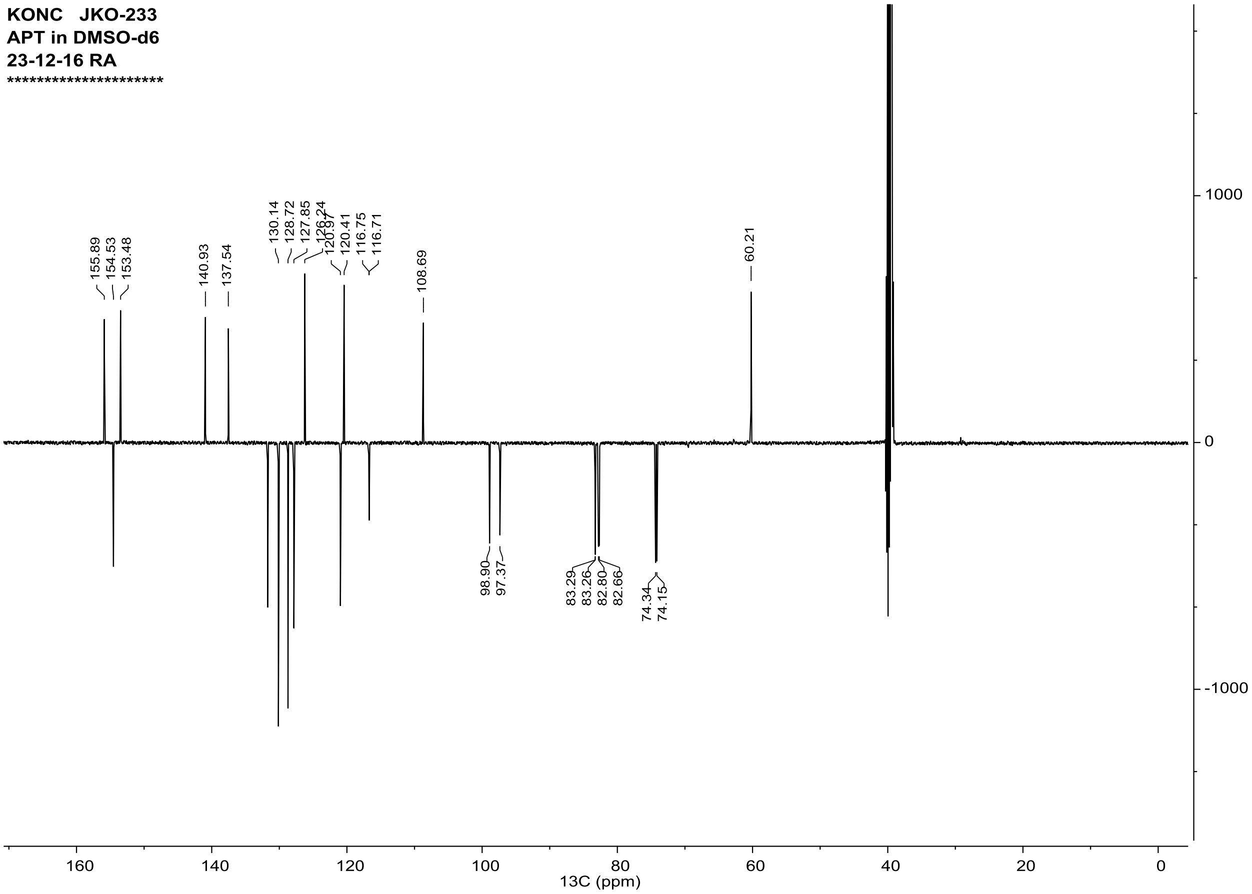

KONC JKO-233
19F NMR in DMSO-d6
27-12-16 RA
****

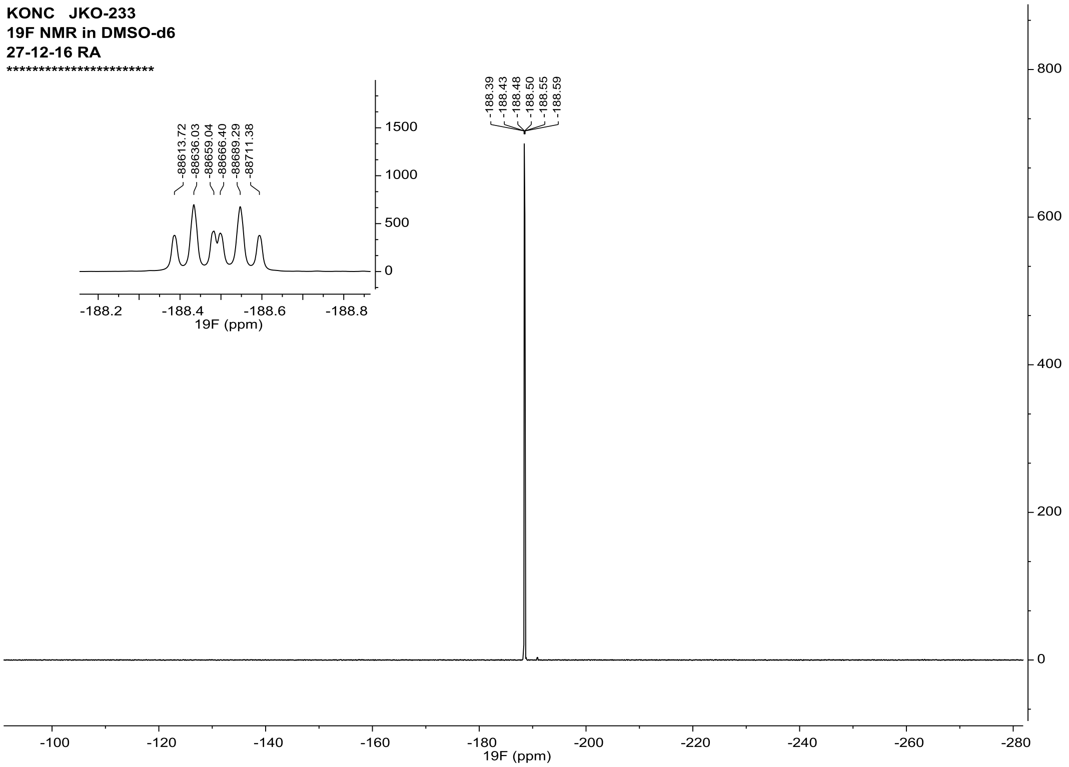

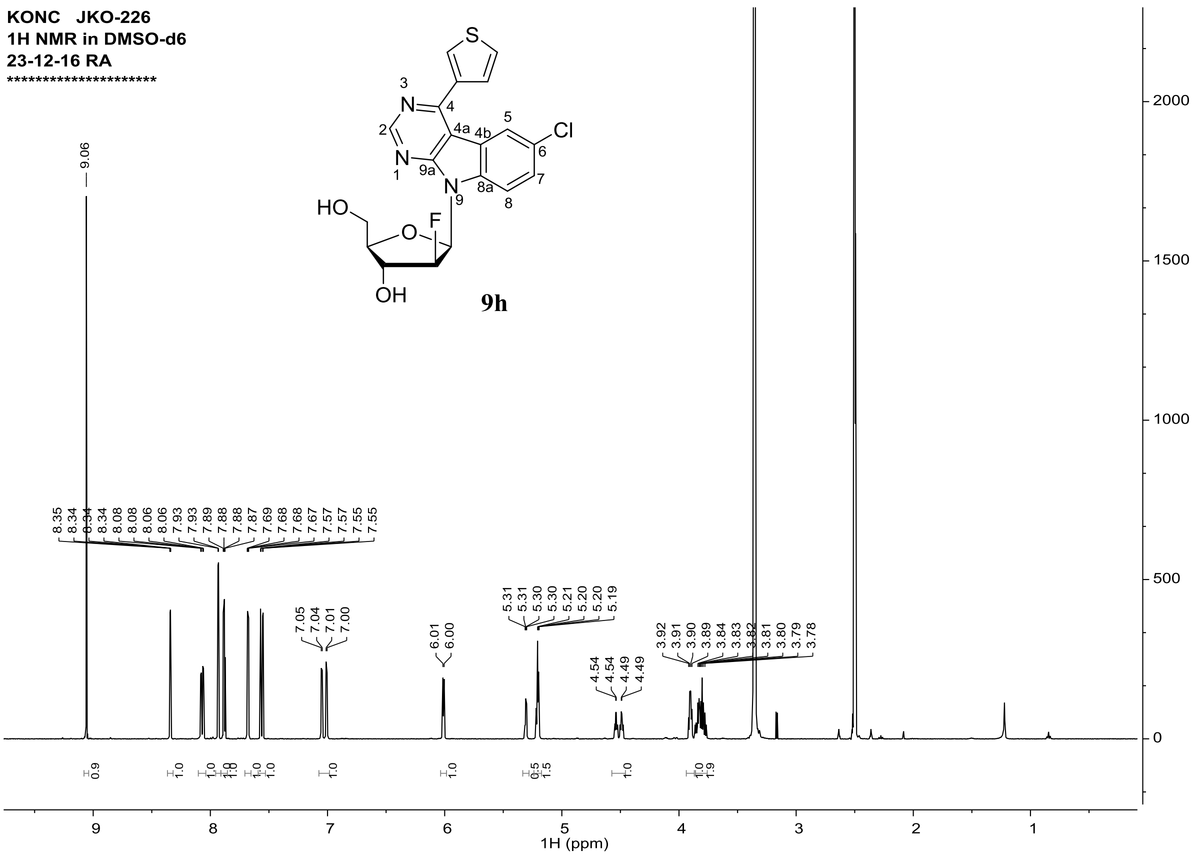
KONC JKO-226
1H NMR in DMSO-d6
23-12-16 RA
****
9h
1H (ppm)

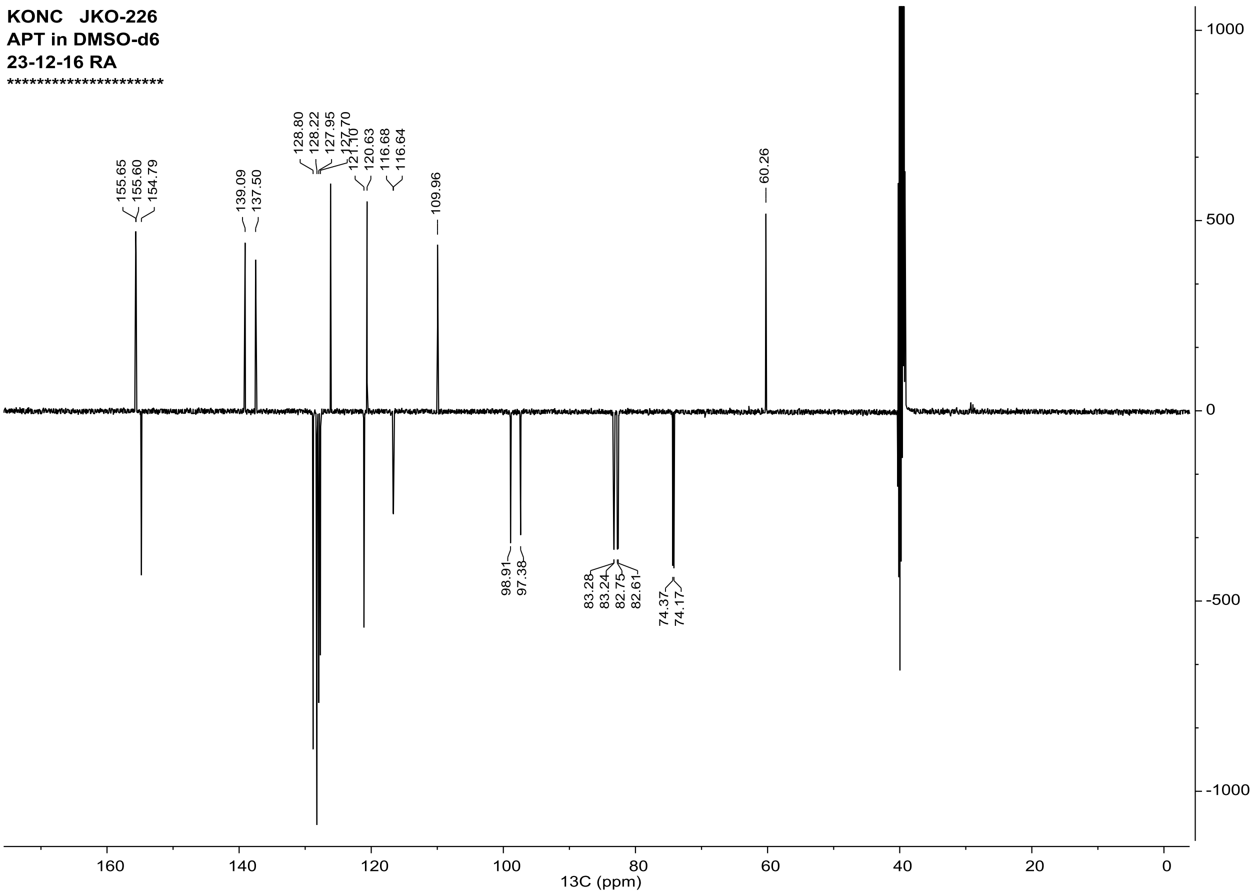
KONC JKO-226
APT in DMSO-d6
23-12-16 RA
****
13C (ppm)

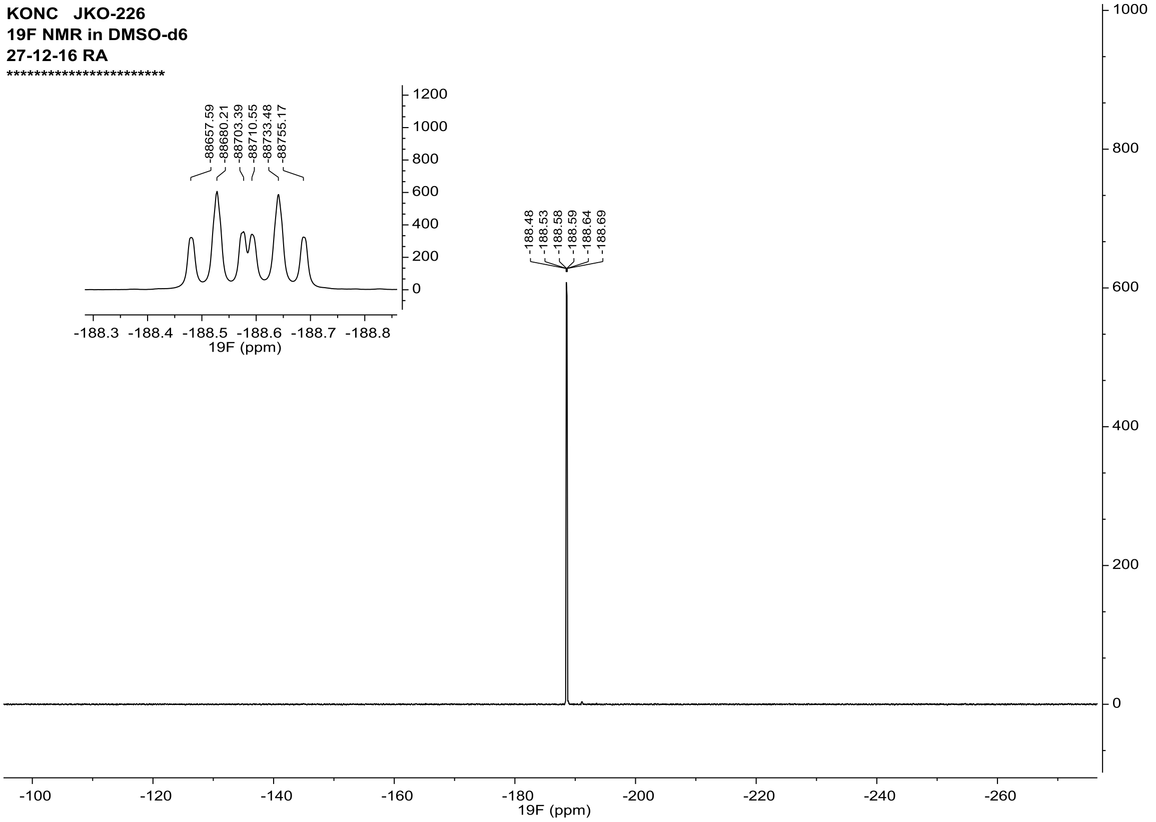
KONC JKO-226
19F NMR in DMSO-d6
27-12-16 RA
-188.48
-188.53
-188.58
-188.59
-188.64
-188.69
-88657.59
-88680.21
-88703.39
-88710.55
-88733.48
-88755.17
19F (ppm)
19F (ppm)

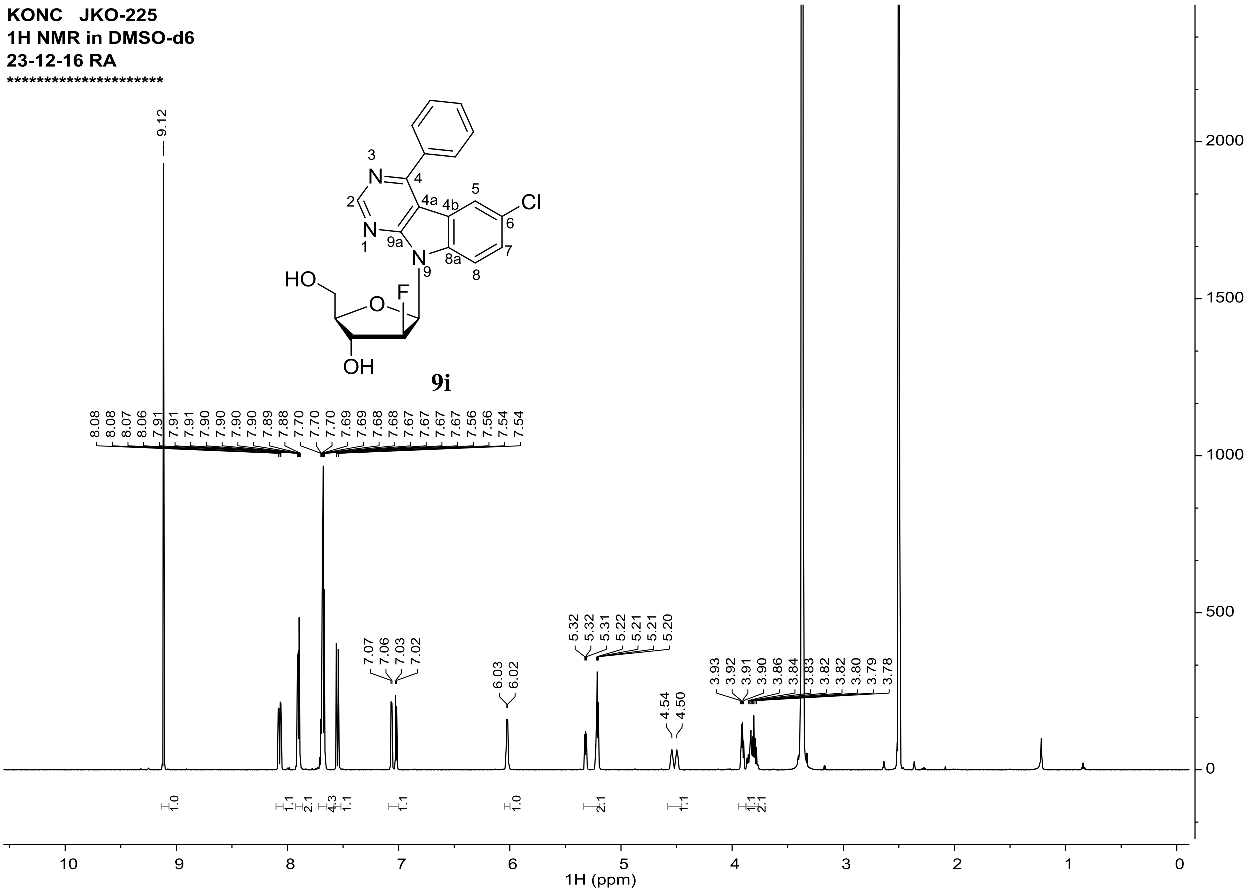
KONC JKO-225
1H NMR in DMSO-d6
23-12-16 RA
9.12
HO
O
F
N
N
N
OH
Cl
9i
1H (ppm)

KONC JKO-225
APT in DMSO-d6
23-12-16 RA
****

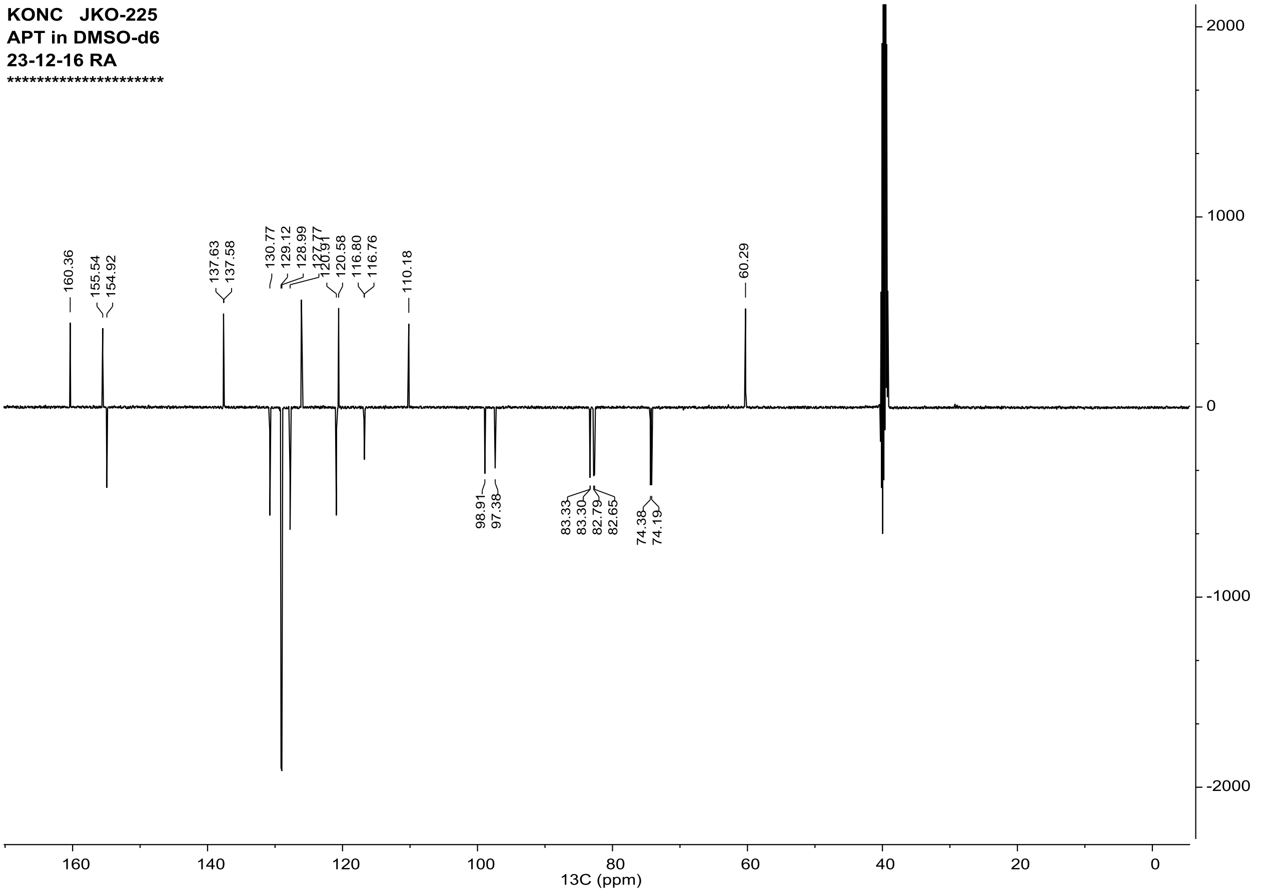

KONC JKO-225
19F NMR in DMSO-d6
27-12-16 RA
****

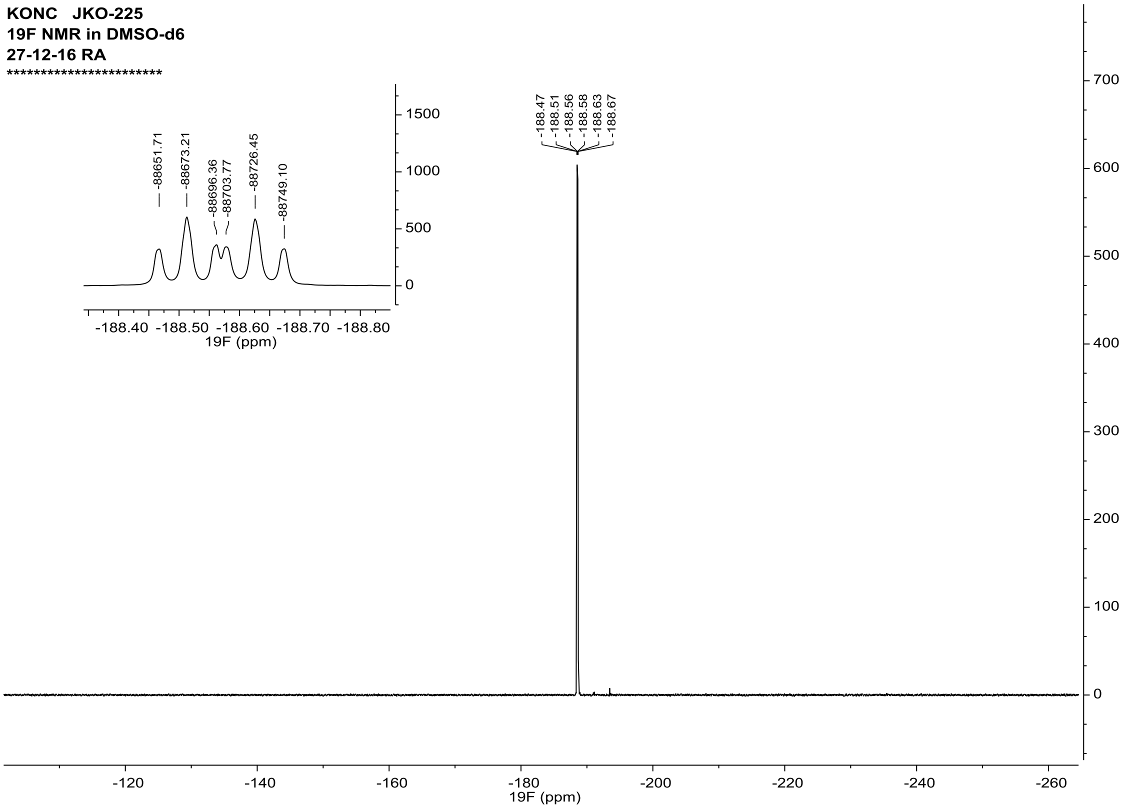

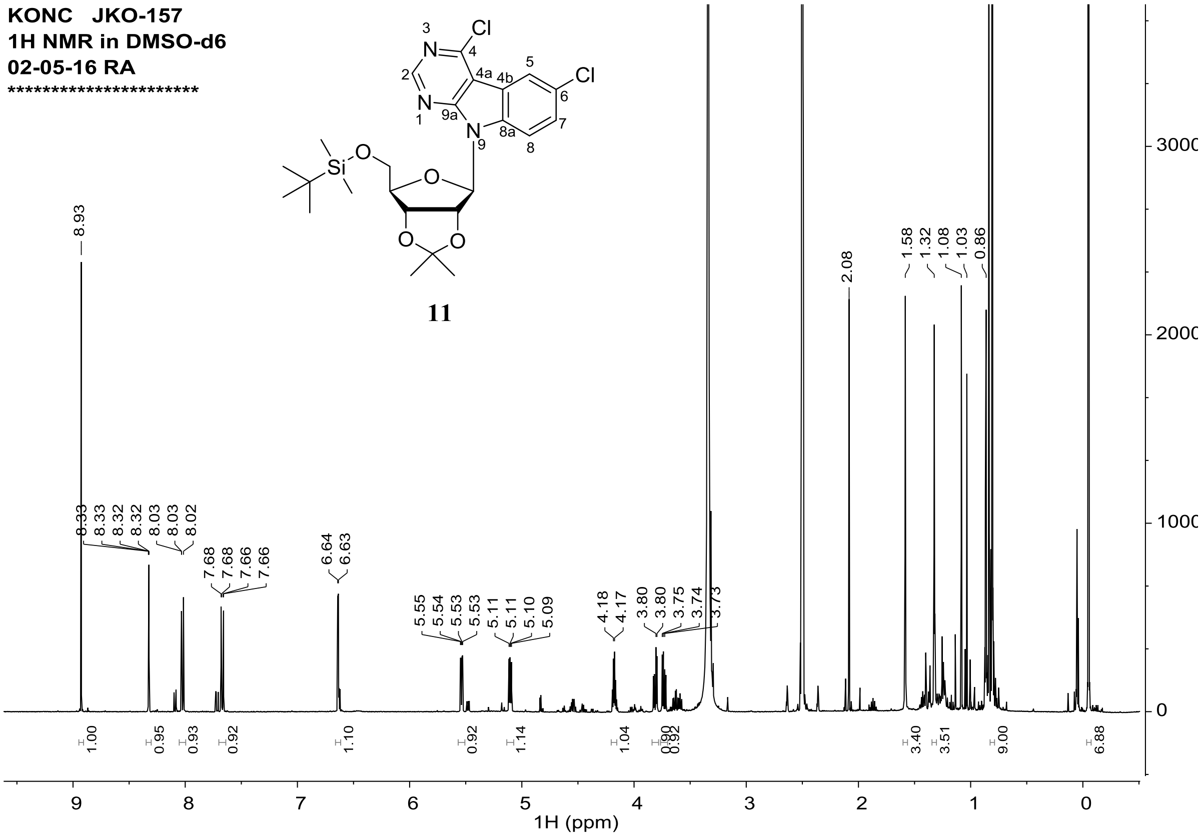
KONC JKO-157
1H NMR in DMSO-d6
02-05-16 RA
****
11
8.93
8.33 8.33 8.32 8.32 8.03 8.03 8.02
7.68 7.68 7.66 7.66
6.64 6.63
5.55 5.54 5.53 5.53 5.11 5.11 5.10 5.09
4.18 4.17 3.80 3.80 3.75 3.74 3.73
2.08
1.58 1.32 1.08 1.03 0.86
1.00 0.95 0.93 0.92 1.10 0.92 1.14 1.04 0.90 0.92 3.40 3.51 9.00 6.88
1H (ppm)

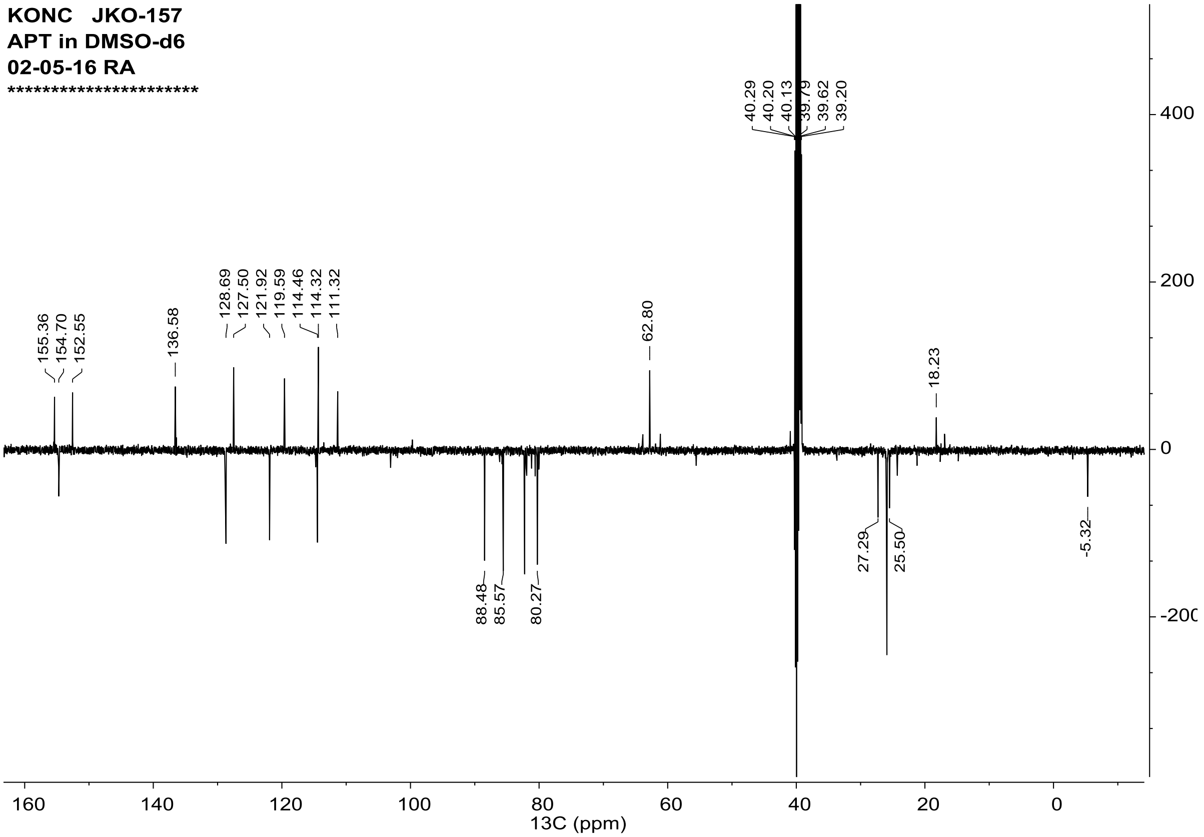
KONC JKO-157
APT in DMSO-d6
02-05-16 RA
****
155.36 154.70 152.55
136.58
128.69 127.50 121.92 119.59 114.46 114.32 111.32
62.80
40.29 40.20 40.13 39.79 39.62 39.20
18.23
88.48 85.57 80.27
27.29 25.50
-5.32
13C (ppm)

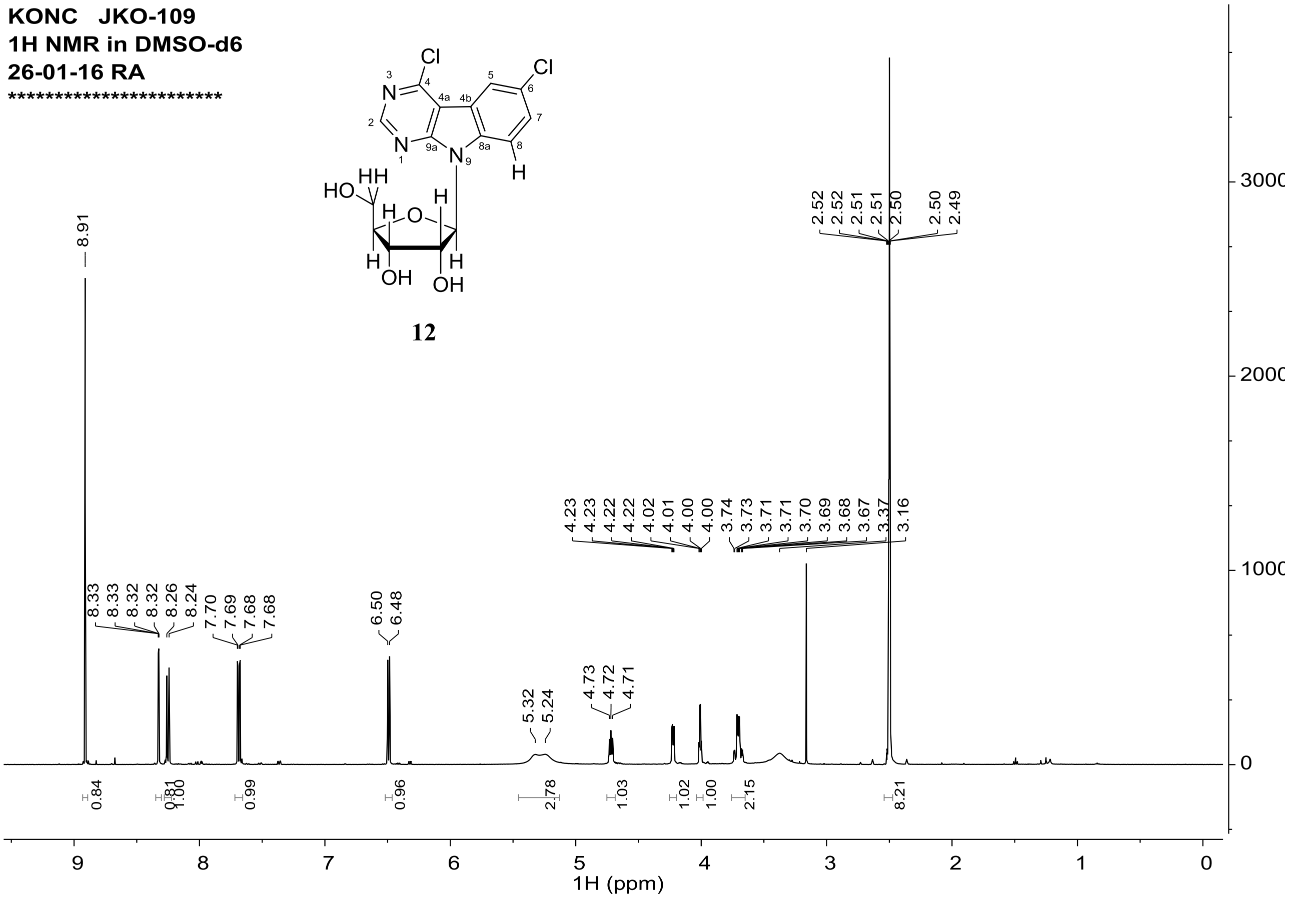
KONC JKO-109
1H NMR in DMSO-d6
26-01-16 RA
****
12
8.91
8.33 8.33 8.32 8.32 8.26 8.24
7.70 7.69 7.68 7.68
6.50 6.48
5.32 5.24
4.73 4.72 4.71
4.23 4.23 4.22 4.22 4.02 4.01 4.00 4.00 3.74 3.73 3.71 3.71 3.70 3.69 3.68 3.67 3.37 3.16
2.52 2.52 2.51 2.51 2.50 2.50 2.49
0.84 0.81 1.00 0.99 0.96 2.78 1.03 1.02 1.00 2.15 8.21
1H (ppm)

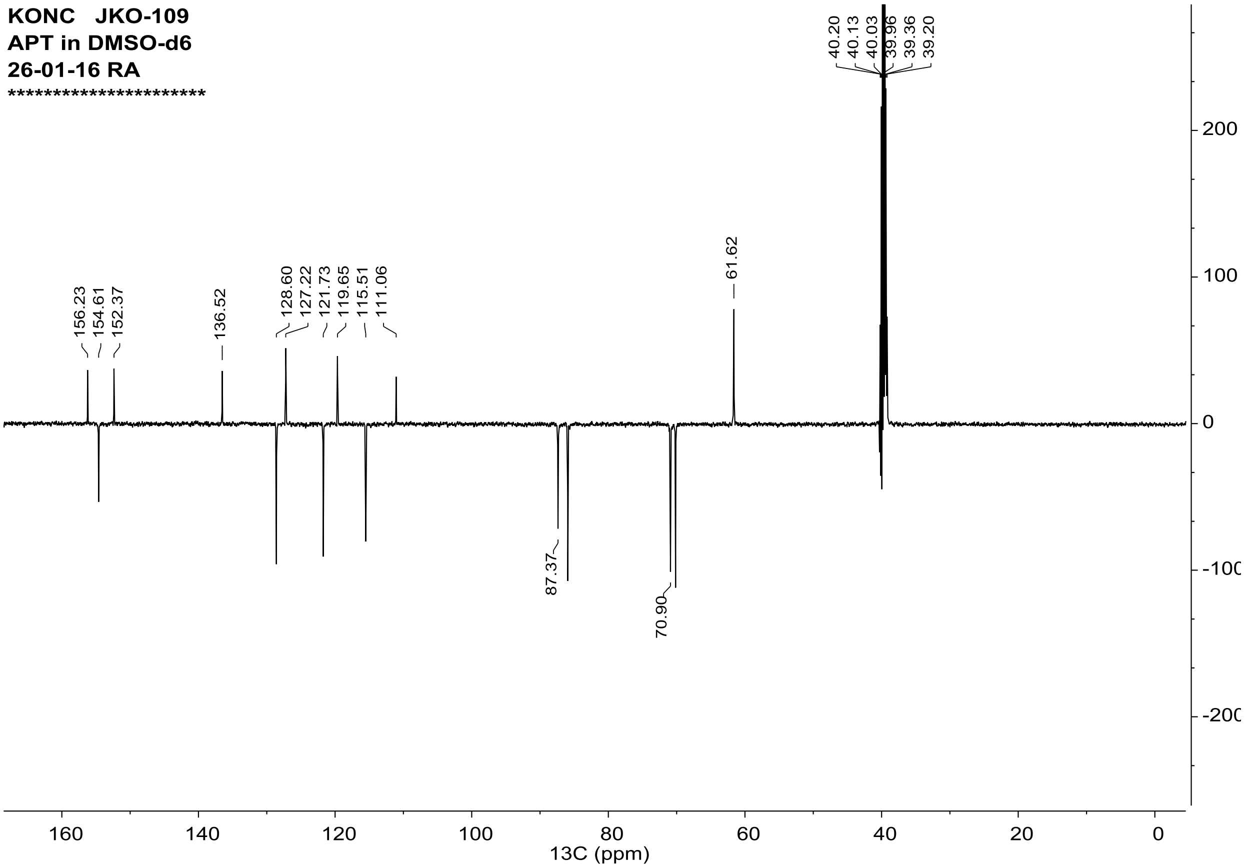
KONC JKO-109
APT in DMSO-d6
26-01-16 RA
****
156.23 154.61 152.37
136.52
128.60 127.22 121.73 119.65 115.51 111.06
87.37
70.90
61.62
40.20 40.13 40.03 39.96 39.36 39.20
13C (ppm)

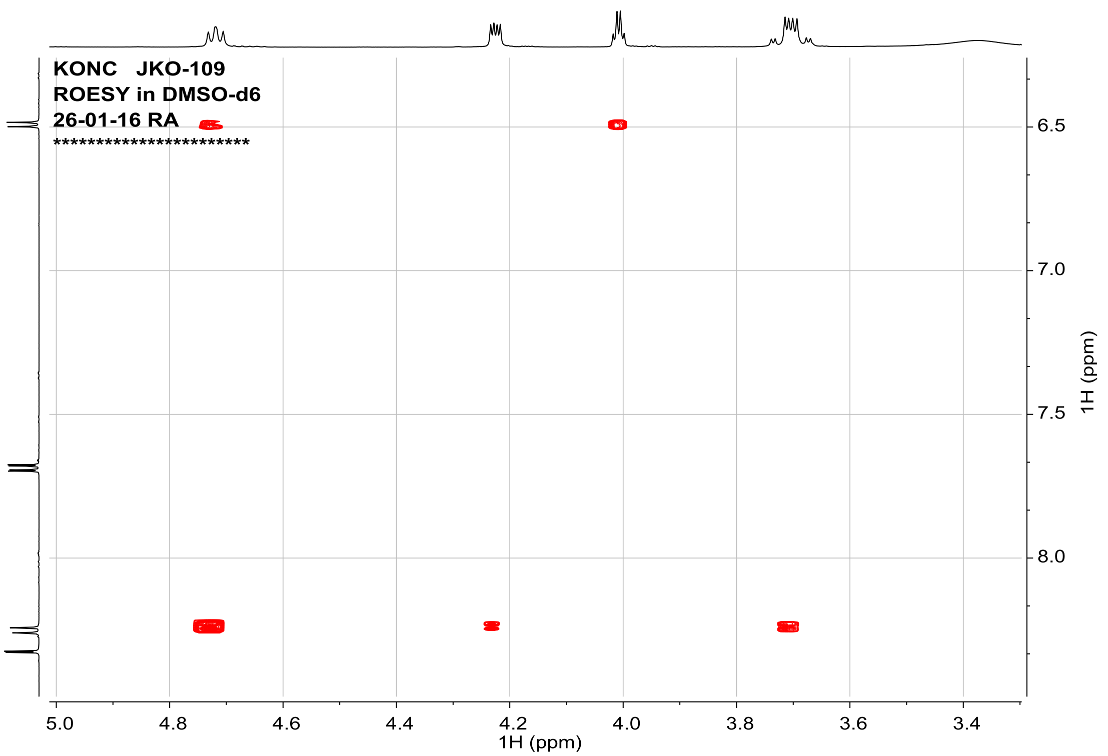
KONC JKO-109
ROESY in DMSO-d6
26-01-16 RA
1H (ppm)
1H (ppm)
5.0 4.8 4.6 4.4 4.2 4.0 3.8 3.6 3.4
6.5 7.0 7.5 8.0

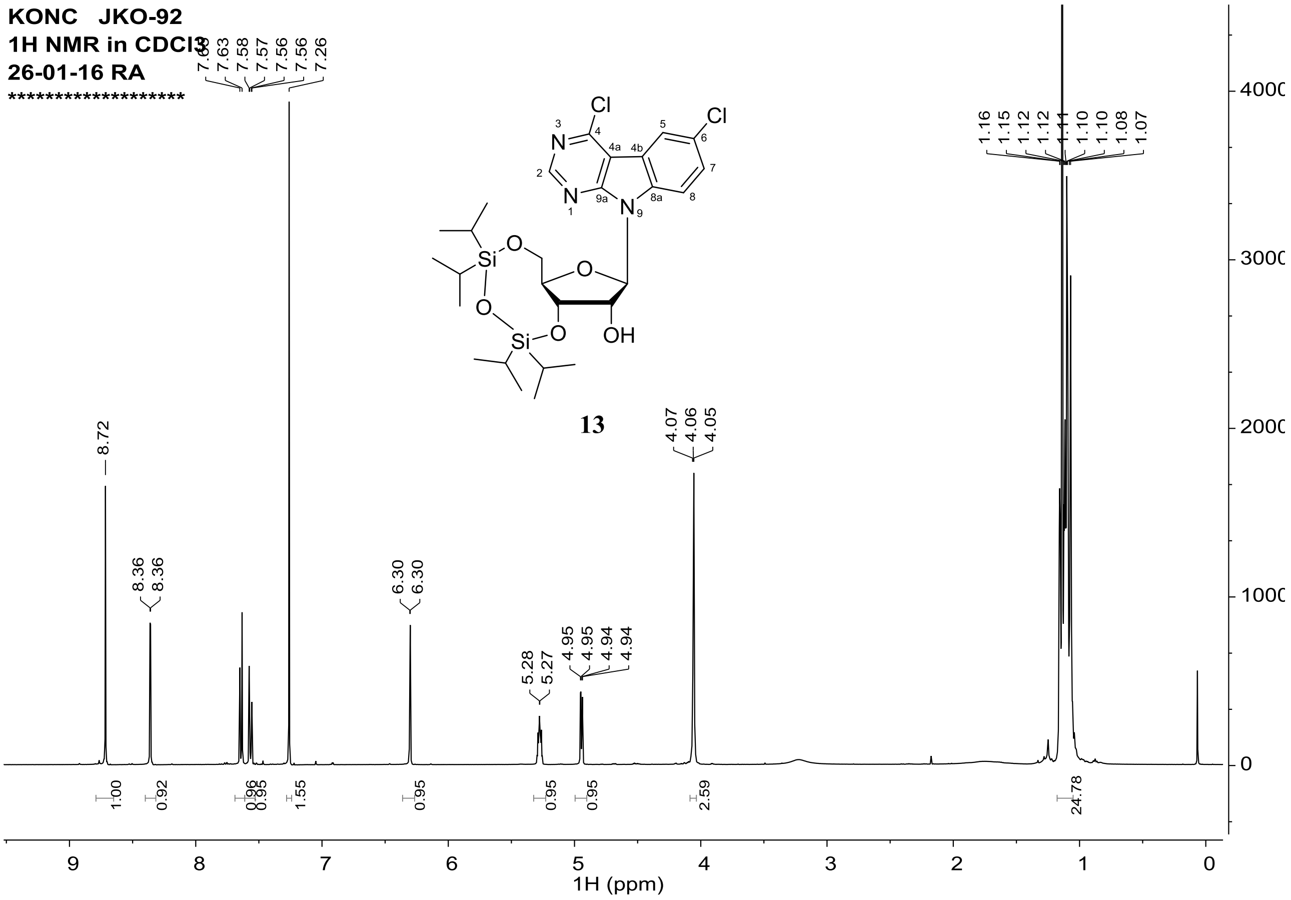
KONC JKO-92
1H NMR in CDCl3
26-01-16 RA
8.72
8.36 8.36
7.63 7.58 7.57 7.56 7.56 7.26
6.30 6.30
5.28 5.27
4.95 4.95 4.94 4.94
4.07 4.06 4.05
1.16 1.15 1.12 1.12 1.11 1.10 1.10 1.08 1.07
1.00 0.92 0.96 0.95 1.55 0.95 0.95 0.95 2.59 24.78
13
1H (ppm)
9 8 7 6 5 4 3 2 1 0
4000 3000 2000 1000 0

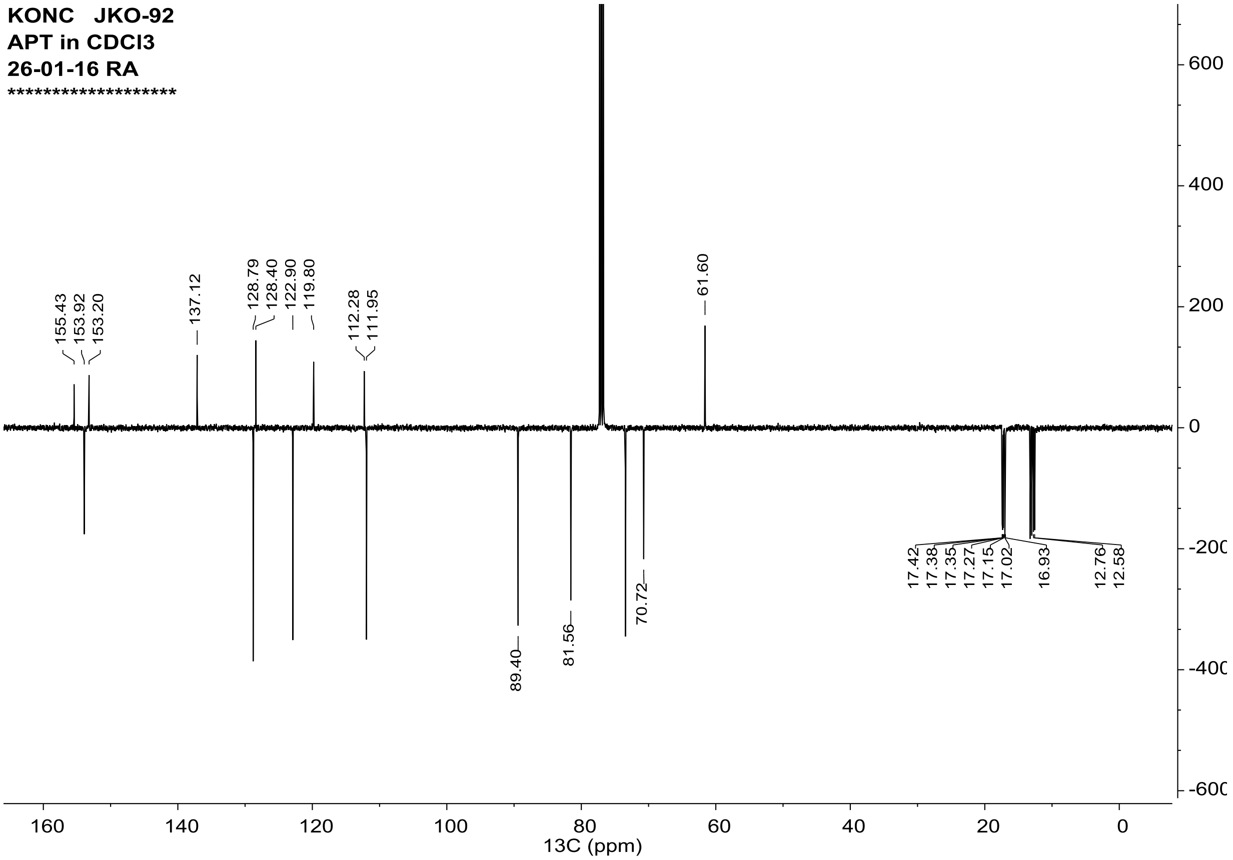
KONC JKO-92
APT in CDCl3
26-01-16 RA
155.43
153.92
153.20
137.12
128.79
128.40
122.90
119.80
112.28
111.95
61.60
89.40
81.56
70.72
17.42
17.38
17.35
17.27
17.15
17.02
16.93
12.76
12.58
13C (ppm)

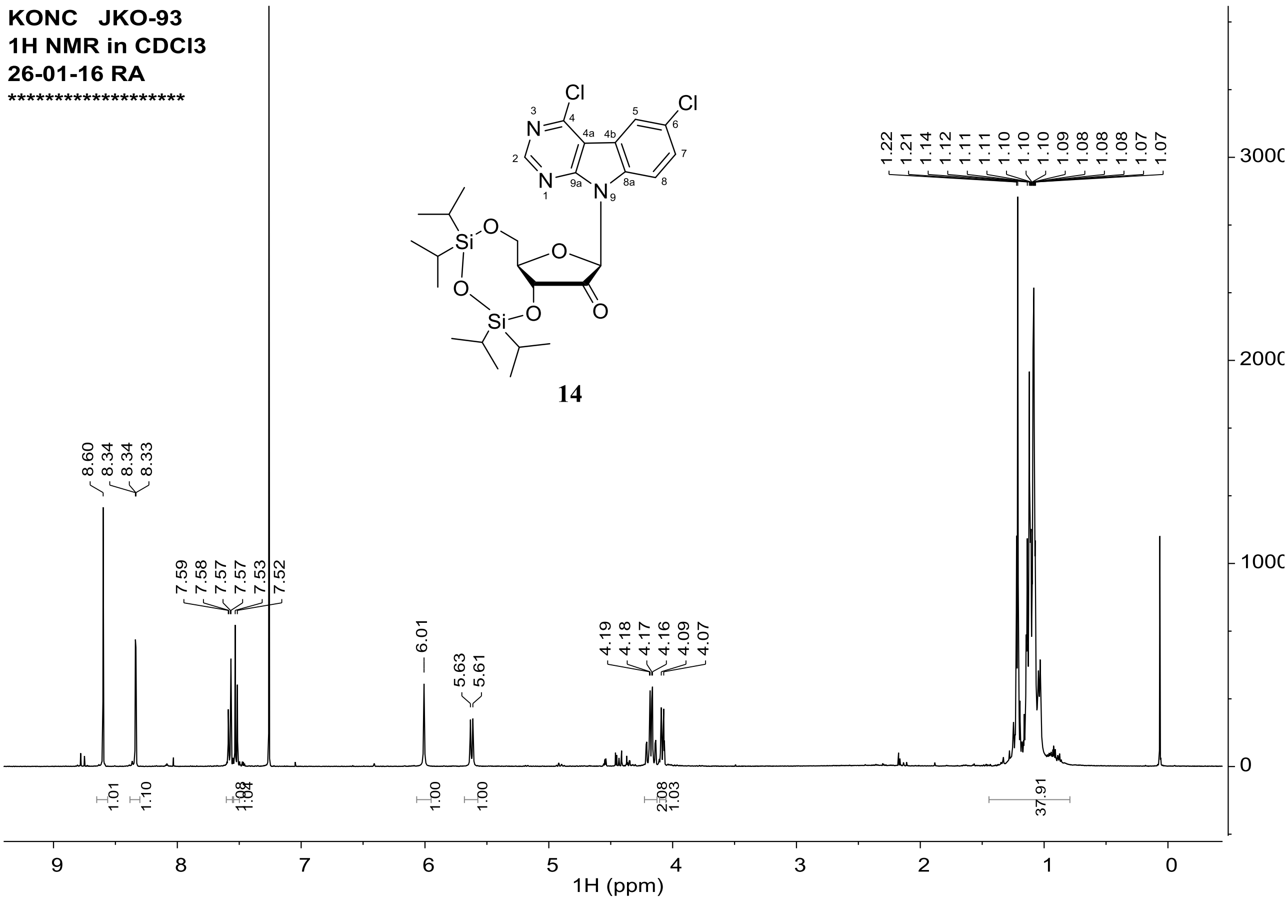
KONC JKO-93
1H NMR in CDCl3
26-01-16 RA
14
8.60
8.34
8.34
8.33
7.59
7.58
7.57
7.57
7.53
7.52
6.01
5.63
5.61
4.19
4.18
4.17
4.16
4.09
4.07
1.22
1.21
1.14
1.12
1.11
1.11
1.10
1.10
1.09
1.08
1.08
1.07
1.07
1.01
1.10
1.00
1.00
2.08
1.03
37.91
1H (ppm)

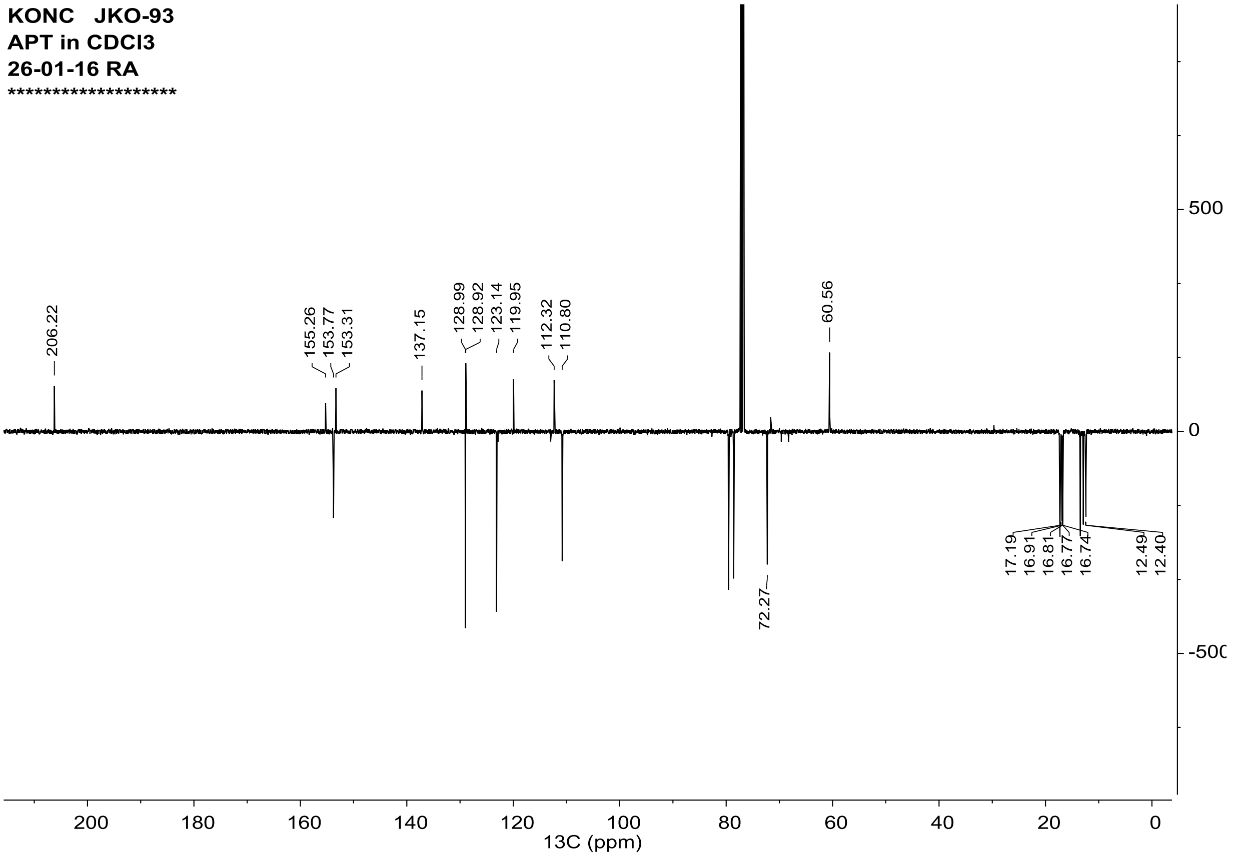
KONC JKO-93
APT in CDCl3
26-01-16 RA
****
206.22
155.26
153.77
153.31
137.15
128.99
128.92
123.14
119.95
112.32
110.80
60.56
72.27
17.19
16.91
16.81
16.77
16.74
12.49
12.40
500
0
-500
200
180
160
140
120
100
80
60
40
20
0
13C (ppm)

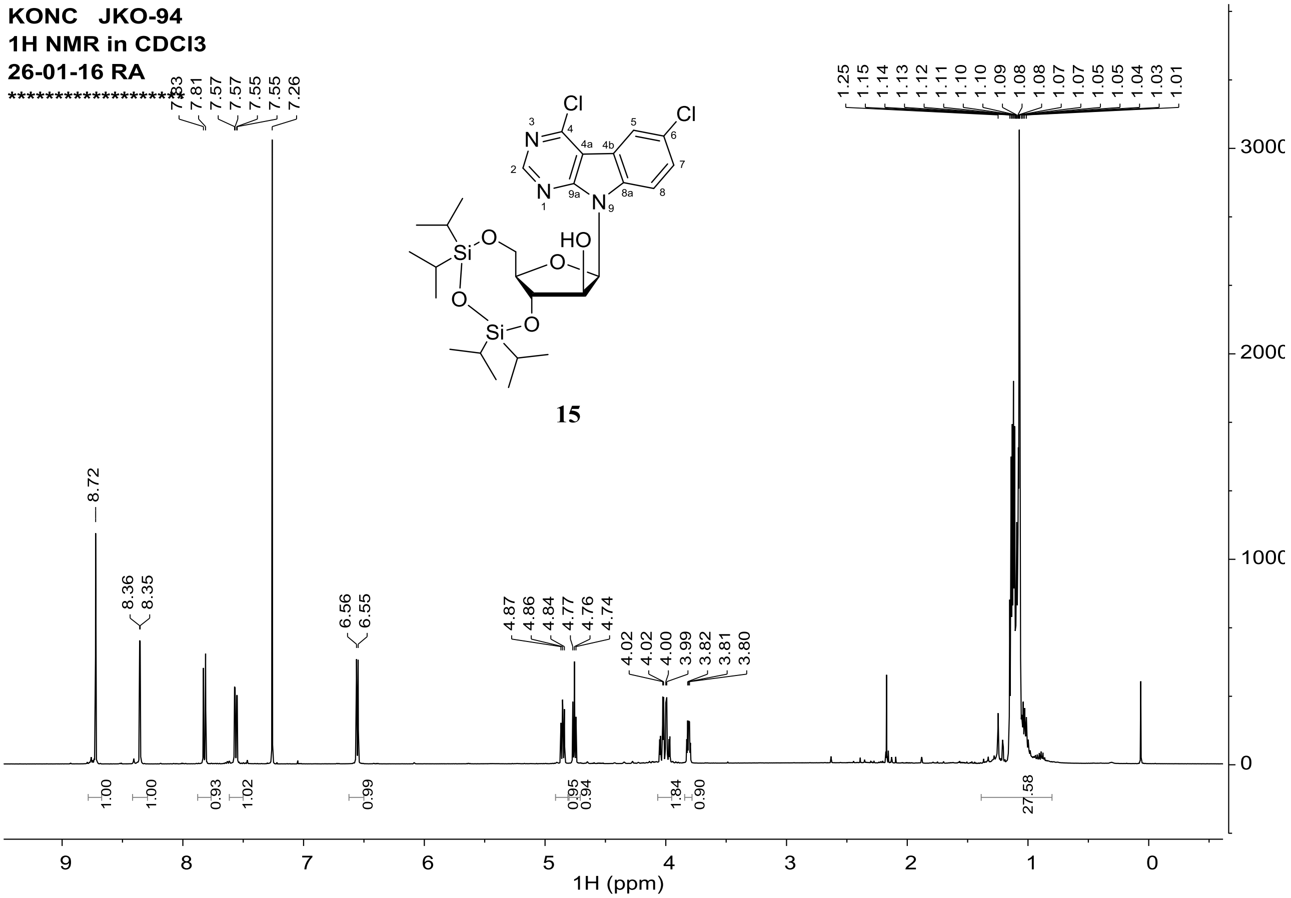
KONC JKO-94
1H NMR in CDCl3
26-01-16 RA
****
7.83
7.81
7.57
7.57
7.55
7.55
7.26
1.25
1.15
1.14
1.13
1.12
1.11
1.10
1.10
1.09
1.08
1.08
1.07
1.07
1.05
1.05
1.04
1.03
1.01
8.72
8.36
8.35
6.56
6.55
4.87
4.86
4.84
4.77
4.76
4.74
4.02
4.02
4.00
3.99
3.82
3.81
3.80
1.00
1.00
0.93
1.02
0.99
0.95
0.94
1.84
0.90
27.58
3000
2000
1000
0
9
8
7
6
5
4
3
2
1
0
1H (ppm)
Cl
Cl
N
N
N
HO
O
O
O
O
Si
Si
15

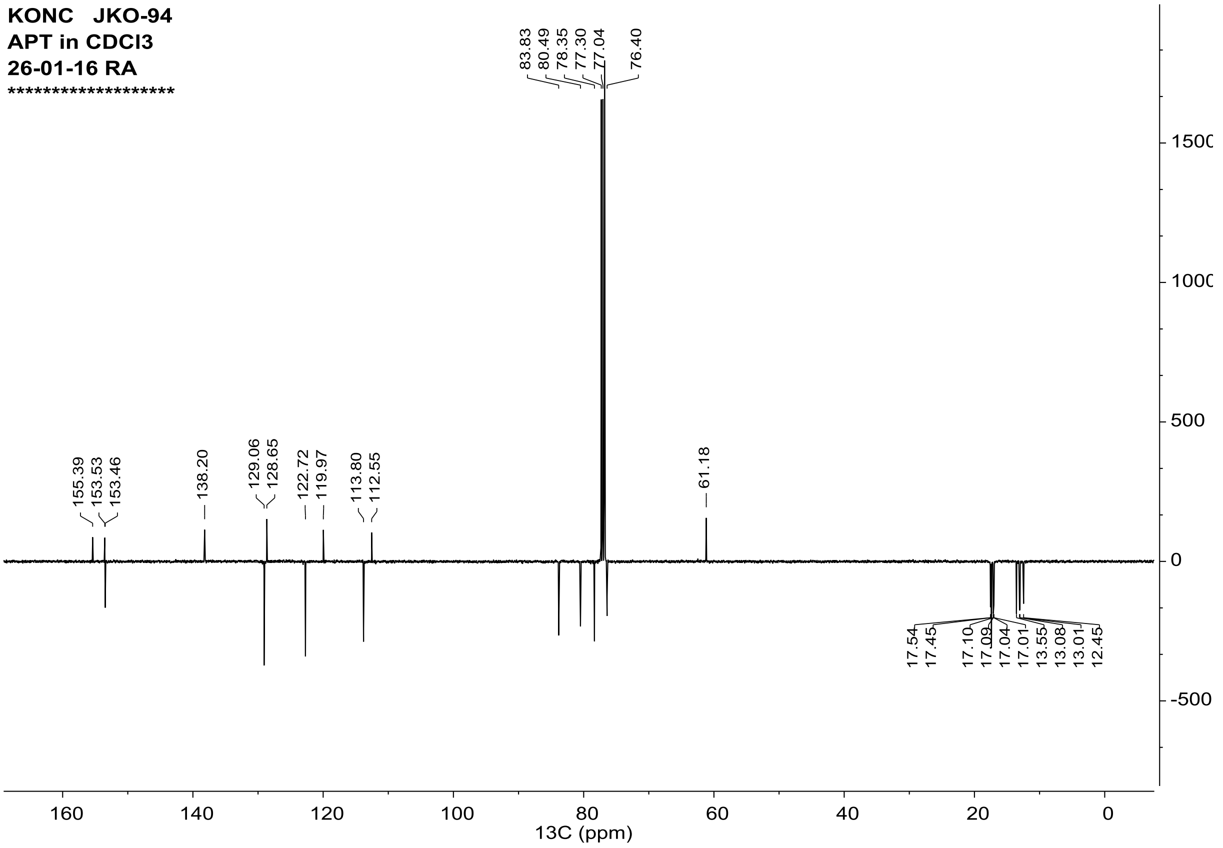
KONC JKO-94
APT in CDCl3
26-01-16 RA
****
83.83
80.49
78.35
77.30
77.04
76.40
155.39
153.53
153.46
138.20
129.06
128.65
122.72
119.97
113.80
112.55
61.18
17.54
17.45
17.10
17.09
17.04
17.01
13.55
13.08
13.01
12.45
1500
1000
500
0
-500
160
140
120
100
80
60
40
20
0
13C (ppm)

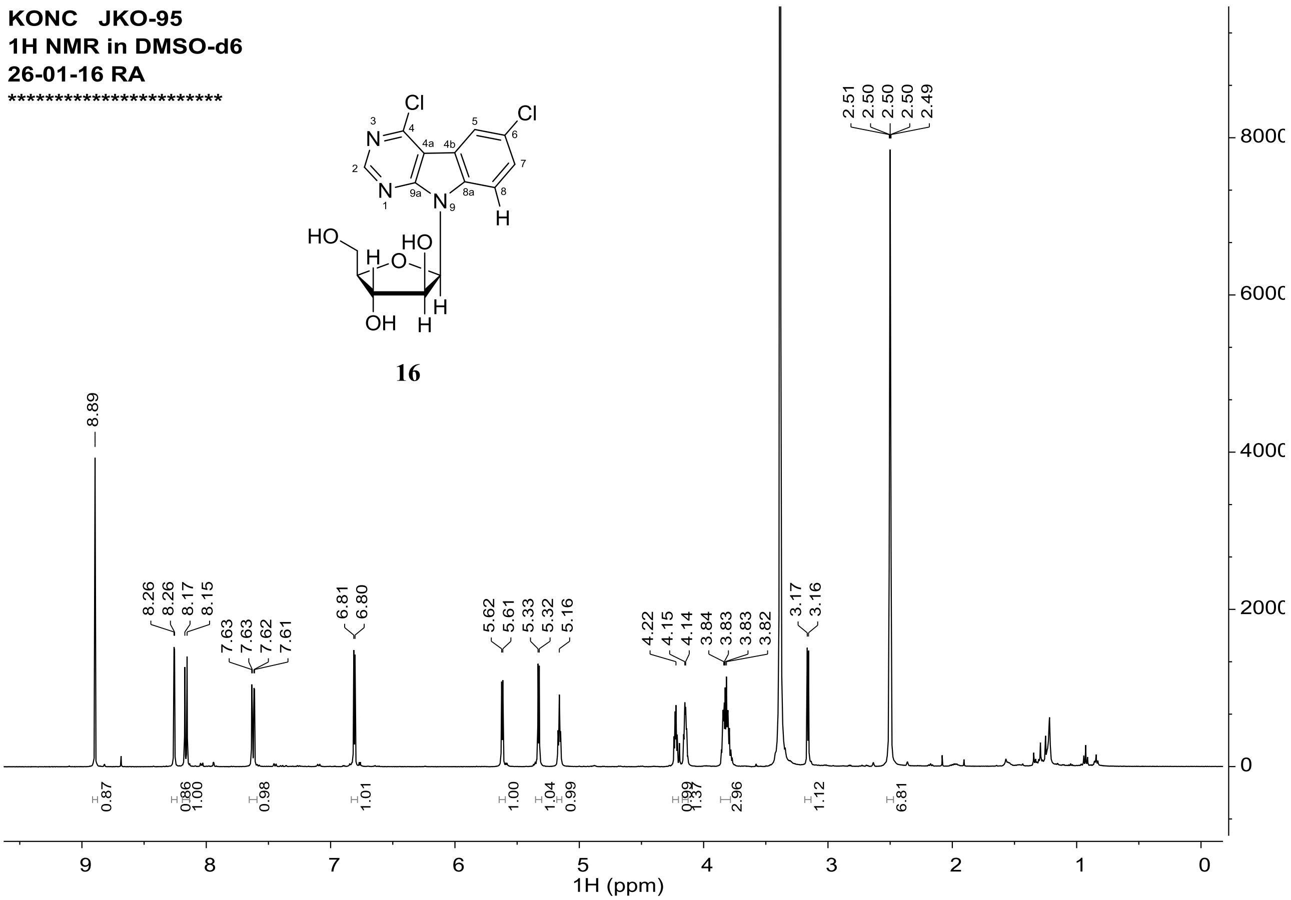
KONC JKO-95
1H NMR in DMSO-d6
26-01-16 RA
****
16
8.89
8.26
8.26
8.17
8.15
7.63
7.63
7.62
7.61
6.81
6.80
5.62
5.61
5.33
5.32
5.16
4.22
4.15
4.14
3.84
3.83
3.83
3.82
3.17
3.16
2.51
2.50
2.50
2.50
2.49
0.87
0.86
1.00
0.98
1.01
1.00
1.04
0.99
0.99
1.37
2.96
1.12
6.81
8000
6000
4000
2000
0
9
8
7
6
5
4
3
2
1
0
1H (ppm)

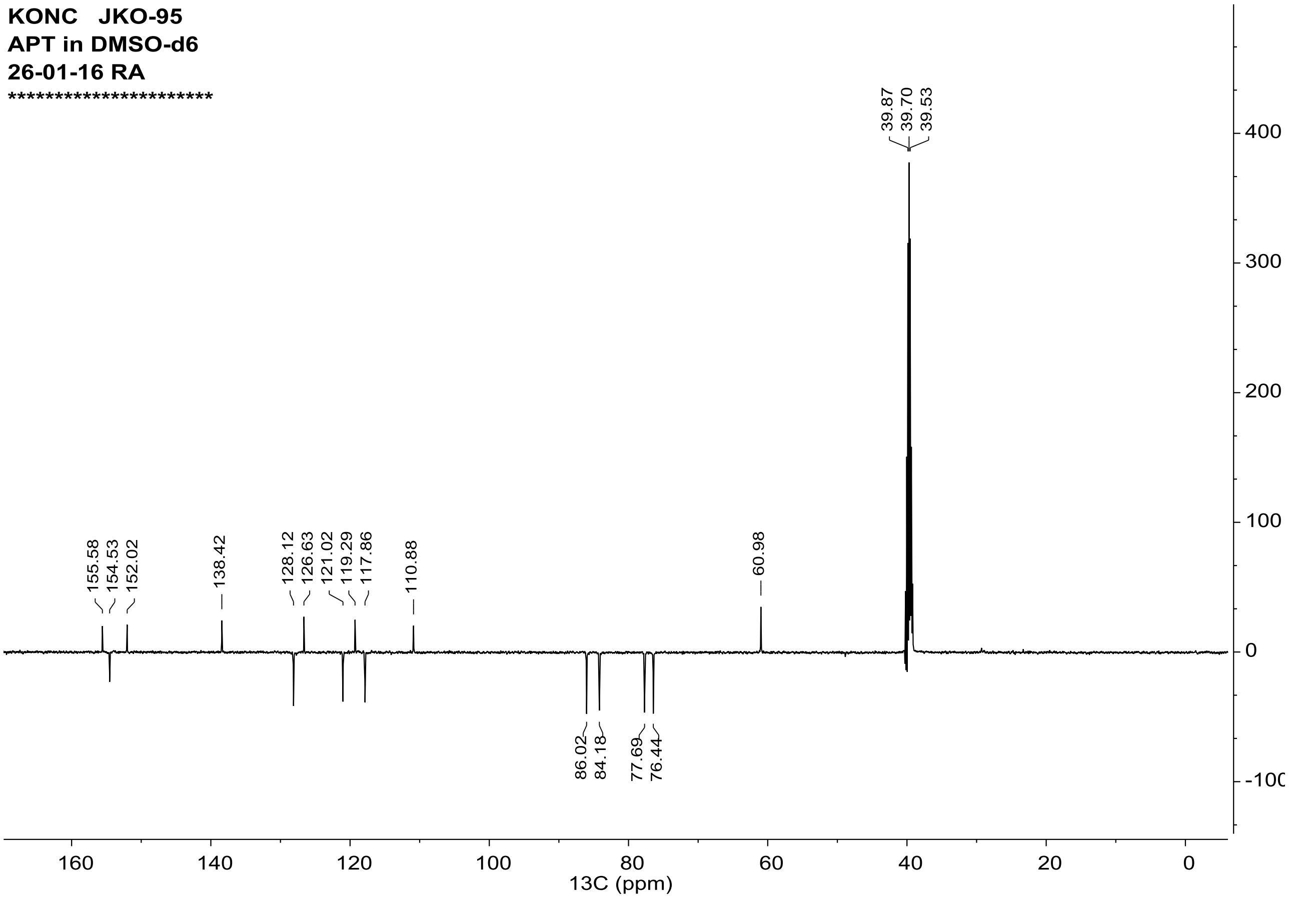
KONC JKO-95
APT in DMSO-d6
26-01-16 RA
****
39.87
39.70
39.53
155.58
154.53
152.02
138.42
128.12
126.63
121.02
119.29
117.86
110.88
60.98
86.02
84.18
77.69
76.44
13C (ppm)

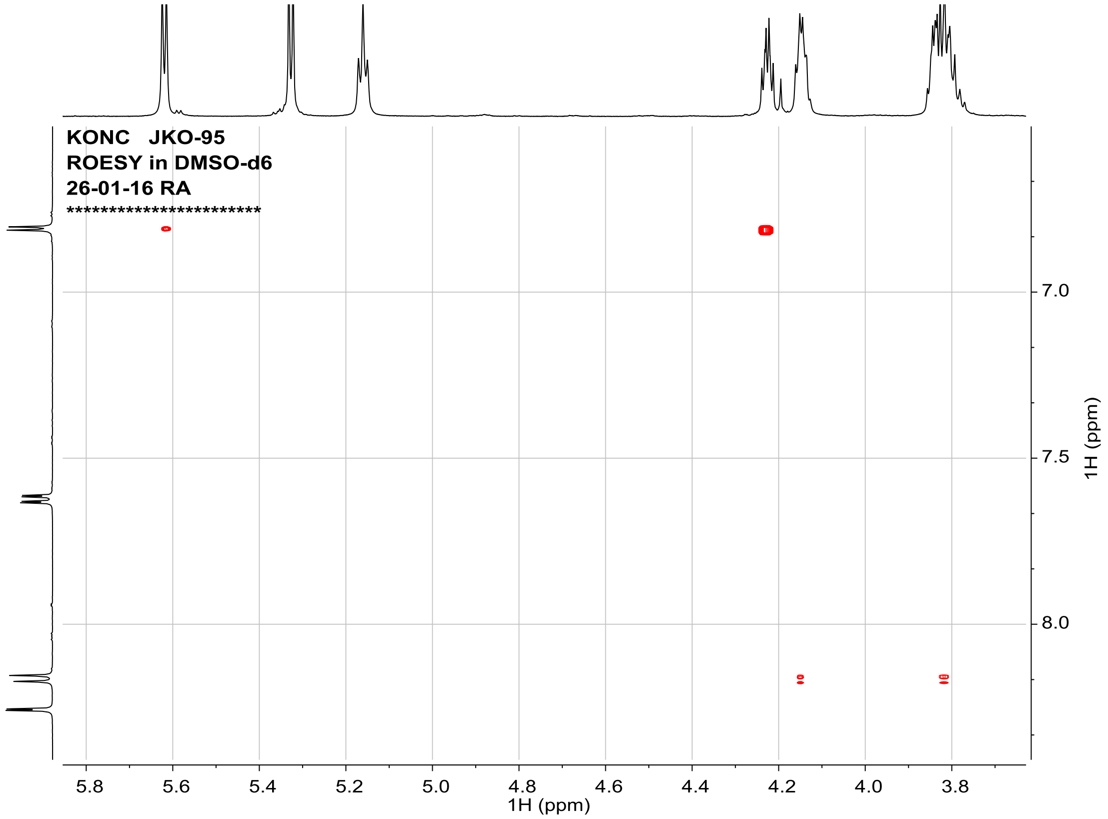
KONC JKO-95
ROESY in DMSO-d6
26-01-16 RA
****
1H (ppm)
1H (ppm)

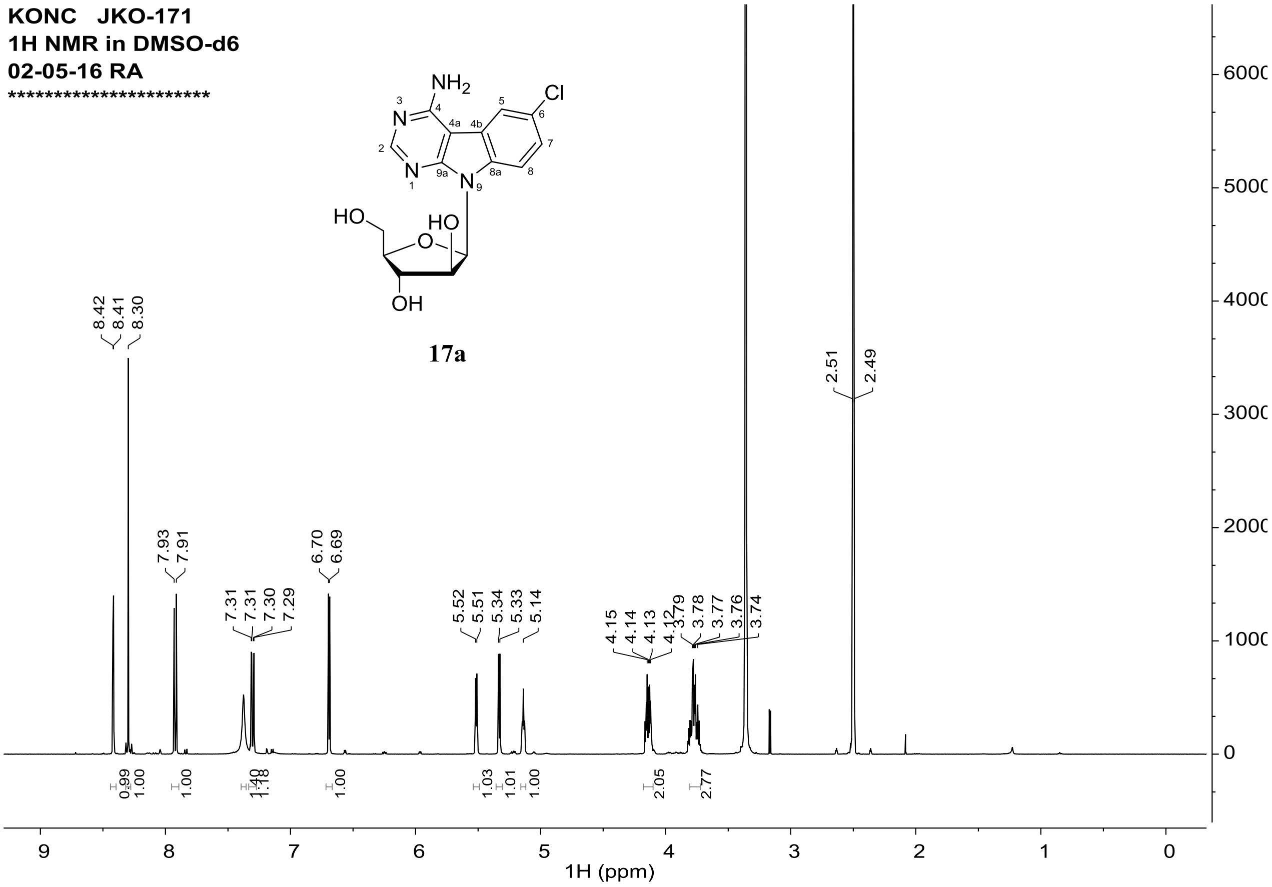
KONC JKO-171
1H NMR in DMSO-d6
02-05-16 RA
****
NH2
Cl
HO
HO
O
OH
N
17a
8.42
8.41
8.30
7.93
7.91
7.31
7.31
7.30
7.29
6.70
6.69
5.52
5.51
5.34
5.33
5.14
4.15
4.14
4.13
4.12
3.79
3.78
3.77
3.76
3.74
2.51
2.49
0.99
1.00
1.00
1.40
1.18
1.00
1.03
1.01
1.00
2.05
2.77
1H (ppm)

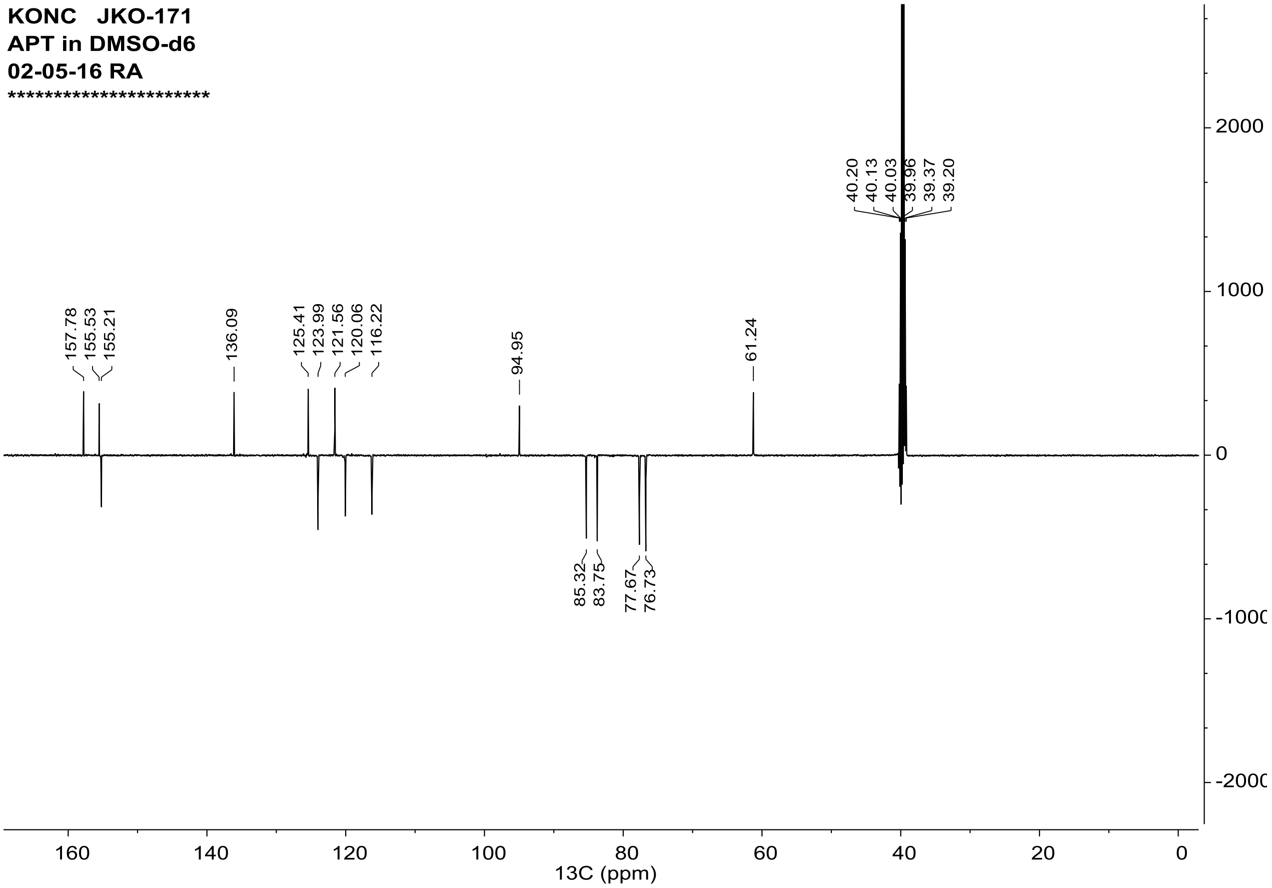
KONC JKO-171
APT in DMSO-d6
02-05-16 RA
****
157.78
155.53
155.21
136.09
125.41
123.99
121.56
120.06
116.22
94.95
85.32
83.75
77.67
76.73
61.24
40.20
40.13
40.03
39.96
39.37
39.20
13C (ppm)

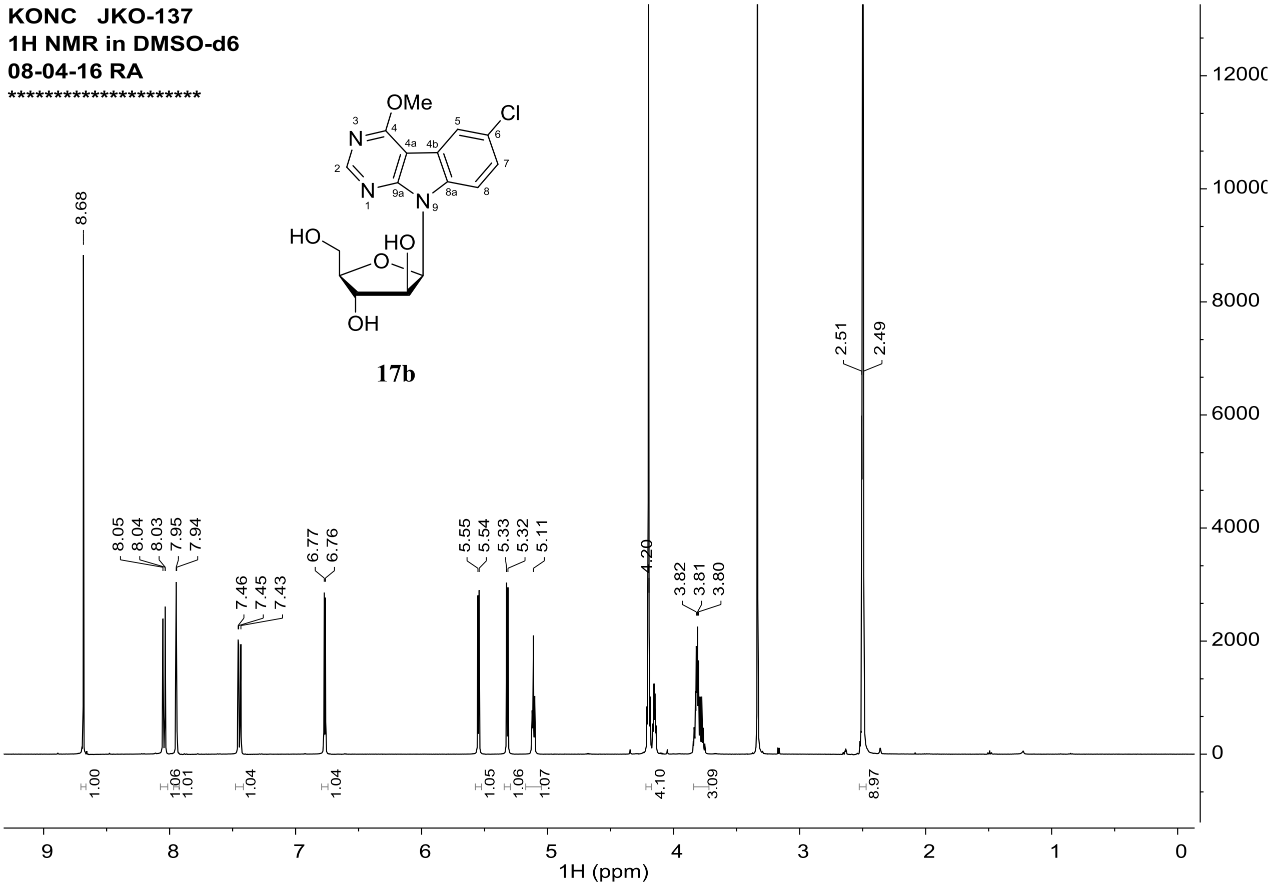
KONC JKO-137
1H NMR in DMSO-d6
08-04-16 RA
****
OMe
Cl
HO
HO
OH
17b
8.68
8.05
8.04
8.03
7.95
7.94
7.46
7.45
7.43
6.77
6.76
5.55
5.54
5.33
5.32
5.11
4.20
3.82
3.81
3.80
2.51
2.49
1.00
1.06
1.01
1.04
1.04
1.05
1.06
1.07
4.10
3.09
8.97
1H (ppm)

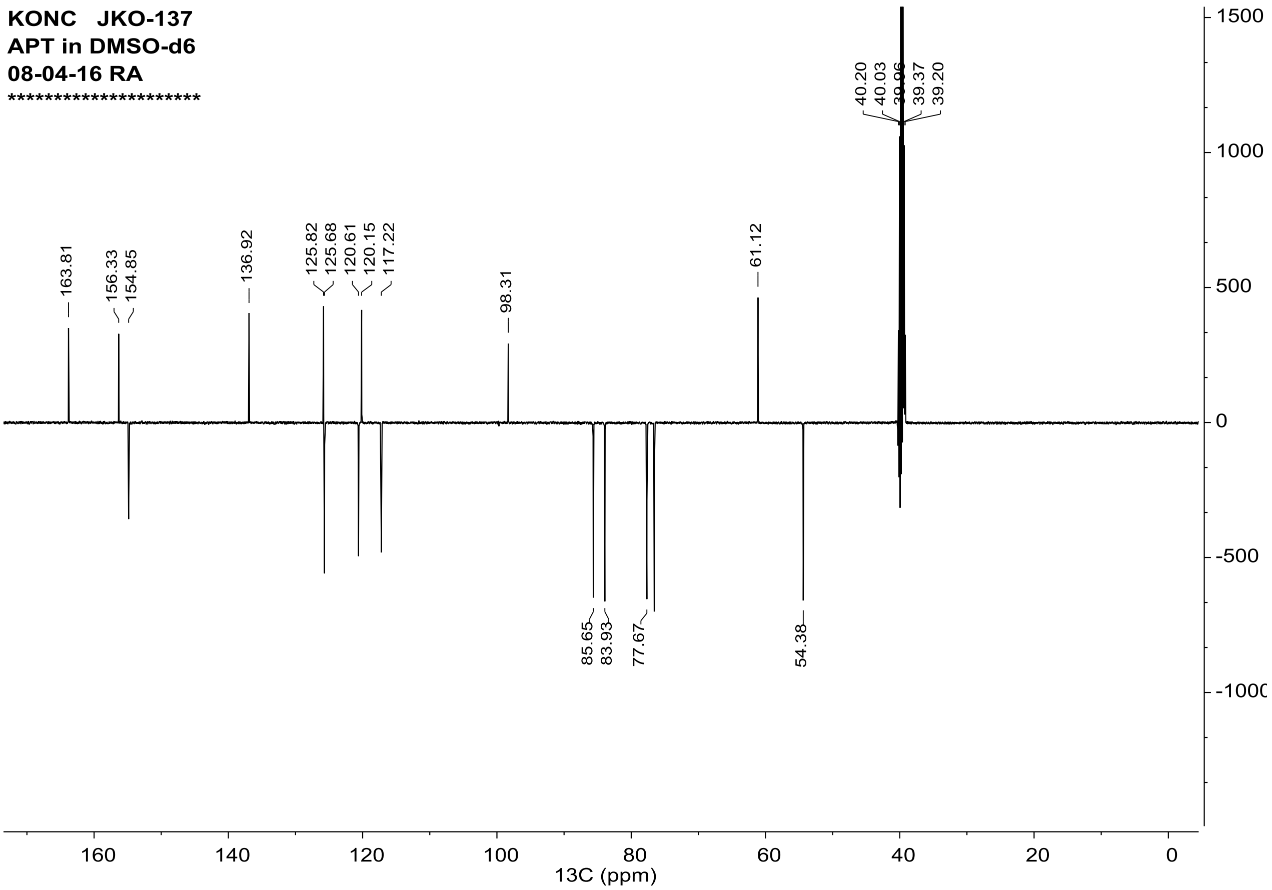
KONC JKO-137
APT in DMSO-d6
08-04-16 RA
****
163.81
156.33
154.85
136.92
125.82
125.68
120.61
120.15
117.22
98.31
85.65
83.93
77.67
61.12
54.38
40.20
40.03
39.86
39.37
39.20
13C (ppm)

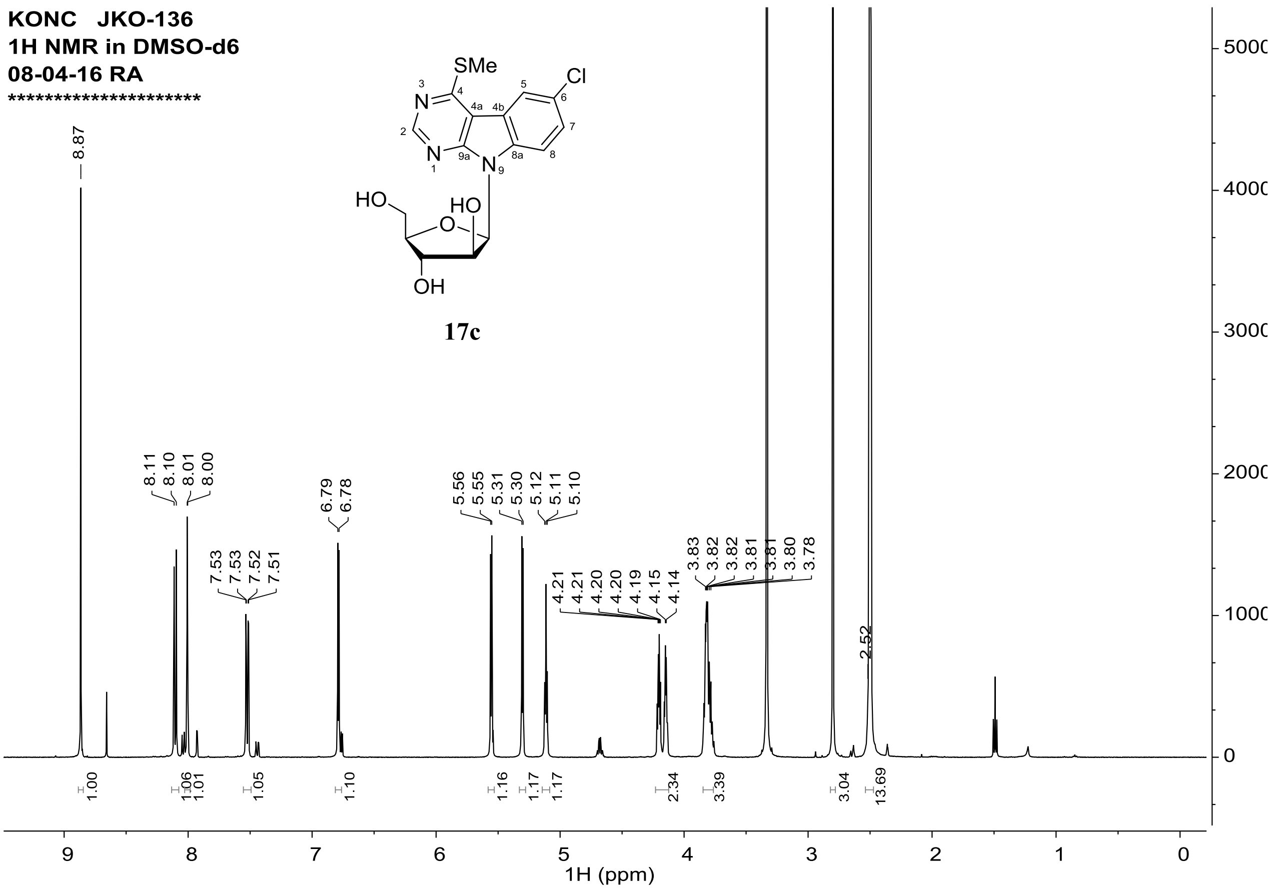
KONC JKO-136
1H NMR in DMSO-d6
08-04-16 RA
****
SMe
Cl
HO
HO
OH
17c
8.87
8.11
8.10
8.01
8.00
7.53
7.53
7.52
7.51
6.79
6.78
5.56
5.55
5.31
5.30
5.12
5.11
5.10
4.21
4.21
4.20
4.20
4.19
4.15
4.14
3.83
3.82
3.82
3.81
3.81
3.80
3.78
2.52
1.00
1.06
1.01
1.05
1.10
1.16
1.17
1.17
2.34
3.39
3.04
13.69
1H (ppm)

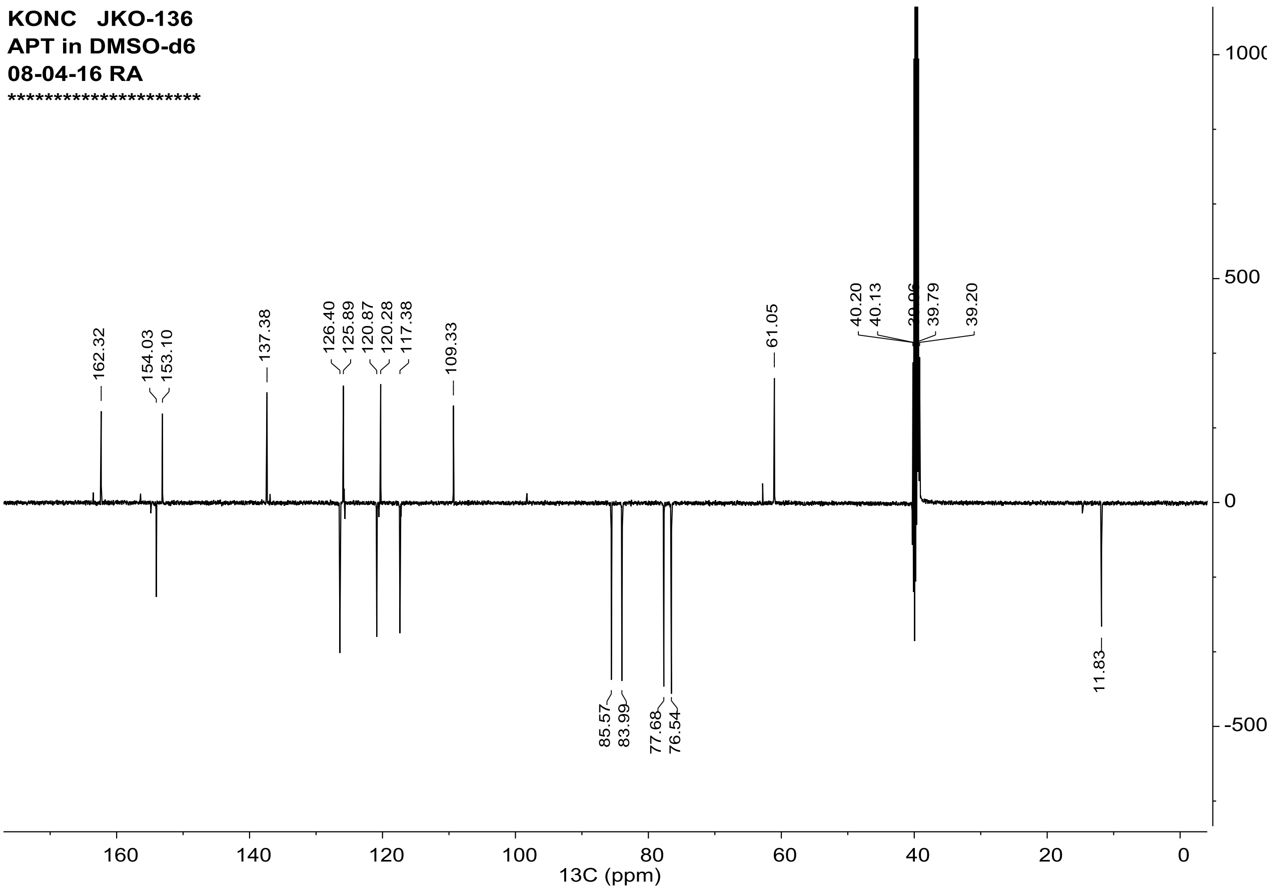
KONC JKO-136
APT in DMSO-d6
08-04-16 RA
****
162.32
154.03
153.10
137.38
126.40
125.89
120.87
120.28
117.38
109.33
61.05
40.20
40.13
39.79
39.20
85.57
83.99
77.68
76.54
11.83
13C (ppm)

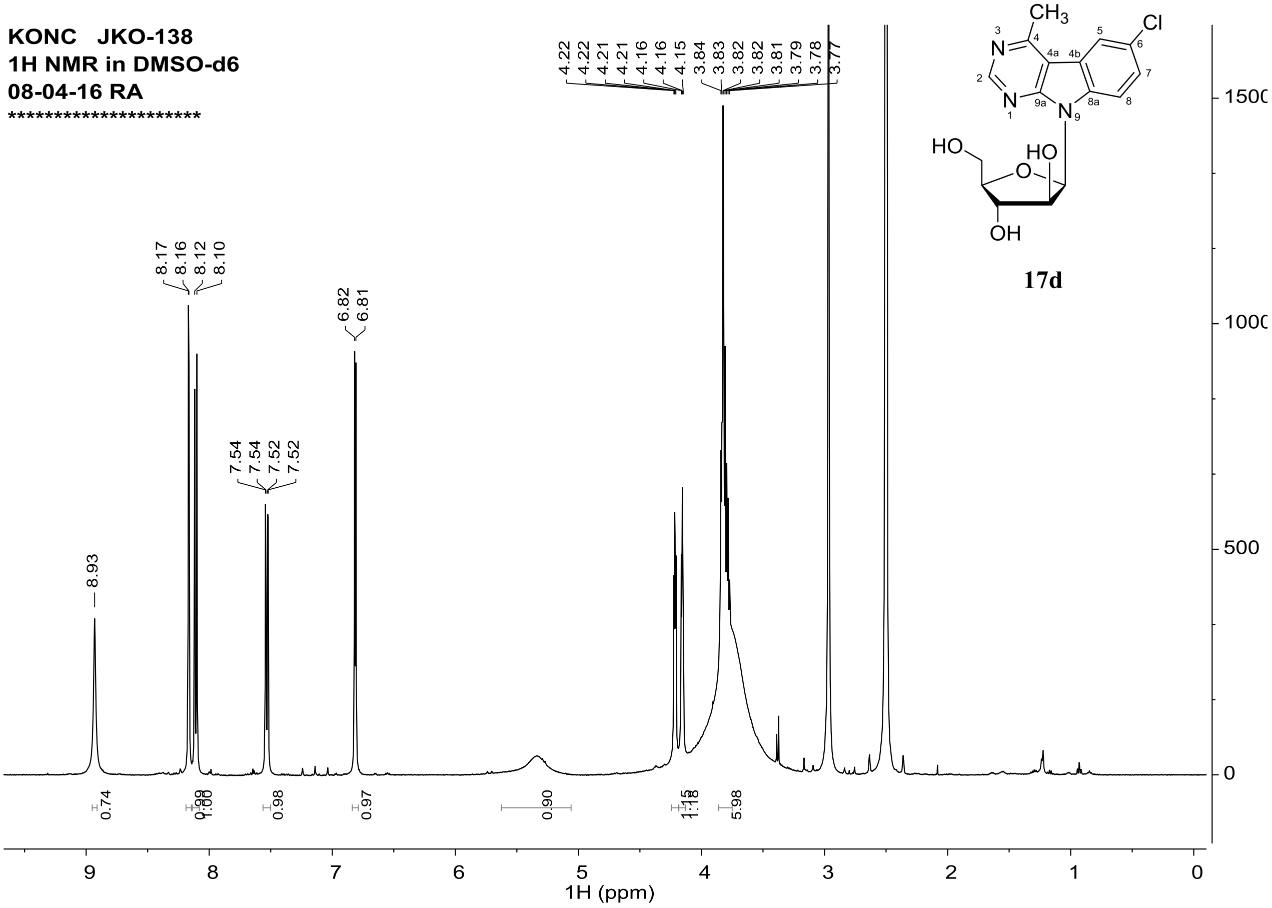
KONC JKO-138
1H NMR in DMSO-d6
08-04-16 RA
****
4.22
4.22
4.21
4.21
4.16
4.16
4.15
3.84
3.83
3.82
3.82
3.81
3.79
3.78
3.77
8.17
8.16
8.12
8.10
6.82
6.81
7.54
7.54
7.52
7.52
8.93
0.74
0.99
1.00
0.98
0.97
0.90
1.15
1.18
5.98
CH3
Cl
HO
HO
O
OH
N
N
N
17d
1H (ppm)

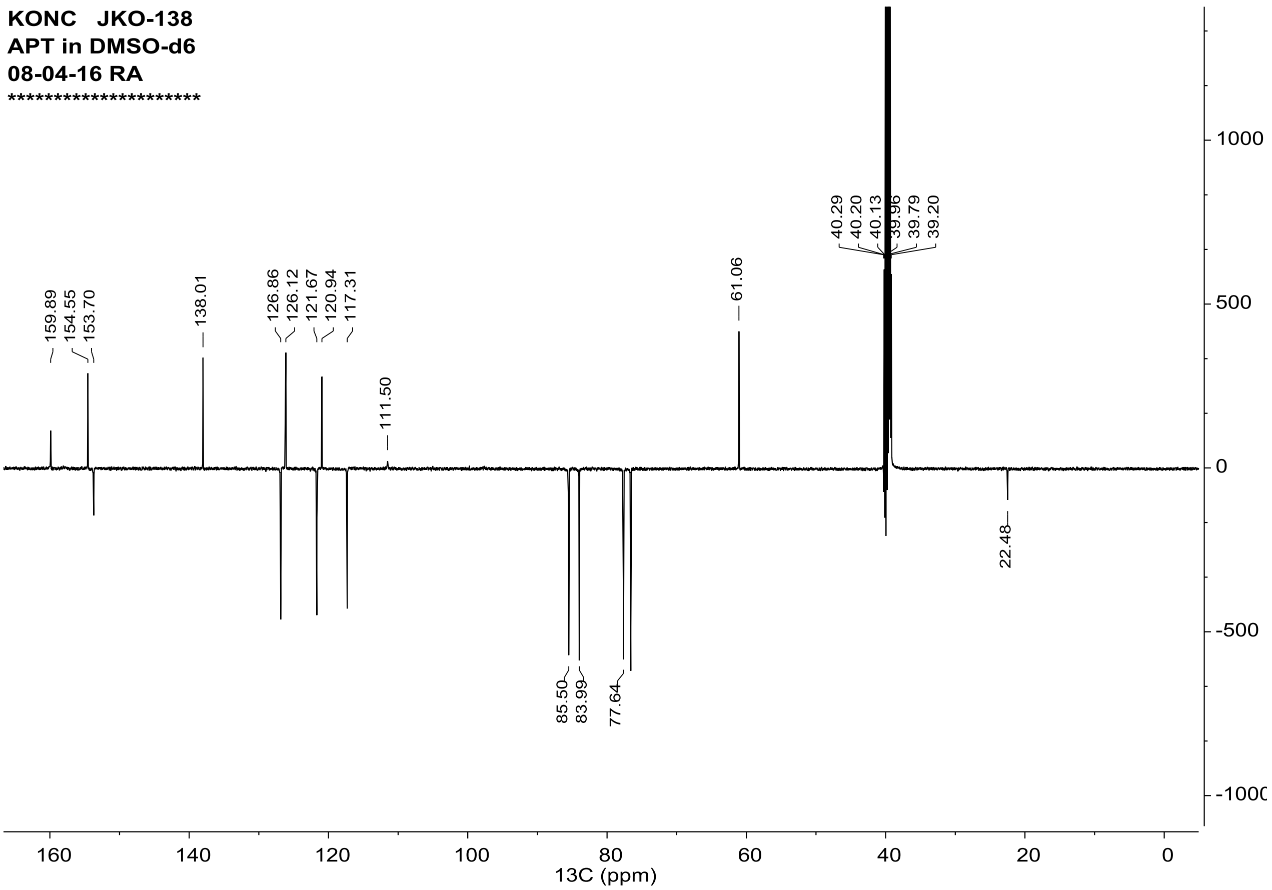
KONC JKO-138
APT in DMSO-d6
08-04-16 RA
****
159.89
154.55
153.70
138.01
126.86
126.12
121.67
120.94
117.31
111.50
61.06
40.29
40.20
40.13
39.96
39.79
39.20
85.50
83.99
77.64
22.48
13C (ppm)

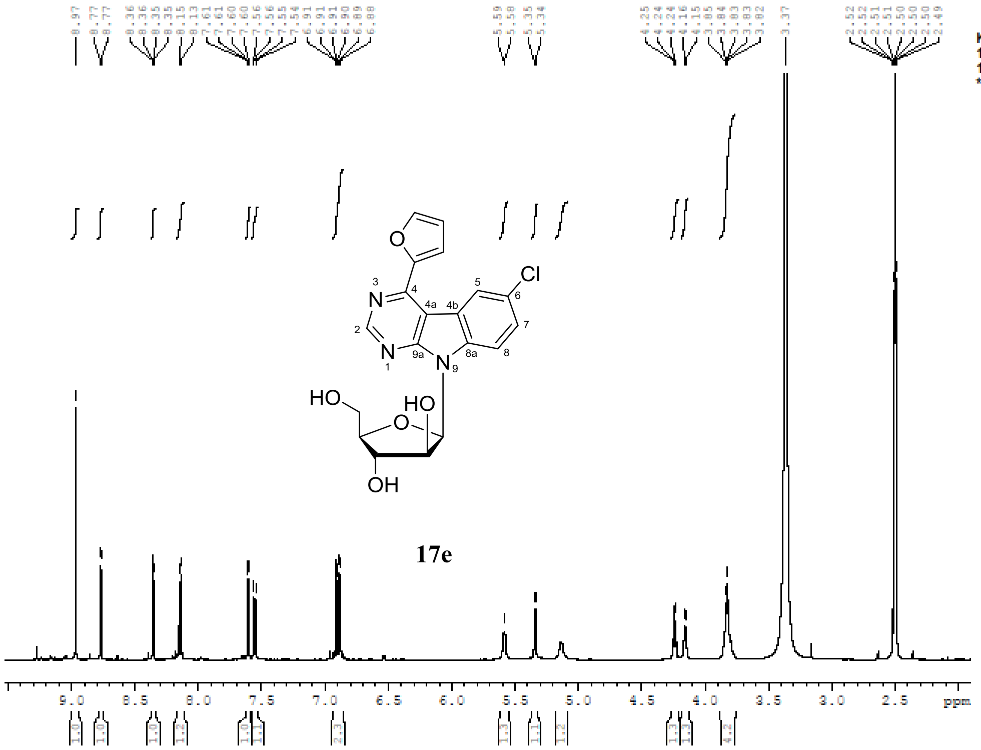

KONC JKO-206
1H NMR in DMSO-d6
12-12-16 RA
****

Current Data Parameters

NAME	KONC_JKO-206
EXPNO	1
PROCNO	1

F2 - Acquisition Parameters

Date_	20161212
Time	22.13
INSTRUM	spect
PROBHD	5 mm CPBBO BB-
PULPROG	zg30
TD	60094
SOLVENT	DMSO
NS	32
DS	2
SWH	6009.615 Hz
FIDRES	0.100004 Hz
AQ	4.9998207 sec
RG	18.17
DW	83.200 usec
DE	10.00 usec
TE	298.0 K
D1	1.00000000 sec
TD0	1

======== CHANNEL f1 ========

SFO1	499.9827499 MHz
NUC1	1H
P1	10.00 usec
PLW1	7.40000010 W

F2 - Processing parameters

SI	65536
SF	499.9800035 MHz
WDW	EM
SSB	0
LB	0.30 Hz
GB	0
PC	1.00

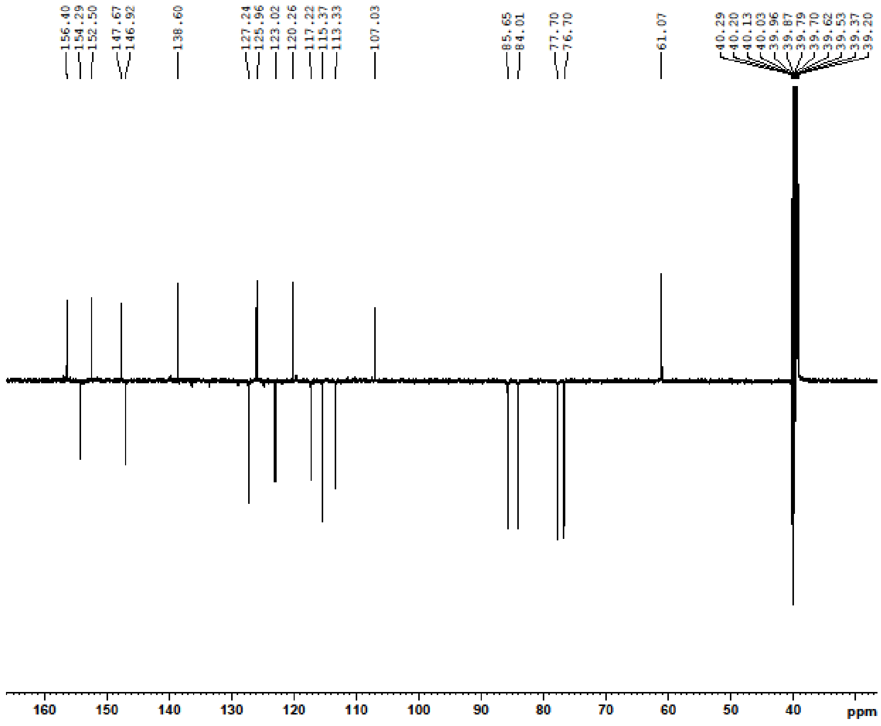

KONC JKO-206
APT in DMSO-d6
12-12-16 RA
****

Current Data Parameters

NAME	KONC_JKO-206
EXPNO	2
PROCNO	1

F2 - Acquisition Parameters

Date_	20161213
Time	0.01
INSTRUM	spect
PROBHD	5 mm CPBBO BB-
PULPROG	jmod
TD	65536
SOLVENT	DMSO
NS	2048
DS	4
SWH	29761.904 Hz
FIDRES	0.454131 Hz
AQ	1.1010048 sec
RG	199.71
DW	16.800 usec
DE	18.00 usec
TE	298.0 K
CNST2	160.0000000
CNST11	1.0000000
D1	2.00000000 sec
D20	0.00625000 sec
TD0	1

======== CHANNEL f1 ========

SFO1	125.7326430 MHz
NUC1	13C
P1	12.00 usec
P2	24.00 usec
PLW1	35.00000000 W

======== CHANNEL f2 ========

SFO2	499.9819999 MHz
NUC2	1H
CPDPRG[2	waltz16
PCPD2	80.00 usec
PLW2	8.60000038 W
PLW12	0.19350000 W

F2 - Processing parameters

SI	65536
SF	125.7201016 MHz
WDW	EM
SSB	0
LB	1.00 Hz
GB	0
PC	1.40

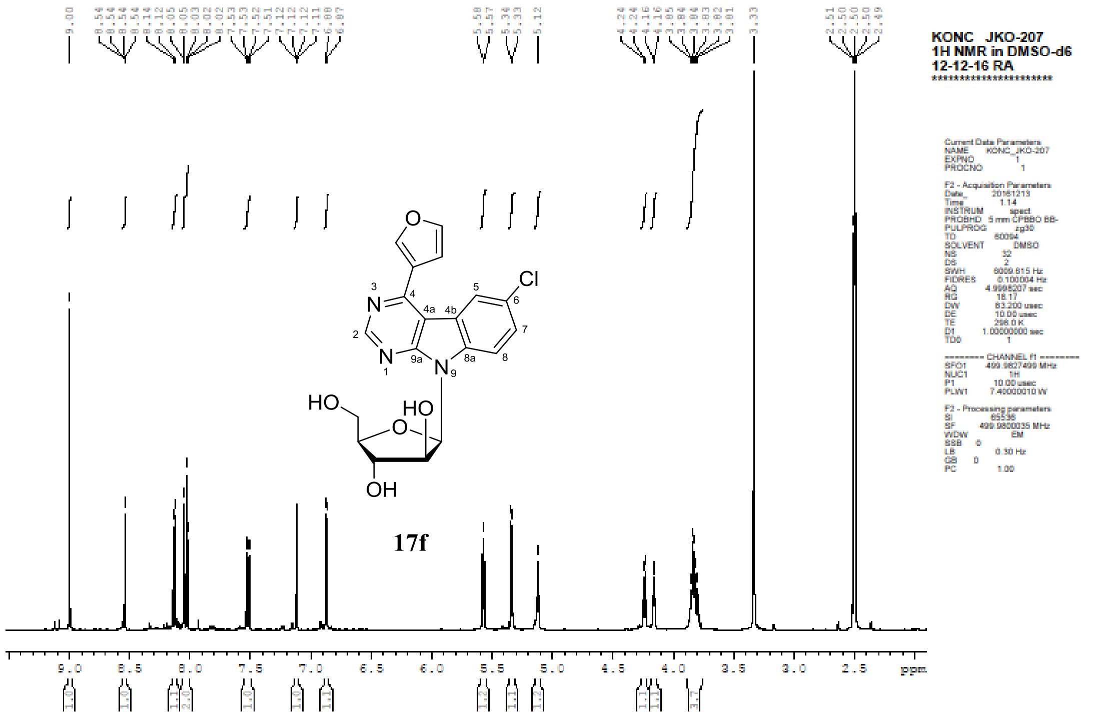
KONC JKO-207
1H NMR in DMSO-d6
12-12-16 RA
17f

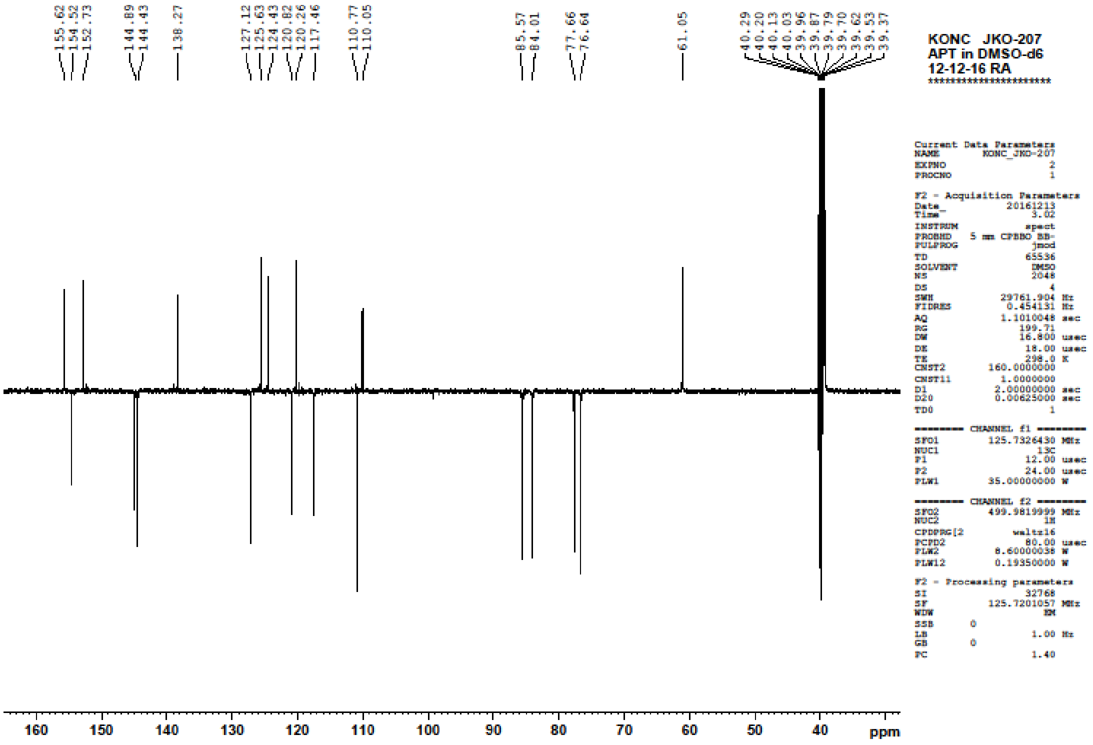
KONC JKO-207
APT in DMSO-d6
12-12-16 RA
155.62
154.52
152.73
144.89
144.43
138.27
127.12
125.63
124.43
120.82
120.26
117.46
110.77
110.05
85.57
84.01
77.66
76.64
61.05
40.29
40.20
40.13
40.03
39.96
39.87
39.79
39.70
39.62
39.53
39.37
ppm

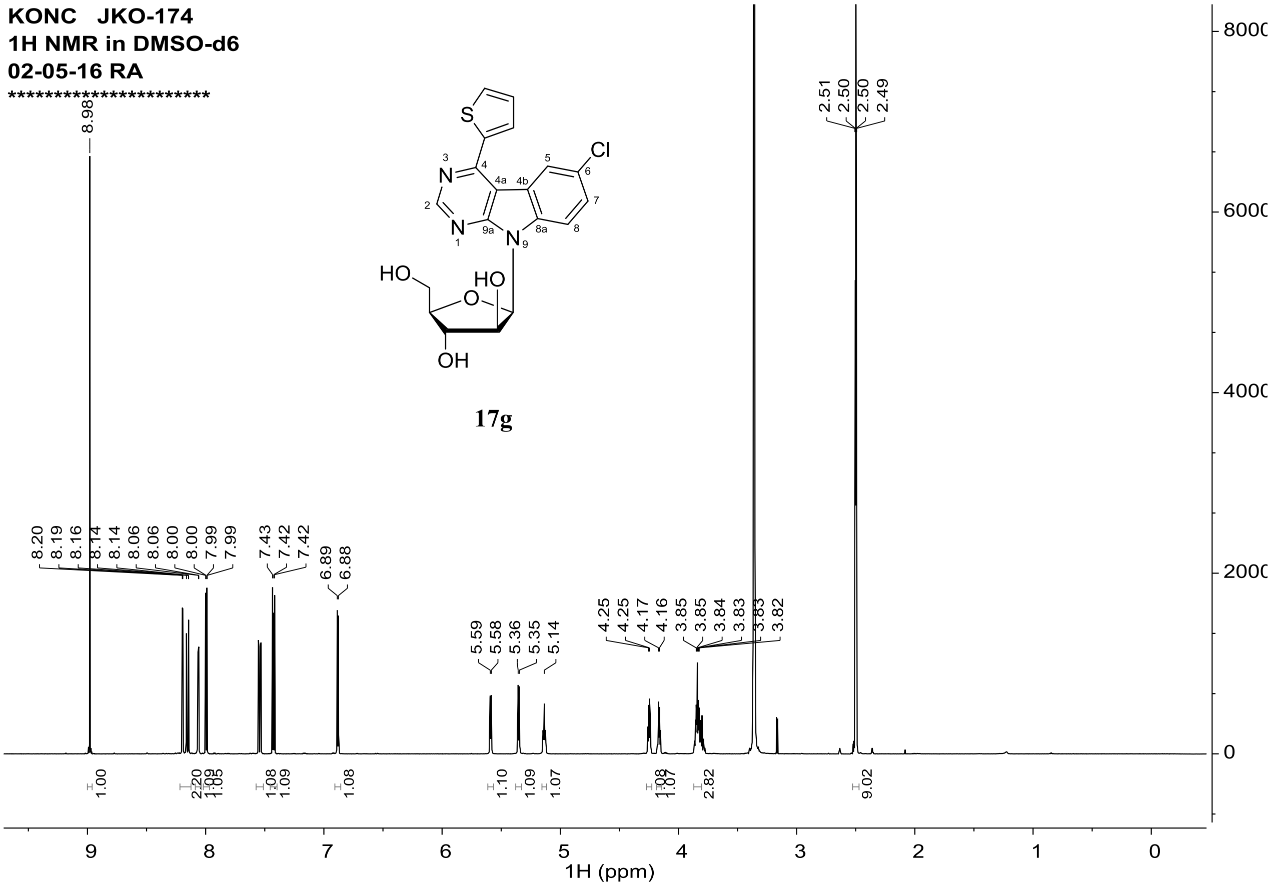
KONC JKO-174
1H NMR in DMSO-d6
02-05-16 RA
17g
S
Cl
HO
HO
OH
8.98
8.20 8.19 8.16 8.14 8.14 8.06 8.06 8.00 8.00 7.99 7.99
7.43 7.42 7.42
6.89 6.88
5.59 5.58 5.36 5.35 5.14
4.25 4.25 4.17 4.16 3.85 3.85 3.84 3.83 3.83 3.82
2.51 2.50 2.50 2.49
1.00
1.05
1.08
1.09
1.08
1.10
1.09
1.07
1.07
2.82
9.02
1H (ppm)

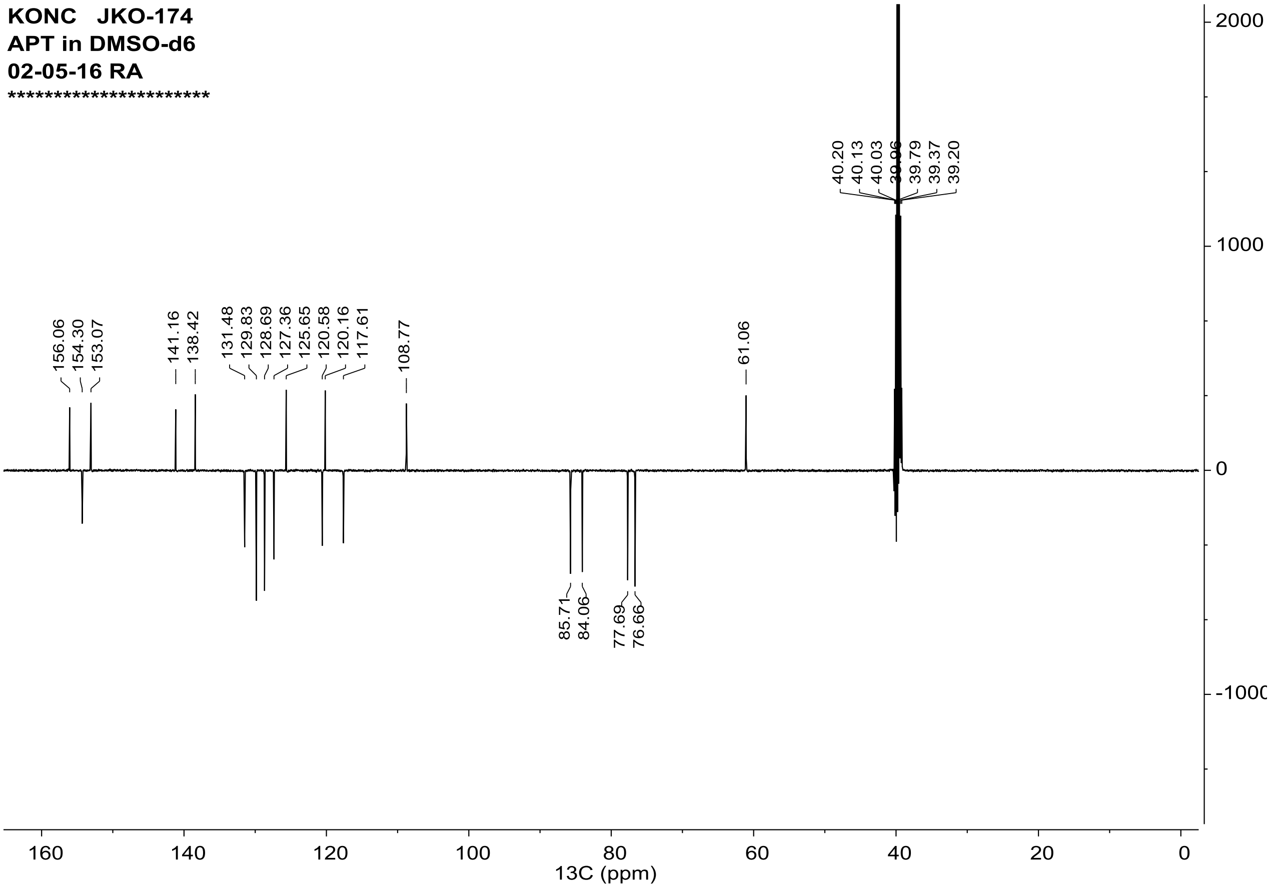
KONC JKO-174
APT in DMSO-d6
02-05-16 RA
156.06 154.30 153.07
141.16 138.42
131.48 129.83 128.69 127.36 125.65 120.58 120.16 117.61
108.77
61.06
40.20 40.13 40.03 39.96 39.79 39.37 39.20
85.71 84.06
77.69 76.66
13C (ppm)

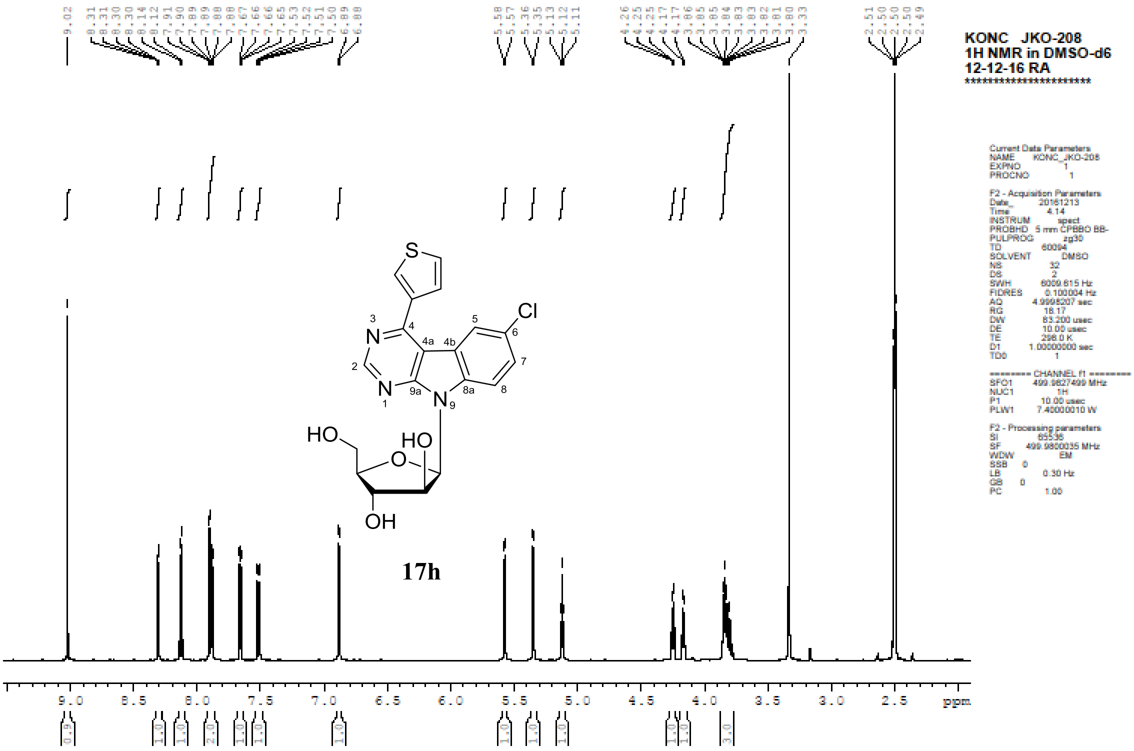

17h

KONC JKO-208
1H NMR in DMSO-d6
12-12-16 RA
****

Current Data Parameters
NAME KONC_JKO-208
EXPNO 1
PROCNO 1

F2 - Acquisition Parameters
Date_ 20161213
Time 4.14
INSTRUM spect
PROBHD 5 mm CPBBO BB-
PULPROG zg30
TD 60094
SOLVENT DMSO
NS 32
DS 2
SWH 6009.615 Hz
FIDRES 0.100004 Hz
AQ 4.9995207 sec
RG 18.17
DW 83.200 usec
DE 10.00 usec
TE 298.0 K
D1 1.00000000 sec
TD0 1

======== CHANNEL f1 ========
SFO1 499.9827499 MHz
NUC1 1H
P1 10.00 usec
PLW1 7.40000010 W

F2 - Processing parameters
SI 65536
SF 499.9800035 MHz
WDW EM
SSB 0
LB 0.30 Hz
GB 0
PC 1.00

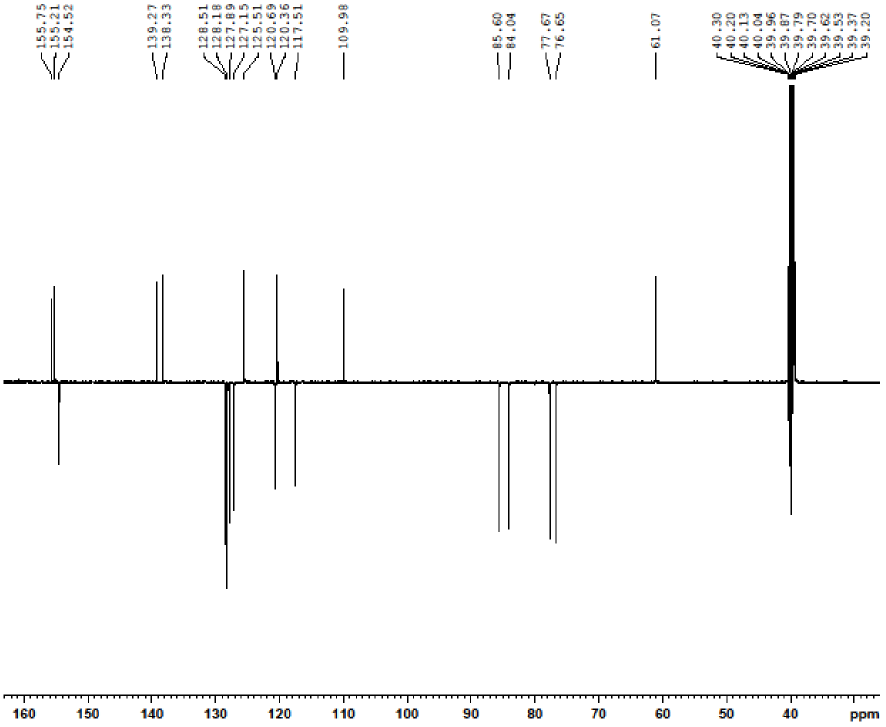

KONC JKO-208
APT in DMSO-d6
12-12-16 RA
****

Current Data Parameters
NAME KONC_JKO-208
EXPNO 2
PROCNO 1

F2 - Acquisition Parameters
Date_ 20161213
Time 6.02
INSTRUM spect
PROBHD 5 mm CPBBO BB-
PULPROG jmod
TD 65536
SOLVENT DMSO
NS 2048
DS 4
SWH 29761.904 Hz
FIDRES 0.454131 Hz
AQ 1.1010048 sec
RG 199.71
DW 16.800 usec
DE 18.00 usec
TE 298.0 K
CNST2 160.0000000
CNST11 1.0000000
D1 2.00000000 sec
D20 0.00625000 sec
TD0 1

======== CHANNEL f1 ========
SFO1 125.7326430 MHz
NUC1 13C
P1 12.00 usec
P2 24.00 usec
PLW1 35.00000000 W

======== CHANNEL f2 ========
SFO2 499.9819999 MHz
NUC2 1H
CPDPRG[2 waltz16
PCPD2 80.00 usec
PLW2 8.60000038 W
PLW12 0.19350000 W

F2 - Processing parameters
SI 65536
SF 125.7201059 MHz
WDW EM
SSB 0
LB 1.00 Hz
GB 0
PC 1.40

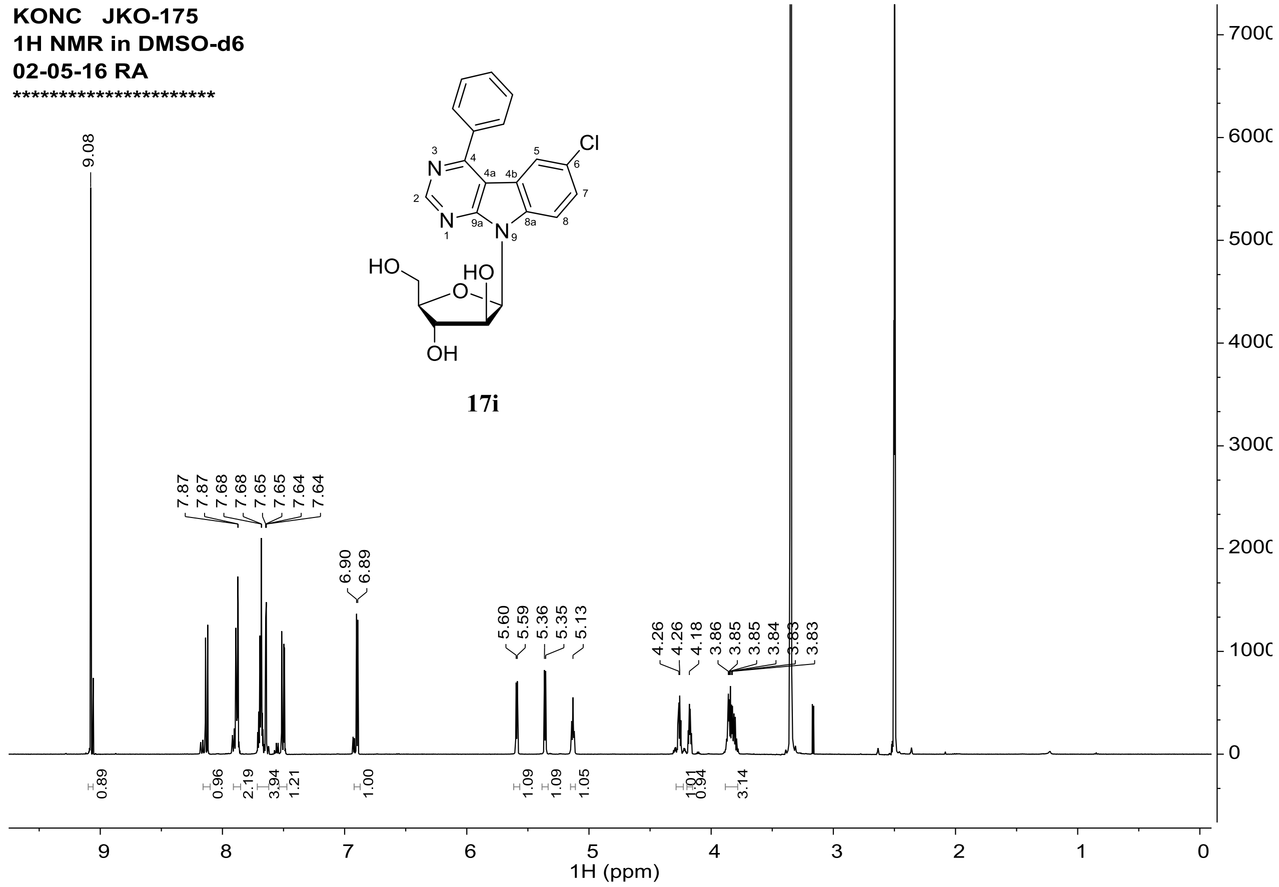
KONC JKO-175
1H NMR in DMSO-d6
02-05-16 RA
****
17i
9.08
7.87
7.87
7.68
7.68
7.65
7.65
7.64
7.64
6.90
6.89
5.60
5.59
5.36
5.35
5.13
4.26
4.26
4.18
3.86
3.85
3.85
3.84
3.83
3.83
0.89
0.96
2.19
3.94
1.21
1.00
1.09
1.09
1.05
1.01
0.94
3.14
1H (ppm)

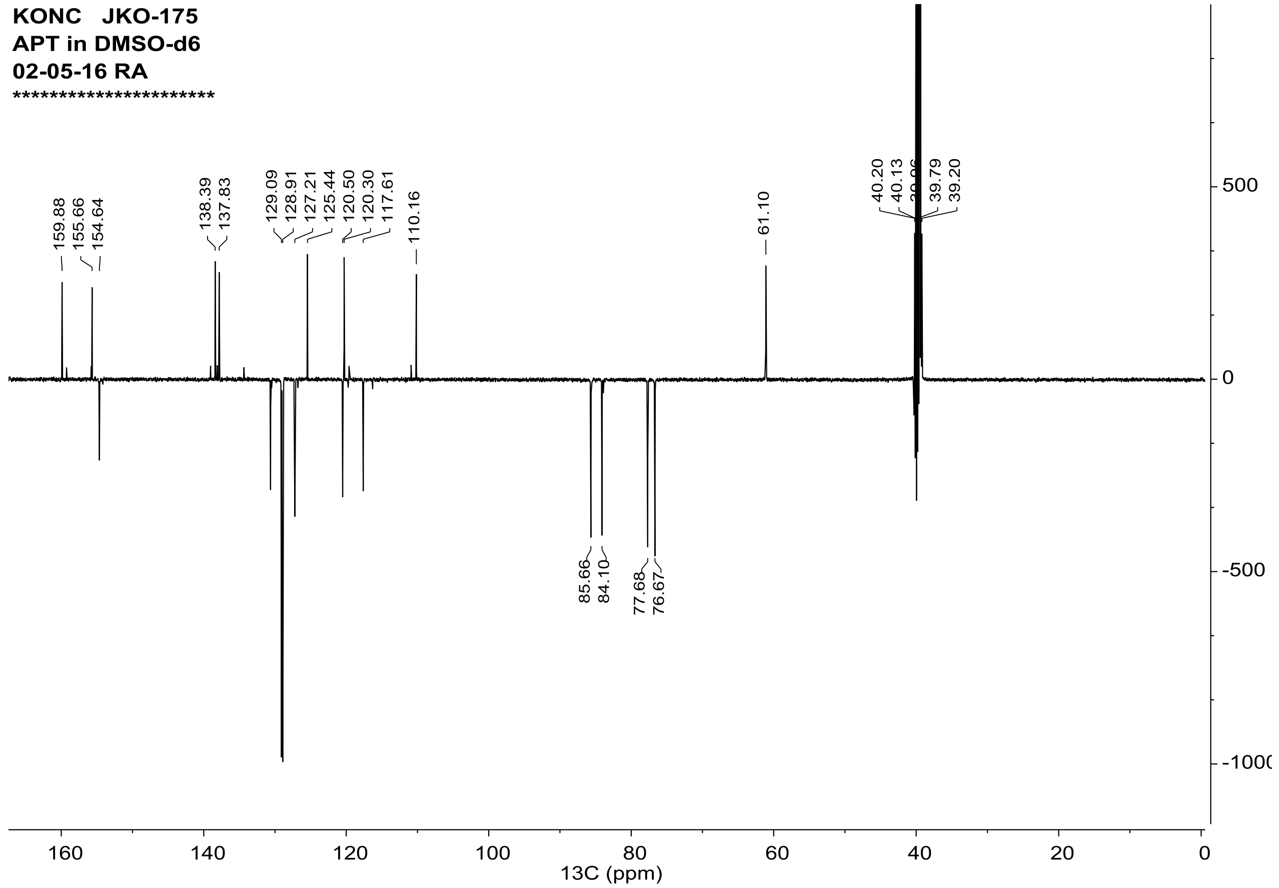
KONC JKO-175
APT in DMSO-d6
02-05-16 RA
****
159.88
155.66
154.64
138.39
137.83
129.09
128.91
127.21
125.44
120.50
120.30
117.61
110.16
61.10
40.20
40.13
39.79
39.20
85.66
84.10
77.68
76.67
13C (ppm)

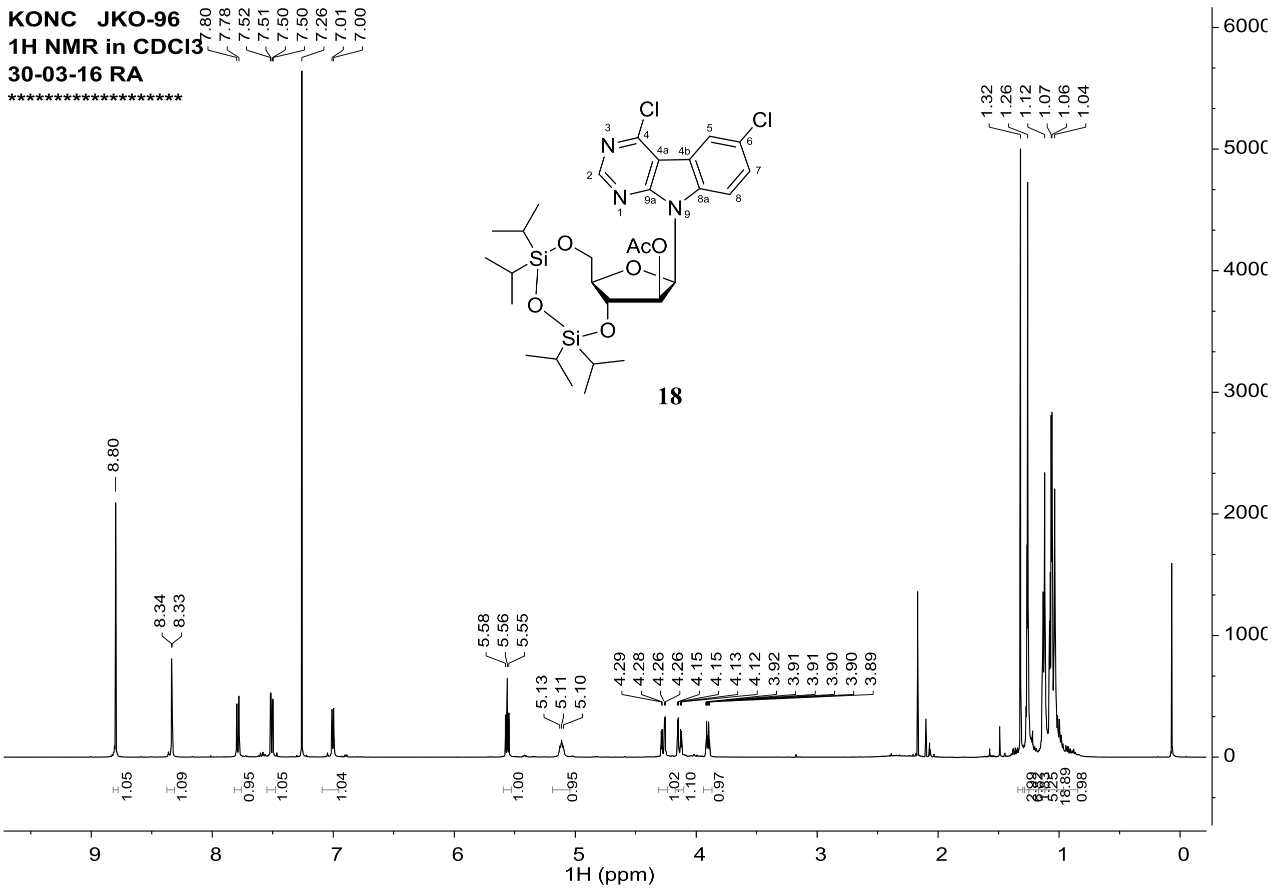
KONC JKO-96
1H NMR in CDCl3
30-03-16 RA
****
7.80 7.78 7.52 7.51 7.50 7.50 7.26 7.01 7.00
1.32 1.26 1.12 1.07 1.06 1.04
8.80
8.34 8.33
5.58 5.56 5.55
5.13 5.11 5.10
4.29 4.28 4.26 4.26 4.15 4.15 4.13 4.12 3.92 3.91 3.91 3.90 3.89
1.05 1.09 0.95 1.05 1.04 1.00 0.95 1.02 1.10 0.97 18.89 0.98
Cl
Cl
N
N
N
AcO
O
O
Si
O
Si
O
18
1H (ppm)

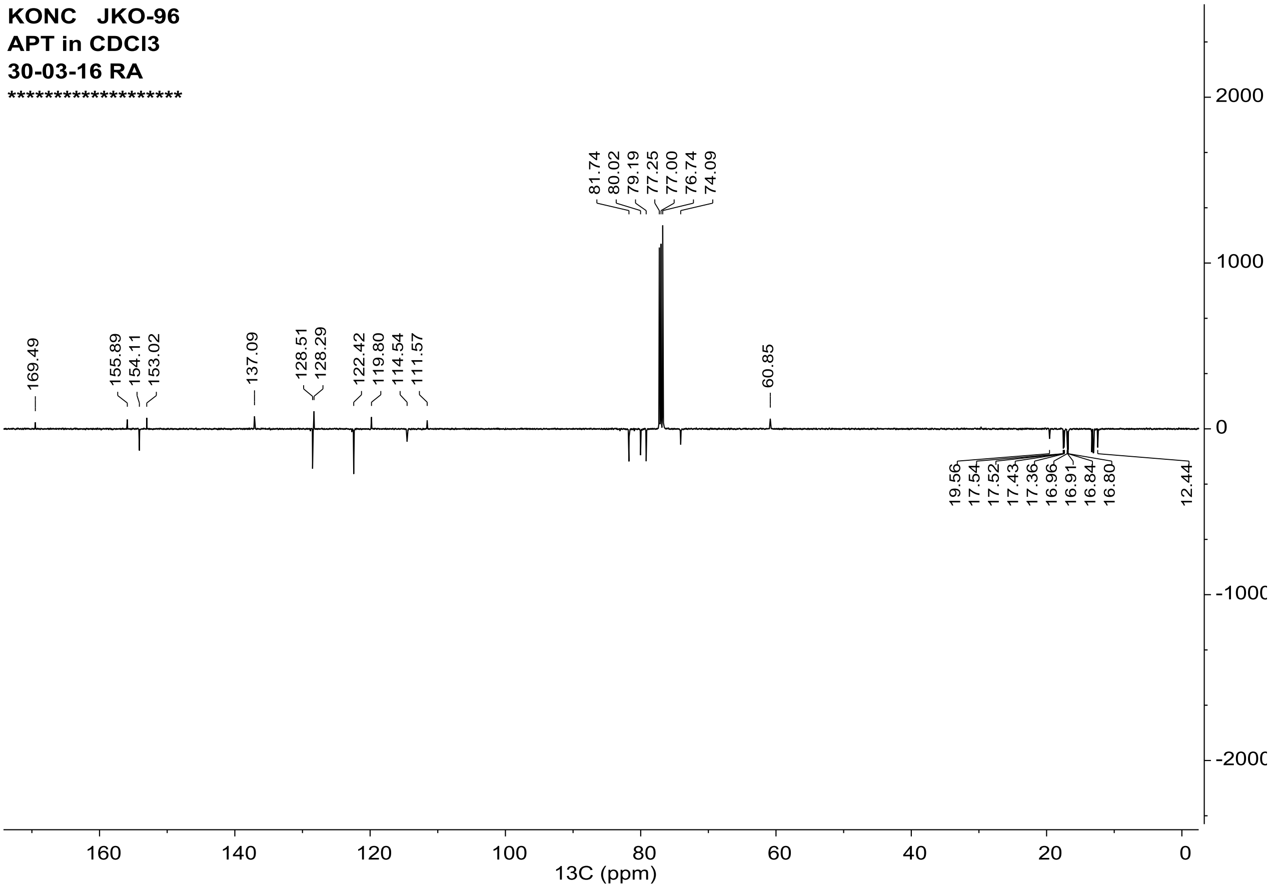
KONC JKO-96
APT in CDCl3
30-03-16 RA
****
81.74 80.02 79.19 77.25 77.00 76.74 74.09
169.49
155.89 154.11 153.02
137.09
128.51 128.29
122.42 119.80 114.54 111.57
60.85
19.56 17.54 17.52 17.43 17.36 16.96 16.91 16.84 16.80
12.44
13C (ppm)

KONC JKO-97
1H NMR in DMSO-d6
08-04-16 RA
****

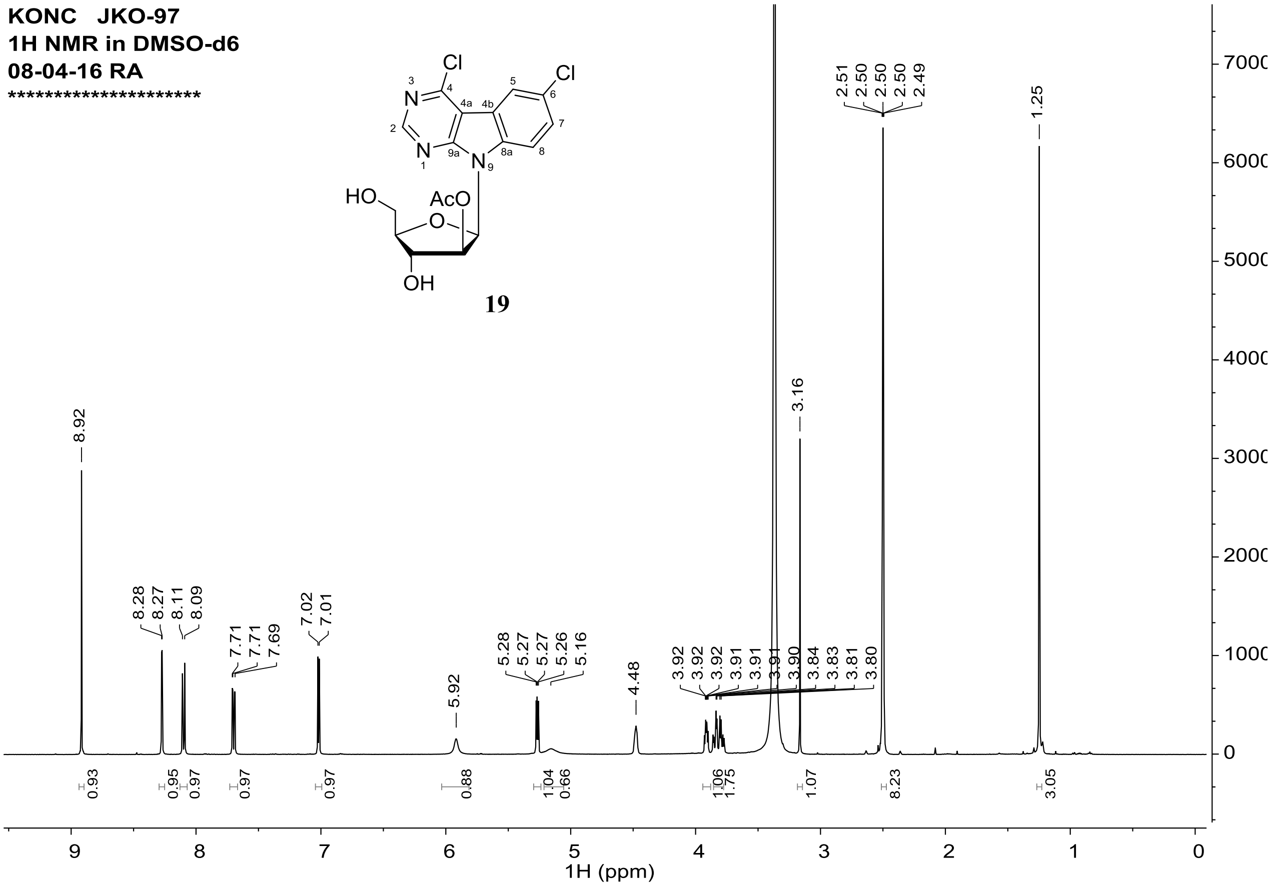

KONC JKO-97
APT in DMSO-d6
08-04-16 RA
****

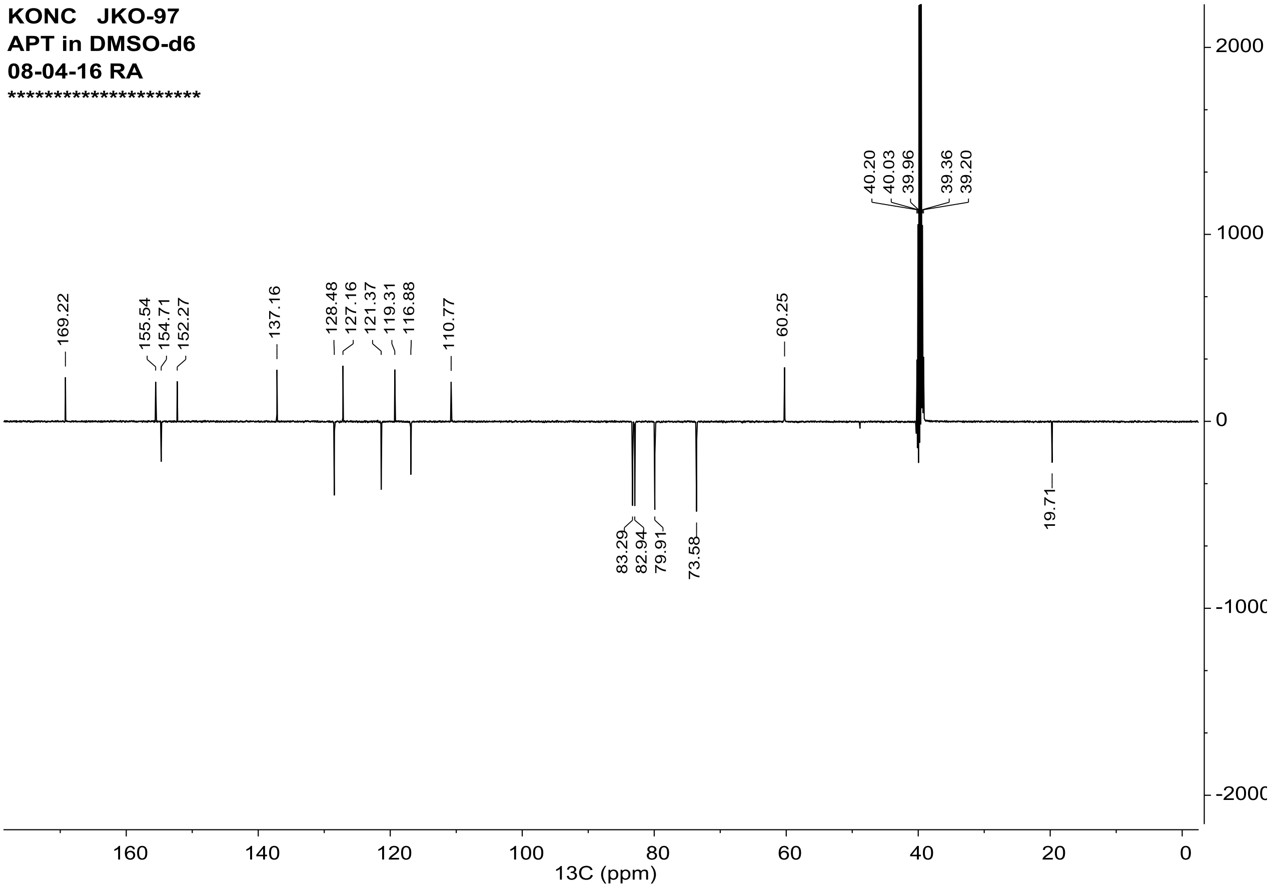

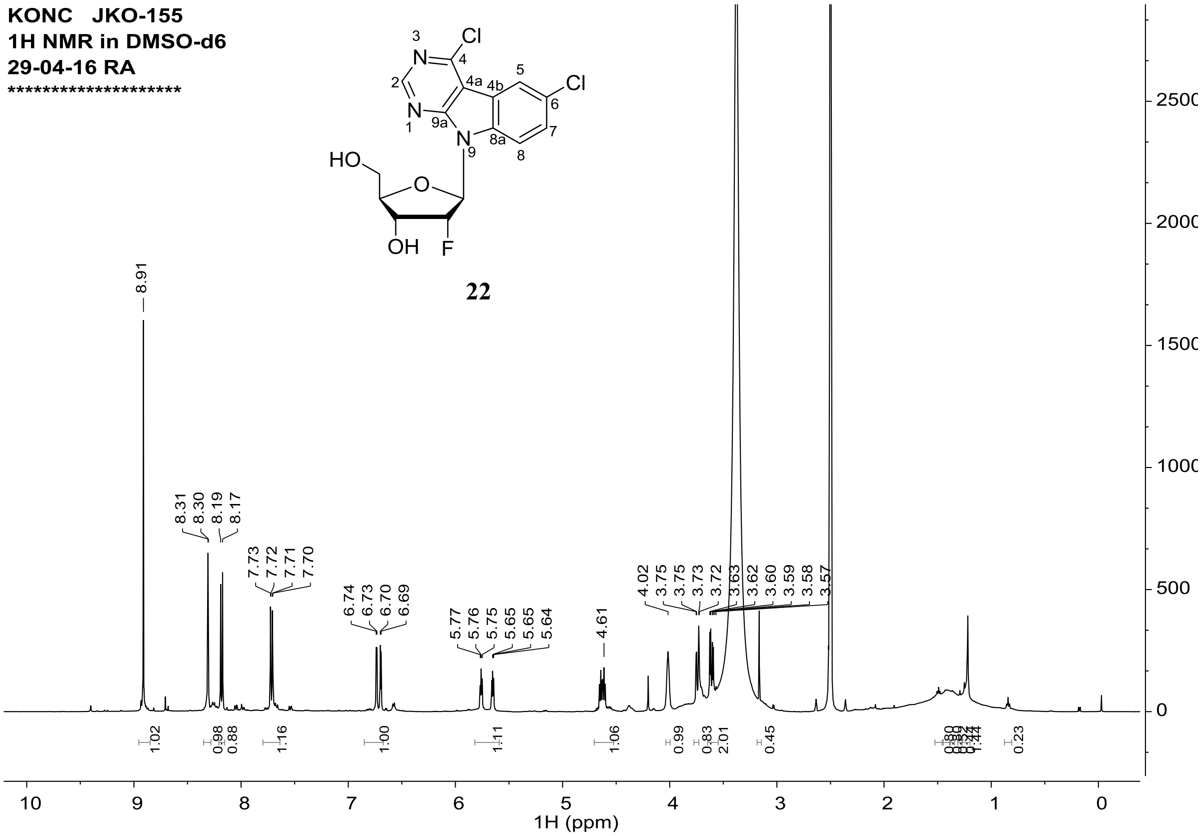
KONC JKO-155
1H NMR in DMSO-d6
29-04-16 RA
****
22
Cl
N
3
4
2
4a
4b
5
6
Cl
N
1
9a
8a
7
N
9
8
HO
O
OH
F
8.91
8.31
8.30
8.19
8.17
7.73
7.72
7.71
7.70
6.74
6.73
6.70
6.69
5.77
5.76
5.75
5.65
5.64
4.61
4.02
3.75
3.75
3.73
3.72
3.63
3.62
3.60
3.59
3.58
3.57
1.02
0.98
0.88
1.16
1.00
1.11
1.06
0.99
0.83
2.01
0.45
1.44
0.23
2500
2000
1500
1000
500
0
10
9
8
7
6
5
4
3
2
1
0
1H (ppm)

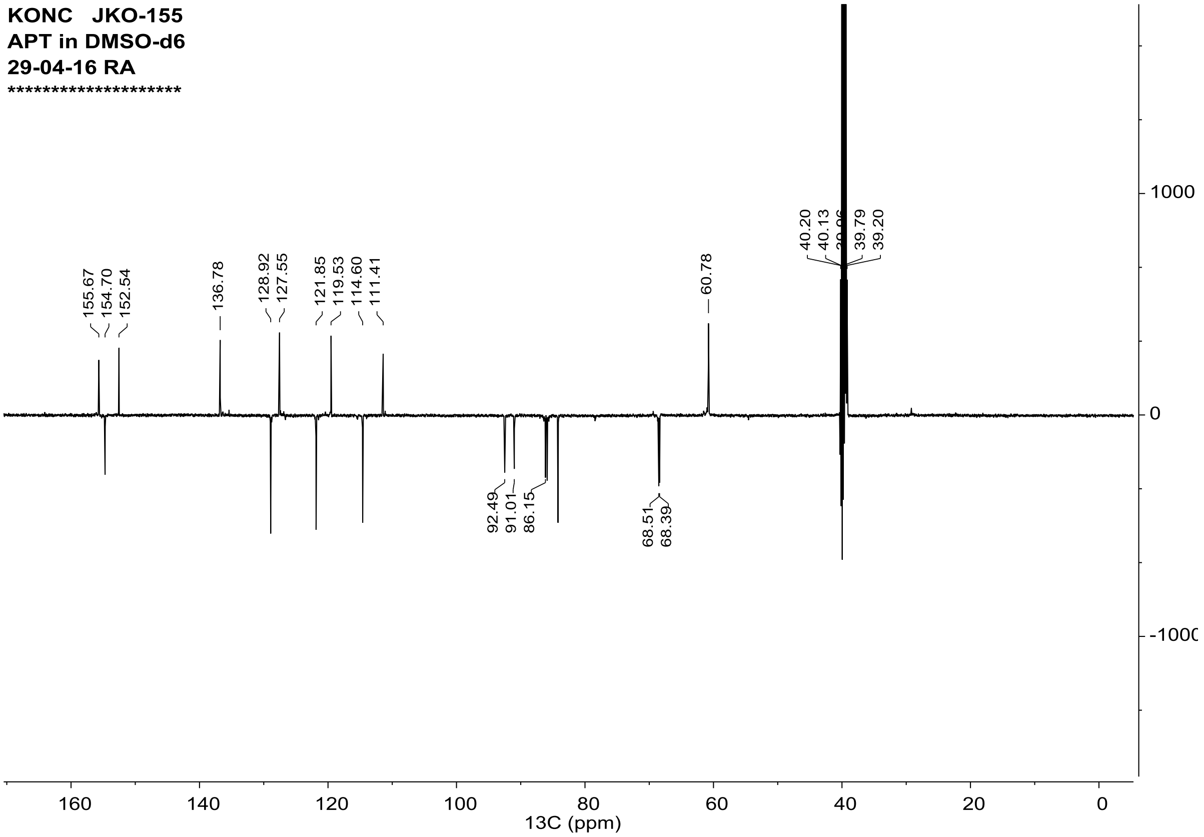
KONC JKO-155
APT in DMSO-d6
29-04-16 RA
****
155.67
154.70
152.54
136.78
128.92
127.55
121.85
119.53
114.60
111.41
60.78
40.20
40.13
39.79
39.20
92.49
91.01
86.15
68.51
68.39
1000
0
-1000
160
140
120
100
80
60
40
20
0
13C (ppm)

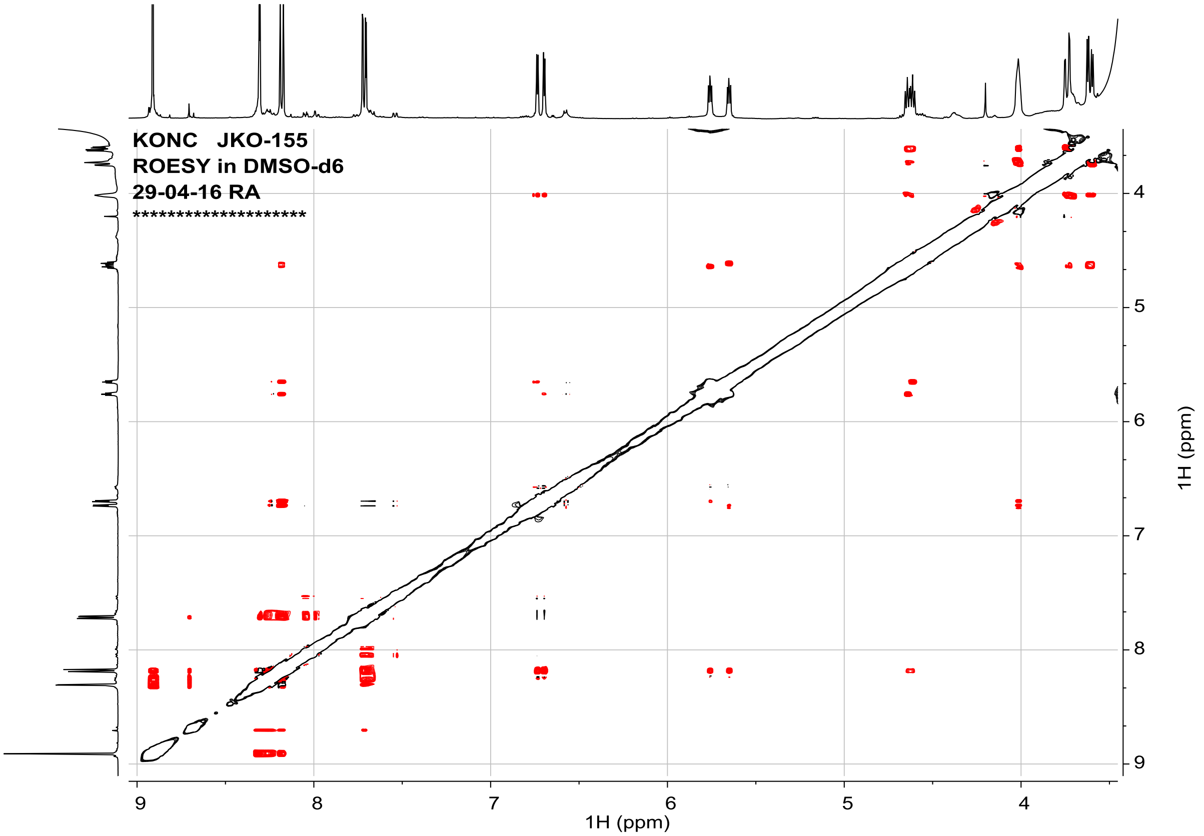
KONC JKO-155
ROESY in DMSO-d6
29-04-16 RA
1H (ppm)
1H (ppm)

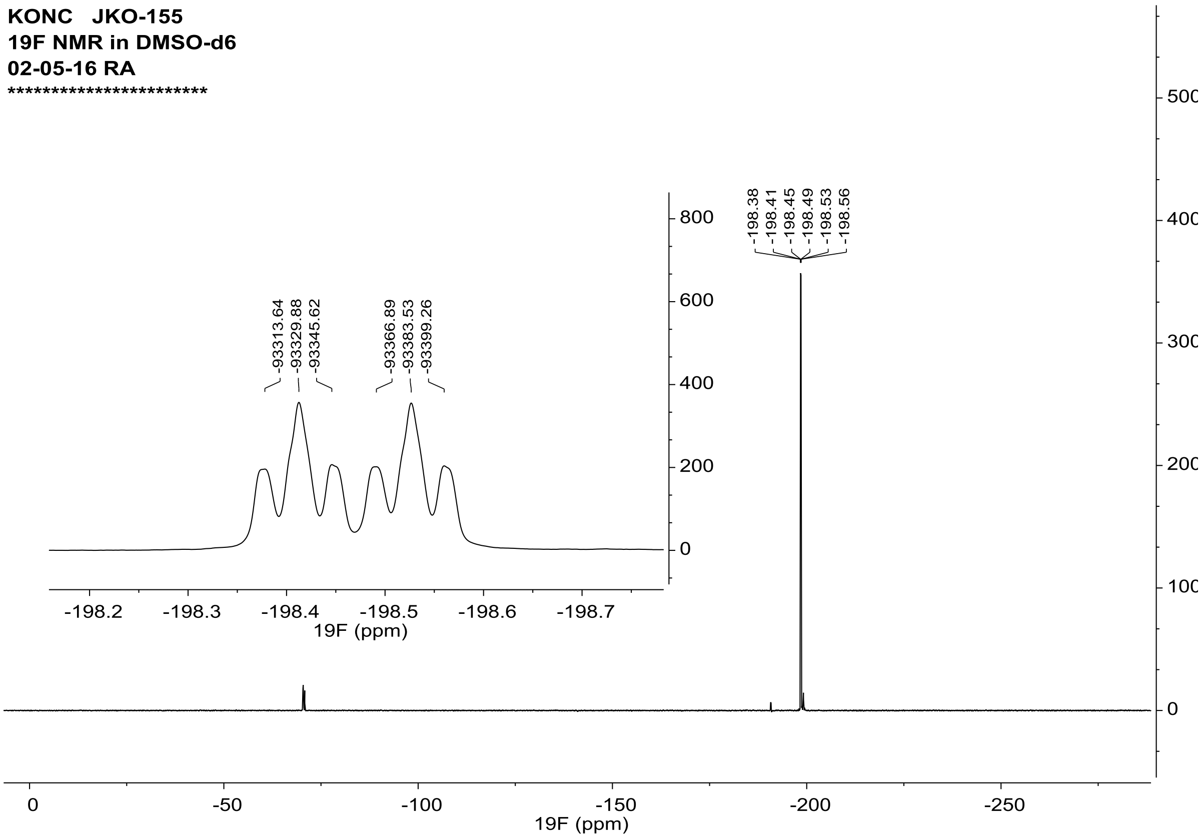
KONC JKO-155
19F NMR in DMSO-d6
02-05-16 RA
-198.38
-198.41
-198.45
-198.49
-198.53
-198.56
-93313.64
-93329.88
-93345.62
-93366.89
-93383.53
-93399.26
19F (ppm)
19F (ppm)

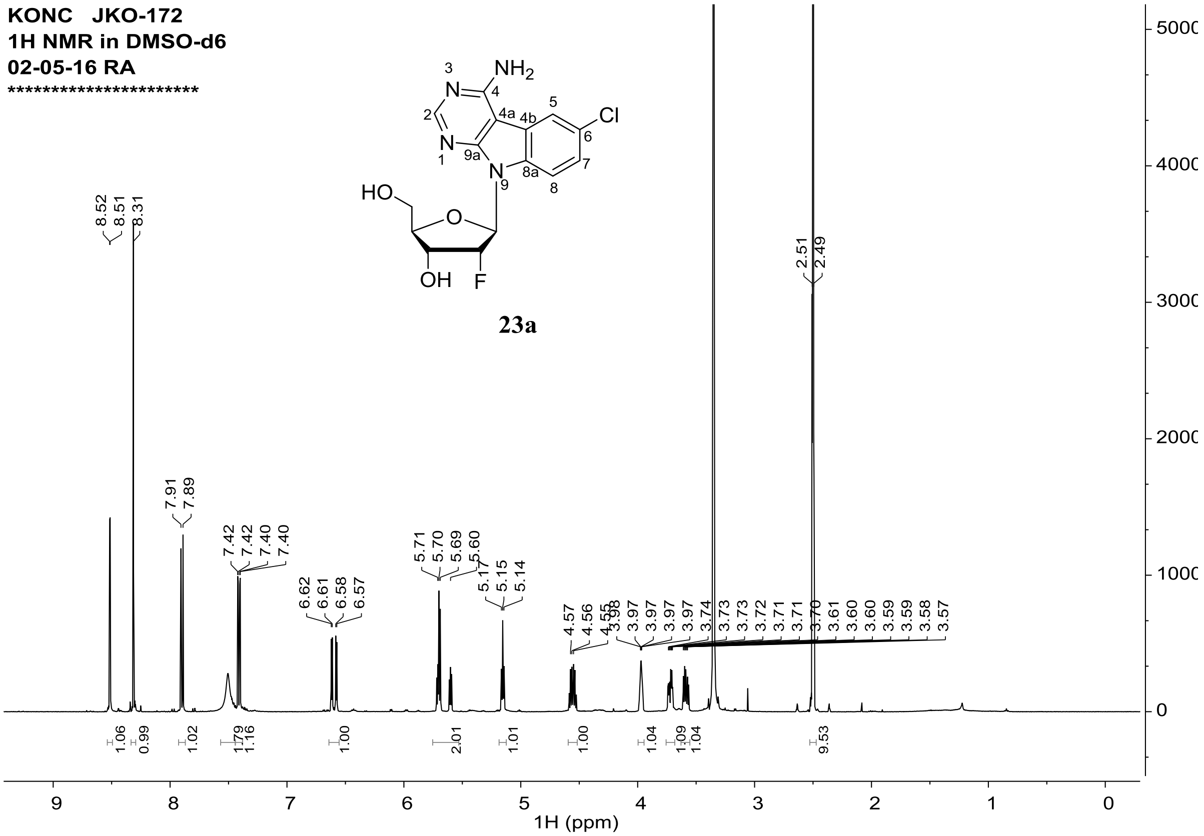
KONC JKO-172
1H NMR in DMSO-d6
02-05-16 RA
****
NH2
Cl
HO
O
OH
F
23a
8.52
8.51
8.31
7.91
7.89
7.42
7.42
7.40
7.40
6.62
6.61
6.58
6.57
5.71
5.70
5.69
5.60
5.17
5.15
5.14
4.57
4.56
4.55
3.98
3.97
3.97
3.97
3.97
3.74
3.73
3.73
3.72
3.71
3.71
3.70
3.61
3.60
3.60
3.59
3.59
3.58
3.57
2.51
2.49
1.06
0.99
1.02
1.79
1.16
1.00
2.01
1.01
1.00
1.04
1.09
1.04
9.53
1H (ppm)

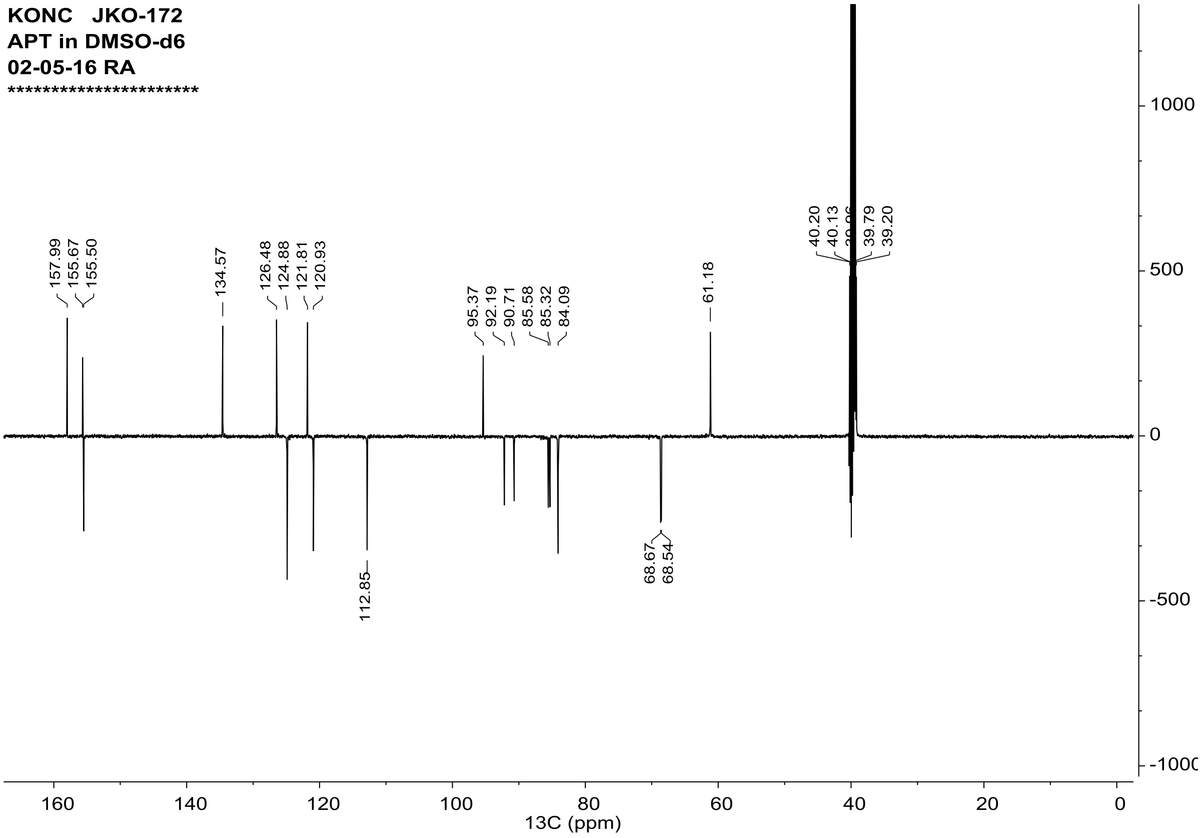
KONC JKO-172
APT in DMSO-d6
02-05-16 RA
****
157.99
155.67
155.50
134.57
126.48
124.88
121.81
120.93
112.85
95.37
92.19
90.71
85.58
85.32
84.09
68.67
68.54
61.18
40.20
40.13
39.79
39.20
13C (ppm)

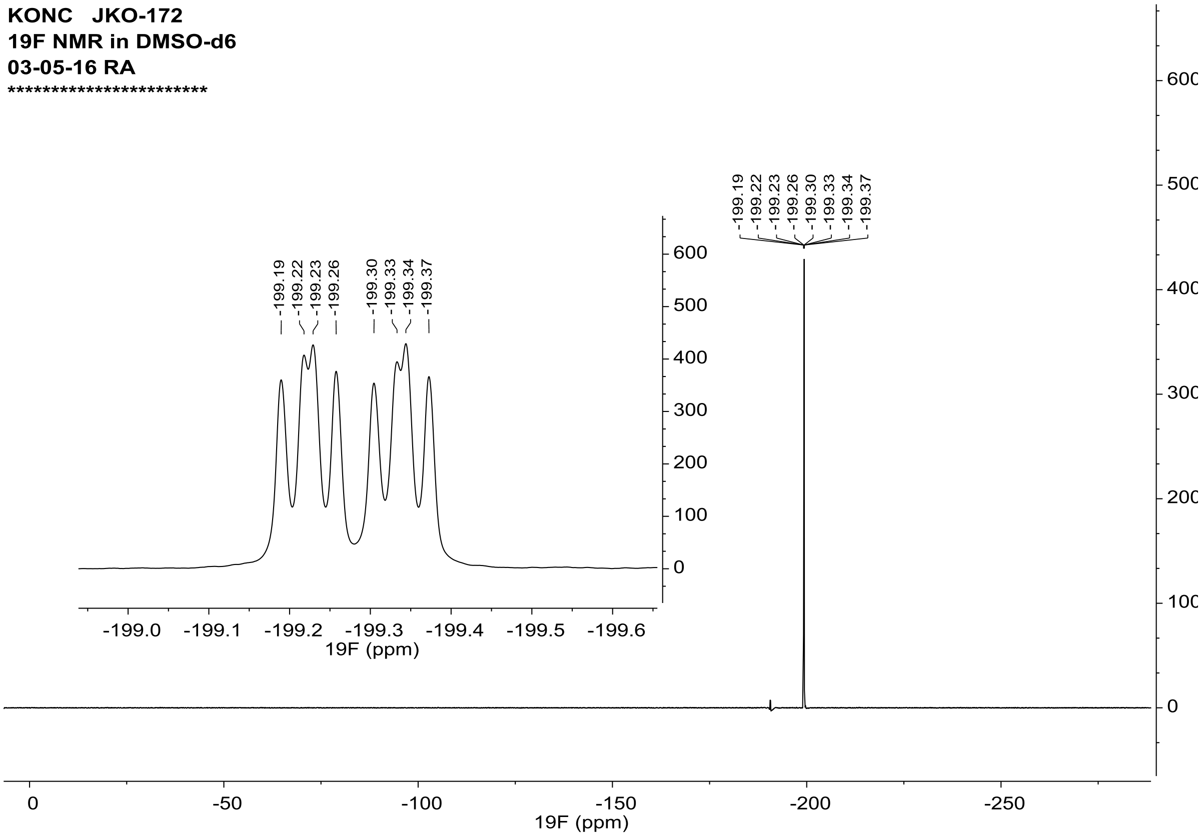
KONC JKO-172
19F NMR in DMSO-d6
03-05-16 RA
****
-199.19
-199.22
-199.23
-199.26
-199.30
-199.33
-199.34
-199.37
19F (ppm)

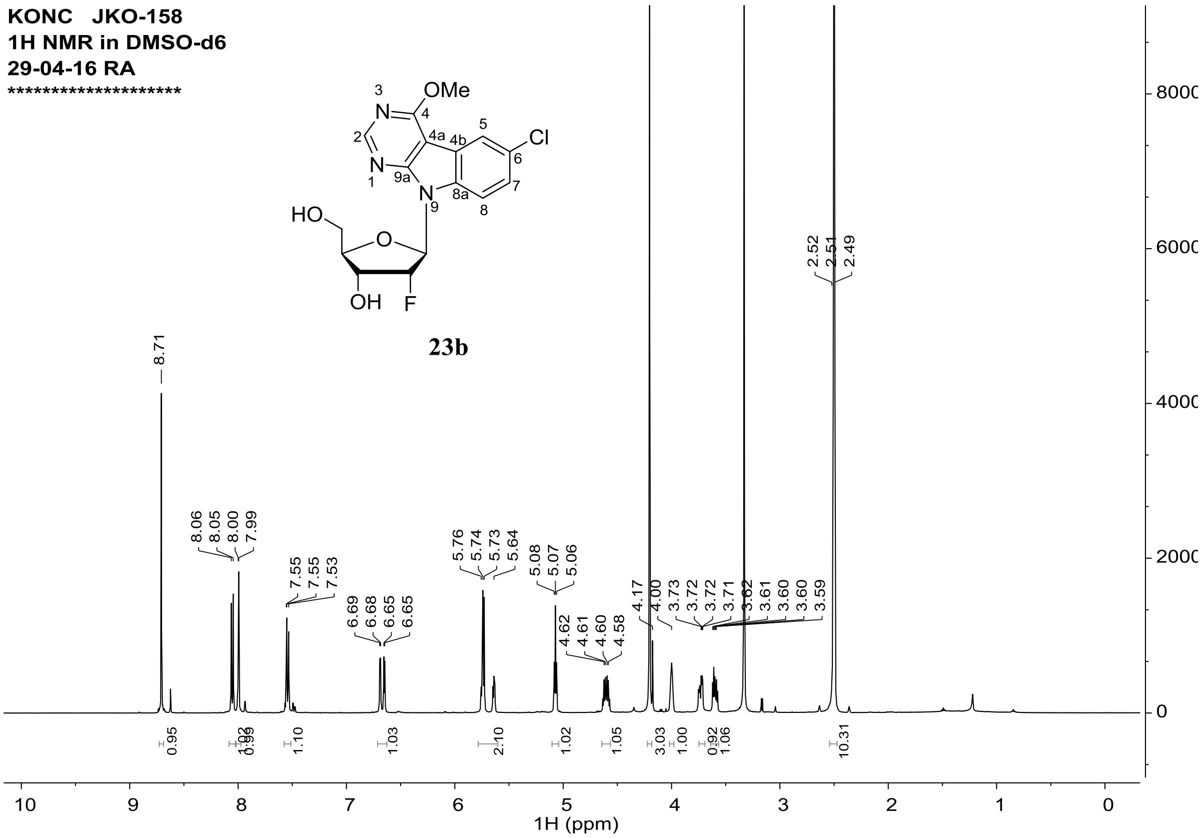
KONC JKO-158
1H NMR in DMSO-d6
29-04-16 RA
****
OMe
HO
OH
F
Cl
23b
8.71
8.06
8.05
8.00
7.99
7.55
7.55
7.53
6.69
6.68
6.65
6.65
5.76
5.74
5.73
5.64
5.08
5.07
5.06
4.62
4.61
4.60
4.58
4.17
4.00
3.73
3.72
3.72
3.71
3.62
3.61
3.60
3.60
3.59
2.52
2.51
2.49
1H (ppm)

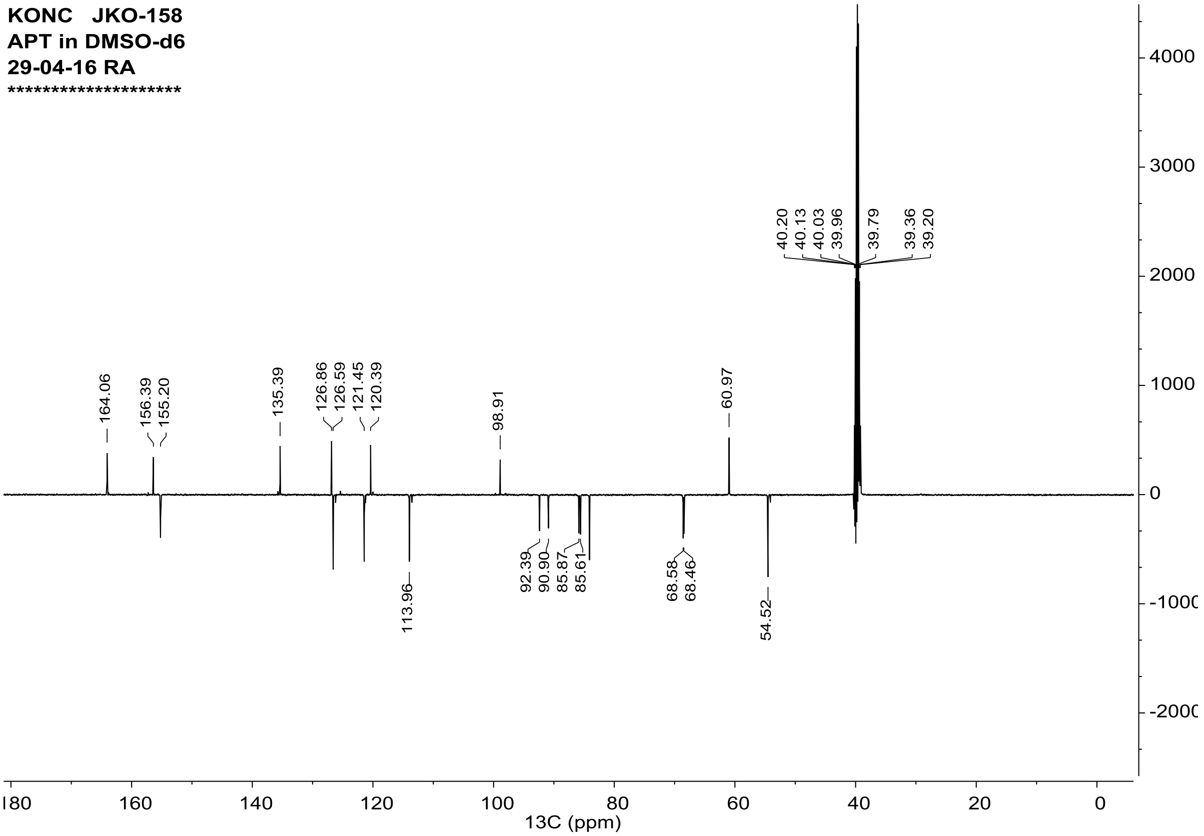
KONC JKO-158
APT in DMSO-d6
29-04-16 RA
****
164.06
156.39
155.20
135.39
126.86
126.59
121.45
120.39
98.91
60.97
40.20
40.13
40.03
39.96
39.79
39.36
39.20
113.96
92.39
90.90
85.87
85.61
68.58
68.46
54.52
13C (ppm)

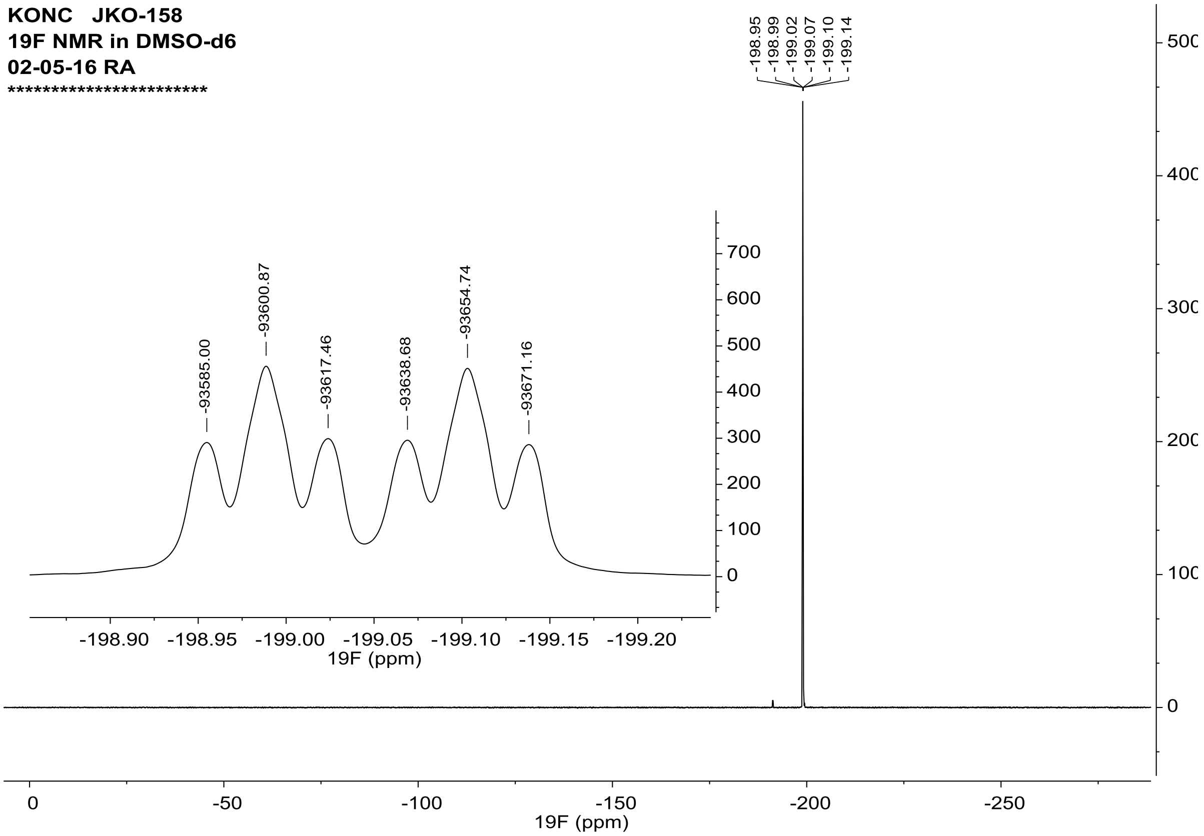
KONC JKO-158
19F NMR in DMSO-d6
02-05-16 RA
****
-198.95
-198.99
-199.02
-199.07
-199.10
-199.14
-93585.00
-93600.87
-93617.46
-93638.68
-93654.74
-93671.16
19F (ppm)
19F (ppm)

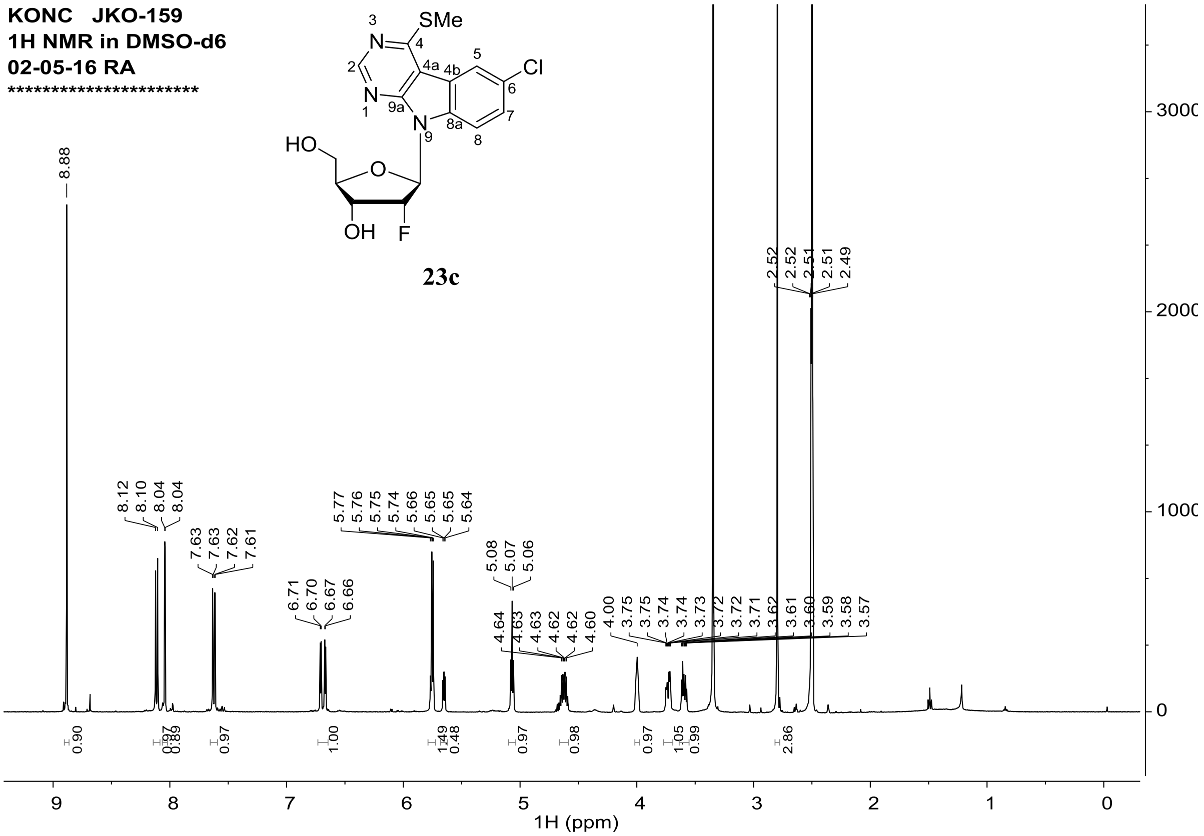
KONC JKO-159
1H NMR in DMSO-d6
02-05-16 RA
****
SMe
Cl
HO
OH
F
1H (ppm)

23c

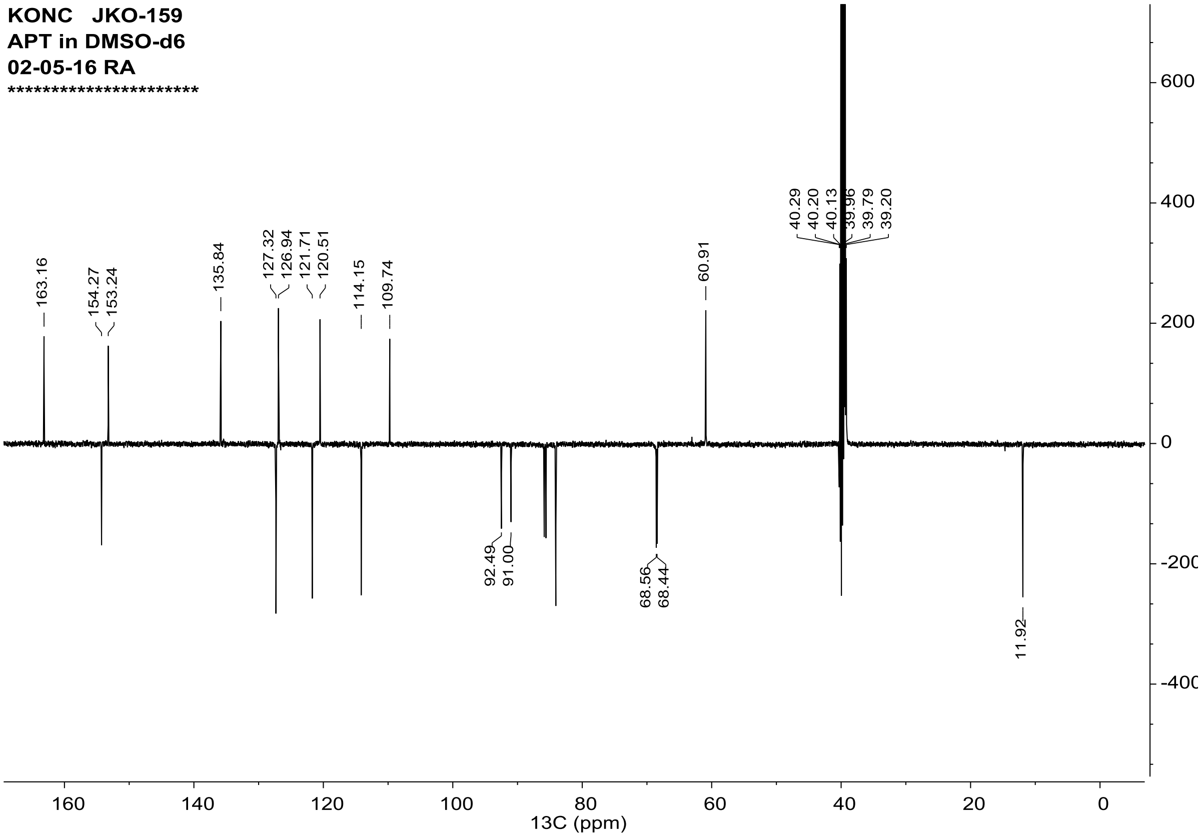
KONC JKO-159
APT in DMSO-d6
02-05-16 RA
****
13C (ppm)

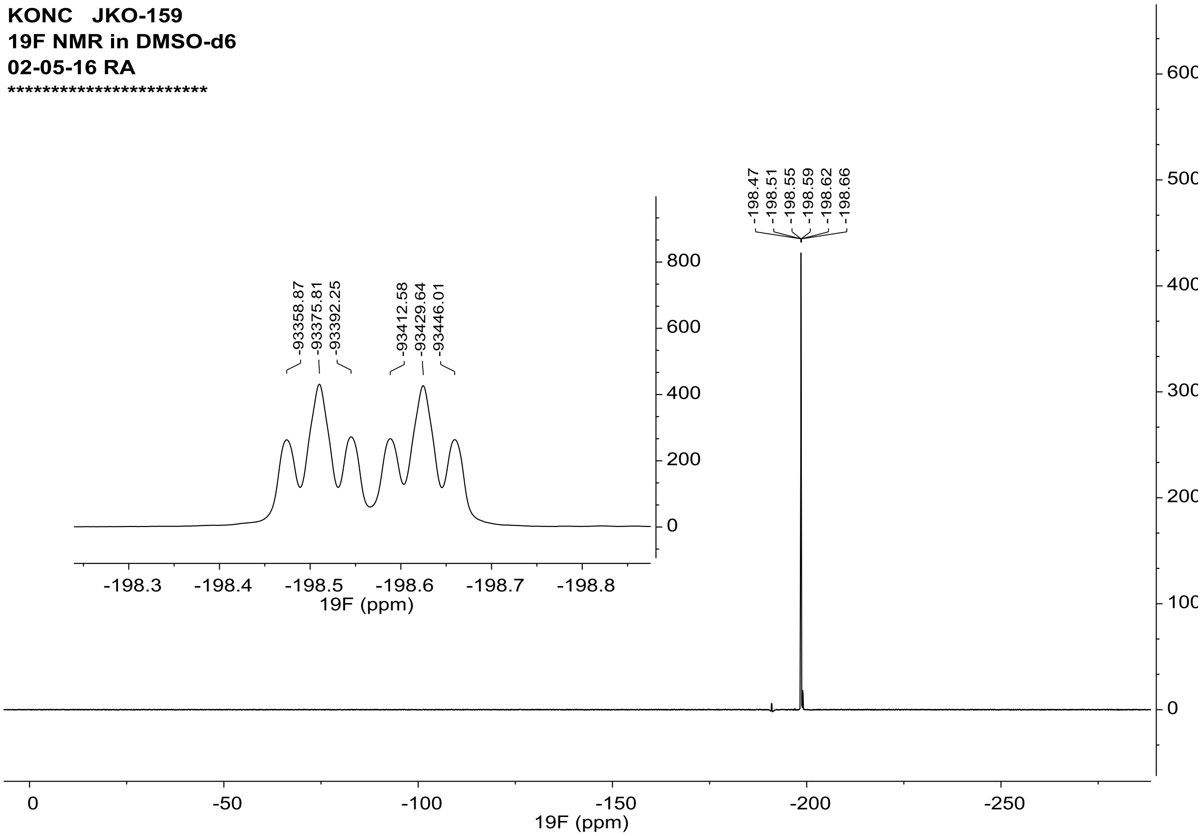
KONC JKO-159
19F NMR in DMSO-d6
02-05-16 RA
****
-198.47
-198.51
-198.55
-198.59
-198.62
-198.66
-93358.87
-93375.81
-93392.25
-93412.58
-93429.64
-93446.01
-198.3 -198.4 -198.5 -198.6 -198.7 -198.8
19F (ppm)
0 -50 -100 -150 -200 -250
19F (ppm)

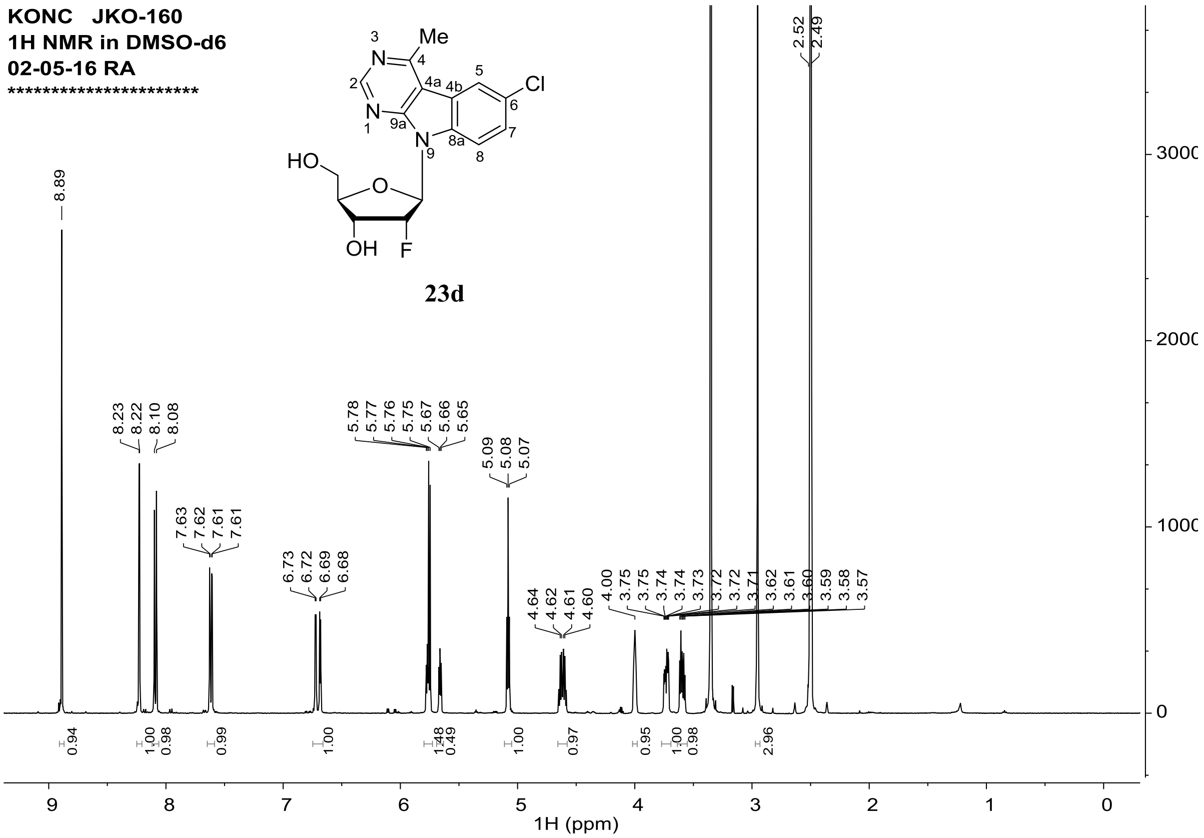
KONC JKO-160
1H NMR in DMSO-d6
02-05-16 RA
****
Me
N
N
N
Cl
HO
O
OH
F
23d
8.89
8.23
8.22
8.10
8.08
7.63
7.62
7.61
7.61
6.73
6.72
6.69
6.68
5.78
5.77
5.76
5.75
5.67
5.66
5.65
5.09
5.08
5.07
4.64
4.62
4.61
4.60
4.00
3.75
3.75
3.74
3.74
3.73
3.72
3.72
3.71
3.62
3.61
3.60
3.59
3.58
3.57
2.52
2.49
0.94
1.00
0.98
0.99
1.00
1.48
0.49
1.00
0.97
0.95
1.00
0.98
2.96
9 8 7 6 5 4 3 2 1 0
1H (ppm)

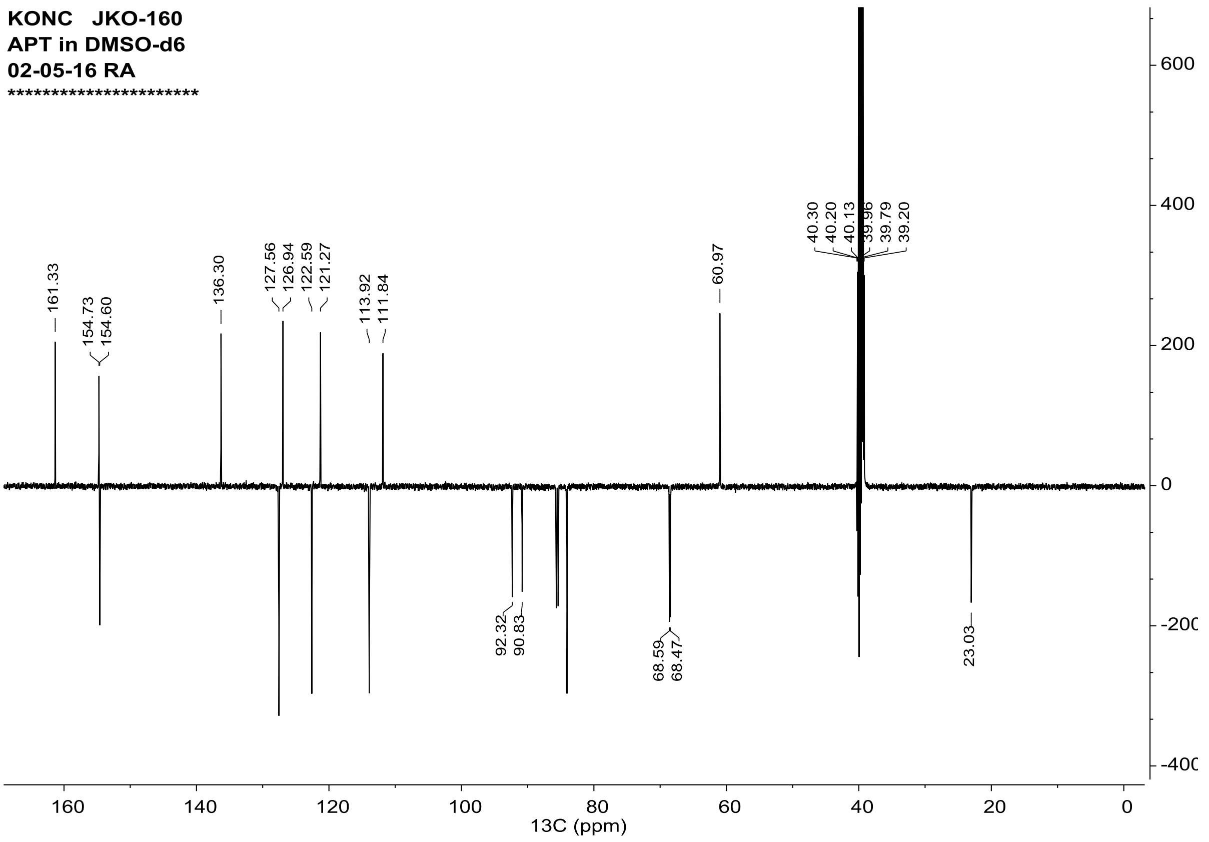
KONC JKO-160
APT in DMSO-d6
02-05-16 RA
****
161.33
154.73
154.60
136.30
127.56
126.94
122.59
121.27
113.92
111.84
92.32
90.83
68.59
68.47
60.97
40.30
40.20
40.13
39.96
39.79
39.20
23.03
13C (ppm)

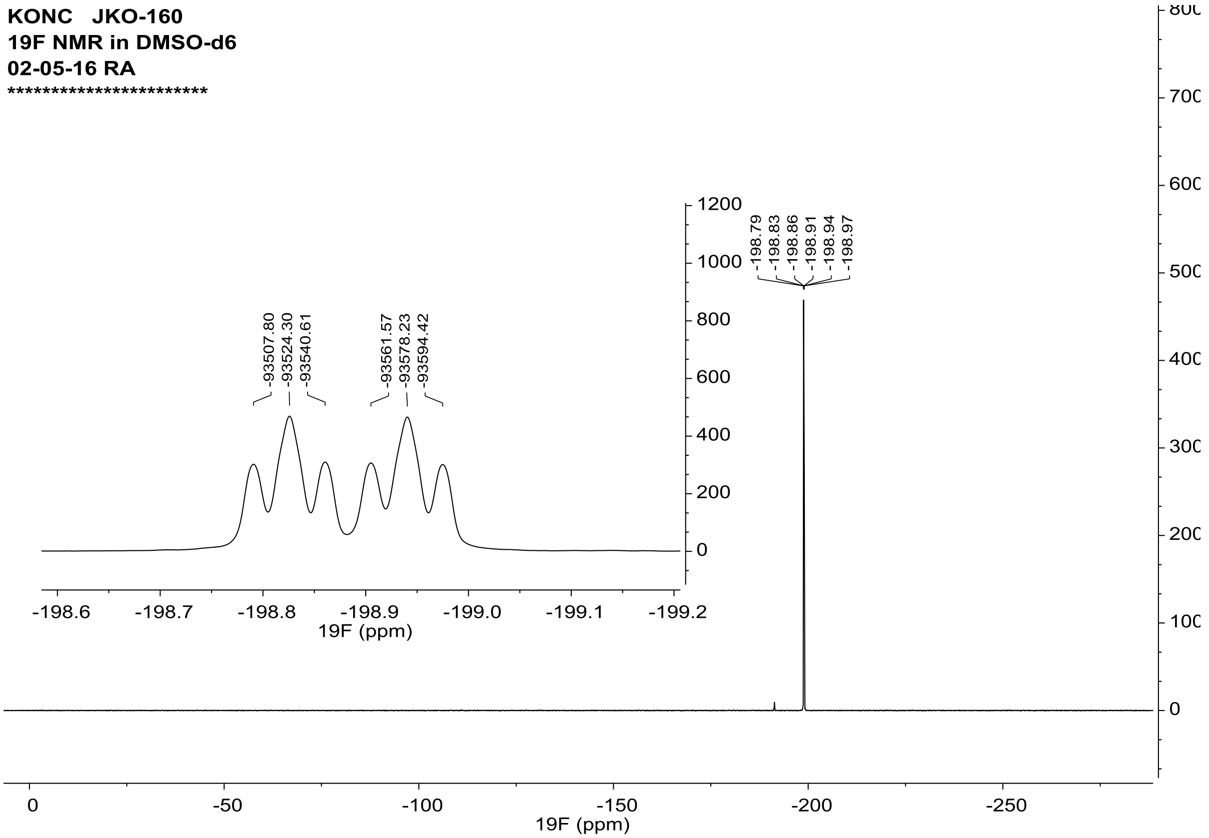
KONC JKO-160
19F NMR in DMSO-d6
02-05-16 RA
****
-93507.80
-93524.30
-93540.61
-93561.57
-93578.23
-93594.42
-198.79
-198.83
-198.86
-198.91
-198.94
-198.97
19F (ppm)
19F (ppm)

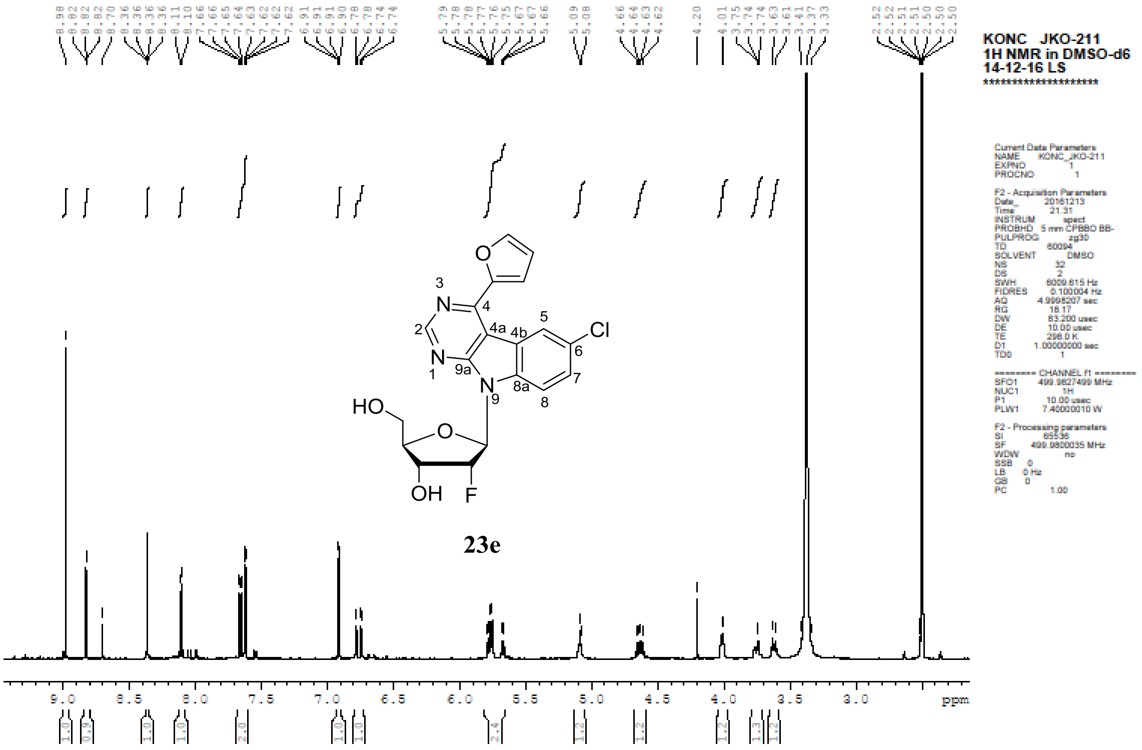
KONC JKO-211
1H NMR in DMSO-d6
14-12-16 LS
23e
ppm

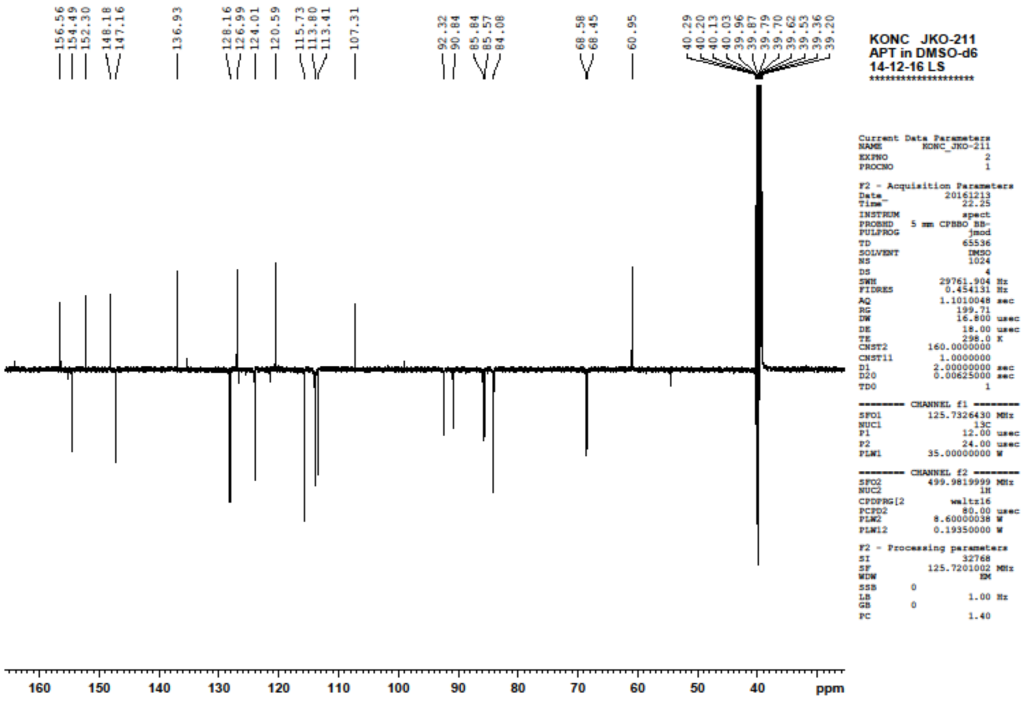
KONC JKO-211
APT in DMSO-d6
14-12-16 LS
ppm

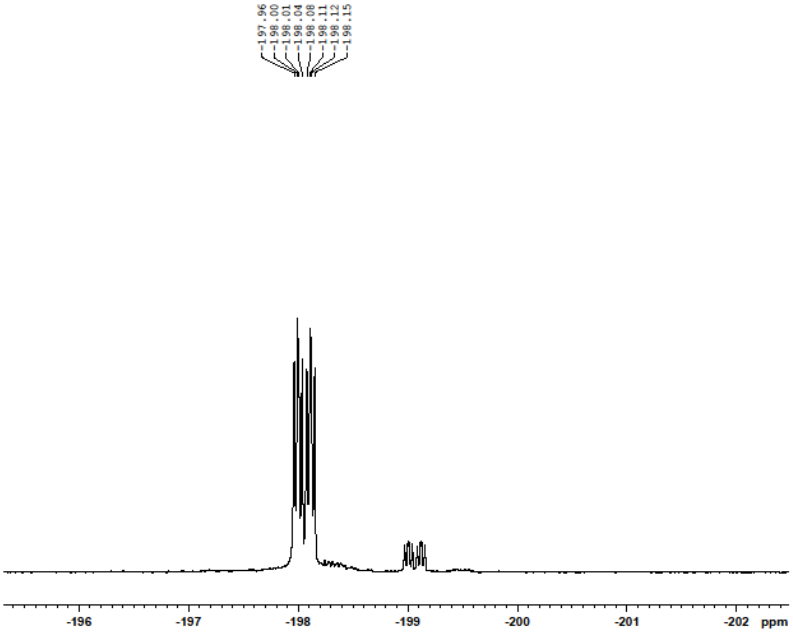

KONC JKO-211
19F NMR in DMSO-d6
19-12-16 RA
****

```
Current Data Parameters
NAME          KONC_JKO-211
EXPNO                   19
PROCNO                   1

F2 - Acquisition Parameters
Date_             20161219
Time                 13.35
INSTRUM              spect
PROBHD    5 mm CPBBO BB-
PULPROG             zgflqn
TD                  277762
SOLVENT               DMSO
NS                      32
DS                       0
SWH          138888.891 Hz
FIDRES         0.500028 Hz
AQ            0.9999432 sec
RG                  199.71
DW                3.600 usec
DE               18.00 usec
TE                300.0 K
D1           1.00000000 sec
TD0                      1

======== CHANNEL f1 ========
SFO1        470.3830206 MHz
NUC1                   19F
P1               15.00 usec
PLW1         6.40000010 W

F2 - Processing parameters
SI                  524288
SF          470.4493301 MHz
WDW                     EM
SSB       0
LB                 1.00 Hz
GB        0
PC                    1.00
```

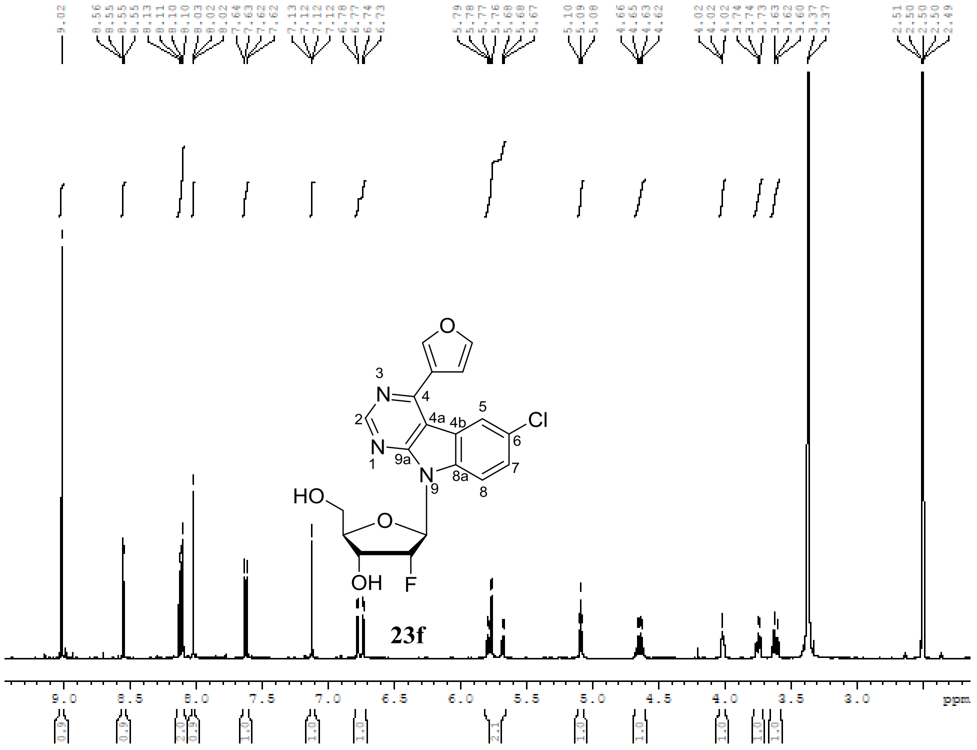

KONC JKO-209
1H NMR in DMSO-d6
14-12-16 LS
****

```
Current Data Parameters
NAME       KONC_JKO-209
EXPNO              1
PROCNO             1

F2 - Acquisition Parameters
Date_         20161213
Time            17.36
INSTRUM         spect
PROBHD    5 mm CPBBO BB-
PULPROG          zg30
TD              60094
SOLVENT          DMSO
NS                 32
DS                  2
SWH          6009.615 Hz
FIDRES       0.100004 Hz
AQ          4.9998207 sec
RG               11.5
DW            83.200 usec
DE             10.00 usec
TE             298.0 K
D1        1.00000000 sec
TD0                 1

======== CHANNEL f1 ========
SFO1      499.9827499 MHz
NUC1               1H
P1             10.00 usec
PLW1        7.40000010 W

F2 - Processing parameters
SI              65536
SF        499.9800035 MHz
WDW                no
SSB      0
LB       0 Hz
GB       0
PC               1.00
```

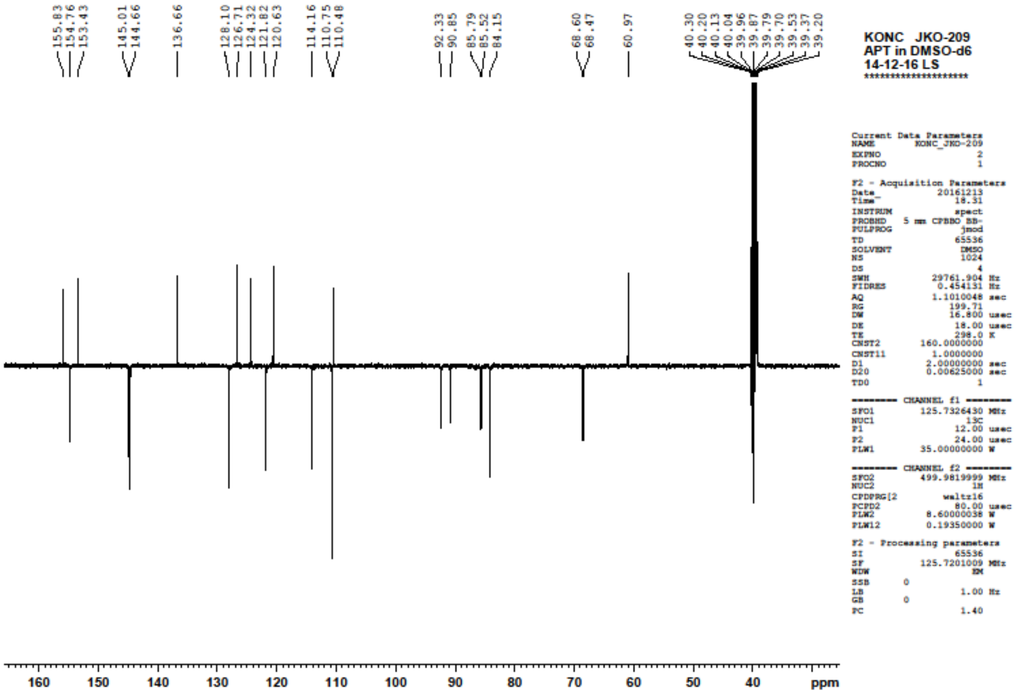
KONC JKO-209
APT in DMSO-d6
14-12-16 LS
155.83
154.76
153.43
145.01
144.66
136.66
128.10
126.71
124.32
121.82
120.63
114.16
110.75
110.48
92.33
90.85
85.79
85.52
84.15
68.60
68.47
60.97
40.30
40.20
40.13
40.04
39.96
39.87
39.79
39.70
39.53
39.37
39.20
ppm

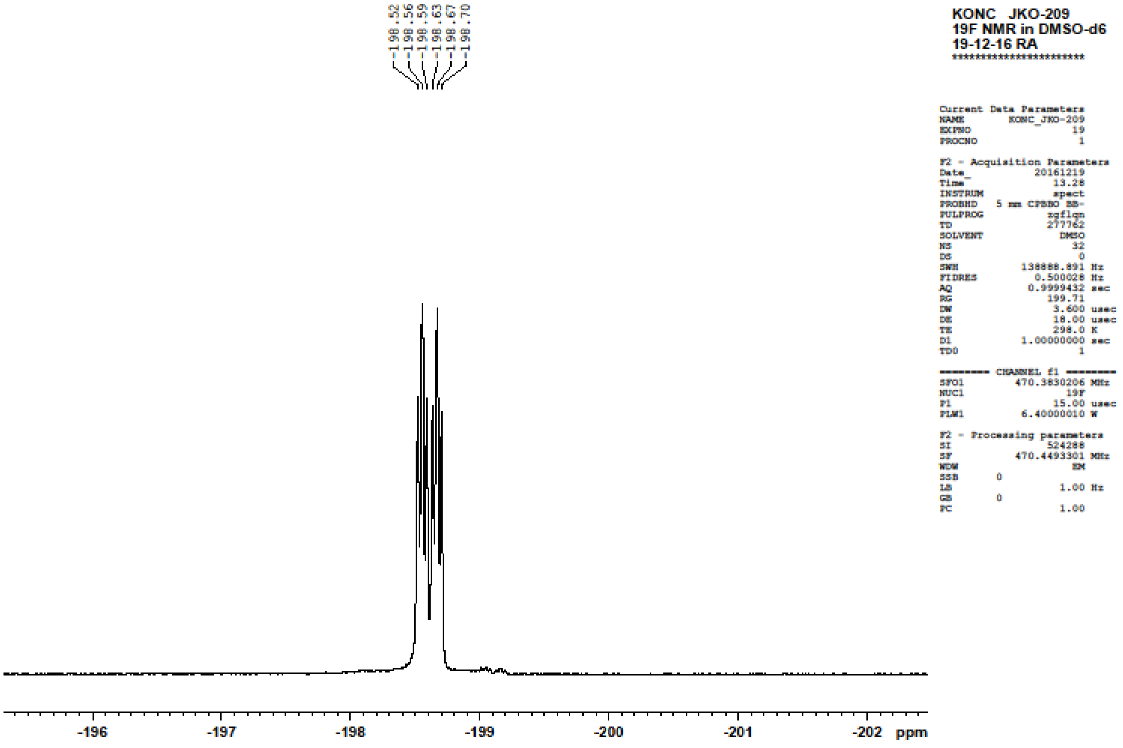
KONC JKO-209
19F NMR in DMSO-d6
19-12-16 RA
-198.52
-198.56
-198.59
-198.63
-198.67
-198.70
-196
-197
-198
-199
-200
-201
-202
ppm

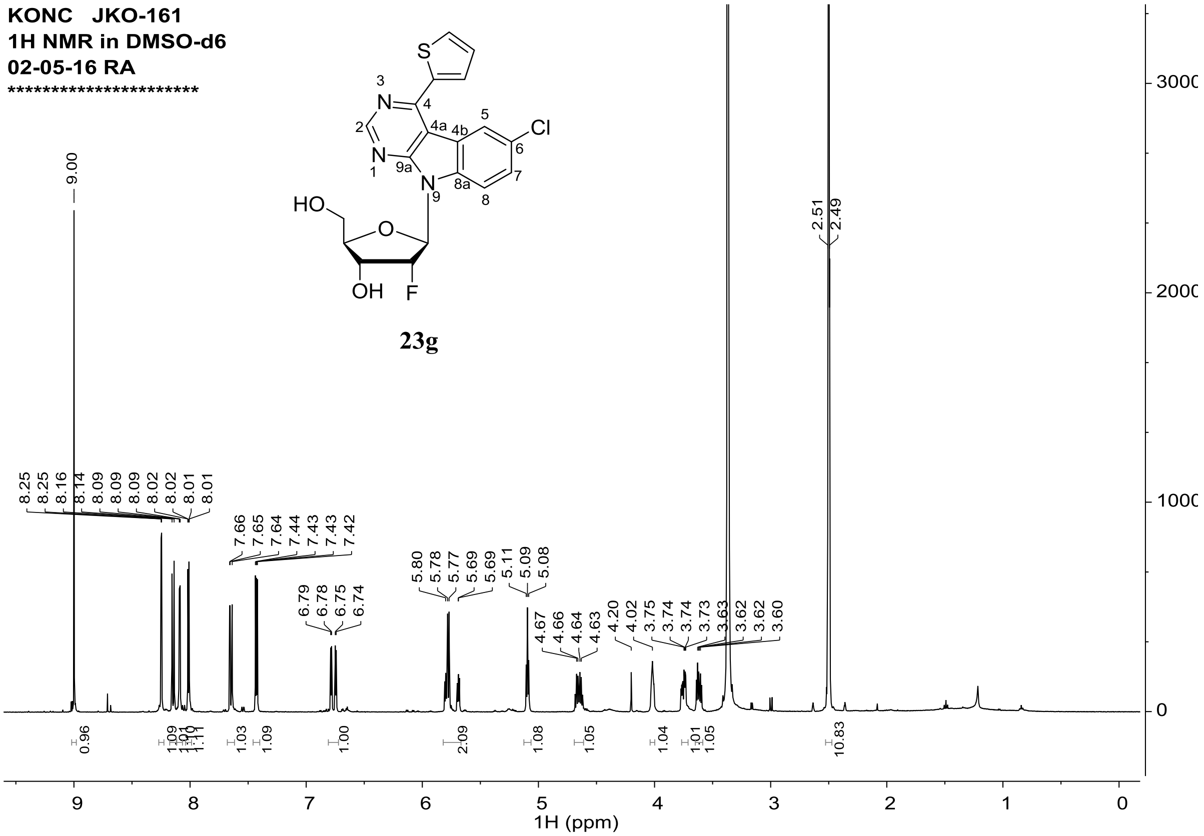
KONC JKO-161
1H NMR in DMSO-d6
02-05-16 RA
****
23g
9.00
8.25 8.25 8.16 8.14 8.09 8.09 8.09 8.02 8.02 8.01 8.01
7.66 7.65 7.64 7.44 7.43 7.43 7.42
6.79 6.78 6.75 6.74
5.80 5.78 5.77 5.69 5.69 5.11 5.09 5.08
4.67 4.66 4.64 4.63 4.20 4.02 3.75 3.74 3.74 3.73 3.63 3.62 3.62 3.60
2.51 2.49
0.96 1.09 1.01 1.10 1.11 1.03 1.09 1.00 2.09 1.08 1.05 1.04 1.01 1.05 10.83
1H (ppm)

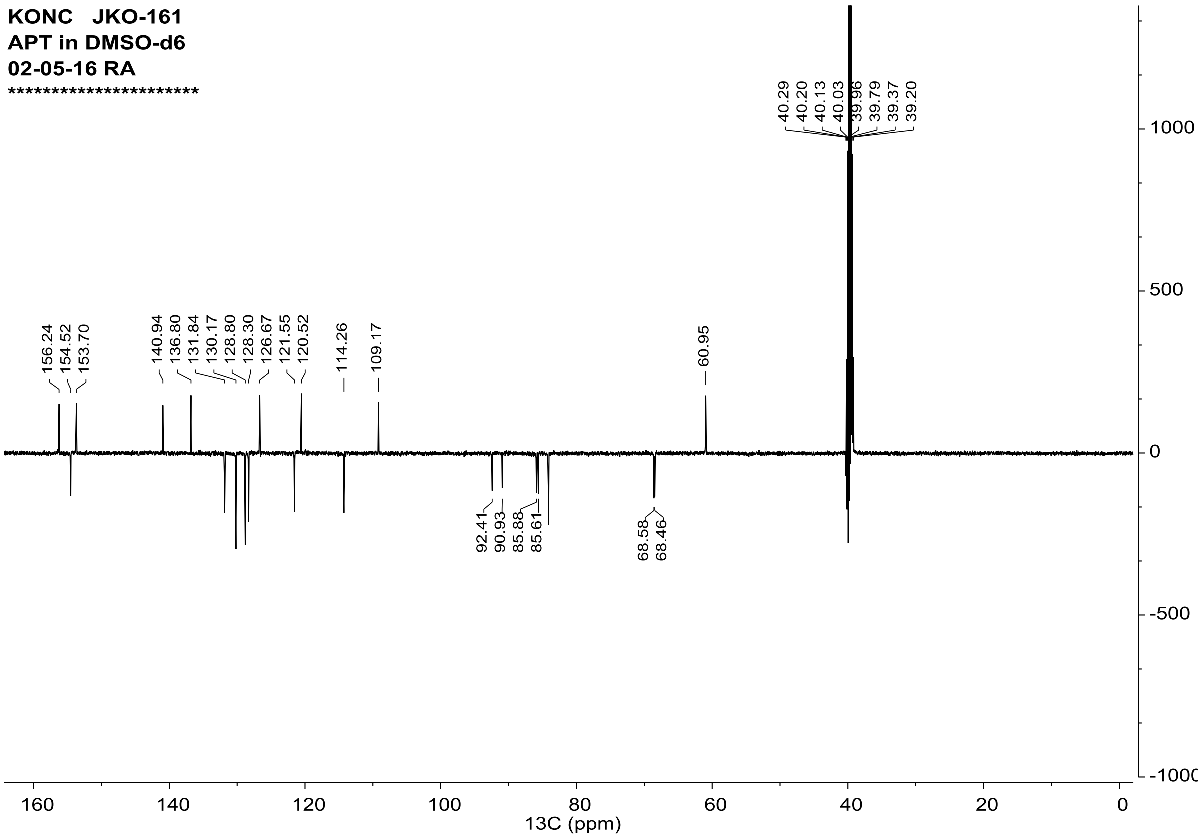
KONC JKO-161
APT in DMSO-d6
02-05-16 RA
****
156.24 154.52 153.70 140.94 136.80 131.84 130.17 128.80 128.30 126.67 121.55 120.52 114.26 109.17 60.95
40.29 40.20 40.13 40.03 39.96 39.79 39.37 39.20
92.41 90.93 85.88 85.61 68.58 68.46
13C (ppm)

KONC JKO-161
19F NMR in DMSO-d6
02-05-16 RA
****

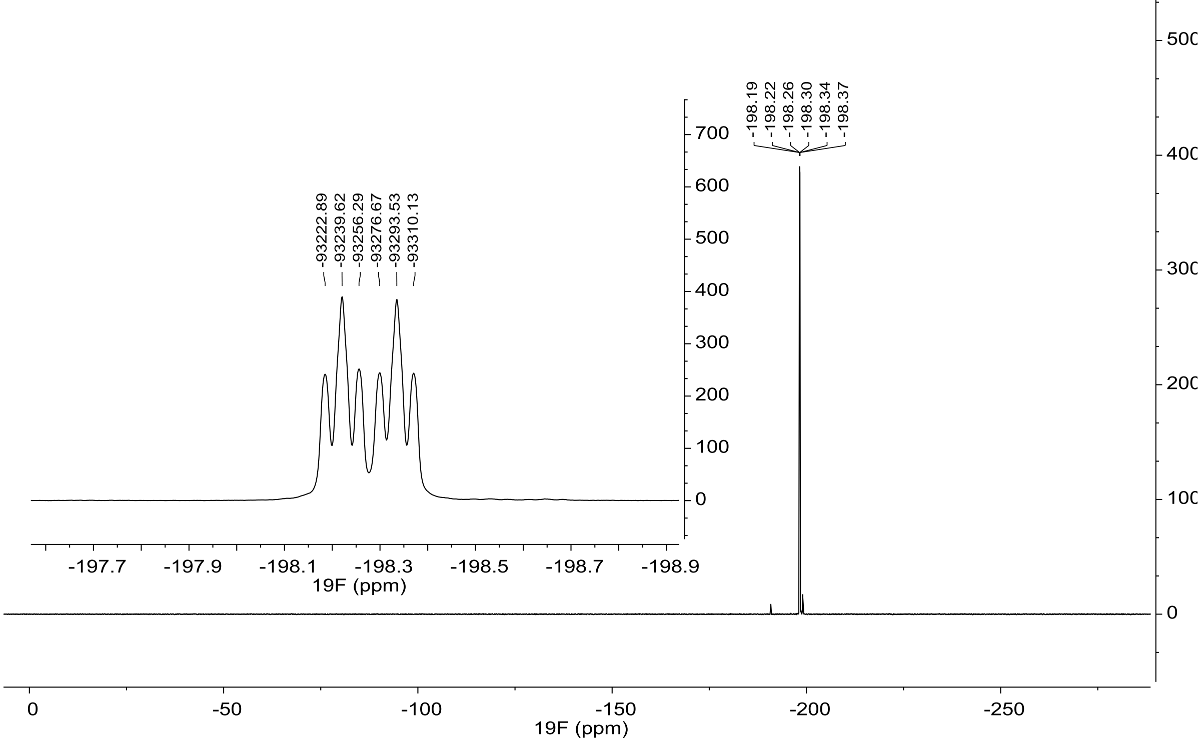

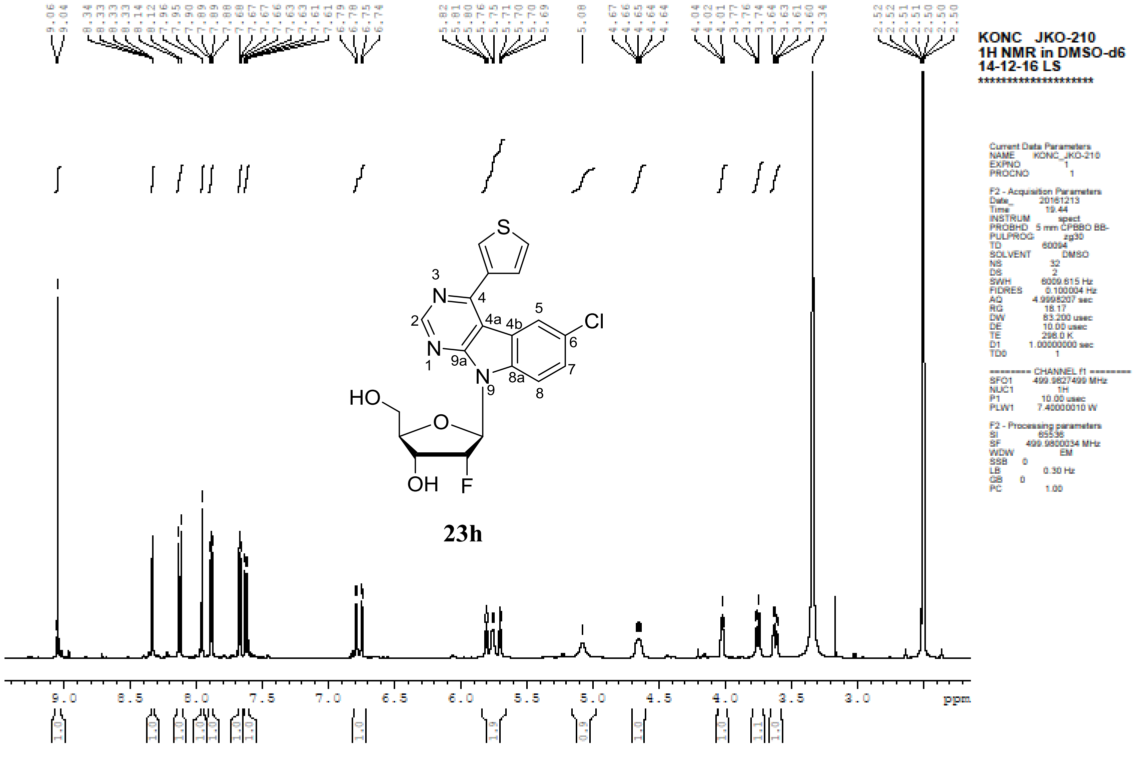

23h

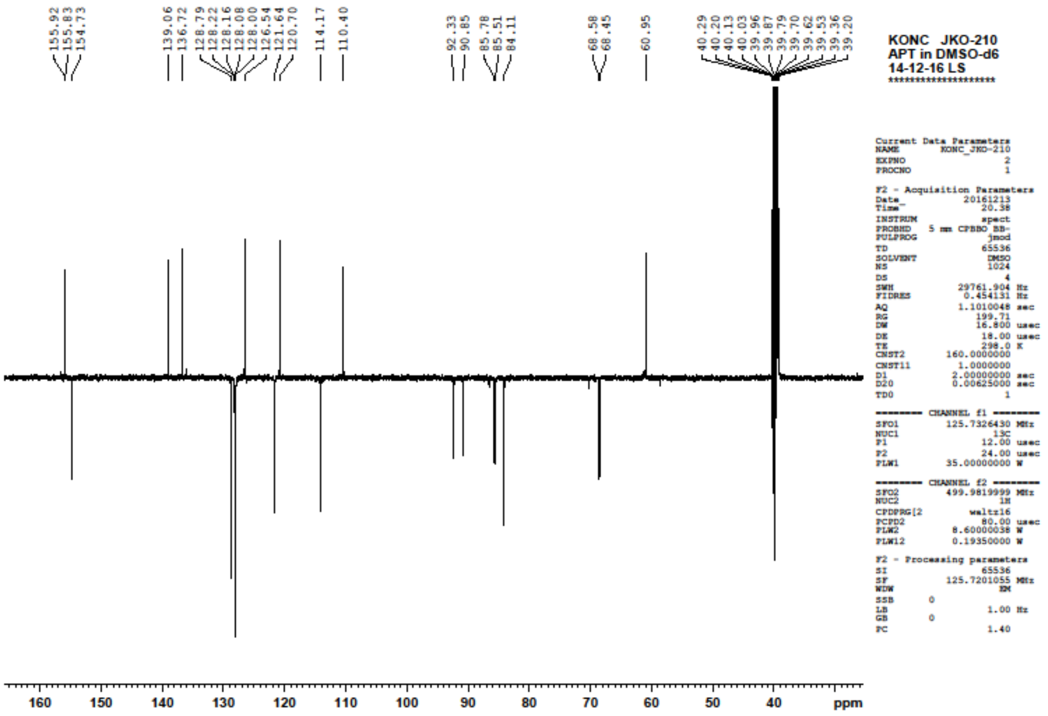
KONC JKO-210
APT in DMSO-d6
14-12-16 LS
155.92
155.83
154.73
139.06
136.72
128.79
128.22
128.16
128.08
128.00
126.54
121.64
120.70
114.17
110.40
92.33
90.85
85.78
85.51
84.11
68.58
68.45
60.95
40.29
40.20
40.13
40.03
39.96
39.87
39.79
39.70
39.62
39.53
39.36
39.20
ppm

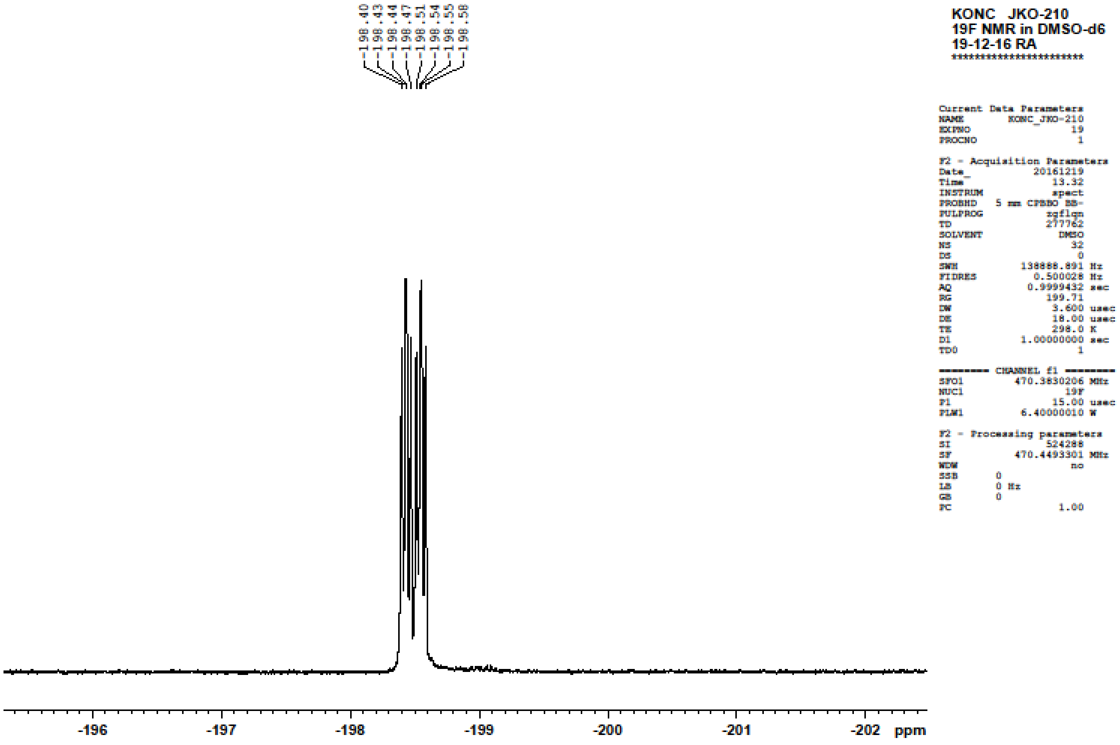
KONC JKO-210
19F NMR in DMSO-d6
19-12-16 RA
-198.40
-198.43
-198.44
-198.47
-198.51
-198.54
-198.55
-198.58
ppm

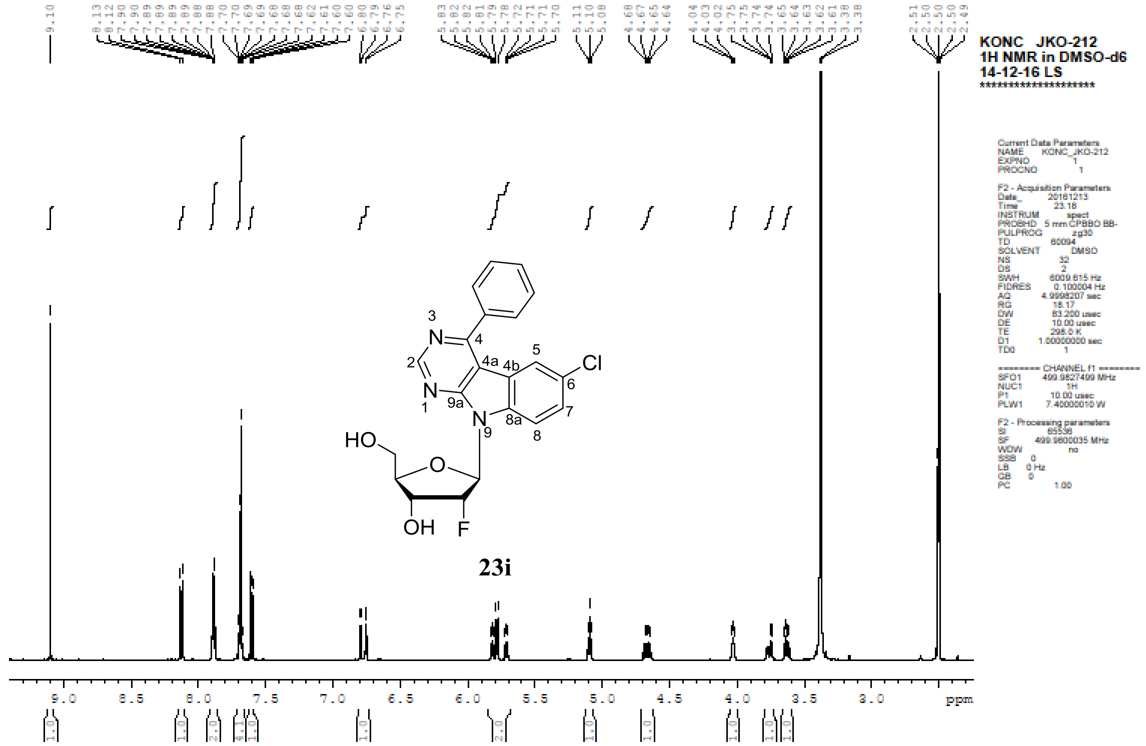
KONC JKO-212
1H NMR in DMSO-d6
14-12-16 LS
23i
ppm

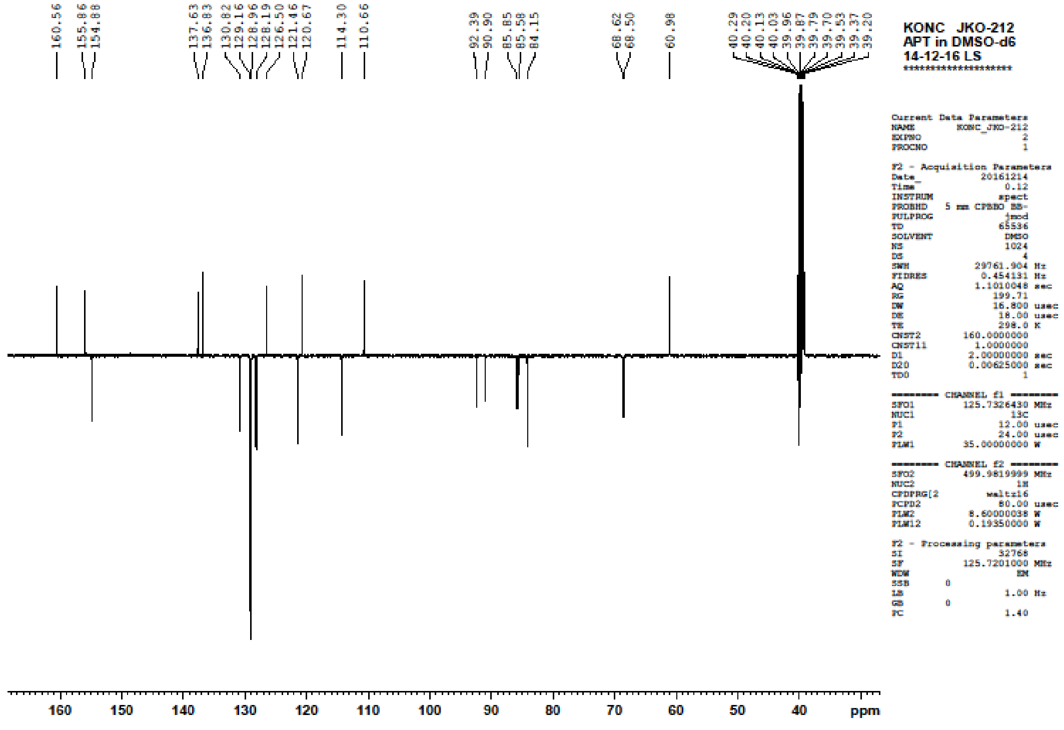
KONC JKO-212
APT in DMSO-d6
14-12-16 LS
ppm

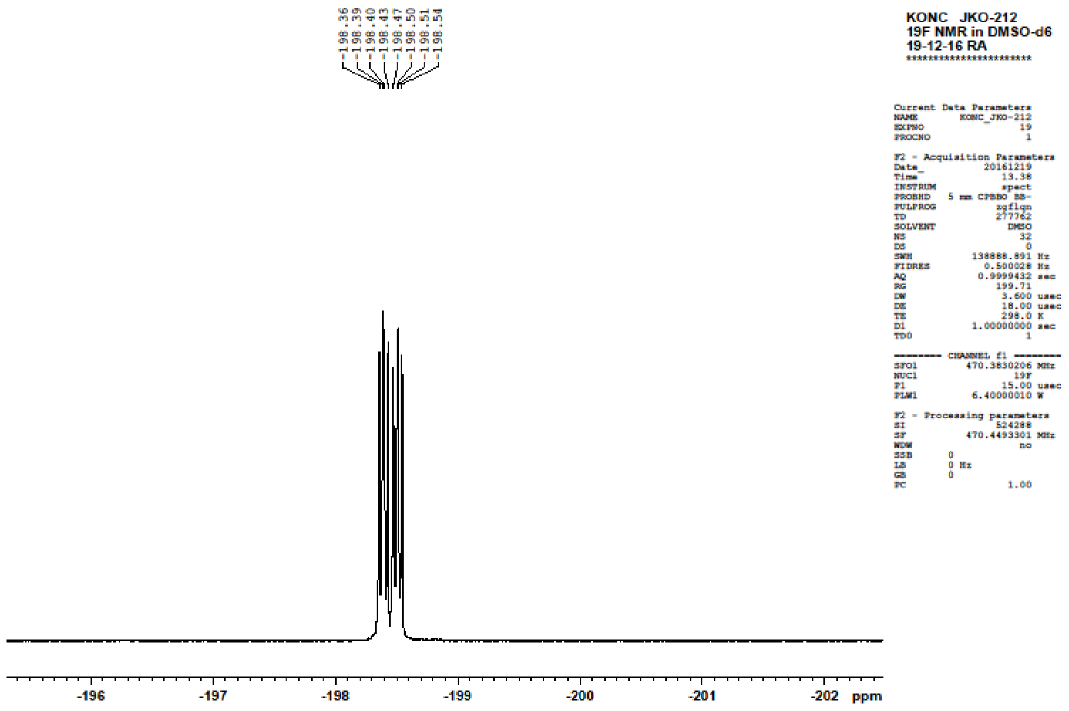
-198.36
-198.39
-198.40
-198.43
-198.47
-198.50
-198.51
-198.54
KONC JKO-212
19F NMR in DMSO-d6
19-12-16 RA
****
Current Data Parameters
NAME KONC_JKO-212
EXPNO 19
PROCNO 1
F2 - Acquisition Parameters
Date_ 20161219
Time 13.38
INSTRUM spect
PROBHD 5 mm CPBBO BB-
PULPROG zgflqn
TD 277762
SOLVENT DMSO
NS 32
DS 0
SWH 138888.891 Hz
FIDRES 0.500028 Hz
AQ 0.9999432 sec
RG 199.71
DW 3.600 usec
DE 18.00 usec
TE 298.0 K
D1 1.00000000 sec
TD0 1
======== CHANNEL f1 ========
SFO1 470.3830206 MHz
NUC1 19F
P1 15.00 usec
PLW1 6.40000010 W
F2 - Processing parameters
SI 524288
SF 470.4493301 MHz
WDW no
SSB 0
LB 0 Hz
GB 0
PC 1.00
-196
-197
-198
-199
-200
-201
-202
ppm

1) M. Tichý, R. Pohl, H. Y. Xu, Y. L. Chen, F. Yokokawa, P.-Y. Shi and M. Hocek, *Bioorg. Med. Chem.* **2012**, *20*, 6123–6133.

2) C. H. Tann, P. R. Brodfuehrer, S. P. Brundidge, C. Sapino Jr. and H. G. Howell, *J. Org. Chem.* **1985**, *50*, 3644–3647.

3) Y. A. Kim, A. Sharon, C. K. Chu, R. H. Rais, O. N. Al Safarjalani, F. N. M Naguib, and M. H. el Kouni, *J. Med. Chem.* **2008**, *51*, 3934–3945.

4) M. Tichý, R. Pohl, E. Tloušt'ová, J. Weber, G. Bahador, Y. J. Lee and M. Hocek, *Bioorg. Med. Chem.* **2013**, *21*, 5362–5372.

5) I. Mejdrová, D. Chalupská, M. Kögler, M. Šála, P. Plačková, A. Baumlová, H. Hřebabecký, E. Procházková, M. Dejmek, R. Guillon, D. Strunin, J. Weber, G. Lee, G. Birkus, H. Mertlíková-Kaiserová, E. Boura and R. Nencka, *J. Med. Chem.* **2015**, *58*, 3767–3793.

6) J. Y. Feng, G. Cheng, J. Perry, O. Barauskas, Y. Xu, M. Fenaux, S. Eng, N. Tirunagari, B. Peng, M. Yu, Y. Tian, Y. J. Lee, G. Stepan, L. L. Lagpacan, D. Jin, M. Hung, K. S. Ku, B. Han, K. Kitrinos, M. Perron, G. Birkus, K. A. Wong, W. Zhong, C. U. Kim, A. Carey, A. Cho and A. S. Ray, *Antimicrob. Agents Chemother.* **2014**, *58*, 1930–1942.

7) H. Yang, M. Robinson, A. C. Corsa, B. Peng, G. Cheng, Y. Tian, Y. Wang, R. Pakdaman, M. Shen, X. Qi, H. Mo, C. Tay, S. Krawczyk, X. C. Sheng, C. U. Kim, C. Yang and W. E. Delaney, *Antimicrob. Agents Chemother.* **2014**, *58*, 647–653.

8) W. E. Delaney, R. Edwards, D. Colledge, T. Shaw, J. Torresi, T. G. Miller, H. C. Isom, C. T. Bock, M. P. Manns, C. Trautwein and S. Locarnini, *Antimicrob. Agents Chemother.* **2001**, *45*, 1705–1713.

9) M. Perron, K. Stray, A. Kinkade, D. Theodore, G. Lee, E. Eisenberg, M. Sangi, B. E. Gilbert, R. Jordan, P. A. Piedra, G. L. Toms, R. Mackman and T. Cihlar, *Antimicrob. Agents Chemother.* **2016**, *60*, 1264–1273.

10) T. C. Appleby, J. K. Perry, E. Murakami, O. Barauskas, J. Feng, A. Cho, D. Fox, D. R. Wetmore, M. E. McGrath, A. S. Ray, M. J. Sofia, S. Swaminathan, T. E. Edwards, *Science* 2015, **347**, 771–775.

11) P. R. Gerber, K. J. Müller, *Comput.-Aided Mol. Des.* 1995, **9**, 251−268.
